# Enantioselective organocatalytic syntheses of α-selenated α- and β-amino acid derivatives†

**DOI:** 10.1039/d1ob02235k

**Published:** 2022-01-03

**Authors:** Victoria Haider, Paul Zebrowski, Jessica Michalke, Uwe Monkowius, Mario Waser

**Affiliations:** Institute of Organic Chemistry, Johannes Kepler University Linz Altenbergerstrasse 69 4040 Linz Austria mario.waser@jku.at; Institute of Catalysis, Johannes Kepler University Linz Altenbergerstrasse 69 4040 Linz Austria; School of Education, Chemistry, Johannes Kepler University Linz Altenbergerstrasse 69 4040 Linz Austria

## Abstract

Selenium-containing amino acids are valuable targets but methods for the stereoselective α-selenation of simple amino acid precursors are rare. We herein report the enantioselective electrophilic α-selenation of azlactones (masked α-amino acid derivatives) and isoxazolidin-5-ones (masked β-amino acids) using Cinchona alkaloids as easily accessible organocatalysts. A variety of differently substituted derivatives was accessed with reasonable levels of enantioselectivities and further studies concerning the stability and suitability of these compounds for further manipulations have been carried out as well.

## Abstract 2

The enantioselective electrophilic α-selenation of masked α- and β-amino acid derivatives has been carried under organocatalytic conditions in good yields and with moderate to good enantioselectivities.

## Graphical abstract

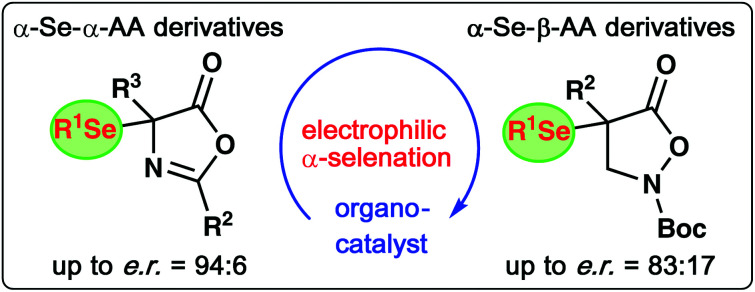

## Introduction

Non-natural α- and β-amino acids (AA) are compounds of high significance and value.^1–3^ Accordingly, the introduction of efficient and reliable strategies to access novel AA-derivatives in an enantioselective fashion has been a heavily investigated topic within the synthesis- and catalysis-oriented community.^2–6^ Hereby, the development of methods that allow for direct stereoselective functionalizations of simple (masked) α- or β-AA derivatives and precursors became an especially thoroughly explored field of research. More specifically, direct electrophilic α-functionalizations of suited (prochiral) AA-precursors emerged as versatile strategies to obtain valuable enantioenriched compounds straightforwardly. Besides asymmetric C–C bond formations, direct enantioselective C_α_-heteroatom forming approaches to access diversely decorated chiral α-heterofunctionalized α- and β-AA-derivatives have been investigated in much detail.^4^ While (enantioselective) syntheses of α-halogenated, α-aminated, α-sulfanylated, and to some extent also α-oxygenated, α- and β-AA-derivatives have been regularly reported,^4^ conceptually similar asymmetric α-selenations of prochiral (masked) α- and β-AA precursors are, to the best of our knowledge, so far still missing.^7^ Considering the high value of (chiral) organoselenium compounds for medical applications,^8–10^ as well as their use in (or for) stereoselective syntheses and catalysis approaches,^11^ and the importance of Se-containing AA in particular,^9^ the lack in generally applicable methods to access novel α-selenated α-AA (α-Se-α-AA) or β-AA derivatives (α-Se-β-AA) comes as a surprise.

**Scheme 1:**
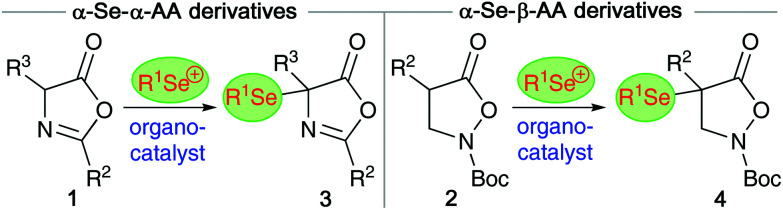
Targeted organocatalytic electrophilic α-selenation of masked α- and β-AA derivatives 1 and 2.

Over the last years, our group has had a fundamental interest in the development of organocatalytic protocols to access enantioenriched α-(hetero)-functionalized α- and β-AA derivatives.^12,13^ Inspired by the value of organoselenium compounds and considering the availability of established electrophilic Se-transfer reagents,^14^ we now became interested in developing organocatalytic protocols to control the asymmetric α-selenation of easily accessible azlactones 1 (as α-AA precursors)^6^ and isoxazolidin-5-ones 2 (as β-AA building blocks)^15,16^ (Scheme 1).^17^

## Results and discussion

**Fig. 1:**
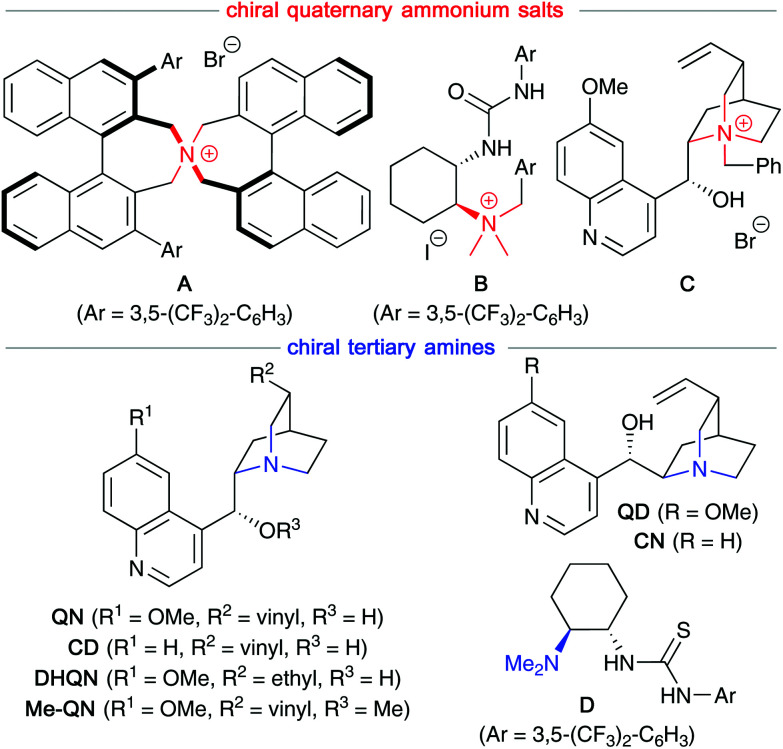
Chiral organocatalysts tested herein.

Based on our own recent experience in the field of asymmetric organocatalysis, we opted for the use of chiral quaternary ammonium salts (compounds A–C)^18^ and chiral organobases (*i.e.* Cinchona alkaloids^19^ and Takemoto's bifunctional thiourea-containing amine D;^20^Fig. 1) as catalysts for the herein investigated electrophilic α-selenation reactions.

### α-Se-α-AA derivatives

We started our investigations by optimizing the α-selenation of the parent azlactone 1a with the commercially available phthalimide-based PhSe-transfer agent 5a^14,21^ (Table 1 gives an overview of the most significant results obtained in an exhaustive screening of different organocatalysts and conditions). First attempts with the established chiral ammonium salt catalysts A–C^18^ were found to be discouraging, as illustrated by the non-selective examples summarized in entries 1–3. A variety of other ammonium salt derivatives and conditions/bases were tested as well, but racemic 3a was obtained in each case only. By switching to classical Cinchona alkaloids as chiral organobase catalysts next, results were more encouraging. First experiments utilizing the four naturally available alkaloids quinine (**QN**), quinidine (**QD**), cinchonine (**CN**), and cinchonidine (**CD**) gave promising results (entries 4–7), with **QN** and **QD** being more selective than their structural analogues **CD** and **CN**. Due to their pseudoenantiomeric relationship, the diastereomeric **QN** and **QD** gave access to both enantiomers of product 3a in an enantioenriched manner, albeit with a slightly higher enantioselectivity for the (−)-enantiomer (accessible by using **QN**; entry 4).

**Table 1:** Optimization of the organocatalytic asymmetric α-selenation of azlactone 1a^a^

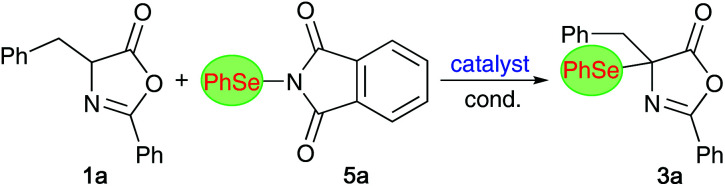						
Entry	Cat. (mol%)	Base	Solvent	*T* [°C]	Yield^b^ [%]	er^c^
1	A (10%)	K_3_PO_4_	Toluene	25	85	52 : 48
2	B (10%)	K_3_PO_4_	Toluene	25	34	51 : 49
3	C (10%)	K_3_PO_4_	Toluene	25	78	53 : 47
4	**QN** (10%)	—	Toluene	25	82	82 : 18
5	**QD** (10%)	—	Toluene	25	96	28 : 72
6	**CN** (10%)	—	Toluene	25	84	38 : 62
7	**CD** (10%)	—	Toluene	25	95	60 : 40
8	**QN** (10%)	—	CH_2_Cl_2_	25	92	78 : 22
9	**QN** (10%)	—	MTBE	25	98	50 : 50
10	**Me-QN** (10%)	—	Toluene	25	90	68 : 32
11	**DHQN** (10%)	—	Toluene	25	90	85 : 15
12	D (10%)	—	Toluene	25	68	50 : 50
13	**DHQN** (5%)	—	Toluene	25	97	85 : 15
14	**DHQN** (1%)	—	Toluene	25	85	62 : 38
15	**DHQN** (5%)	—	Toluene^d^	25	97	87 : 13
16	**DHQN** (5%)	—	Toluene^d^	0	92(94)^e^	89 : 11 (88 : 12)^e^
17	**DHQN** (5%)	—	Toluene^d^	−20	95	82 : 18

a Unless otherwise stated, reactions were run for 1 h using 0.05 mmol 1a and 0.055 mmol 5a in the presence of the given catalyst in the indicated solvent (*c* = 0.05 M with respect to 1a) under Ar and exclusion of light.

b Isolated yields.

c Determined by HPLC using a chiral stationary phase (given as (−)/(+)-enantiomeric ratio).

d *c* = 0.0125 (based on 1a).

e 1 mmol scale.

Comparing different solvents next (entries 4, 8, and 9), toluene was found to be superior to halogenated or ether-based solvents (other aromatic solvents did not allow for any improvement anymore). Modified quinine-derivatives were screened as well but, as exemplified for **Me-QN**, *O*-alkylation had a detrimental effect on the enantioselectivity (entry 10). Encouragingly however, dihydroquinine (**DHQN**) allowed for a slightly higher selectivity than **QN** (compare entries 11 and 4). Considering the beneficial effect of free OH-groups within the tested Cinchona alkaloids, we also used Takemoto's bifunctional thiourea-containing catalyst D,^20^ which however did not allow for any enantioinduction (entry 12).

Having identified **DHQN** as the best-suited (easily accessible) chiral organobase catalyst for the asymmetric synthesis of the masked α-Se-α-AA 3a, we carried out a final optimization with this alkaloid (entries 13–17). Lowering of the catalyst loading to 5 mol% was possible without negatively effecting the outcome (see entries 13 and 14) and a slightly higher dilution had a beneficial effect on the enantioselectivity (entry 15). With respect to the reaction temperature, 0 °C was found to be the optimum (entry 16), while lower temperatures had a detrimental effect on the selectivity again (entry 17). Gratifyingly, the reaction could also successfully be carried out under the optimized conditions on 1 mmol 1a scale (entry 16), substantiating the robustness of the protocol.

**Scheme 2:**
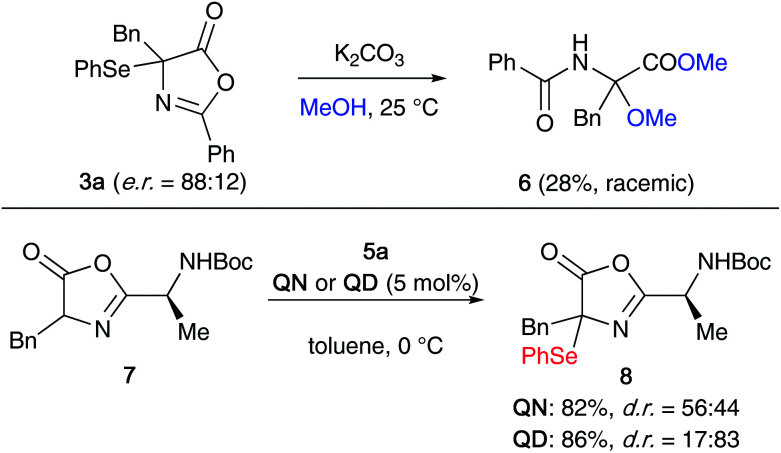
Attempted ring opening of 3a and direct α-selenation of the dipeptide-based azlactone 7.

One important observation that we made during this optimization process was that product 3a slowly decomposes when kept in solution, especially in the presence of light, by forming diphenyldiselenide. The stability is however significantly improved in the absence of light and the compound was found to be benchstable for several weeks when stored in substance under argon in the dark. With this obvious sensitivity noted, we next tested the suitability of compound 3a for further manipulations. Unfortunately however, we were not able to carry out the selective (nucleophilic) ring opening of this azlactone to analogous acyclic α-Se-α-AA derivatives, as compound 3a undergoes relatively rapid decomposition and deselenation reactions upon treatment with acid or base. For example, as outlined in the upper part of Scheme 2, the treatment of enantioenriched 3a with K_2_CO_3_ in MeOH lead to the formation of rac-6 in low yield only (accompanied with unidentified side-products). Opting for other bases and solvents turned out to be even worse and besides several not further specified decomposition products again the formation of diphenyldiselenide was observed.

As it was not possible to access more advanced α-Se-α-AA derivatives from 3a, we also explored the direct α-selenation of the dipeptide-based azlactone 7 (Scheme 2, lower reaction). Although it was possible to carry out this reaction with reasonable yields in the presence of either **QN** or **QD** (leading to opposite diastereoisomer preferences), product 8 was found to be very unstable and decomposed within a few hours when kept in solution.

**Scheme 3:**
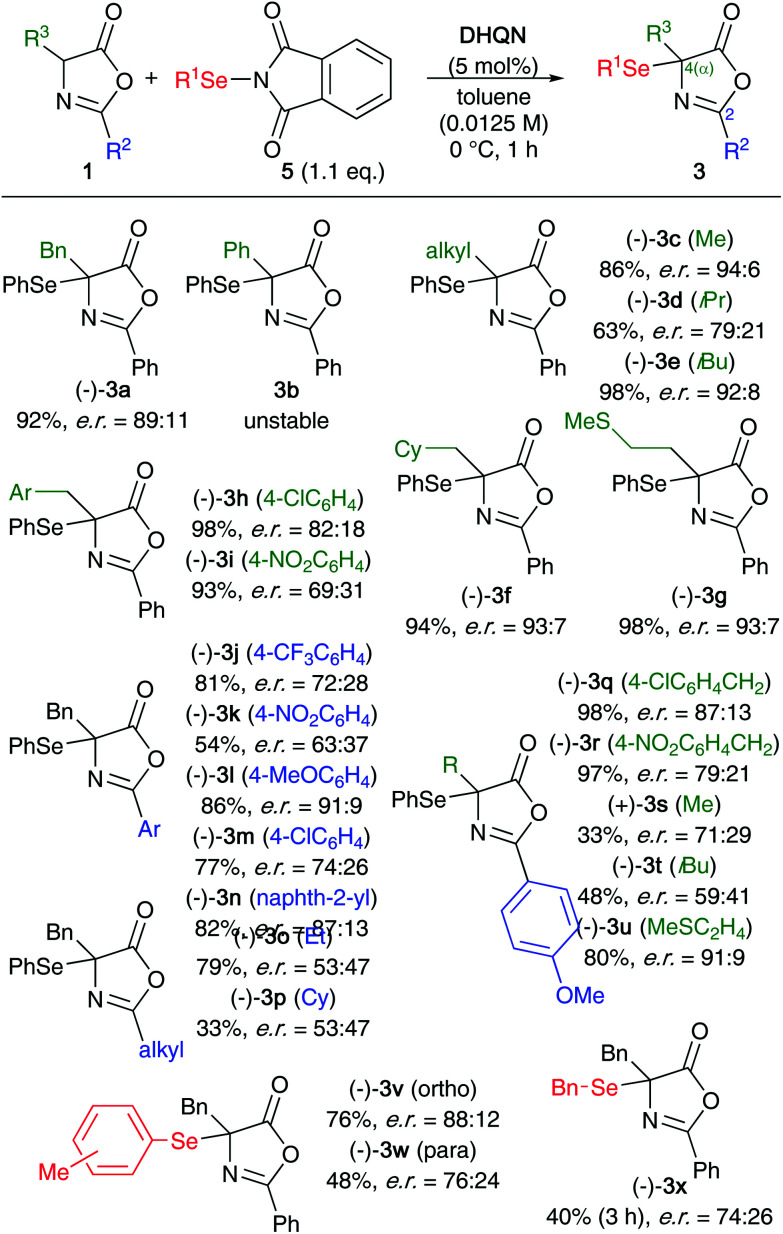
Application scope for the enantioselective α-selenation of azlactones 1.

Having identified suited conditions for the enantioselective α-selenation of 1a (entry 16, Table 1) and with a better understanding of the sensitivity of the hereby accessed product 3a at hand, we next investigated the application scope (Scheme 3) for a variety of differently substituted azlactones 1 (giving products 3a–u) as well as utilizing alternative phthalimide-based Se-transfer reagents 5 (products 3v–x). Unfortunately, the α-phenyl product 3b was found to be very sensitive and although its formation was indicated by crude product ^1^H NMR analysis, it was not possible to isolate this derivative at all because of its fast decomposition. In sharp contrast, the α-alkylated targets 3c–g were found to be more stable and could be obtained in satisfying isolated yields and with moderate to good enantioselectivities, depending on their substitution pattern (*i.e.* the presence of a bulky i-Pr group (3d) or a nitrobenzyl group (3i) had a negative effect on the enantioselectivity). Interesting effects were observed when varying the substituent in the 2-position of the azlactone skeleton (products 3j–p). While the presence of an electronrich methoxybenzene group (3l) had a positive effect on the enantioselectivity, electronpoor aryl substituents, *i.e.* –CF_3_ (3j) and –NO_2_ (3k), lead to significantly reduced selectivities. Moreover, the presence of alkyl substituents in this position resulted in more or less racemic product formation only (3o and 3p). Testing a few other 2-methoxybenzene containing substrates 1 with different residues in the α-position revealed a complex scenario of substituent effects. While the products 3q and 3r could be obtained with higher enantioselectivities than their 2-phenyl analogues 3h and 3i, the presence of an isobutyl group had a detrimental effect hereby (3t*vs.*3e).

A few alternative selenation reagents were prepared as well^22^ and tested herein (products 3v–x), showing a rather pronounced dependency of the overall reaction progress and the enantioselectivity based on the nature of these reagents.

### α-Se-β-AA derivatives

Having investigated the α-selenation of the masked α-AA compounds 1, we next put our focus on the cyclic β-AA derivatives 2 (Table 2). We started again by using chiral ammonium salt catalysts for the α-selenation of the parent substrate 2a with the phthalimide-based reagent 5a (entries 1–3). Surprisingly, although Maruoka's catalyst A was found to be very well-suited for asymmetric α-(hetero)functionalizations of compounds 2 in the past,^12,13,16^ only low levels of enantioselectivities could be achieved for the α-selenation of 2a (entry 1 gives the best result obtained after screening a variety of different conditions). The alternative ammonium salts B and C as well as the bifunctional catalyst D were even less promising (entries 2–4) and we thus tested Cinchona alkaloids next. Interestingly, these chiral amines have so far only sparingly been used for asymmetric α-functionalizations of compounds 2^23^ and we were glad to see that they allowed for promising initial levels of enantioselectivities accompanied with reasonable yields under conditions similar to those used for azlactones 1. Again, **QN** and **DHQN** performed slightly better than the other derivatives (entries 5–8) and the reaction was found to be carried out best in toluene in the presence of 10 mol% catalyst at temperatures between 0–25 °C (please see entries 9–14 for the optimization of conditions with **QN** as a catalyst). Interestingly, the enantioselectivity could be improved to some extent when using the succinimide-based reagent 5b instead of 5a (entry 15).^24^ Finally, **DHQN** was used with 5b under slightly diluted conditions, resulting in robust and scalable conditions allowing for moderate enantioselectivities (entry 17; in general, the reaction is complete within 2 h but can also be run overnight to ensure full conversion, *i.e.* for the larger scale experiment and for some other derivatives (*vide infra*)).

**Table 2:** Optimization of the organocatalytic asymmetric α-selenation of isoxazolidin-5-one 2a^a^

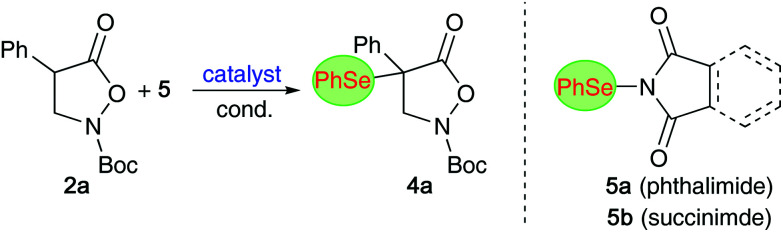							
Entry	Cat.	5	Solvent	*t* [h]	*T* [°C]	Yield^b^ [%]	er^c^
1	A (5%)^d^	5a	Toluene	20	25	37	62 : 38
2	B (5%)^d^	5a	Toluene	20	25	48	51 : 49
3	C (5%)^d^	5a	Toluene	20	25	47	54 : 46
4	D (5%)	5a	Toluene	2	25	81	51 : 49
5	**QN** (10%)	5a	Toluene	2	25	68	75 : 25
6	**QD** (10%)	5a	Toluene	2	25	65	32 : 68
7	**CD** (10%)	5a	Toluene	2	25	65	73 : 27
8	**DHQN** (10%)	5a	Toluene	2	25	73	76 : 24
9	**QN** (5%)	5a	Toluene	2	25	43	69 : 31
10	**QN** (10%)	5a	CH_2_Cl_2_	2	25	76	69 : 31
11	**QN** (10%)	5a	MTBE	2	25	76	61 : 39
12	**QN** (10%)	5a	Toluene	2	0	82	75 : 25
13	**QN** (10%)	5a	Toluene	2	−20	76	75 : 25
14	**QN** (10%)	5a	Toluene^e^	2	25	78	76 : 24
15	**QN** (10%)	5b	Toluene	2	25	74	81 : 19
16	**DHQN** (10%)	5b	Toluene^e^	2	25	72	83 : 17
17	**DHQN** (10%)	5b	Toluene^e^	14	0–25	69(72)^f^	83 : 17(83 : 17)^f^

a Unless otherwise stated, reactions were run using 0.05 mmol 2a and 0.055 mmol 5 in the presence of the given catalyst in the indicated solvent (*c* = 0.05 M with respect to 2a) under Ar and exclusion of light.

b Isolated yields.

c Determined by HPLC using a chiral stationary phase (given as (+)/(−)-enantiomeric ratio).

d With 1.1 equiv. K_2_CO_3_.

e *c* = 0.025 based on 2a.

f 0.8 mmol scale.

Interestingly, it was also possible to increase the enantiopurity of product 4a up to er = 98 : 2 by slowly crystallizing racemic 4a out of a solution of enantioenriched 4a (er = 83 : 17) in Et_2_O.^25^

**Scheme 4:**
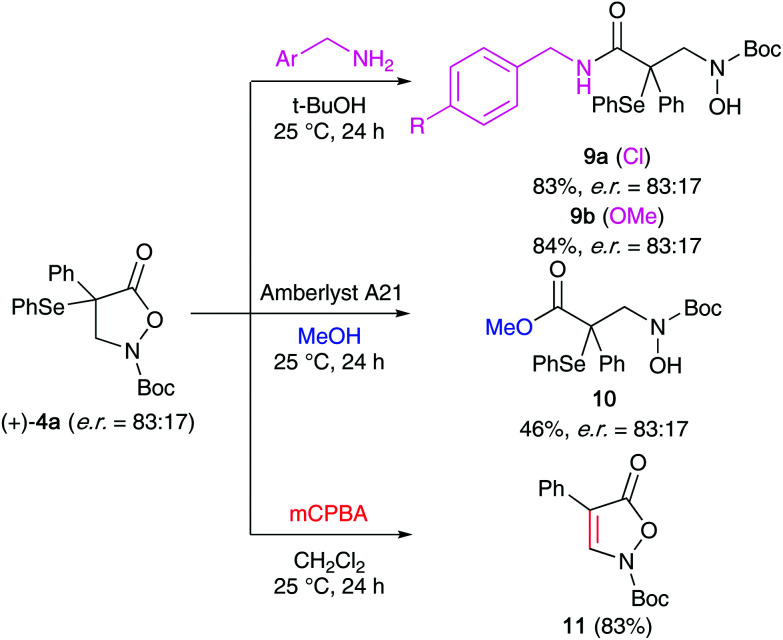
Further manipulations of compound 4a.

We next tested the suitability of compound 4a for the transformations outlined in Scheme 4. Gratifyingly, the nucleophilic ring opening with benzylic amines (giving amides 9) as well as with MeOH in the presence of Amberlyst A21 (accessing ester 10) proceeded reasonably well without causing any erosion of er. The treatment of 4a with mCPBA on the other hand led to a direct elimination reaction, most presumably *via syn*-elimination of the *in situ* formed selenoxide-species, which allows for the straightforward synthesis of alkene 11 hereby.

**Scheme 5:**
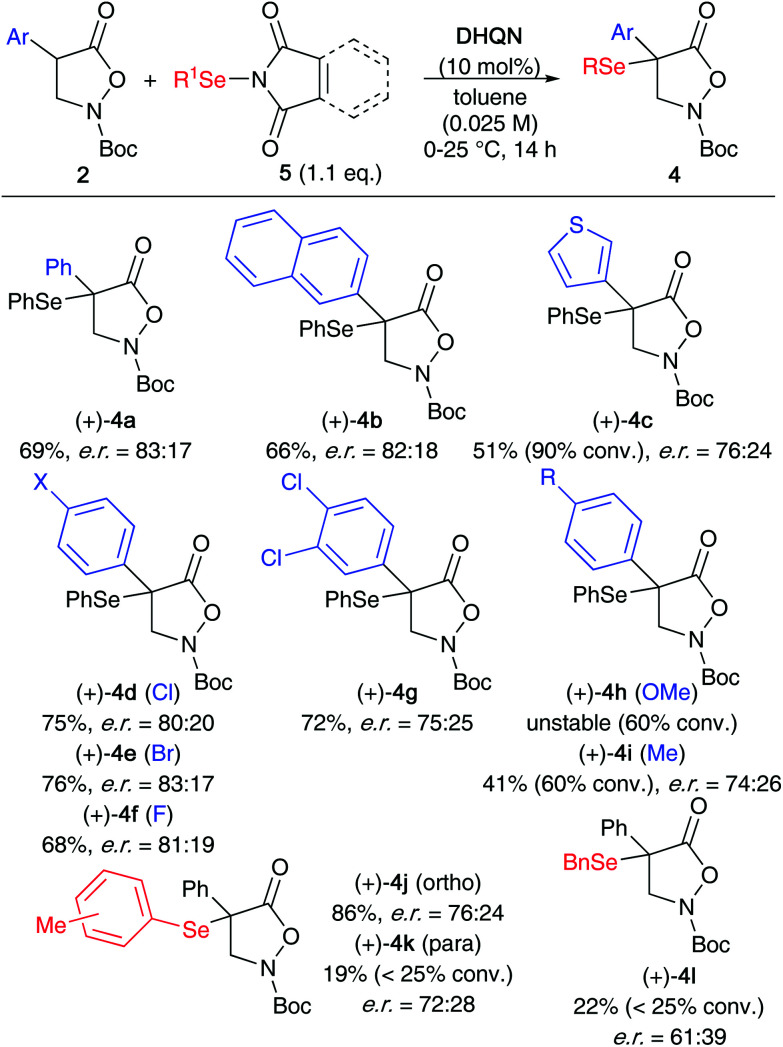
Application scope for the enantioselective α-selenation of isoxazolidinones 2.

Finally, we also investigated the application scope for the **DHQN**-catalysed α-selenation of a variety of differently substituted α-arylated derivatives 2 (Scheme 5). It should be noted that we also tested the analogous α-benzylated substrate (Bn instead of Ar). Unfortunately however, and in accordance with our previous α-halogenation results,^13*b*^ this compound was found to be much less reactive than the α-aryl analogues, giving traces of targeted product only. Furthermore, we also observed a strong influence of the nature of the aryl substituent (Ar) on the outcome of the reaction in terms of yield, conversion, and enantioselectivity. The naphthalene-based 4b as well as the halogenated products 4d–4g could be obtained with complete conversion of the respective starting materials 2 and with moderate levels of enantioselectivities. In contrast, the thiophene-substituted 4c as well as the MeO- and the Me-containing 4h and 4i were formed much slower and in these cases the enantioselectivities were also lower (4h could not be isolated at all because of its low stability). Also, the use of alternative Se-transfer reagents (accessing products 4j–l) was found to be difficult herein, illustrating that our protocol for the α-selenation of compounds 2 is unfortunately rather sensitive to substituent effects.

## Conclusions

Using simple Cinchona alkaloids as chiral organobase catalysts allowed for the, to the best of our knowledge so far unprecedented, enantioselective electrophilic α-selenation of azlactones 1 (which were used as masked α-amino acid derivatives) and isoxazolidin-5-ones 2 (representing masked β-amino acids). Utilizing the herein developed methodology gave access to a variety of differently substituted α-Se-amino acid derivatives 3 and 4 with reasonable levels of enantioselectivities and in good yields. Unfortunately, it was not possible to obtain suited crystals of enantioenriched 3 and 4 that allowed for single crystal X-ray analysis and therefore the absolute configuration of the products accessed herein remains yet unknown.

Further studies concerning the stability and suitability of these compounds for further manipulations have been carried out as well, demonstrating a certain sensitivity, especially in the presence of light and/or in solution.

## Experimental details

Full experimental procedures as well as analytical details can be found in the online ESI.†

### General procedure for the α-selenation of azlactones 1

To a solution of 1 (1 equiv.) and **DHQN** (5 mol%) in toluene (0.0125 M with respect to 1) under an atmosphere of argon was added the corresponding Se-transfer agent 5 (1.1 equiv.) at 0 °C and under exclusion of light. The reaction mixture was stirred for 1 h under these conditions. After filtration through a pad of Na_2_SO_4_ the crude product was subjected to silica gel column chromatography (eluent: heptanes : EtOAc gradient) giving products 3 in the report yields and enantiopurities (compare with Scheme 3).

### Analytical details of product 3a

Obtained as a white amorphous solid in 92% yield with er = 89 : 11. TLC (heptanes : EtOAc = 3.5 : 1): *R*_f_ = 0.49. [*α*]^24^_D_ = −65.1 (*c* 1.00, CHCl_3_). ^1^H-NMR (300 MHz, CDCl_3_, 298.0 K): *δ*/ppm = 7.57 (d, *J* = 9.0 Hz, 2H), 7.50 (d, *J* = 6.0 Hz, 2H), 7.39 (t, *J* = 9.0 Hz, 1H), 7.26 (t, *J* = 9.0 Hz, 2H), 7.19–7.14 (m, 4H), 7.12–7.02 (m, 4H), 3.54 (d, *J* = 12.0 Hz, 1H), 3.42 (d, *J* = 15.0 Hz, 1H). ^13^C-NMR (75 MHz, CDCl_3_, 298.0 K): *δ*/ppm = 176.4, 160.7, 138.3, 134.6, 132.8, 130.2, 130.2, 129.2, 128.7, 128.5, 127.9, 127.6, 125.3, 73.9, 41.3. HRMS (ESI-QTOF, MeOH) *m*/*z*: [M + H]^+^ calculated for C_23_H_17_NO_2_Se, 408.0498; found, 408.0500. HPLC: Chiralpak AD-H (*n*-hexane : i-PrOH = 20 : 1, flow rate 0.3 mL min^−1^, 10 °C, *λ* = 220 nm), retention times *t*_R_(minor) = 26.4 min, *t*_R_(major) = 27.8 min.

### General procedure for the α-selenation of isoxazolidinones 2

A mixture of 2 (1 equiv.), **DHQN** (10 mol%) and dry toluene (0.025 M with respect to 2) was stirred under Ar and cooled on an icebath. The selenation reagent 5 (1.1–1.2 equiv.) was added at once and the reaction flask was covered with aluminium foil. The reaction mixture was gradually warmed to room temperature and stirred for 14 h, whereupon it was concentrated under reduced pressure and directly subjected to column chromatography with gradient elution (silica gel, heptanes : EtOAc = 1 : 0 to 10 : 1) to obtain products 4 in the given yields and enantiopurities (compare with Scheme 5).

### Analytical details of compound 4a

Obtained in 72% yield with er = 83 : 17. TLC (EtOAc : heptanes = 10 : 1): *R*_f_ = 0.24 (UV). [*α*]^23^_D_ = +90.2 (*c* 1.01, CHCl_3_). ^1^H-NMR (300 MHz, CDCl_3_, 298.0 K): *δ*/ppm = 7.41–7.34 (m, 3H), 7.30–7.18 (m, 7H), 4.75 (d, *J* = 12.7 Hz, 1H), 4.34 (d, *J* = 12.7 Hz, 1H), 1.56 (s, 9H). ^13^C-NMR (75 MHz, CDCl_3_, 298.0 K): *δ*/ppm = 172.1, 156.6, 138.3, 135.7, 130.6, 129.3, 128.8, 128.6, 127.5, 126.3, 84.7, 59.5, 49.3, 28.4. HRMS (ESI-QTOF, MeOH) *m*/*z*: [M + NH_4_]^+^ calculated for C_20_H_25_N_2_O_4_Se^+^, 437.0974; found, 437.0973. HPLC: Chiralpak AD-H (*n*-hexane : i-PrOH = 20 : 1, flow rate 1.0 mL min^−1^, 20 °C, *λ* = 254 nm), retention times *t*_R_(minor) = 9.7 min, *t*_R_(major) = 11.0 min.

## Author contributions

V. Haider and P. Zebrowski contributed equally to the experimental work as well as the preparation of the manuscript. J. Michalke carried out additional supporting experiments. U. Monkowius carried out single crystal X-ray diffraction analysis. M. Waser initiated and supervised the project and wrote the manuscript with input from all authors. All authors have given approval to the final version.

## Conflicts of interest

There are no conflicts to declare.

## Supplementary Material

OB-020-D1OB02235K-s001

OB-020-D1OB02235K-s002
