# Supplementary material for: Enantioselective organocatalytic syntheses of α-selenated α- and β-amino acid derivatives
Source: Org Biomol Chem. 2022 Jan 3;20(4):824–30. doi: 10.1039/d1ob02235k (PMC8790592; doi:10.1039/d1ob02235k)

Electronic Supplementary Material (ESI) for Organic & Biomolecular Chemistry.
This journal is © The Royal Society of Chemistry 2022

SUPPORTING INFORMATION

Enantioselective organocatalytic syntheses of α-selenated α- and β-amino acid derivatives

Victoria Haider[a], Paul Zebrowski[a], Jessica Michalke[b], Uwe Monkowius[c] and Mario Waser*[a]

a) Institute of Organic Chemistry, Johannes Kepler University Linz, Altenbergerstraße 69, 4040 Linz, Austria.

Fax: +43 732 2468 5411; Tel: +43 732 2468 5402;

E-mail: mario.waser@jku.at

b) Institute of Catalysis, Johannes Kepler University Linz, Altenbergerstraße 69, 4040 Linz, Austria.

c) School of Education, Chemistry, Johannes Kepler University Linz, Altenbergerstraße 69, 4040 Linz, Austria.

Table of Contents

1. General Information 1

2. Synthesis of α-Selenated Azlactones 3 2

   2.1 General Racemic Procedure 2

   2.2 General Enantioselective Procedure 2

3. Characterization Data of α-Selenated Azlactones 3 3

   4-Benzyl-2-phenyl-4-(phenylselanyl)oxazol-5(4*H*)-one (3a): 3

   4-Methyl-2-phenyl-4-(phenylselanyl)oxazol-5(4*H*)-one (3c): 3

   4-Isopropyl- 2-phenyl-4-(phenylselanyl)oxazol-5(4*H*)-one (3d): 4

   4-Isobutyl- 2-phenyl-4-(phenylselanyl)oxazol-5(4*H*)-one (3e): 4

   4-(Cyclohexylmethyl)-2-phenyl-4-(phenylselanyl)oxazol-5(4*H*)-one (3f): 5

   4-(2-(Methylthio)ethyl)-2-phenyl-4-(phenylselanyl)oxazol-5(4*H*)-one (3g): 5

   4-(4-Chlorobenzyl)- 2-phenyl-4-(phenylselanyl)oxazol-5(4*H*)-one (3h): 6

   4-(4-Nitrobenzyl)- 2-phenyl-4-(phenylselanyl)oxazol-5(4*H*)-one (3i): 6

   4-Benzyl-4-(phenylselanyl)-2-(4-(trifluoromethyl)phenyl)oxazol-5(4*H*)-one (3j): 7

   4-Benzyl-2-(4-nitrophenyl)-4-(phenylselanyl)oxazol-5(4*H*)-one (3k): 7

   4-Benzyl-2-(4-methoxyphenyl)-4-(phenylselanyl)oxazol-5(4*H*)-one (3l): 8

   4-Benzyl-2-(4-chlorophenyl)-4-(phenylselanyl)oxazol-5(4*H*)-one (3m): 8

   4-Benzyl-2-(naphthalen-2-yl)-4-(phenylselanyl)oxazol-5(4*H*)-one (3n): 9

   4-Benzyl-2-ethyl-4-(phenylselanyl)oxazol-5(4*H*)-one (3o): 9

   4-Benzyl-2-cyclohexyl-4-(phenylselanyl)oxazol-5(4*H*)-one (3p): 10

   4-(4-Chlorobenzyl)-2-(4-methoxyphenyl)-4-(phenylselanyl)oxazol-5(4*H*)-one (3q): 10

   2-(4-Methoxyphenyl)-4-(4-nitrobenzyl)-4-(phenylselanyl)oxazol-5(4*H*)-one (3r): 11

   2-(4-Methoxyphenyl)-4-methyl-4-(phenylselanyl)oxazol-5(4*H*)-one (3s): 11

   4-Isobutyl-2-(4-methoxyphenyl)-4-(phenylselanyl)oxazol-5(4*H*)-one (3t): 12

   2-(4-Methoxyphenyl)-4-(2-(methylthio)ethyl)-4-(phenylselanyl)oxazol-5(4*H*)-one (3u): 12

   4-Benzyl-2-phenyl-4-(*o*-tolylselanyl)oxazol-5(4*H*)-one (3v): 13

   4-Benzyl-2-phenyl-4-(*p*-tolylselanyl)oxazol-5(4*H*)-one (3w): 13

   4-Benzyl-4-(benzylselanyl)-2-phenyloxazol-5(4*H*)-one (3x): 14

   tert-Butyl- ((1*S*)-1-(4-benzyl-5-oxo-4-(phenylselanyl)-4,5-dihydrooxazol-2-yl)ethyl)carbamate (8): 14

4. Further Transformations of 4-Benzyl-2-phenyl-4-(phenylselanyl)oxazol-5(4*H*)-one 3a 15

   Methyl 2-benzamido-2-methoxy-3-phenylpropanoate (6): 15

5. Synthesis of α-Selenated α-Aryl-Isoxazolidin-5-ones 4 16

   5.1 General Racemic Procedure 16

   5.2 General Enantioselective Procedure 16

6. Characterization Data of α-Selenated α-Aryl-Isoxazolidin-5-ones 4 17

tert-Butyl 5-oxo-4-phenyl-4-(phenylselanyl)isoxazolidine-2-carboxylate (4a): 17
tert-Butyl 4-(naphthalen-2-yl)-5-oxo-4-(phenylselanyl)isoxazolidine-2-carboxylate (4b): 17
tert-Butyl 5-oxo-4-(phenylselanyl)-4-(thiophen-3-yl)isoxazolidine-2-carboxylate (4c): 18
tert-Butyl 4-(4-chlorophenyl)-5-oxo-4-(phenylselanyl)isoxazolidine-2-carboxylate (4d): 18
tert-Butyl 4-(4-bromophenyl)-5-oxo-4-(phenylselanyl)isoxazolidine-2-carboxylate (4e): 19
tert-Butyl 4-(4-fluorophenyl)-5-oxo-4-(phenylselanyl)isoxazolidine-2-carboxylate (4f): 19
tert-Butyl 4-(3,4-dichlorophenyl)-5-oxo-4-(phenylselanyl)isoxazolidine-2-carboxylate (4g): ... 20
tert-Butyl 5-oxo-4-(phenylselanyl)-4-(*p*-tolyl)isoxazolidine-2-carboxylate (4i): 20
tert-Butyl 5-oxo-4-phenyl-4-(*o*-tolylselanyl)isoxazolidine-2-carboxylate (4j): 21
tert-Butyl 5-oxo-4-phenyl-4-(*p*-tolylselanyl)isoxazolidine-2-carboxylate (4k): 21
tert-Butyl 4-(benzylselanyl)-5-oxo-4-phenylisoxazolidine-2-carboxylate (4l): 22

7. Further Transformations of *N*-Boc 4,4-phenyl(phenylselanyl)isoxazolidin-5-one 4a 23

tert-Butyl (3-((4-chlorobenzyl)amino)-3-oxo-2-phenyl-2-(phenylselanyl)propyl)(hydroxy)carbamate (9a): 23
tert-Butyl hydroxy(3-((4-methoxybenzyl)amino)-3-oxo-2-phenyl-2-(phenylselanyl)propyl)carbamate (9b): 23
Methyl 3-((*tert*-butoxycarbonyl)(hydroxy)amino)-2-phenyl-2-(phenylselanyl)propanoate (10): 24
tert-Butyl 5-oxo-4-phenylisoxazole-2(5*H*)-carboxylate (11): 24

8. Single-Crystal Analysis 25

9. NMR Spectra of Selenation Products 27

10. HPLC Traces of Racemic and Enantioenriched Selenation Products 69

11. HRMS Data 107

1. General Information

^{1}H-, ^{13}C- and ^{19}F-NMR spectra were recorded on a Bruker Avance III 300 MHz spectrometer with a broad band observe probe and a sample changer for 16 samples, a Bruker Avance DRX 500 MHz spectrometer and on a Bruker Avance III 700 MHz spectrometer with an Ascend magnet and TCI cryoprobe, which are property of the Austro-Czech NMR-Research Center "RERI-uasb". NMR spectra were referenced on the solvent peak and chemical shifts are given in ppm.

High resolution mass spectra were obtained using an Agilent 6520 Q-TOF mass spectrometer with an ESI source. Analyses were made in the positive ionization mode if not otherwise stated. Purine (exact mass for $[M+H]^+$ = 121.050873) and 1,2,3,4,5,6-hexakis(2,2,3,3-tetrafluoropropoxy)-1,3,5,2,4,6-triazatriphosphinane (exact mass for $[M+H]^+$ = 922.009798) were used for internal mass calibration.

HPLC was performed using a Thermo Scientific Dionex Ultimate 3000 or a Shimadzu Prominence system with diode array detector with a CHIRALPAK AD-H, OD-H or YMC CHIRAL ART Cellulose-SB (250 × 4.6 mm, 5 µm) chiral stationary phase. Optical rotations were recorded on a Schmidt + Haensch Polarimeter Model UniPol L1000 at 589 nm.

All chemicals were purchased from commercial suppliers and used without further purification unless otherwise stated. Dry solvents were obtained from an MBraun-SPS-800 solvent purification system. All reactions were carried out under argon atmosphere and in absence of light, unless stated otherwise.

Azlactones[1] **1**, α-Aryl-Isoxazolidin-5-ones[2] **2** and selenation reagents[3] **5** were prepared following established procedures.

[1] (a) C. Macovai, P. Vicennati, J. Quinton, M.-C. Nevers, H. Volland, C. Créminon and F. Taran, *Chem. Commun.* 2012, **48**, 4411-4413; (b) A. D. Melhado, M. Luparia and F. D. Toste, *J. Am. Chem. Soc.* 2007, **129**, 42, 12638-12639; (c) D. N. Le, J. Riedel, N. Kozlyuk, R. W. Martin and V. M. Dong, *Org. Lett.* 2017, **19**, 1, 114-117
[2] M. N. Oliveira, S. Arseniyadis and J. Cossy, *Chem. Eur. J.* 2018, **24,** 4810–4814.
[3] (a) X.-Y. Wang, Y.-F. Zhong, Z.-Y. Mo, S.-H. Wu, Y.-L. Xu, H.-T. Tang and Y.-M. Pan, *Adv. Synth. Catal.* 2021, **363,** 208–214; (b) T. Hori and K. B. Sharpless, *J. Org. Chem.* 1979, **44,** 4208–4210.

2. Synthesis of α-Selenated Azlactones 3

2.1 General Racemic Procedure

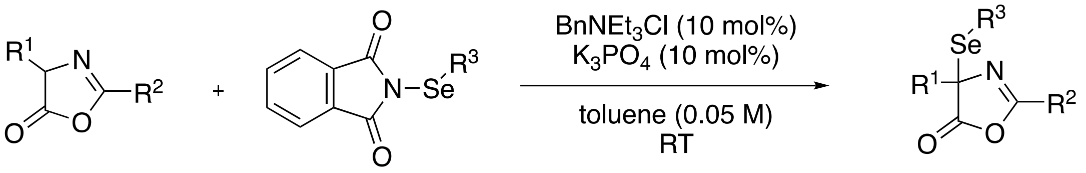

1 1.1 equiv. (*rac*)-**3**

To a solution of azlactone (**1**, 1 equiv.), benzyltriethylammonium chloride (BTEAC, 10 mol%) and K_3PO_4 (10 mol%) in toluene (0.05 M with respect to azlactone substrate **1**) under an atmosphere of argon was added the corresponding *N*-(Aryl-seleno)phthalimide (1.1 equiv.) at room temperature and under exclusion of light. The reaction mixture was stirred for 1 h under these conditions and the reaction was monitored by thin layer chromatography using heptanes:EtOAc (3.5:1) as mobile phase. After filtration through a pad of Na_2SO_4 the crude product was subjected to silica gel column chromatography (eluent: heptanes:EtOAc) for purification.

2.2 General Enantioselective Procedure

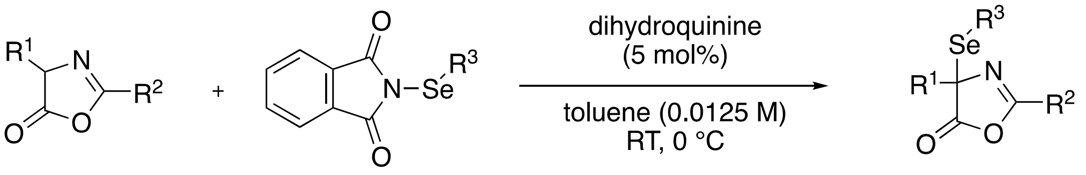

1 1.1 equiv. **3**

To a solution of azlactone (**1**, 1 equiv.) and dihydroquinine (5 mol%) in toluene (0.0125 M related to azlactone **1**) under an atmosphere of argon was added the corresponding *N*-(Aryl-seleno)phthalimide (1.1 equiv.) at 0 °C and under exclusion of light. The reaction mixture was stirred for 1 h under these conditions and the reaction was monitored by thin layer chromatography using heptanes:EtOAc (3.5:1) as mobile phase. After filtration through a pad of Na_2SO_4 the crude product was subjected to silica gel column chromatography (eluent: heptanes:EtOAc) for purification.

3. Characterization Data of α-Selenated Azlactones 3

4-Benzyl-2-phenyl-4-(phenylselanyl)oxazol-5(4*H*)-one (3a):

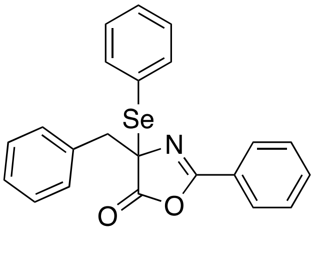

3a

Compound **3a** was prepared according to the general procedure described in **2.2** and was obtained as a white solid (18.7 mg, 46 μmol, 92% yield, e.r. = 89:11). **TLC** (heptanes:EtOAc = 3.5:1): R_f = 0.49 (UV). $[\alpha]_D^{24}$ = -65.1 (*c* 1.00, $CHCl_3$, e.r. = 89:11). **^{1}H-NMR** (300 MHz, $CDCl_3$, 298.0 K): δ / ppm = 7.57 (d, *J* = 9.0 Hz, 2H), 7.50 (d, *J* = 6.0 Hz, 2H), 7.39 (t, *J* = 9.0 Hz, 1H), 7.26 (t, *J* = 9.0 Hz, 2H), 7.19-7.14 (m, 4H), 7.12-7.02 (m, 4H), 3.54 (d, *J* = 12.0 Hz, 1H), 3.42 (d, *J* = 15.0 Hz, 1H). **^{13}C-NMR** (75 MHz, $CDCl_3$, 298.0 K): δ / ppm = 176.4, 160.7, 138.3, 134.6, 132.8, 130.2, 130.2, 129.2, 128.7, 128.5, 127.9, 127.6, 125.3, 73.9, 41.3. **HRMS** (ESI-QTOF, MeOH) *m/z*: $[M+H]^+$ calculated for $C_{23}H_{17}NO_2Se$, 408.0498; found, 408.0500. **HPLC**: Chiralpak AD-H (*n*-hexane:*i*-PrOH = 20:1, flow rate 0.3 mL/min, 10 °C, λ = 220 nm), retention times t_R(minor) = 26.4 min, t_R(major) = 27.8 min.

4-Methyl-2-phenyl-4-(phenylselanyl)oxazol-5(4*H*)-one (3c):

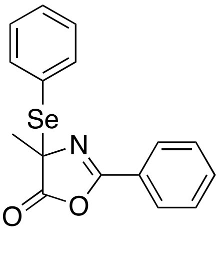

3c

Compound **3c** was prepared according to the general procedure described in **2.2** and was obtained as a white solid (14.2 mg, 43 μmol, 86% yield, e.r. = 94:6). **TLC** (heptanes:EtOAc = 3.5:1): R_f = 0.57 (UV). $[\alpha]_D^{24}$ = -30.1 (*c* 1.00, $CHCl_3$, e.r. = 94:6). **^{1}H-NMR** (700 MHz, $CDCl_3$, 298.0 K): δ / ppm = 7.76 (d, *J* = 7.0 Hz, 2H), 7.57 (d, *J* = 14.0 Hz, 2H), 7.53 (t, *J* = 14.0 Hz, 1H), 7.40 (t, *J* = 7.0 Hz, 2H), 7.25 (t, *J* = 7.0 Hz, 1H), 7.14 (t, *J* = 7.0 Hz, 2H), 1.93 (s, 3H). **^{13}C-NMR** (176 MHz, $CDCl_3$, 298.0 K): δ / ppm = 177.7, 160.6, 138.1, 133.0, 130.2, 129.1, 128.8, 127.9, 125.8, 125.3, 69.2, 21.9. **HRMS** (ESI-QTOF, MeOH) *m/z*: $[M+H]^+$ calculated for $C_{16}H_{14}NO_2Se$, 332.0185; found, 332.0187. **HPLC**: Chiralpak AD-H (*n*-hexane:*i*-PrOH = 20:1, flow rate 0.3 mL/min, 10 °C, λ = 220 nm), retention times t_R(major) = 24.8 min, t_R(minor) = 30.3 min.

4-Isopropyl- 2-phenyl-4-(phenylselanyl)oxazol-5(4*H*)-one (3d):

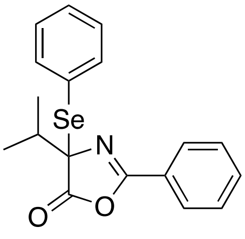

3d

Compound **3d** was prepared according to the general procedure described in **2.2** and was obtained as a colourless solid (11.3 mg, 32 µmol, 63% yield, e.r. = 79:21). **TLC** (heptanes:EtOAc = 3.5:1): R_f = 0.44 (UV). **[α]$_D^{23}$** = -11.0 (*c* 1.00, $CHCl_3$, e.r. = 79:21). **^{1}H-NMR** (500 MHz, $CDCl_3$, 298.0 K): δ / ppm = 7.75 (d, *J* = 7.9 Hz, 2H), 7.55-7.50 (m, 3H), 7.40 (t, *J* = 7.7 Hz, 2H), 7.23 (t, *J* = 7.3 Hz, 1H), 7.11 (t, *J* = 7.7 Hz, 2H), 2.54 (sep, *J* = 6.9 Hz, 1H) 1.33 (d, *J* = 6.7 Hz, 3H), 1.05 (d, *J* = 6.7 Hz, 3H). **^{13}C-NMR** (126 MHz, $CDCl_3$, 298.0 K): δ / ppm = 176.9, 160.7, 138.4, 132.8, 130.0, 129.1, 128.7, 127.9, 125.5, 125.2, 79.2, 33.9, 18.6, 18.5. **HRMS** (ESI-QTOF, MeOH) *m/z*: $[M+H]^+$ calculated for $C_{18}H_{18}NO_2Se$, 360.0498; found, 360.0501. **HPLC**: Chiralpak AD-H (*n*-hexane:*i*-PrOH = 20:1, flow rate 0.3 mL/min, 10 °C, λ = 220 nm), retention times t_R(major) = 17-1 min, t_R(minor) = 18.6 min.

4-Isobutyl- 2-phenyl-4-(phenylselanyl)oxazol-5(4*H*)-one (3e):

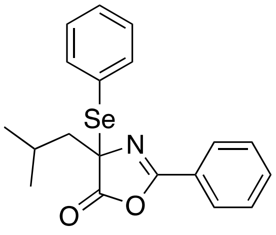

3e

Compound **3e** was prepared according to the general procedure described in **2.2** and was obtained as a colourless solid (18.2 mg, 49 µmol, 98% yield, e.r. = 92:8). **TLC** (heptanes:EtOAc = 3.5:1): R_f = 0.45 (UV). **[α]$_D^{23}$** = -93.3 (*c* 1.00, $CHCl_3$, e.r. = 92:8). **^{1}H-NMR** (500 MHz, $CDCl_3$, 298.0 K): δ / ppm = 7.74 (d, *J* = 7.2 Hz, 2H), 7.54-7.50 (m, 3H), 7.39 (t, *J* = 7.8 Hz, 2H), 7.24 (t, *J* = 7.4 Hz, 1H), 7.10 (t, *J* = 7.7 Hz, 2H), 2.32 (dd, J_1 = 5.6 Hz, J_2 = 14.2 Hz, 1H), 2.17 (dd, J_1 = 7.7 Hz, J_2 = 14.1 Hz, 1H), 1.83 (sep, *J* = 6.6 Hz, 1H), 0.95 (d, *J* = 6.6 Hz, 3H), 0.85 (d, *J* = 6.6 Hz, 3H). **^{13}C-NMR** (126 MHz, $CDCl_3$, 298.0 K): δ / ppm = 177.5, 160.3, 138.3, 132.8, 130.2, 129.0, 128.7, 127.8, 125.4, 125.4, 73.4, 43.3, 27.0, 23.6, 22.7. **HRMS** (ESI-QTOF, MeOH) *m/z*: $[M+H]^+$ calculated for $C_{19}H_{20}NO_2Se$, 374.0654; found, 374.0657. **HPLC**: YMC Chiral Art Cellulose SB (*n*-hexane:*i*-PrOH = 80:1, flow rate 0.2 mL/min, 10 °C, λ = 220 nm), retention times t_R(major) = 28.9 min, t_R(minor) = 30.0 min.

4-(Cyclohexylmethyl)-2-phenyl-4-(phenylselanyl)oxazol-5(4*H*)-one (3f):

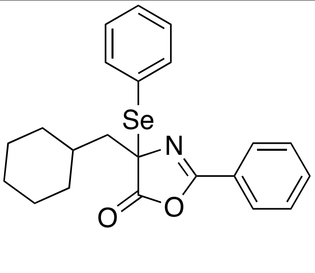

3f

Compound **3f** was prepared according to the general procedure described in **2.2** and was obtained as a white solid (19.4 mg, 47 µmol, 94% yield, e.r. = 93:7). **TLC** (heptanes:EtOAc = 3.5:1): R_f = 0.63 (UV). **$[\alpha]_D^{23}$** = -109.1 (*c* 1.00, $CHCl_3$, e.r. = 93:7). **^{1}H-NMR** (300 MHz, $CDCl_3$, 298.0 K): δ / ppm = 7.75-7.72 (m, 2H), 7.55-7.50 (m, 3H), 7.40 (t, *J* = 7.7 Hz, 2H), 7.23 (t, *J* = 7.4 Hz, 1H), 7.10 (t, *J* = 7.6 Hz, 2H), 2.33-2.15 (m, 2H), 1.73-1.47 (m, 6H), 1.19-0.88 (m, 5H). **^{13}C-NMR** (75 MHz, $CDCl_3$, 298.0 K): δ / ppm = 177.6, 160.3, 138.3, 132.8, 130.2, 129.1, 128.8, 127.9, 125.5, 125.5, 77.4, 73.4, 42.2, 36.1, 34.1, 33.2, 26.2, 26.0. **HRMS** (ESI-QTOF, MeOH) *m/z*: $[M+H]^+$ calculated for $C_{22}H_{24}NO_2Se$, 414.0967; found, 414.0965. **HPLC**: Chiralpak AD-H (*n*-hexane:*i*-PrOH = 20:1, flow rate 0.7 mL/min, 10 °C, λ = 220 nm), retention times t_R(minor) = 20.6 min, t_R(major) = 22.9 min.

4-(2-(Methylthio)ethyl)-2-phenyl-4-(phenylselanyl)oxazol-5(4*H*)-one (3g):

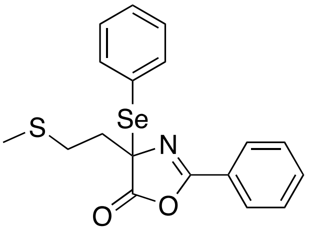

3g

Compound **3g** was prepared according to the general procedure described in **2.2** and was obtained as a colourless solid (19.1 mg, 49 µmol, 98% yield, e.r. = 93:7). **TLC** (heptanes:EtOAc = 3.5:1): R_f = 0.43 (UV). **$[\alpha]_D^{24}$** = -84.9 (*c* 1.00, $CHCl_3$, e.r. = 93:7). **^{1}H-NMR** (300 MHz, $CDCl_3$, 298.0 K): δ / ppm = 7.77-7.75 (m, 2H), 7.57-7.51 (m, 3H), 7.40 (t, *J* = 7.8 Hz, 2H), 7.29-7.23 (m, 1H), 7.14 (t, *J* = 7.6 Hz, 2H), 2.66-2.56 (m, 4H), 2.05 (s, 3H). **^{13}C-NMR** (75 MHz, $CDCl_3$, 298.0 K): δ / ppm = 177.1, 161.5, 138.3, 133.0, 130.3, 129.2, 128.8, 128.0, 125.4, 125.0, 72.3, 34.1, 30.3, 15.1. **HRMS** (ESI-QTOF, MeOH) *m/z*: $[M+H]^+$ calculated for $C_{18}H_{18}NO_2SSe$, 392.0218; found, 392.0219. **HPLC**: Chiralpak AD-H (*n*-hexane:*i*-PrOH = 20:1, flow rate 0.3 mL/min, 10 °C, λ = 220 nm), retention times t_R(minor) = 31.7 min, t_R(major) = 33.2 min.

4-(4-Chlorobenzyl)- 2-phenyl-4-(phenylselanyl)oxazol-5(4*H*)-one (3h):

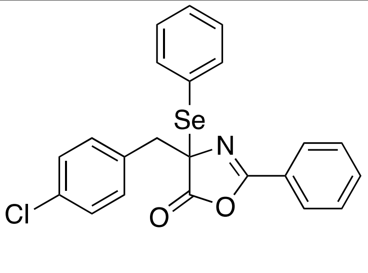

3h

Compound **3h** was prepared according to the general procedure described in **2.2** and was obtained as a colourless solid (21.8 mg, 50 μmol, 98% yield, e.r. = 82:18). **TLC** (heptanes:EtOAc = 3.5:1): R_f = 0.49 (UV). **[α]D²⁴** = -2.3 (*c* 1.00, $CHCl_3$, e.r. = 82:18). **^{1}H-NMR** (300 MHz, $CDCl_3$, 298.0 K): δ / ppm = 7.62 (d, *J* = 6.0 Hz, 2H), 7.54 (d, *J* = 9.0 Hz, 2H), 7.48-7.43 (m, 1H), 7.32 (t, *J* = 9.0 Hz, 2H), 7.24-7.19 (m, 1H), 7.17-7.08 (m, 6H), 3.55 (d, *J* = 12.0 Hz, 1H), 3.42 (d, *J* = 12.0 Hz, 1H). **^{13}C-NMR** (75 MHz, $CDCl_3$, 298.0 K): δ / ppm = 176.3, 160.9, 138.3, 134.5, 133.6, 133.0, 133.0, 131.6, 130.3, 129.2, 128.7, 127.9, 125.2, 125.1, 123.7, 73.4, 40.5. **HRMS** (ESI-QTOF, MeOH) *m/z*: $[M+H]^+$ calculated for $C_{22}H_{17}ClNO_2Se$, 442.0108; found, 442.0106. **HPLC**: Chiralpak AD-H (*n*-hexane:*i*-PrOH = 20:1, flow rate 0.3 mL/min, 10 °C, λ = 220 nm), retention times t_R(major) = 31.4 min, t_R(minor) = 33.9 min.

4-(4-Nitrobenzyl)- 2-phenyl-4-(phenylselanyl)oxazol-5(4*H*)-one (3i):

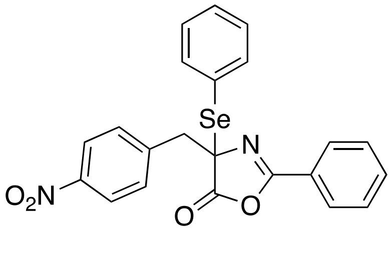

3i

Compound **3i** was prepared according to the general procedure described in **2.2** and was obtained as a light-yellow solid (21.0 mg, 47 μmol, 93% yield, e.r. = 69:31). **TLC** (heptanes:EtOAc = 3.5:1): R_f = 0.42 (UV). **$[\alpha]_D^{24}$** = -27.2 (*c* 1.00, $CHCl_3$, e.r. = 69:31). **^{1}H-NMR** (300 MHz, $CDCl_3$, 298.0 K): δ / ppm = 8.08 (d, *J* = 8.8 Hz, 2H), 7.68-7.65 (m, 2H), 7.59-7.56 (m, 2H), 7.54-7.49 (m, 1H), 7.44 (d, *J* = 8.8 Hz, 2H), 7.37 (t, *J* = 7.8 Hz, 2H), 7.31-7.25 (m, 1H), 7.16 (t, *J* = 7.5 Hz, 2H), 3.72 (d, *J* = 13.7 Hz, 1H), 3.58 (d, *J* = 13.7 Hz, 1H). **^{13}C-NMR** (75 MHz, $CDCl_3$, 298.0 K): δ / ppm = 176.1, 161.2, 147.5, 142.0, 138.3, 133.3, 131.2, 130.5, 129.3, 128.8, 127.9, 124.9, 124.8, 123.7, 72.6, 40.8. **HRMS** (ESI-QTOF, MeOH) *m/z*: $[M+H]^+$ calculated for $C_{22}H_{17}N_2O_4Se$, 453.0348; found, 453.0352. **HPLC**: Chiralpak AD-H (*n*-hexane:*i*-PrOH = 20:1, flow rate 0.7 mL/min, 10 °C, λ = 220 nm), retention times t_R(minor) = 30.0 min, t_R(major) = 32.4 min.

4-Benzyl-4-(phenylselanyl)-2-(4-(trifluoromethyl)phenyl)oxazol-5(4*H*)-one (3j):

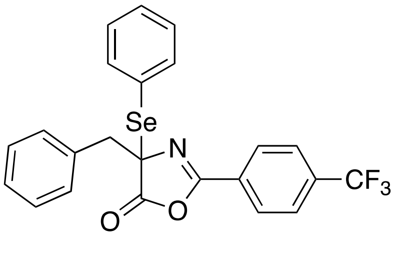

3j

Compound **3j** was prepared according to the general procedure described in **2.2** and was obtained as a colourless solid (19.2 mg, 41 µmol, 81% yield, e.r. = 72:28). **TLC** (heptanes:EtOAc = 3.5:1): R_f = 0.45 (UV). **[α]$_D^{24}$** = -46.4 (*c* 1.00, $CHCl_3$, e.r. = 72:28). **^{1}H-NMR** (700 MHz, $CDCl_3$, 298.0 K): δ / ppm = 7.76 (d, *J* = 14.0 Hz, 2H), 7.62 (d, *J* = 7.0 Hz, 2H), 7.58 (d, *J* = 7.0 Hz, 2H), 7.28-7.24 (m, 3H), 7.21 (t, *J* = 7.0 Hz, 2H), 7.19-7.14 (m, 3H), 3.63 (d, *J* = 14.0 Hz, 1H), 3.54 (d, *J* = 7.0 Hz, 1H). **^{13}C-NMR** (176 MHz, $CDCl_3$, 298.0 K): δ / ppm = 175.9, 159.4, 138.3, 134.3, 130.4, 130.2, 129.3, 128.6, 128.2, 127.7, 125.7, 125.7, 125.1, 122.8, 73.8, 41.1. ^{19}F-NMR (471 MHz, $CDCl_3$, 298.0 K): δ / ppm = -63.21. **HRMS** (ESI-QTOF, MeOH) *m/z*: $[M+H]^+$ calculated for $C_{23}H_{17}F_3NO_2Se$, 476.0371; found, 476.0377. **HPLC**: Chiralpak AD-H (*n*-hexane:*i*-PrOH = 20:1, flow rate 0.3 mL/min, 10 °C, λ = 220 nm), retention times t_R(minor) = 21.3 min, t_R(major) = 26.3 min.

4-Benzyl-2-(4-nitrophenyl)-4-(phenylselanyl)oxazol-5(4*H*)-one (3k):

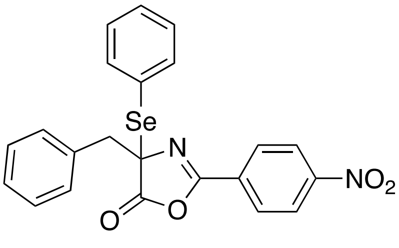

3k

Compound **3k** was prepared according to the general procedure described in **2.2** and was obtained as a yellow solid (12.2 mg, 27 µmol, 54% yield, e.r. = 63:37). **TLC** (heptanes:EtOAc = 3.5:1): R_f = 0.52 (UV). **[α]$_D^{23}$** = -23.1 (*c* 1.00, $CHCl_3$, e.r. = 63:37). **^{1}H-NMR** (300 MHz, $CDCl_3$, 298.0 K): δ / ppm = 8.20 (d, *J* = 9.1 Hz, 2H), 7.81 (d, *J* = 8.7 Hz, 2H), 7.58-7.55 (m, 2H), 7.29-7.12 (m, 8H), 3.65 (d, *J* = 13.7 Hz, 1H), 3.54 (d, *J* = 13.7 Hz, 1H). **^{13}C-NMR** (75 MHz, $CDCl_3$, 298.0 K): δ / ppm = 175.6, 158.8, 150.3, 138.3, 134.3, 130.8, 130.5, 130.2, 129.3, 128.8, 128.6, 127.8, 125.1, 123.9, 73.8, 41.0. **HRMS** (ESI-QTOF, MeOH) *m/z*: $[M+H]^+$ calculated for $C_{22}H_{17}N_2O_4Se$, 453.0348; found, 453.0343. **HPLC**: Chiralpak AD-H (*n*-hexane:*i*-PrOH = 20:1, flow rate 0.7 mL/min, 10 °C, λ = 220 nm), retention times t_R(minor) = 22.5 min, t_R(major) = 29.0 min.

4-Benzyl-2-(4-methoxyphenyl)-4-(phenylselanyl)oxazol-5(4*H*)-one (3l):

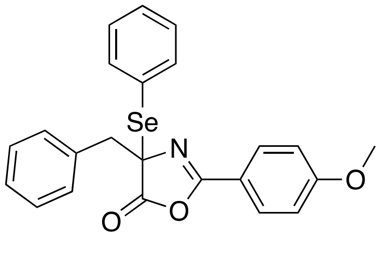

3l

Compound **3l** was prepared according to the general procedure described in **2.2** and was obtained as a colourless solid (18.8 mg, 43 µmol, 86% yield, e.r. = 91:9). **TLC** (heptanes:EtOAc = 3.5:1): R_f = 0.20 (UV). **[α]$_D$^{24}** = -44.9 (*c* 1.00, $CHCl_3$, e.r. = 91:9). **^{1}H-NMR** (300 MHz, $CDCl_3$, 298.0 K): δ / ppm = 7.63-7.58 (m, 4H), 7.29-7.12 (m, 8H), 6.84 (d, *J* = 9.0 Hz, 2H), 3.83 (s, 3H), 3.62 (d, *J* = 13.6 Hz, 1H), 3.48 (d, *J* = 13.6 Hz, 1H). **^{13}C-NMR** (75 MHz, $CDCl_3$, 298.0 K): δ / ppm = 176.6, 163.3, 160.5, 138.2, 134.7, 130.3, 130.1, 129.8, 129.1, 128.5, 127.5, 125.5, 117.6, 114.1, 74.0, 55.6, 41.4. **HRMS** (ESI-QTOF, MeOH) *m/z*: $[M+H]^+$ calculated for $C_{23}H_{20}NO_3Se$, 438.0603; found, 438.0605. **HPLC**: Chiralpak AD-H (*n*-hexane:*i*-PrOH = 20:1, flow rate 0.7 mL/min, 10 °C, λ = 270 nm), retention times t_R(minor) = 18.2 min, t_R(major) = 23.3 min.

4-Benzyl-2-(4-chlorophenyl)-4-(phenylselanyl)oxazol-5(4*H*)-one (3m):

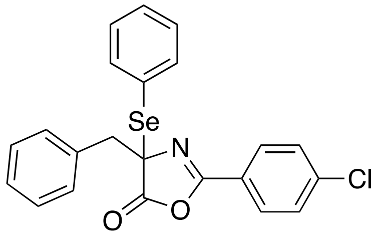

3m

Compound **3m** was prepared according to the general procedure described in **2.2** and was obtained as a colourless solid (17.0 mg, 39 µmol, 77% yield, e.r. = 74:26). **TLC** (heptanes:EtOAc = 3.5:1): R_f = 0.58 (UV). **[α]$_D$^{24}** = -37.7 (*c* 1.00, $CHCl_3$, e.r. = 74:26). **^{1}H-NMR** (300 MHz, $CDCl_3$, 298.0 K): δ / ppm = 7.60-7.55 (m, 4H), 7.35-7.31 (m, 2H), 7.27-7.12 (m, 8H), 3.62 (d, *J* = 13.7 Hz, 1H), 3.50 (d, *J* = 13.7 Hz, 1H). **^{13}C-NMR** (75 MHz, $CDCl_3$, 298.0 K): δ / ppm = 176.1, 159.8, 139.3, 138.3, 134.5, 130.3, 130.2, 129.2, 129.2, 128.6, 127.7, 125.2, 123.7, 73.9, 41.2. **HRMS** (ESI-QTOF, MeOH) *m/z*: $[M+H]^+$ calculated for $C_{22}H_{17}ClNO_2Se$, 442.0108; found, 442.0108. **HPLC**: Chiralpak AD-H (*n*-hexane:*i*-PrOH = 20:1, flow rate 0.3 mL/min, 10 °C, λ = 220 nm), retention times t_R(minor) = 25.1 min, t_R(major) = 33.2 min.

4-Benzyl-2-(naphthalen-2-yl)-4-(phenylselanyl)oxazol-5(4*H*)-one (3n):

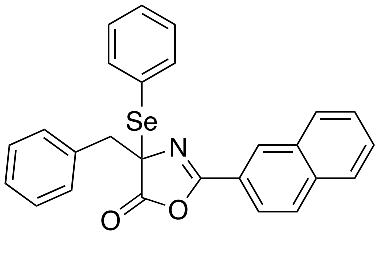

3n

Compound **3n** was prepared according to the general procedure described in **2.2** and was obtained as a light-yellow solid (18.7 mg, 41 µmol, 82% yield, e.r. = 87:13). **TLC** (heptanes:EtOAc = 3.5:1): R_f = 0.47 (UV). **[α]$_D^{24}$** = -88.0 (*c* 1.00, $CHCl_3$, e.r. = 87:13). **^{1}H-NMR** (300 MHz, $CDCl_3$, 298.0 K): δ / ppm = 8.07 (s, 1H), 7.83 (t, *J* = 6.8 Hz, 4H), 7.64-7.61 (m, 2H), 7.58-7.49 (m, 2H), 7.31-7.28 (m, 2H), 7.24-7.09 (m, 6H), 3.68 (d, *J* = 13.5 Hz, 1H), 3.55 (d, *J* = 13.7 Hz, 1H). **^{13}C-NMR** (75 MHz, $CDCl_3$, 298.0 K): δ / ppm = 176.4, 160.8, 138.2, 135.4, 134.6, 132.5, 130.2, 130.2, 129.4, 129.2, 129.2, 128.6, 128.5, 128.0, 127.6, 127.1, 125.3, 123.4, 122.5, 74.1, 41.3. **HRMS** (ESI-QTOF, MeOH) *m/z*: $[M+H]^+$ calculated for $C_{26}H_{20}NO_2Se$, 458.0654; found, 458.0649. **HPLC**: Chiralpak AD-H (*n*-hexane:*i*-PrOH = 20:1, flow rate 0.7 mL/min, 10 °C, λ = 220 nm), retention times t_R(minor) = 13.7 min, t_R(major) = 16.2 min.

4-Benzyl-2-ethyl-4-(phenylselanyl)oxazol-5(4*H*)-one (3o):

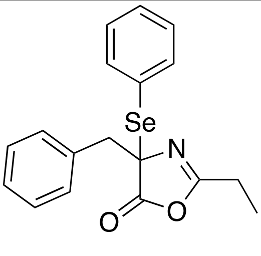

3o

Compound **3o** was prepared according to the general procedure described in **2.2** and was obtained as a colourless solid (14.2 mg, 40 µmol, 79% yield, e.r. = 53:47). **TLC** (heptanes:EtOAc = 3.5:1): R_f = 0.36 (UV). **^{1}H-NMR** (500 MHz, $CDCl_3$, 298.0 K): δ / ppm = 7.63-7.60 (m, 2H), 7.42 (t, *J* = 7.7 Hz, 1H), 7.32 (t, *J* = 7.7 Hz, 2H), 7.25-7.19 (m, 5H), 3.48 (d, *J* = 13.5 Hz, 1H), 3.42 (d, *J* = 13.5 Hz, 1H), 2.02-1.88 (m, 2H), 0.84 (t, *J* = 7.7 Hz, 3H). **^{13}C-NMR** (126 MHz, $CDCl_3$, 298.0 K): δ / ppm = 177.0, 166.0, 138.3, 134.4, 130.3, 130.2, 129.3, 128.5, 127.6, 125.3, 72.7, 41.0, 22.1, 8.8. **HPLC**: Chiralpak AD-H (*n*-hexane:*i*-PrOH = 20:1, flow rate 0.3 mL/min, 10 °C, λ = 220 nm), retention times t_R(major) = 21.5 min, t_R(minor) = 22.6 min.

4-Benzyl-2-cyclohexyl-4-(phenylselanyl)oxazol-5(4*H*)-one (3p):

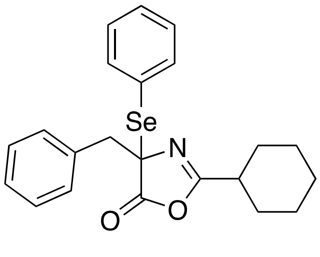

3p

Compound **3p** was prepared according to the general procedure described in **2.2** and was obtained as a colourless solid (6.8 mg, 17 µmol, 33% yield, e.r. = 53:47). **TLC** (heptanes:EtOAc = 3.5:1): R_f = 0.57 (UV). **^{1}H-NMR** (500 MHz, CDCl$_3$, 298.0 K): δ / ppm = 7.64-7.62 (m, 2H), 7.42-7.39 (m, 1H), 7.32 (t, *J* = 7.7 Hz, 2H), 7.25-7.17 (m, 5H), 3.47 (d, *J* = 13.6 Hz, 1H), 3.42 (d, *J* = 13.2 Hz, 1H), 1.93-1.89 (m, 1H), 1.59-1.51 (m, 4H), 1.42-1.36 (m, 3H), 1.10-0.98 (m, 3H). **HRMS** (ESI-QTOF, MeOH) *m/z*: [M+H]$^+$ calculated for $C_{22}H_{24}NO_2Se$, 414.0967; found, 414.0963. **HPLC**: Chiralpak AD-H (*n*-hexane:*i*-PrOH = 20:1, flow rate 0.3 mL/min, 10 °C, λ = 220 nm), retention times t_R(major) = 19.2 min, t_R(minor) = 19.9 min.

4-(4-Chlorobenzyl)-2-(4-methoxyphenyl)-4-(phenylselanyl)oxazol-5(4*H*)-one (3q):

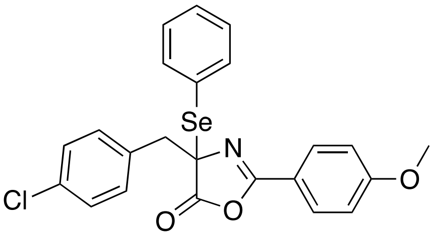

3q

Compound **3q** was prepared according to the general procedure described in **2.2** and was obtained as a colourless solid (23.1 mg, 49 µmol, 98% yield, e.r. = 87:13). **TLC** (heptanes:EtOAc = 3.5:1): R_f = 0.34 (UV). **$[\alpha]_D^{23}$** = -7.5 (*c* 1.00, CHCl$_3$, e.r. = 87:13). **^{1}H-NMR** (300 MHz, CDCl$_3$, 298.0 K): δ / ppm = 7.72-7.65 (m, 4H), 7.38-7.33 (m, 2H), 7.29-7.21 (m, 5H), 6.94 (d, *J* = 8.9 Hz, 2H), 3.92 (s, 3H), 3.66 (d, *J* = 13.8 Hz, 1H), 3.52 (d, *J* = 13.8 Hz, 1H). **^{13}C-NMR** (75 MHz, CDCl$_3$, 298.0 K): δ / ppm = 176.5, 163.5, 160.7, 138.3, 133.5, 133.2, 131.6, 130.2, 129.9, 129.2, 128.7, 125.3, 117.4, 114.2, 73.5, 55.6, 40.7. **HRMS** (ESI-QTOF, MeOH) *m/z*: [M+H]$^+$ calculated for $C_{23}H_{19}ClNO_3Se$, 472.0213; found, 472.0205. **HPLC**: Chiralpak AD-H (*n*-hexane:*i*-PrOH = 20:1, flow rate 0.7 mL/min, 10 °C, λ = 220 nm), retention times t_R(minor) = 21.5 min, t_R(major) = 25.2 min.

2-(4-Methoxyphenyl)-4-(4-nitrobenzyl)-4-(phenylselanyl)oxazol-5(4*H*)-one (3r):

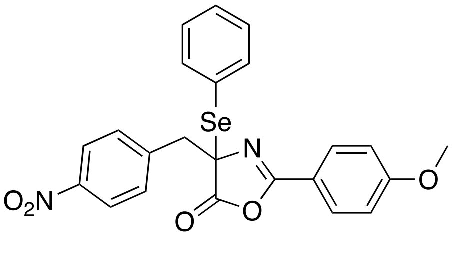

3r

Compound **3r** was prepared according to the general procedure described in **2.2** and was obtained as a yellow solid (23.6 mg, 49 µmol, 98% yield, e.r. = 79:21). **TLC** (heptanes:EtOAc = 3.5:1): R_f = 0.24 (UV). **$[\alpha]_D^{23}$** = -21.5 (*c* 1.00, $CHCl_3$, e.r. = 79:21). **^{1}H-NMR** (300 MHz, $CDCl_3$, 298.0 K): δ / ppm = 8.07 (d, *J* = 8.7 Hz, 2H), 7.63-7.57 (m, 4H), 7.43 (d, *J* = 8.4 Hz, 2H), 7.31-7.24 (m, 1H), 7.17 (t, *J* = 7.7 Hz, 2H), 6.85 (d, *J* = 9.1 Hz, 2H), 3.84 (s, 3H), 3.70 (d, *J* = 13.3 Hz, 1H), 3.56 (d, *J* = 13.9 Hz, 1H). **^{13}C-NMR** (75 MHz, $CDCl_3$, 298.0 K): δ / ppm = 176.2, 163.7, 161.0, 147.5, 142.2, 138.3, 131.2, 130.4, 129.9, 129.2, 125.1, 123.7, 117.0, 114.3, 72.8, 55.6, 41.0. **HRMS** (ESI-QTOF, MeOH) *m/z*: $[M+H]^+$ calculated for $C_{23}H_{19}N_2O_5Se$, 483.0454; found, 483.0462. **HPLC**: Chiralpak AD-H (*n*-hexane:*i*-PrOH = 20:1, flow rate 1.2 mL/min, 10 °C, λ = 220 nm), retention times t_R(minor) = 28.0 min, t_R(major) = 32.2 min.

2-(4-Methoxyphenyl)-4-methyl-4-(phenylselanyl)oxazol-5(4*H*)-one (3s):

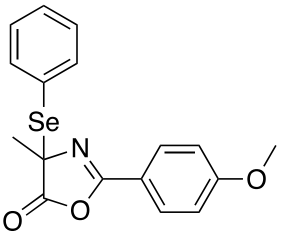

3s

Compound **3s** was prepared according to the general procedure described in **2.2** and was obtained as a colourless solid (5.9 mg, 17 µmol, 33% yield, e.r. = 71:29). **TLC** (heptanes:EtOAc = 3.5:1): R_f = 0.28 (UV). **$[\alpha]_D^{23}$** = +18.4 (*c* 0.47, $CHCl_3$, e.r. = 71:29). **^{1}H-NMR** (300 MHz, $CDCl_3$, 298.0 K): δ / ppm = 7.71 (d, *J* = 8.5 Hz, 2H), 7.57 (d, *J* = 7.7 Hz, 2H), 7.28-7.24 (m, 1H), 7.15 (t, *J* = 7.4 Hz, 2H), 6.89 (d, *J* = 8.5 Hz, 2H), 3.86 (s, 3H), 1.92 (s, 3H). **HRMS** (ESI-QTOF, MeOH) *m/z*: $[M+H]^+$ calculated for $C_{17}H_{16}NO_3Se$, 362.0290; found, 362.0294. **HPLC**: Chiralpak AD-H (*n*-hexane:*i*-PrOH = 20:1, flow rate 0.7 mL/min, 10 °C, λ = 220 nm), retention times t_R(minor) = 16.7 min, t_R(major) = 17.8 min.

4-Isobutyl-2-(4-methoxyphenyl)-4-(phenylselanyl)oxazol-5(4*H*)-one (3t):

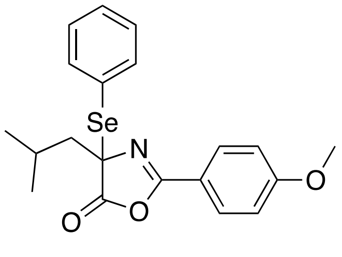

3t

Compound **3t** was prepared according to the general procedure described in **2.2** and was obtained as a colourless solid (9.7 mg, 24 µmol, 48% yield, e.r. = 59:41). **TLC** (heptanes:EtOAc = 3.5:1): R_f = 0.43 (UV). **[α]$_D^{23}$** = -32.6 (*c* 0.61, $CHCl_3$, e.r. = 59:41). **^{1}H-NMR** (300 MHz, $CDCl_3$, 298.0 K): δ / ppm = 7.70 (d, *J* = 8.7 Hz, 2H), 7.54 (d, *J* = 7.9 Hz, 2H), 7.26-7.23 (m, 1H), 7.12 (t, *J* = 7.7 Hz, 2H), 6.89 (d, *J* = 8.7 Hz, 2H), 3.86 (s, 3H), 2.33-2.29 (m, 1H), 2.18-2.14 (m, 1H), 1.82 (sep, *J* = 6.8 Hz, 1H), 0.94 (d, *J* = 6.8 Hz, 1H), 0.84 (d, *J* = 6.6 Hz, 1H). **^{13}C-NMR** (126 MHz, $CDCl_3$, 298.0 K): δ / ppm = 177.7, 163.4, 160.2, 138.3, 130.1, 129.8, 129.0, 125.5, 117.8, 114.2, 73.6, 55.6, 43.5, 27.0, 23.7, 22.7. **HRMS** (ESI-QTOF, MeOH) *m/z*: $[M+H]^+$ calculated for $C_{20}H_{22}NO_3Se$, 404.0760; found, 404.0770. **HPLC**: Chiralpak AD-H (*n*-hexane:*i*-PrOH = 20:1, flow rate 0.7 mL/min, 10 °C, λ = 220 nm), retention times t_R(minor) = 21.1 min, t_R(major) = 26.1 min.

2-(4-Methoxyphenyl)-4-(2-(methylthio)ethyl)-4-(phenylselanyl)oxazol-5(4*H*)-one (3u):

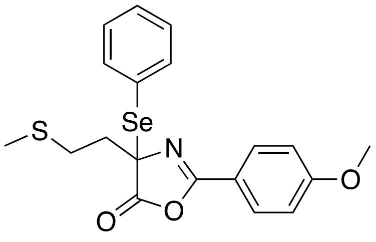

3u

Compound **3u** was prepared according to the general procedure described in **2.2** and was obtained as a colourless solid (16.8 mg, 40 µmol, 80% yield, e.r. = 91:9). **TLC** (heptanes:EtOAc = 3.5:1): R_f = 0.25 (UV). **[α]$_D^{23}$** = -32.1 (*c* 1.00, $CHCl_3$, e.r. = 91:9). **^{1}H-NMR** (300 MHz, $CDCl_3$, 298.0 K): δ / ppm = 7.72 (d, *J* = 8.6 Hz, 2H), 7.56 (d, *J* = 7.3 Hz, 2H), 7.29-7.24 (m, 1H), 7.15 (t, *J* = 8.0 Hz, 2H), 6.89 (d, *J* = 9.0 Hz, 2H), 3.86 (s, 3H), 2.71-2.52 (m, 4H), 2.04 (s, 3H). **^{13}C-NMR** (75 MHz, $CDCl_3$, 298.0 K): δ / ppm = 177.2, 163.5, 161.2, 138.3, 130.2, 130.0, 129.1, 125.1, 117.7, 114.2, 72.5, 55.6, 34.3, 30.3, 15.1. **HRMS** (ESI-QTOF, MeOH) *m/z*: $[M+H]^+$ calculated for $C_{19}H_{20}NO_3SSe$, 422.0324; found, 422.0324. **HPLC**: Chiralpak AD-H (*n*-hexane:*i*-PrOH = 20:1, flow rate 0.7 mL/min, 10 °C, λ = 220 nm), retention times t_R(minor) = 24.2 min, t_R(major) = 29.7 min.

4-Benzyl-2-phenyl-4-(*o*-tolylselanyl)oxazol-5(4*H*)-one (3v):

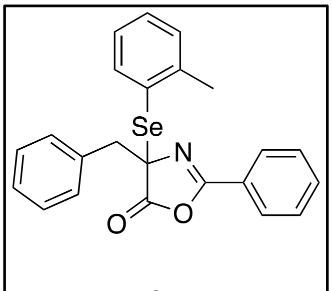

3v

Compound **3v** was prepared according to the general procedure described in **2.2** using *N*-(*o*-tolylseleno)phthalimide as a selenation agent and was obtained as a colourless solid (16.0 mg, 38 μmol, 76% yield, e.r. = 88:12). **TLC** (heptanes:EtOAc = 3.5:1): R_f = 0.53 (UV). **[α]$_D^{24}$** = -84.4 (*c* 1.00, $CHCl_3$, e.r. = 88:12). **^{1}H-NMR** (300 MHz, $CDCl_3$, 298.0 K): δ / ppm = 7.65-7.62 (m, 2H), 7.58 (d, *J* = 7.5 Hz, 1H), 7.50-7.45 (m, 1H), 7.34 (t, *J* = 7.7 Hz, 2H), 7.29-7.16 (m, 5H), 7.12-7.10 (m, 2H), 6.92-6.87 (m, 1H), 3.66 (d, *J* = 13.8 Hz, 1H), 3.54 (d, *J* = 13.8 Hz, 1H), 2.52 (s, 3H). **^{13}C-NMR** (75 MHz, $CDCl_3$, 298.0 K): δ / ppm = 176.7, 160.3, 144.4, 139.9, 134.7, 132.7, 130.7, 130.3, 130.2, 128.6, 128.5, 127.8, 127.5, 126.5, 126.5, 125.3, 74.2, 41.2, 23.7. **HRMS** (ESI-QTOF, MeOH) *m/z*: $[M+H]^+$ calculated for $C_{23}H_{20}NO_2Se$, 422.0654; found, 422.0650. **HPLC**: YMC Chiral Art Cellulose SB (*n*-hexane:*i*-PrOH = 20:1, flow rate 0.3 mL/min, 10 °C, λ = 270 nm), retention times t_R(minor) = 24.1 min, t_R(major) = 25.3 min.

4-Benzyl-2-phenyl-4-(*p*-tolylselanyl)oxazol-5(4*H*)-one (3w):

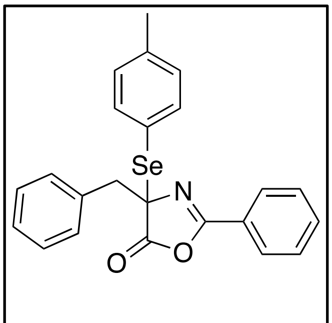

3w

Compound **3w** was prepared according to the general procedure described in **2.2** using *N*-(*p*-tolylseleno)phthalimide as a selenation agent and was obtained as a colourless solid (10.1 mg, 24 μmol, 48% yield, e.r. = 76:24). **TLC** (heptanes:EtOAc = 3.5:1): R_f = 0.44 (UV). **[α]$_D^{23}$** = -51.8 (*c* 0.80, $CHCl_3$, e.r. = 76:24). **^{1}H-NMR** (300 MHz, $CDCl_3$, 298.0 K): δ / ppm = 7.66 (d, *J* = 7.2 Hz, 2H), 7.51-7.44 (m, 3H), 7.36 (t, *J* = 7.8 Hz, 2H), 7.27-7.16 (m, 5H), 6.94 (d, *J* = 7.9 Hz, 2H), 3.62 (d, *J* = 13.8 Hz, 1H), 3.49 (d, *J* = 13.8 Hz, 1H), 2.20 (s, 3H). **^{13}C-NMR** (75 MHz, $CDCl_3$, 298.0 K): δ / ppm = 176.5, 160.6, 140.6, 138.2, 134.7, 132.8, 130.2, 130.0, 128.6, 128.5, 127.9, 127.5, 125.4, 122.0, 73.7, 41.2, 21.3. **HRMS** (ESI-QTOF, MeOH) *m/z*: $[M+H]^+$ calculated for $C_{23}H_{20}NO_2Se$, 422.0654; found, 422.0658. **HPLC**: Chiralpak AD-H (*n*-hexane:*i*-PrOH = 20:1, flow rate 0.3 mL/min, 10 °C, λ = 220 nm), retention times t_R(major) = 30.9 min, t_R(minor) = 32.8 min.

4-Benzyl-4-(benzylselanyl)-2-phenyloxazol-5(4*H*)-one (3x):

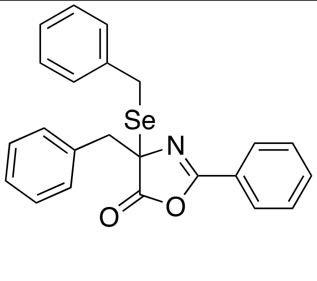

3x

Compound **3x** was prepared according to the general procedure described in **2.2** using *N*-(phenylseleno)phthalimide as a selenation agent and a reaction time of 3 h. It was obtained as a colourless solid (8.4 mg, 20 µmol, 40% yield, e.r. = 74:26). **TLC** (heptanes:EtOAc = 3.5:1): R_f = 0.51 (UV). **$[\alpha]_D^{23}$** = -61.3 (*c* 0.53, $CHCl_3$, e.r. = 74:26). **^{1}H-NMR** (300 MHz, $CDCl_3$, 298.0 K): δ / ppm = 7.92-7.89 (m, 2H), 7.58-7.52 (m, 1H), 7.47-7.42 (m, 2H), 7.32-7.15 (m, 10H), 4.07 (d, *J* = 11.2 Hz, 1H), 4.01 (d, *J* = 11.2 Hz, 1H), 3.56 (d, *J* = 13.8 Hz, 1H), 3.43 (d, *J* = 13.8 Hz, 1H). **^{13}C-NMR** (75 MHz, $CDCl_3$, 298.0 K): δ / ppm = 177.0, 161.1, 136.4, 134.3, 133.2, 130.3, 129.5, 128.9, 128.8, 128.5, 128.2, 127.6, 127.4, 125.3, 69.4, 42.7, 28.7. **HRMS** (ESI-QTOF, MeOH) *m/z*: $[M+H]^+$ calculated for $C_{23}H_{20}NO_2Se$, 422.0654; found, 422.0652. **HPLC**: Chiralpak AD-H (*n*-hexane:*i*-PrOH = 20:1, flow rate 0.3 mL/min, 10 °C, λ = 220 nm), retention times t_R(minor) = 39.3 min, t_R(major) = 45.9 min.

tert-Butyl- ((1*S*)-1-(4-benzyl-5-oxo-4-(phenylselanyl)-4,5-dihydrooxazol-2-yl)ethyl)carbamate (8):

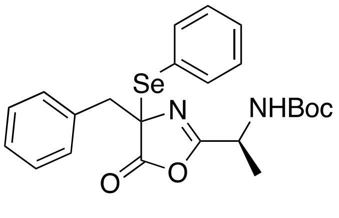

8

Compound **8** was prepared according to the general procedure described in **2.2** using *N*-(phenylseleno)phthalimide as a selenation agent and quinidine **QD** as a catalyst. It was obtained as a colourless solid (20.4 mg, 43 µmol, 86% yield, d.r. = 17:83). The two diastereomers were separated using preparative HPLC. **TLC** (heptanes:EtOAc = 3.5:1): R_f = 0.67 (UV). **^{1}H-NMR** (300 MHz, $CDCl_3$, 298.0 K, major diastereomer): δ / ppm = 7.60 (d, *J* = 7.1 Hz, 2H), 7.47-7.41 (m, 1H), 7.36-7.31 (m, 2H), 7.24-7.15 (m, 5H), 4.66 (d, *J* = 8.1 Hz, 1H), 4.10-4.05 (m, 1H), 3.51-3.41 (m, 2H), 1.46 (s, 9H), 0.89 (d, *J* = 6.9 Hz, 3H). **^{1}H-NMR** (300 MHz, $CDCl_3$, 298.0 K, minor diastereomer): δ / ppm = 7.64-7.61 (m, 2H), 7.44-7.38 (m, 1H), 7.35-7.30 (m, 2H), 7.25-7.23 (m, 3H), 7.19-7.15 (m, 2H), 4.66 (d, *J* = 7.9 Hz, 1H), 4.23-4.11 (m, 1H), 3.48 (d, *J* = 13.6 Hz, 1H), 3.39 (d, *J* = 13.6 Hz, 1H), 1.45 (s, 9H), 0.82 (d, *J* = 7.1 Hz, 3H). **HRMS** (ESI-QTOF, MeOH) *m/z*: $[M+H]^+$ calculated for $C_{23}H_{27}N_2O_4Se$, 475.1131; found, 475.1126. **Preparative HPLC**: Grace Alltima Silica 10 µm, 250 x 10 mm (*n*-hexane:EtOAc = 9:1, flow rate 5 mL/min, λ = 230 nm), retention times t_R(major) = 12.3 min, t_R(minor) = 14.4 min.

4. Further Transformations of 4-Benzyl-2-phenyl-4-(phenylselanyl)oxazol-5(4*H*)-one 3a

Methyl 2-benzamido-2-methoxy-3-phenylpropanoate (6):

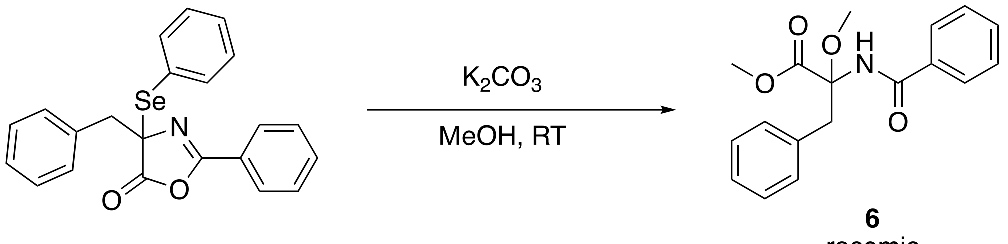

3a
76 % ee

6
racemic
28% yield

Compound **6** was prepared in analogy to a reported procedure[4] by addition of a solution of 4-benzyl-2-phenyl-4-(phenylselanyl)oxazol-5(4*H*)-one (**3a**, 0.046 mmol, 18.6 mg, 1 equiv.) in 0.115 mL methanol to a suspension of K_2CO_3 (0.046 mmol, 6.4 mg, 1 equiv.) in 0.115 mL methanol (in total 0.2 M concerning the substrate **3a**). The resulting mixture was stirred at room temperature under an argon atmosphere for 21 h. After extractive work up using DCM and sat. NaCl solution (aq.) the organic phases were dried over Na_2SO_4 and the solvent was evaporated to obtain 10.5 mg of crude product **6** (28 % yield, e.r. = 52.5:47.5). **TLC** (heptanes:EtOAc = 4:1): R_f = 0.24 (UV). **^{1}H-NMR** (300 MHz, $CDCl_3$, 298.0 K): δ / ppm = 7.74-7.71 (m, 2H), 7.56-7.50 (m, 1H), 7.46-7.41 (m, 2H), 7.27-7.24 (m, 2H), 7.17-7.10 (m, 3H), 4.06 (d, *J* = 13.4 Hz, 1H), 3.88 (s, 3H), 3.33 (d, *J* = 13.2 Hz, 1H), 3.31 (s, 3H). **^{13}C-NMR** (75 MHz, $CDCl_3$, 298.0 K): δ / ppm = 170.8, 166.9, 134.5, 134.3, 132.2, 130.3, 128.9, 128.6, 127.5, 127.2, 89.2, 53.3, 52.3, 41.2. **HRMS** (ESI-QTOF, MeOH) *m/z*: $[M+Na]^+$ calculated for $C_{18}H_{19}NO_4Na$, 336.1206; found, 336.1207. **HPLC**: Chiralpak AD-H (*n*-hexane:*i*-PrOH = 20:1, flow rate 0.9 mL/min, 10 °C, λ = 220 nm), retention times t_R(major) = 41.7 min, t_R(minor) = 43.6 min.

[4] J. Yang, W. Sun, Z. He, C. Yu, G. Bao, Y. Li, Y. Liu, L. Hong and R. Wang, *Org. Lett.* 2018, **20,** 22, 7080-7084

5. Synthesis of α-Selenated α-Aryl-Isoxazolidin-5-ones 4

5.1 General Racemic Procedure

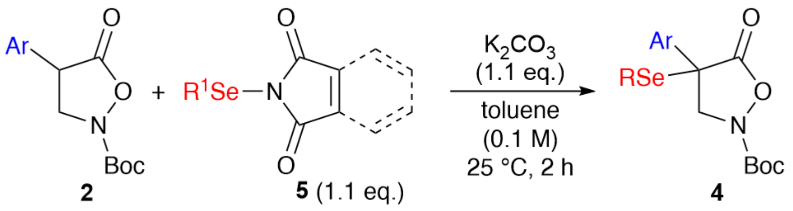

A flame-dried Schlenk-tube equipped with a stirring bar under argon atmosphere was charged with α-Aryl-Isoxazolidin-5-one **2** (0.1 mmol, 1 equiv), K_2CO_3 (15.2 mg, 1.1 equiv) and dry toluene (1 mL, 0.1 M with respect to **2**). Selenation reagent **5** (1.1-1.2 equiv) was added and the reaction flask was covered with aluminum foil. The reaction mixture was stirred for 2 h, whereupon it was concentrated under reduced pressure and directly subjected to column chromatography with gradient elution (silica gel, heptanes/EtOAc = 1/0 to 10/1) to obtain alpha-selenation products **4.**

5.2 General Enantioselective Procedure

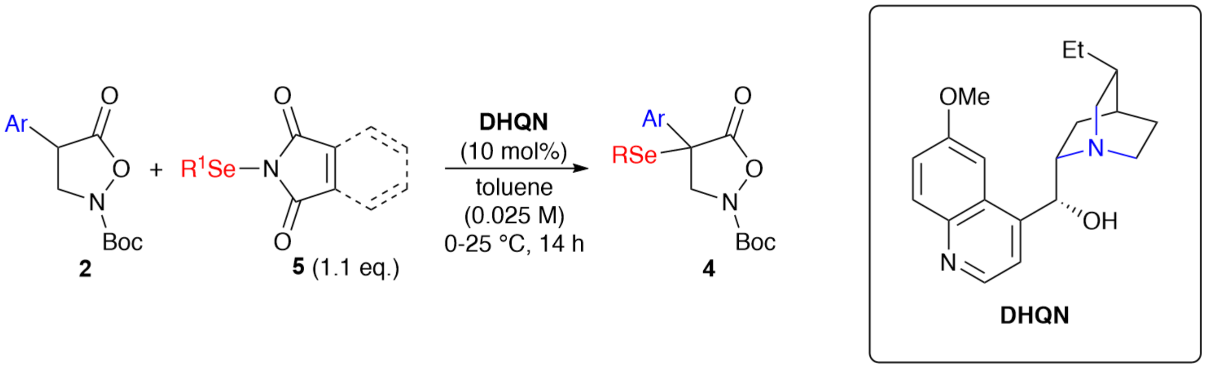

A flame-dried Schlenk-tube equipped with a stirring bar under argon atmosphere was charged with α-Aryl-Isoxazolidin-5-one **2** (1 equiv), **DHQN** (10 mol%) and dry toluene (0.025 M with respect to **2**). The mixture was stirred until all solids dissolved to give a clear, colorless solution which was cooled in an ice bath for 10 min. Selenation reagent **5** (1.1-1.2 equiv) was added at once and the reaction flask was covered with aluminum foil. The reaction mixture was gradually warmed to room temperature and stirred for 14 h, whereupon it was concentrated under reduced pressure and directly subjected to column chromatography with gradient elution (silica gel, heptanes/EtOAc = 1/0 to 10/1) to obtain alpha-selenation products **4** in the given yields and enantiopurities.

6. Characterization Data of α-Selenated α-Aryl-Isoxazolidin-5-ones 4

tert-Butyl 5-oxo-4-phenyl-4-(phenylselanyl)isoxazolidine-2-carboxylate (4a):

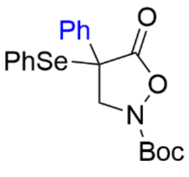

(+)-**4a**

Compound (+)-**4a** was prepared from **2a** (199 mg, 756 µmol) and *N*-(phenylseleno)succinimide **9** (207 mg, 1.1 equiv) according to the general procedure described in **5.2** and was obtained as a colorless oil, which solidifies upon storage in a refrigerator. (227 mg, 543 µmol, 72% yield, e.r. = 83:17). **TLC** (heptanes:EtOAc = 10:1): R_f = 0.24 (UV). **[α]$_D^{23}$** = +90.2 (*c* 1.01, $CHCl_3$). **^{1}H-NMR** (300 MHz, $CDCl_3$, 298.0 K): δ / ppm = 7.41-7.34 (m, 3H), 7.30-7.18 (m, 7H), 4.75 (d, *J* = 12.7 Hz, 1H), 4.34 (d, *J* = 12.7 Hz, 1H), 1.56 (s, 9H). **^{13}C-NMR** (75 MHz, $CDCl_3$, 298.0 K): δ / ppm = 172.1, 156.6, 138.3, 135.7, 130.6, 129.3, 128.8, 128.6, 127.5, 126.3, 84.7, 59.5, 49.3, 28.4. **HRMS** (ESI-QTOF, MeOH) *m/z*: $[M+NH_4]^+$ calculated for $C_{20}H_{25}N_2O_4Se^+$, 437.0974; found, 437.0973. **HPLC**: Chiralpak AD-H (*n*-hexane:*i*-PrOH = 20:1, flow rate 1.0 mL/min, 20 °C, λ = 254 nm), retention times t_R(minor) = 9.7 min, t_R(major) = 11.0 min.

tert-Butyl 4-(naphthalen-2-yl)-5-oxo-4-(phenylselanyl)isoxazolidine-2-carboxylate (4b):

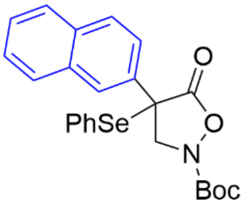

(+)-**4b**

Compound (+)-**4b** was prepared from **2b** (31.8 mg, 101 µmol) and *N*-(phenylseleno)succinimide **9** (28 mg, 1.1 equiv) according to the general procedure described in **5.2** and was obtained as a colorless oil (31.2 mg, 67 µmol, 66% yield, e.r. = 82:18). **TLC** (heptanes:EtOAc = 10:1): R_f = 0.23 (UV). **[α]$_D^{23}$** = +84.6 (*c* 1.02, $CHCl_3$). **^{1}H-NMR** (300 MHz, $CDCl_3$, 298.0 K): δ / ppm = 7.85-7.78 (m, 2H), 7.68-7.60 (m, 3H), 7.53-7.43 (m, 2H), 7.37-7.30 (m, 1H), 7.22-7.10 (m, 4H), 4.85 (d, *J* = 12.7 Hz, 1H), 4.43 (d, *J* = 12.7 Hz, 1H), 1.57 (s, 9H). **^{13}C-NMR** (75 MHz, $CDCl_3$, 298.0 K): δ / ppm = 172.0, 156.7, 138.3, 132.9, 132.6, 130.6, 129.3, 128.9, 128.7, 127.8, 127.2, 126.9, 126.7, 126.4, 124.9, 84.8, 59.5, 49.7, 28.4. **HRMS** (ESI-QTOF, MeOH) *m/z*: $[M+NH_4]^+$ calculated for $C_{24}H_{27}N_2O_4Se^+$, 487.1131; found, 487.1129. **HPLC**: Chiralpak AD-H (*n*-hexane:*i*-PrOH = 20:1, flow rate 1.0 mL/min, 20 °C, λ = 254 nm), retention times t_R(minor) = 12.5 min, t_R(major) = 16.7 min.

***tert*-Butyl 5-oxo-4-(phenylselanyl)-4-(thiophen-3-yl)isoxazolidine-2-carboxylate (4c):**

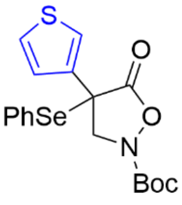

(+)-**4c**

Compound (+)-**4c** was prepared from **2c** (26.8 mg, 100 µmol) and *N*-(phenylseleno)succinimide **9** (28 mg, 1.1 equiv) according to the general procedure described in **5.2** and was obtained as a yellowish oil (21.6 mg, 51 µmol, 51% yield, e.r. = 76:24). **TLC** (heptanes:EtOAc = 10:1): R_f = 0.30 (UV). $[\alpha]_D^{23}$ = +54.7 (*c* 0.98, $CHCl_3$). **^{1}H-NMR** (300 MHz, $CDCl_3$, 298.0 K): δ / ppm = 7.43-7.36 (m, 1H), 7.33 (dd, *J* = 5.1, 3.0 Hz, 1H), 7.28-7.21 (m, 4H), 7.17 (dd, *J* = 5.1, 1.4 Hz, 1H), 7.05 (dd, *J* = 3.0, 1.4 Hz, 1H), 4.74 (d, *J* = 12.6 Hz, 1H), 4.27 (d, *J* = 12.6 Hz, 1H), 1.58 (s, 9H). **^{13}C-NMR** (75 MHz, $CDCl_3$, 298.0 K): δ / ppm = 172.1, 156.7, 138.3, 135.2, 130.6, 129.3, 126.8, 126.4, 124.2, 84.8, 59.6, 46.7, 28.5. **HRMS** (ESI-QTOF, MeOH) *m/z*: $[M+NH_4]^+$ calculated for $C_{18}H_{23}N_2O_4SSe^+$, 443.0538; found, 443.0538. **HPLC**: Chiralpak AD-H (*n*-hexane:*i*-PrOH = 20:1, flow rate 1.0 mL/min, 20 °C, λ = 254 nm), retention times t_R(minor) = 14.1 min, t_R(major) = 15.7 min.

***tert*-Butyl 4-(4-chlorophenyl)-5-oxo-4-(phenylselanyl)isoxazolidine-2-carboxylate (4d):**

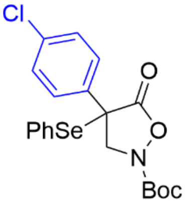

(+)-**4d**

Compound (+)-**4d** was prepared from **2d** (30.6 mg, 103 µmol) and *N*-(phenylseleno)succinimide **9** (28 mg, 1.1 equiv) according to the general procedure described in **5.2** and was obtained as a colorless oil (34.8 mg, 77 µmol, 75% yield, e.r. = 80:20). **TLC** (heptanes:EtOAc = 10:1): R_f = 0.24 (UV). $[\alpha]_D^{24}$ = +93.3 (*c* 1.01, $CHCl_3$). **^{1}H-NMR** (300 MHz, $CDCl_3$, 298.0 K): δ / ppm = 7.45-7.36 (m, 1H), 7.32-7.21 (m, 8H), 4.77 (d, *J* = 12.7 Hz, 1H), 4.27 (d, *J* = 12.7 Hz, 1H), 1.58 (s, 9H). **^{13}C-NMR** (75 MHz, $CDCl_3$, 298.0 K): δ / ppm = 171.8, 156.6, 138.3, 134.7, 134.4, 130.9. 129.5, 128.9, 128.8, 126.0, 84.9, 59.2, 48.4, 28.4. **HRMS** (ESI-QTOF, MeOH) *m/z*: $[M+NH_4]^+$ calculated for $C_{20}H_{24}ClN_2O_4Se^+$, 471.0584; found, 471.0582. **HPLC**: Chiralpak AD-H (*n*-hexane:*i*-PrOH = 20:1, flow rate 1.0 mL/min, 20 °C, λ = 254 nm), retention times t_R(minor) = 12.0 min, t_R(major) = 16.1 min.

tert-Butyl 4-(4-bromophenyl)-5-oxo-4-(phenylselanyl)isoxazolidine-2-carboxylate (4e):

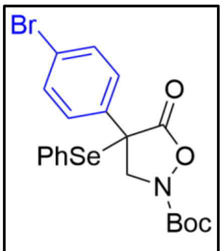

(+)-**4e**

Compound (+)-**4e** was prepared from **2e** (34.6 mg, 101 µmol) and *N*-(phenylseleno)succinimide **9** (28 mg, 1.1 equiv) according to the general procedure described in **5.2** and was obtained as a colorless oil (38.1 mg, 77 µmol, 76% yield, e.r. = 83:17). **TLC** (heptanes:EtOAc = 10:1): R_f = 0.27 (UV). $[\alpha]_D^{24}$ = +93.3 (*c* 1.00, $CHCl_3$). **^{1}H-NMR** (300 MHz, $CDCl_3$, 298.0 K): δ / ppm = 7.44-7.36 (m, 3H), 7.28-7.20 (m, 6H), 4.76 (d, *J* = 12.7 Hz, 1H), 4.26 (d, *J* = 12.7 Hz, 1H), 1.58 (s, 9H). **^{13}C-NMR** (75 MHz, $CDCl_3$, 298.0 K): δ / ppm = 171.7, 156.5, 138.3, 134.9, 131.9, 130.9, 129.5, 129.0, 126.0, 122.9, 84.9, 59.2, 48.5, 28.4. **HRMS** (ESI-QTOF, MeOH) *m/z*: $[M+NH_4]^+$ calculated for $C_{20}H_{24}BrN_2O_4Se^+$, 515.0079; found, 515.0079. **HPLC**: Chiralpak AD-H (*n*-hexane:*i*-PrOH = 20:1, flow rate 1.0 mL/min, 20 °C, λ = 254 nm), retention times t_R(minor) = 12.6 min, t_R(major) = 17.6 min.

tert-Butyl 4-(4-fluorophenyl)-5-oxo-4-(phenylselanyl)isoxazolidine-2-carboxylate (4f):

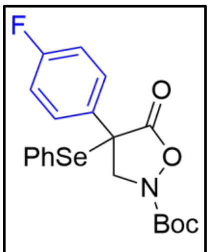

(+)-**4f**

Compound (+)-**4f** was prepared from **2f** (28.4 mg, 101 µmol) and *N*-(phenylseleno)succinimide **9** (28 mg, 1.1 equiv) according to the general procedure described in **5.2** and was obtained as a colorless oil (29.8 mg, 68 µmol, 68% yield, e.r. = 81:19). **TLC** (heptanes:EtOAc = 10:1): R_f = 0.24 (UV). $[\alpha]_D^{23}$ = +78.4 (*c* 0.99, $CHCl_3$). **^{1}H-NMR** (300 MHz, $CDCl_3$, 298.0 K): δ / ppm = 7.43-7.31 (m, 3H), 7.27-7.23 (m, 4H), 6.99-6.91 (m, 2H), 4.77 (d, *J* = 12.7 Hz, 1H), 4.29 (d, *J* = 12.7 Hz, 1H), 1.58 (s, 9H). **^{13}C-NMR** (75 MHz, $CDCl_3$, 298.0 K): δ / ppm = 172.0, 162.6 (d, $^1J_{CF}$ = 249.0 Hz), 156.6, 138.3, 131.7 (d, $^3J_{CF}$ = 3.4 Hz), 130.8, 129.4, 129.4 (d, $^3J_{CF}$ = 8.0 Hz), 126.1, 115.7 (d, $^2J_{CF}$ = 21.6 Hz), 84.9, 59.5, 48.5, 28.5. **^{19}F-NMR** (282 MHz, $CDCl_3$, 298.0 K): δ / ppm = -113.1. **HRMS** (ESI-QTOF, MeOH) *m/z*: $[M+NH_4]^+$ calculated for $C_{20}H_{24}FN_2O_4Se^+$, 455.0880; found, 455.0881. **HPLC**: Chiralpak AD-H (*n*-hexane:*i*-PrOH = 20:1, flow rate 1.0 mL/min, 20 °C, λ = 254 nm), retention times t_R(minor) = 12.1 min, t_R(major) = 14.2 min.

tert-Butyl 4-(3,4-dichlorophenyl)-5-oxo-4-(phenylselanyl)isoxazolidine-2-carboxylate (4g):

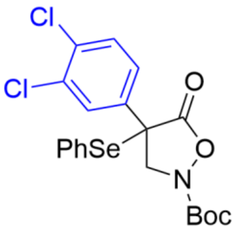

(+)-**4g**

Compound (+)-**4g** was prepared from **2g** (33.0 mg, 99 µmol) and *N*-(phenylseleno)succinimide **9** (28 mg, 1.1 equiv) according to the general procedure described in **5.2** and was obtained as a colorless oil (34.7 mg, 71 µmol, 72% yield, e.r. = 75:25). **TLC** (heptanes:EtOAc = 10:1): $R_f = 0.30$ (UV). $\mathbf{[\alpha]_D^{24}} = +77.2$ (*c* 0.96, $CHCl_3$). **¹H-NMR** (300 MHz, $CDCl_3$, 298.0 K): δ / ppm = 7.46-7.40 (m, 1H), 7.37 (d, *J* = 2.3 Hz, 1H), 7.33 (d, *J* = 8.5 Hz, 1H), 7.31-7.24 (m, 4H), 7.18 (dd, *J* = 8.5, 2.3 Hz, 1H), 4.77 (d, *J* = 12.7 Hz, 1H), 4.23 (d, *J* = 12.7 Hz, 1H), 1.59 (s, 9H). **¹³C-NMR** (75 MHz, $CDCl_3$, 298.0 K): δ / ppm = 171.4, 156.5, 138.4, 136.0, 133.0, 132.9, 131.2, 130.6, 129.6, 129.4, 126.6, 125.8, 85.1, 59.0, 47.6, 28.5 **HRMS** (ESI-QTOF, MeOH) *m/z*: $[M+NH_4]^+$ calculated for $C_{20}H_{23}Cl_2N_2O_4Se^+$, 505.0195; found, 505.0195. **HPLC**: Chiralpak AD-H (*n*-hexane:*i*-PrOH = 20:1, flow rate 1.0 mL/min, 20 °C, λ = 254 nm), retention times t_R(minor) = 10.2 min, t_R(major) = 13.1 min.

tert-Butyl 5-oxo-4-(phenylselanyl)-4-(_p_-tolyl)isoxazolidine-2-carboxylate (4i):

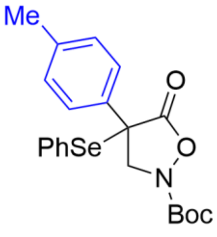

(+)-**4i**

Compound (+)-**4i** was prepared from **2i** (28.4 mg, 102 µmol) and *N*-(phenylseleno)succinimide **9** (28 mg, 1.1 equiv) according to the general procedure described in **5.2** and was obtained as a reddish oil (18.3 mg, 42 µmol, 41% yield, e.r. = 74:26). **TLC** (heptanes:EtOAc = 10:1): $R_f = 0.27$ (UV). $\mathbf{[\alpha]_D^{23}} = +44.6$ (*c* 0.99, $CHCl_3$). **¹H-NMR** (500 MHz, $CDCl_3$, 298.0 K): δ / ppm = 7.40-7.36 (m, 1H), 7.30-7.21 (m, 6H), 7.08 (d, *J* = 8.1 Hz, 2H), 4.71 (d, *J* = 12.6 Hz, 1H), 4.32 (d, *J* = 12.6 Hz, 1H), 2.32 (s, 3H), 1.55 (s, 9H). **¹³C-NMR** (126 MHz, $CDCl_3$, 298.0 K): δ / ppm = 172.2, 156.6, 138.6, 138.3, 132.6, 130.6, 129.5, 129.3, 127.3, 126.4, 84.6, 59.6, 49.5, 28.4, 21.5. **HRMS** (ESI-QTOF, MeOH) *m/z*: $[M+NH_4]^+$ calculated for $C_{21}H_{27}N_2O_4Se^+$, 451.1131; found, 451.1128. **HPLC**: Chiralpak AD-H (*n*-hexane:*i*-PrOH = 20:1, flow rate 1.0 mL/min, 20 °C, λ = 254 nm), retention times t_R(minor) = 9.2 min, t_R(major) = 12.6 min.

***tert*-Butyl 5-oxo-4-phenyl-4-(*o*-tolylselanyl)isoxazolidine-2-carboxylate (4j):**

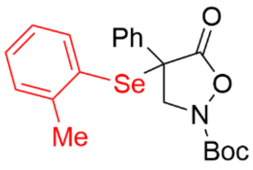

(+)-**4j**

Compound (+)-**4j** was prepared from **2a** (26.5 mg, 101 µmol) and *N*-(*o*-tolylseleno)phthalimide (38 mg, 1.2 equiv) according to the general procedure described in **5.2** and was obtained as a colorless oil (37.6 mg, 87 µmol, 76% yield, e.r. = 76:24). **TLC** (heptanes:EtOAc = 10:1): R_f = 0.32 (UV). **[α]$_D$^{24}** = +79.5 (*c* 0.99, $CHCl_3$). **^{1}H-NMR** (500 MHz, $CDCl_3$, 298.0 K): δ / ppm = 7.36-7.33 (m, 2H), 7.30-7.23 (m, 5H), 7.19 (d, *J* = 7.4 Hz, 1H), 7.05-7.01 (m, 1H), 4.87 (d, *J* = 12.7 Hz, 1H), 4.32 (d, *J* = 12.7 Hz, 1H), 2.07 (s, 3H), 1.58 (s, 9H). **^{13}C-NMR** (126 MHz, $CDCl_3$, 298.0 K): δ / ppm = 171.6, 156.7, 144.9, 140.0, 135.8, 131.2, 130.5, 128.8, 128.6, 127.3, 127.2, 126.7, 84.7, 59.8, 48.3, 28.4, 23.0. **HRMS** (ESI-QTOF, MeOH) *m/z*: $[M+NH_4]^+$ calculated for $C_{21}H_{27}N_2O_4Se^+$, 451.1131; found, 451.1125. **HPLC**: Chiralpak AD-H (*n*-hexane:*i*-PrOH = 20:1, flow rate 1.0 mL/min, 10 °C, λ = 240 nm), retention times t_R(minor) = 9.1 min, t_R(major) = 11.8 min.

***tert*-Butyl 5-oxo-4-phenyl-4-(*p*-tolylselanyl)isoxazolidine-2-carboxylate (4k):**

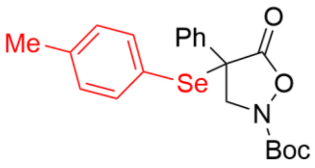

(+)-**4k**

Compound (+)-**4k** was prepared from **2a** (26.4 mg, 100 µmol) and *N*-(*p*-tolylseleno)phthalimide (38 mg, 1.2 equiv) according to the general procedure described in **5.2** and was obtained as a colorless oil (8.3 mg, 19 µmol, 19% yield, e.r. = 72:28). **TLC** (heptanes:EtOAc = 10:1): R_f = 0.32 (UV). **[α]$_D$^{24}** = +44.6 (*c* 0.83, $CHCl_3$). **^{1}H-NMR** (300 MHz, $CDCl_3$, 298.0 K): δ / ppm = 7.41-7.34 (m, 2H), 7.30-7.25 (m, 3H), 7.14 (d, *J* = 7.9 Hz, 2H), 7.03 (d, *J* = 7.9 Hz, 2H), 4.73 (d, *J* = 12.7 Hz, 1H), 4.32 (d, *J* = 12.7 Hz, 1H), 2.33 (s, 3H), 1.56 (s, 9H). **^{13}C-NMR** (75 MHz, $CDCl_3$, 298.0 K): δ / ppm = 172.1, 156.7, 141.1, 138.2, 135.9, 130.2, 128.8, 128.5, 127.4, 122.9, 84.6, 59.4, 49.3, 28.4, 21.7. **HRMS** (ESI-QTOF, MeOH) *m/z*: $[M+NH_4]^+$ calculated for $C_{21}H_{27}N_2O_4Se^+$, 451.1131; found, 451.1129. **HPLC**: Chiralpak AD-H (*n*-hexane:*i*-PrOH = 20:1, flow rate 1.0 mL/min, 10 °C, λ = 240 nm), retention times

tert-Butyl 4-(benzylselanyl)-5-oxo-4-phenylisoxazolidine-2-carboxylate (4l):

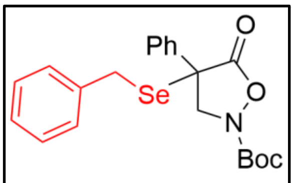

(+)-**4l**

Compound (+)-**4l** was prepared from **2a** (26.6 mg, 101 µmol) and *N*-(benzylseleno)phthalimide (38 mg, 1.2 equiv) according to the general procedure described in **5.2** and was obtained as a colorless oil (9.7 mg, 22 µmol, 22% yield, e.r. = 61:39). **TLC** (heptanes:EtOAc = 10:1): R_f = 0.32 (UV). **$[\alpha]_D^{23}$** = +17.3 (*c* 0.97, $CHCl_3$). **^{1}H-NMR** (500 MHz, $CDCl_3$, 298.0 K): δ / ppm = 7.70-7.67 (m, 2H), 7.42-7.38 (m, 2H), 7.36-7.32 (m, 1H), 7.22-7.15 (m, 3H), 7.11-7.08 (m, 2H), 4.65 (d, *J* = 12.7 Hz, 1H), 4.18 (d, *J* = 12.7 Hz, 1H), 4.04 (d, *J* = 10.8 Hz, 1H), 3.69 (d, *J* = 10.8 Hz, 1H), 1.51 (s, 9H). **^{13}C-NMR** (126 MHz, $CDCl_3$, 298.0 K): δ / ppm = 172.8, 156.5, 136.2, 135.7, 129.6, 129.3, 128.9, 127.8, 127.6, 84.8, 61.5, 47.2, 30.5, 28.4. **HRMS** (ESI-QTOF, MeOH) *m/z*: $[M+NH_4]^+$ calculated for $C_{21}H_{27}N_2O_4Se^+$, 451.1131; found, 451.1133. **HPLC**: Chiralpak OD-H (*n*-hexane:*i*-PrOH = 20:1, flow rate 1.0 mL/min, 10 °C, λ = 220 nm), retention times t_R(minor) = 11.9 min, t_R(major) = 14.1 min.

7. Further Transformations of *N*-Boc 4,4-phenyl(phenylselanyl)isoxazolidin-5-one 4a

tert-Butyl (3-((4-chlorobenzyl)amino)-3-oxo-2-phenyl-2-(phenylselanyl)propyl)(hydroxy)carbamate (9a):

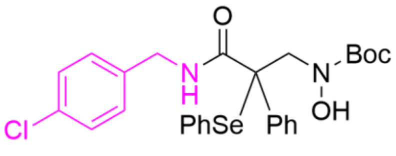

(+)-**9a**

Compound (+)-**9a** was prepared in analogy to a reported procedure[5] by treatment of enantioenriched selenation product (+)-**4a** (38.4 mg, 92 µmol, e.r. = 83:17) with 4-chlorobenzylamine (60 µL, 5 equiv) in *t*BuOH (2 mL, 0.05 M). The crude product was purified *via* flash column chromatography (silica gel, heptanes/EtOAc = 2/1) to obtain amide (+)-**9a** as a white solid (42.8 mg, 76 µmol, 83% yield, e.r. = 83:17). **TLC** (heptanes:EtOAc = 2:1): R_f = 0.44 (UV). $[\alpha]_D^{23}$ = +33.2 (*c* 1.04, $CHCl_3$). **^{1}H-NMR** (300 MHz, $CDCl_3$, 298.0 K): δ / ppm = 8.12 (s, 1H), 7.40-7.15 (m, 15H), 6.83 (bt, *J* = 5.7 Hz, 1H), 4.57 (d, *J* = 15.2 Hz, 1H), 4.39 (d, *J* = 6.0 Hz, 2H), 4.07 (d, *J* = 15.2 Hz, 1H), 1.27 (s, 9H). **^{13}C-NMR** (75 MHz, $CDCl_3$, 298.0 K): δ / ppm = 173.9, 155.3, 138.6, 137.5, 136.2, 133.9, 130.0, 129.5, 129.2, 128.8, 128.7, 128.4, 126.8, 81.6, 61.9, 59.1, 44.3, 28.4. **HRMS** (ESI-QTOF, MeOH) *m/z*: $[M+Na]^+$ calculated for $C_{27}H_{29}ClN_2NaO_4Se^+$, 583.0873; found, 583.0875. **HPLC**: Chiralpak OD-H (*n*-hexane:*i*-PrOH = 2:1, flow rate 0.5 mL/min, 20 °C, λ = 254 nm), retention times t_R(minor) = 13.8 min, t_R(major) = 27.4 min.

tert-Butyl hydroxy(3-((4-methoxybenzyl)amino)-3-oxo-2-phenyl-2-(phenylselanyl)propyl)carbamate (9b):

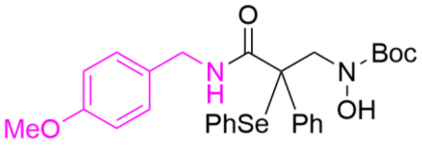

(+)-**9b**

Analogously prepared to **9a**, using 4-methoxybenzylamine (60 µL, 5 equiv). Amide (+)-**9b** was obtained as an off-white solid (42.8 mg, 77 µmol, 84% yield, e.r. = 83:17). **TLC** (heptanes:EtOAc = 2:1): R_f = 0.37 (UV). $[\alpha]_D^{23}$ = +29.4 (*c* 1.07, $CHCl_3$). **^{1}H-NMR** (500 MHz, $CDCl_3$, 298.0 K): δ / ppm = 8.40 (s, 1H), 7.42-7.39 (m, 2H), 7.38-7.33 (m, 3H), 7.29-7.24 (m, 4H), 7.23-7.16 (m, 4H), 6.88-6.84 (m, 2H), 6.79 (bs, 1H), 4.59 (d, *J* = 15.2 Hz, 1H), 4.38 (dd, *J* = 5.6, 2.4 Hz, 2H), 4.02 (d, *J* = 15.2 Hz, 1H), 3.81 (s, 3H), 1.26 (s, 9H). **^{13}C-NMR** (126 MHz, $CDCl_3$, 298.0 K): δ / ppm = 173.9, 159.6, 155.0, 138.6, 137.5, 130.0, 129.5, 128.7, 128.3, 126.8, 114.5, 81.4, 62.0, 59.4, 55.7, 44.5, 28.4. **HRMS** (ESI-QTOF, MeOH) *m/z*: $[M+Na]^+$ calculated for $C_{28}H_{32}N_2NaO_5Se^+$, 579.1369; found,

[5] P. Zebrowski, I. Eder, A. Eitzinger, S. C. Mallojjala and M. Waser, *ACS Org. Inorg. Au* 10.1021/acsorginorgau.1c00025.

579.1364. **HPLC**: Chiralpak OD-H (*n*-hexane:*i*-PrOH = 2:1, flow rate 0.5 mL/min, 20 °C, λ = 254 nm), retention times t_R(minor) = 19.1 min, t_R(major) = 31.3 min.

Methyl 3-((*tert*-butoxycarbonyl)(hydroxy)amino)-2-phenyl-2-(phenylselanyl)propanoate (10):

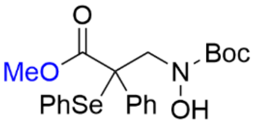

(-)-**10**

A reaction vial was charged with (+)-**4a** (42.4 mg, 101 µmol, e.r. = 83:17), MeOH (1 mL, 0.1 M) and Amberlyst A21 (100 mg). The heterogenous mixture was stirred vigorously for 24 h at room temperature, whereupon it was filtered over cotton and concentrated under reduced pressure. The crude product was purified by preparative TLC (silica gel, heptanes/EtOAc = 2/1) to obtain (-)-**10** as a colorless oil (21.0 mg, 47 µmol, 46% yield, e.r. = 83:17). **TLC** (heptanes:EtOAc = 2:1): R_f = 0.29 (UV). **[α]$_D^{24}$** = -21.1 (*c* 0.43, $CHCl_3$). **^{1}H-NMR** (300 MHz, $CDCl_3$, 298.0 K): δ / ppm = 7.35-7.29 (m, 3H), 7.25-7.14 (m, 7H), 6.41 (s, 1H), 4.43 (d, *J* = 14.8 Hz, 1H), 4.25 (d, *J* = 14.8 Hz, 1H), 3.70 (s, 3H), 1.38 (s, 9H). **^{13}C-NMR** (176 MHz, $CDCl_3$, 298.0 K): δ / ppm = 173.6, 156.0, 138.3, 129.8, 129.0, 128.4, 128.3, 127.9, 127.1, 82.3, 60.3, 57.0, 53.3, 28.4. **HRMS** (ESI-QTOF, MeOH) *m/z*: $[M+Na]^+$ calculated for $C_{21}H_{25}NNaO_5Se^+$, 474.0790; found, 474.0795. **HPLC**: YMC Chiral ART Cellulose-SB (*n*-hexane:*i*-PrOH = 4:1, flow rate 0.5 mL/min, 20 °C, λ = 254 nm), retention times t_R(major) = 22.7 min, t_R(minor) = 26.2 min.

***tert*-Butyl 5-oxo-4-phenylisoxazole-2(5*H*)-carboxylate (11):**

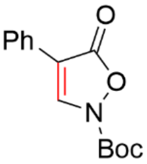

11

A flame-dried Schlenk tube was charged with **4a** (40.8 mg, 98 µmol) and dry CH_2Cl_2 (1 mL, 0.1 M). mCPBA (49.6 mg, 2.3 equiv, ≤77%) was added and the mixture was stirred for 24 h at room temperature. The reaction was quenched by addition of sat. $Na_2S_2O_3$ solution (1 mL) and the organic phase was washed with sat. $Na_2S_2O_3$ (2x 1 mL) and sat. $NaHCO_3$ (3x 1 mL) solution, dried over anhydrous Na_2SO_4, filtered and concentrated under reduced pressure. **11** was obtained as a off-white solid (21.2 mg, 81 µmol, 83% yield). **TLC** (heptanes:EtOAc = 5:1): R_f = 0.34 (UV). **^{1}H-NMR** (300 MHz, $CDCl_3$, 298.0 K): δ / ppm = 8.51 (s, 1H), 7.80-7.75 (m, 2H), 7.45-7.30 (m, 3H), 1.63 (s, 9H). **^{13}C-NMR** (75 MHz, $CDCl_3$, 298.0 K): δ / ppm = 166.6, 144.9, 138.9, 129.2, 128.7, 128.0, 126.1, 107.5, 87.5, 28.4. **HRMS** (ESI-QTOF, MeOH) *m/z*: $[M+Na]^+$ calculated for $C_{14}H_{15}NNaO_4^+$, 284.0893; found, 284.0893.

8. Single-Crystal Analysis

Single crystals suitable for single crystal X-ray diffraction were obtained by re-crystallisation from diethyl ether. Single-crystal structure analysis was carried out at room temperature on a Bruker D8 Quest ECO diffractometer with graphite-monochromated MoK_α radiation (λ = 0.71073 Å). The structures were solved by direct methods (SHELXS-97[6]) and refined by full-matrix least-squares on F^2 (SHELXL-2014/7[7]). The H atoms were calculated geometrically, and a riding model was applied in the refinement process. Crystallographic details for *(rac)*-**4a** can be found in Table 1. CCDC 2121570 contain the supplementary crystallographic data. This information can be obtained free of charge via https://www.ccdc.cam.ac.uk/structures/

Table 1: X-ray diffraction crystal data, data collection and structure refinement for the compound *(rac)*-**4a**.

**Compound**	***(rac)*-4a**
Empirical formula	$C_{20}H_{21}NO_4Se$
Formula weight ($g \cdot mol^{-1}$)	418.34
Crystal system	triclinic
Space group	$P\bar{1}$
Temp (K)	293
a (Å)	9.198(5)
b (Å)	10.355(7)
c (Å)	12.174(7)
α (°)	69.365(16)
β (°)	76.44(2)
γ (°)	64.56(2)
V ($Å^3$)	975.2(10)
Z	2
ρ_{calc} ($g \cdot cm^{-3}$)	1.425
Reflns collected	60514
Indep. reflns	3429
Obs. reflns [I > 2σ(I)]	3176
Param. refin./restr.	238 / 0
Absorption correction	multi-scan
R_1	0.027

6 Sheldrick, GM (1997) SHELXS-97, Program for the Solution of Crystal Structures, Göttingen, Germany. See also: Sheldrick, GM (1990) Acta Crystallogr A 46:467.
7 Sheldrick, GM (2015) Acta Crystallogr A 71:3.

**Compound**	***(rac)*-4a**
wR_2	0.075
CCDC	2121570

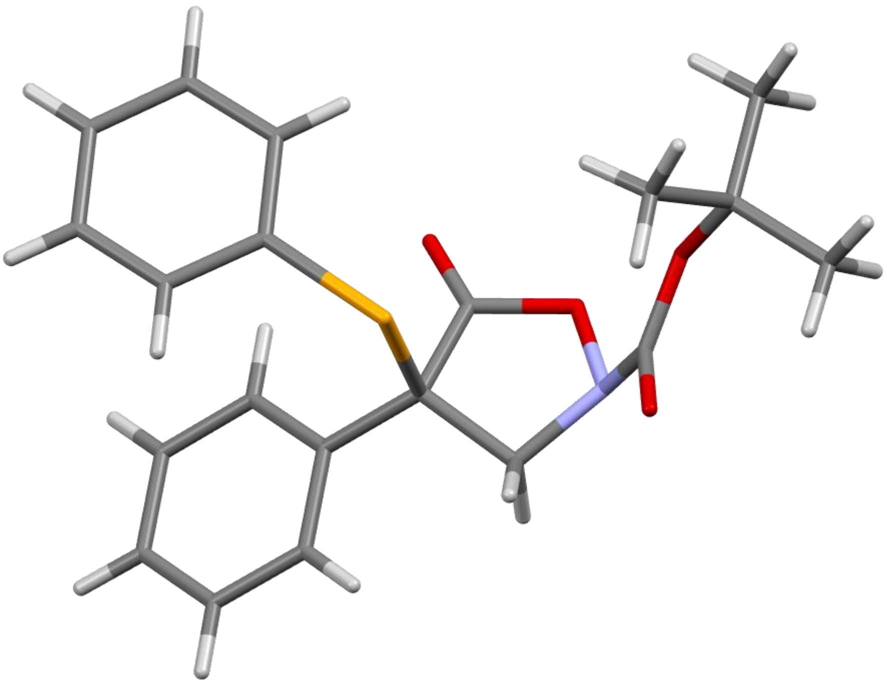

Figure 1: Single crystal structure of *(rac)*-**4a**.

9. NMR Spectra of Selenation Products

NMR spectra of compound **3a**

^{1}H-NMR (300 MHz, $CDCl_3$, 298 K)

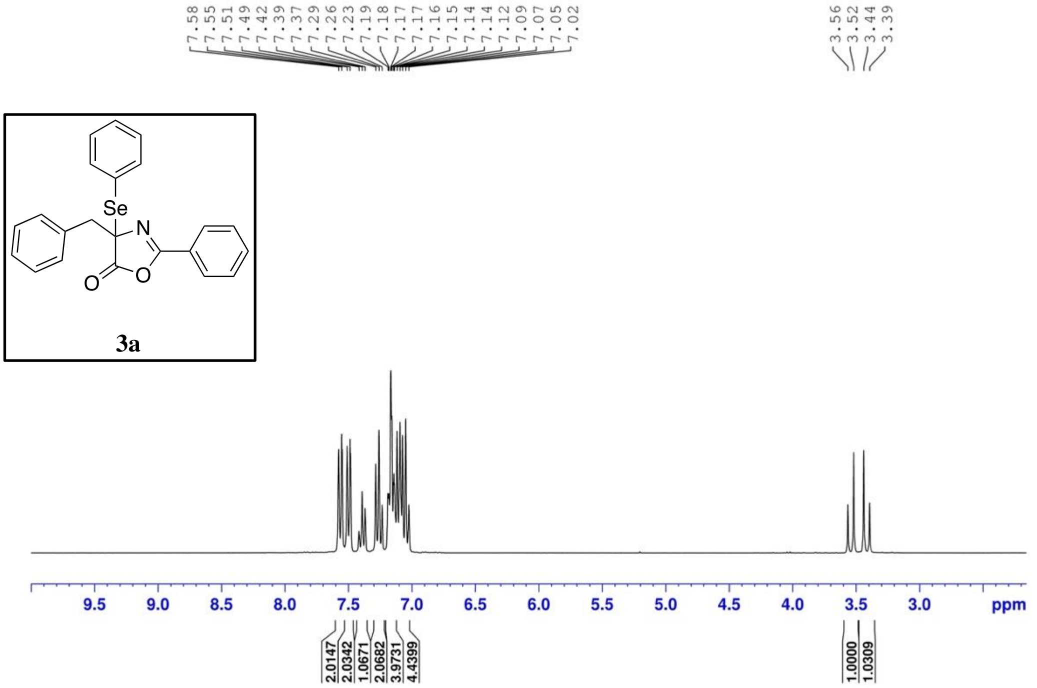

^{13}C-NMR (75 MHz, $CDCl_3$, 298 K)

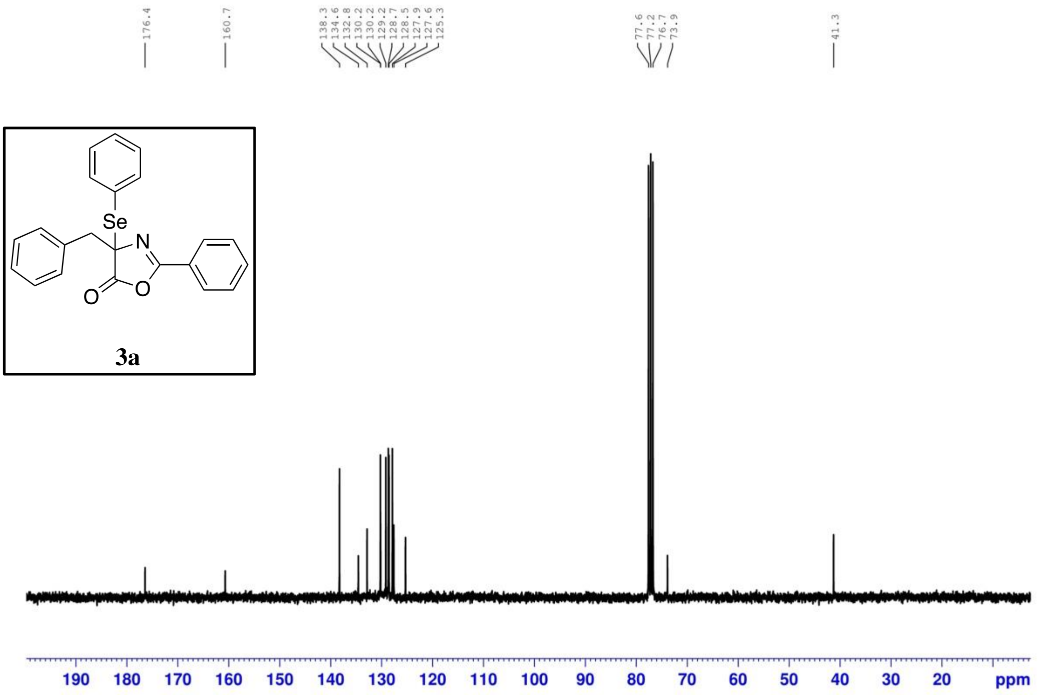

NMR spectra of compound **3c**

^{1}H-NMR (700 MHz, $CDCl_3$, 298 K)

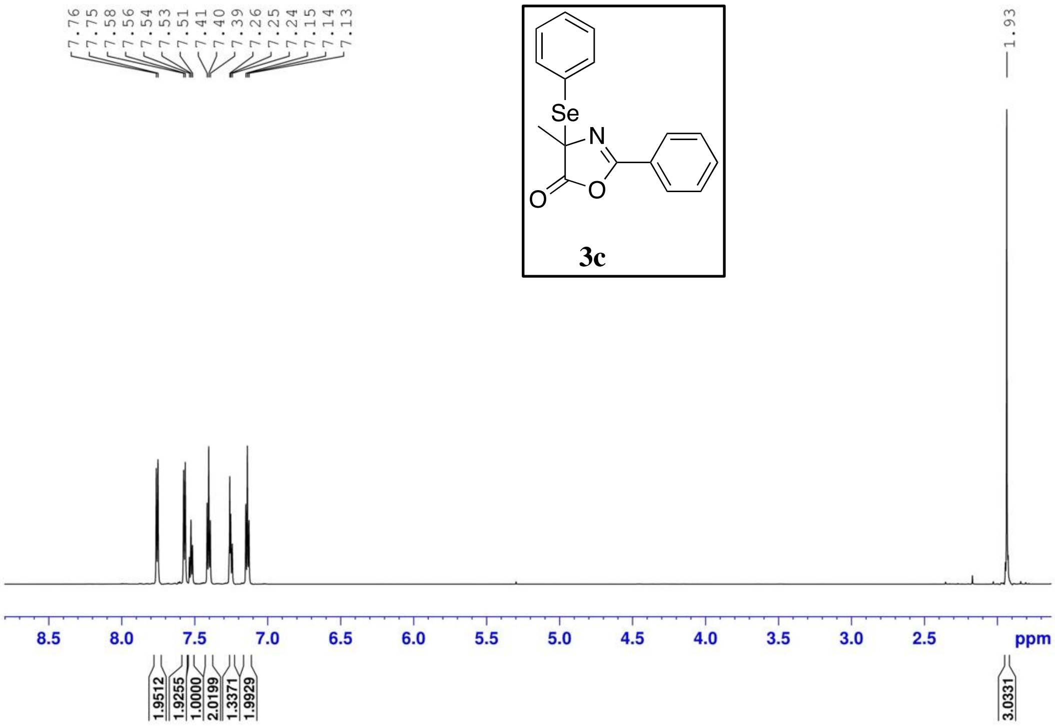

^{13}C-NMR (176 MHz, $CDCl_3$, 298 K)

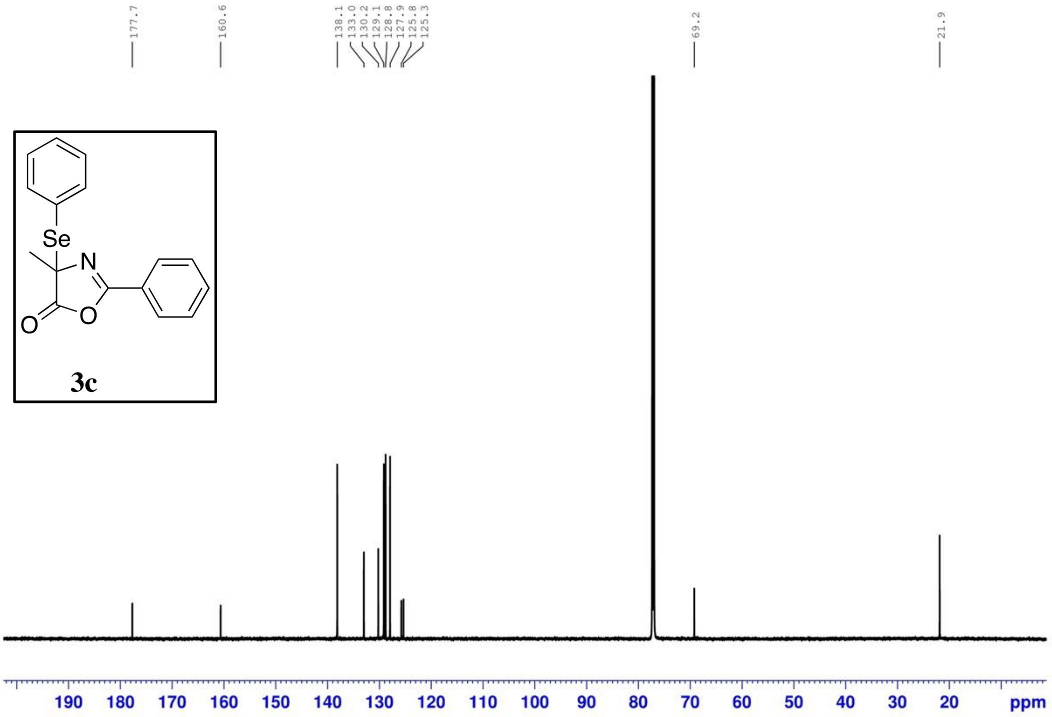

NMR spectra of compound **3d**

^{1}H-NMR (500 MHz, $CDCl_3$, 298 K)

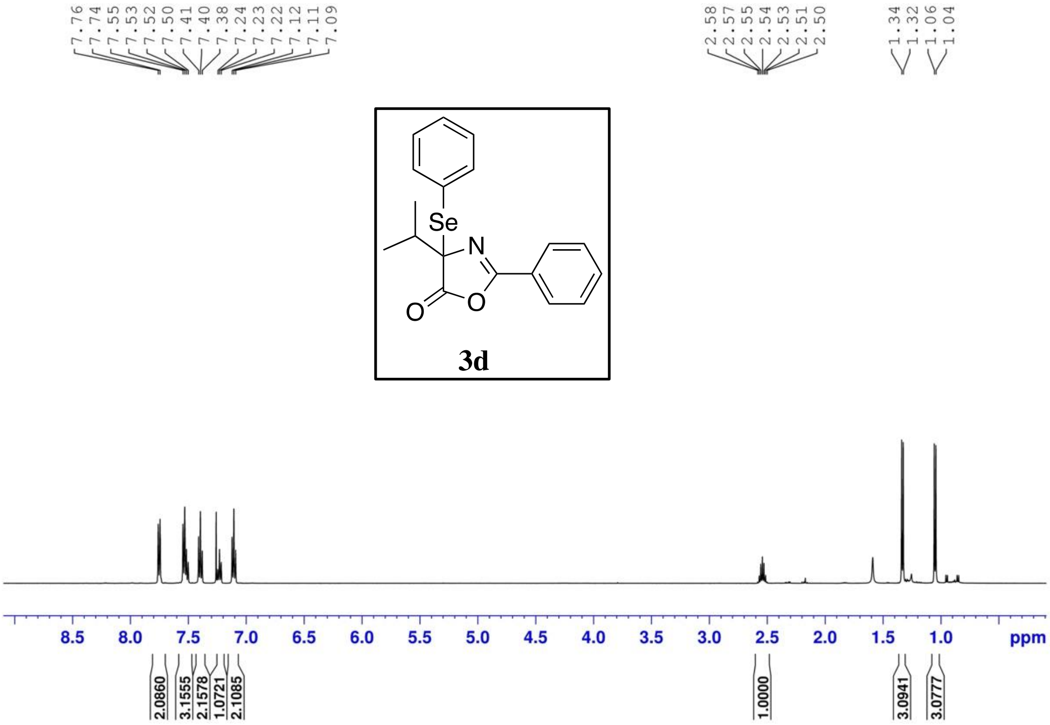

^{13}C-NMR (126 MHz, $CDCl_3$, 298 K)

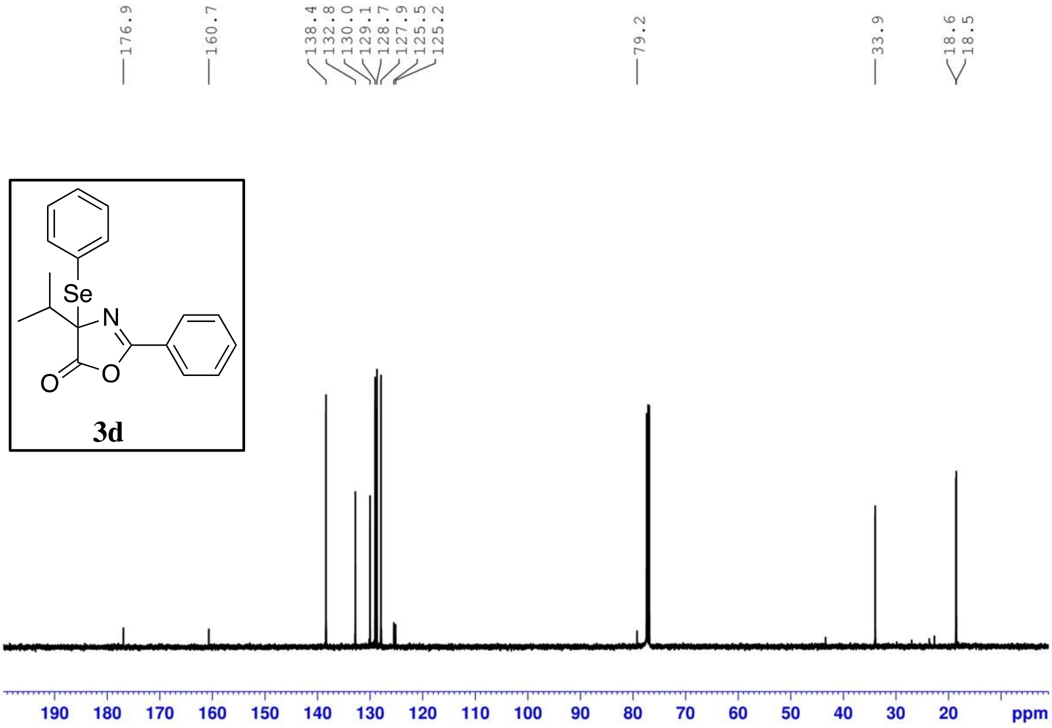

NMR spectra of compound **3e**

^{1}H-NMR (500 MHz, $CDCl_3$, 298 K)

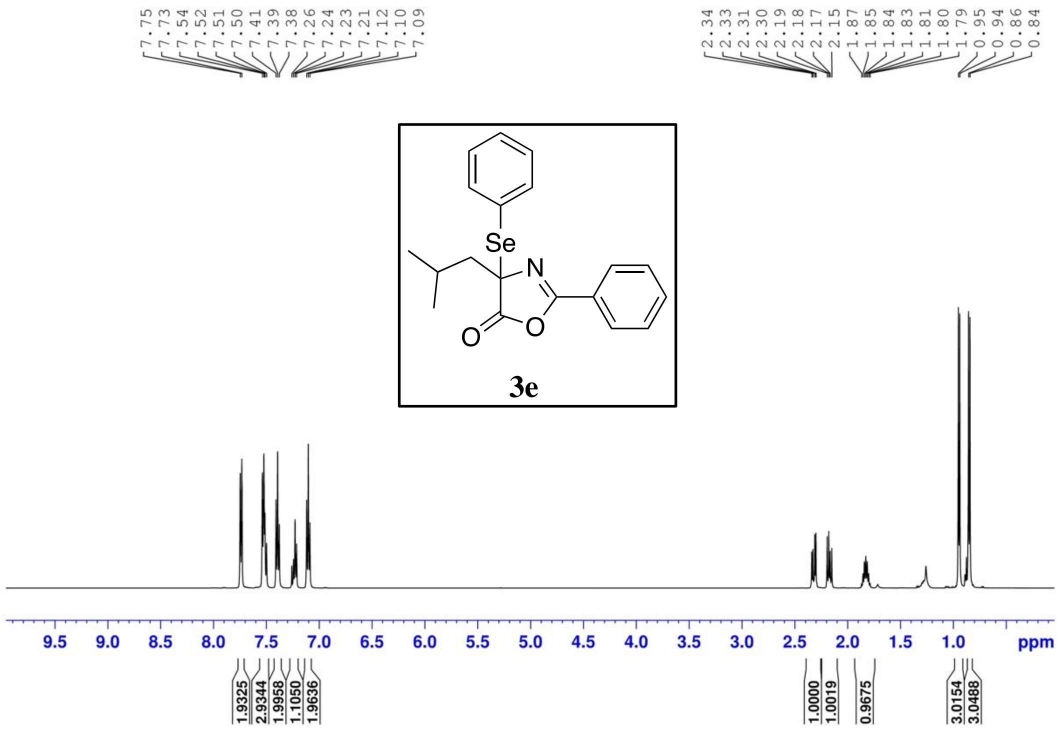

^{13}C-NMR (126 MHz, $CDCl_3$, 298 K)

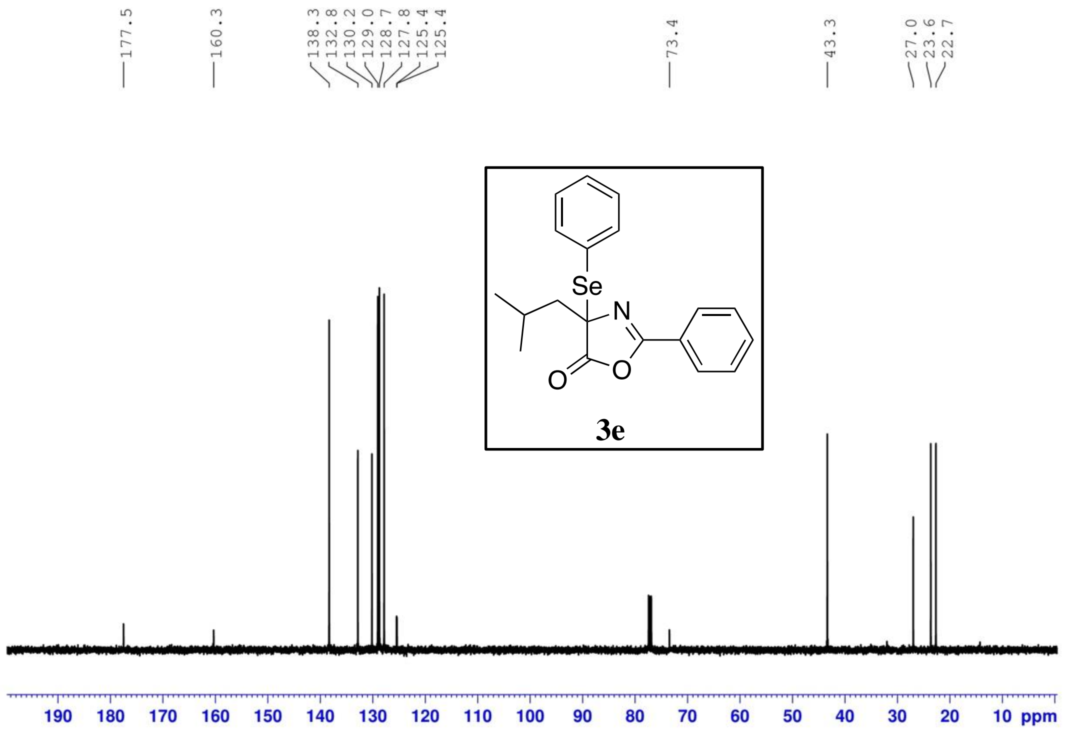

NMR spectra of compound **3f**

^{1}H-NMR (300 MHz, $CDCl_3$, 298 K)

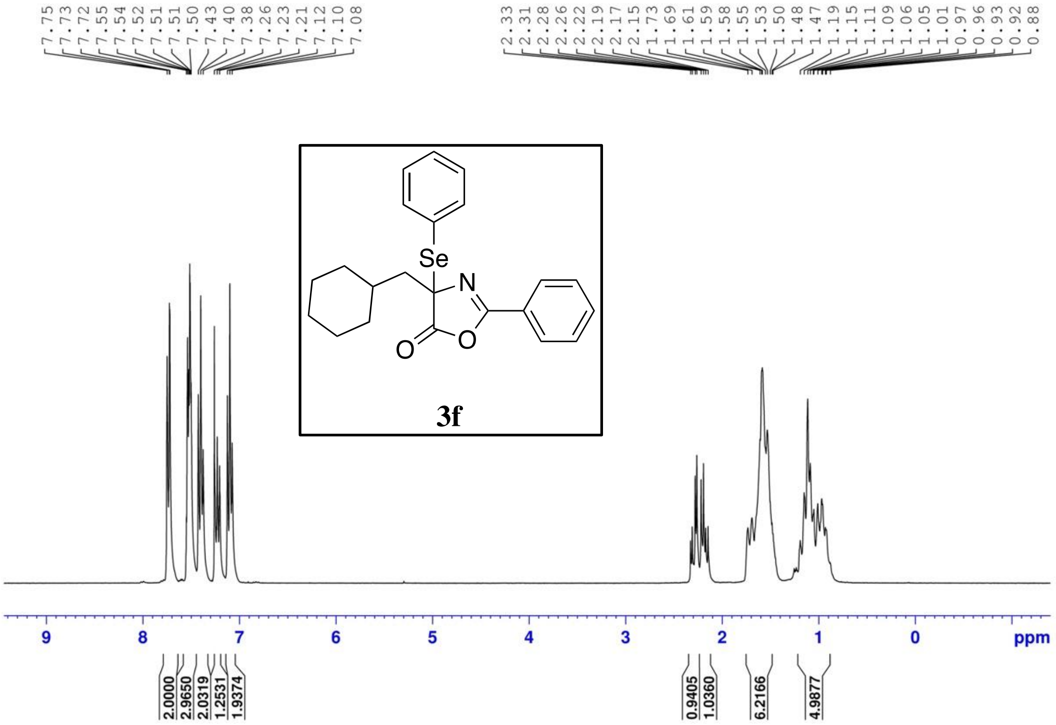

^{13}C-NMR (75 MHz, $CDCl_3$, 298 K)

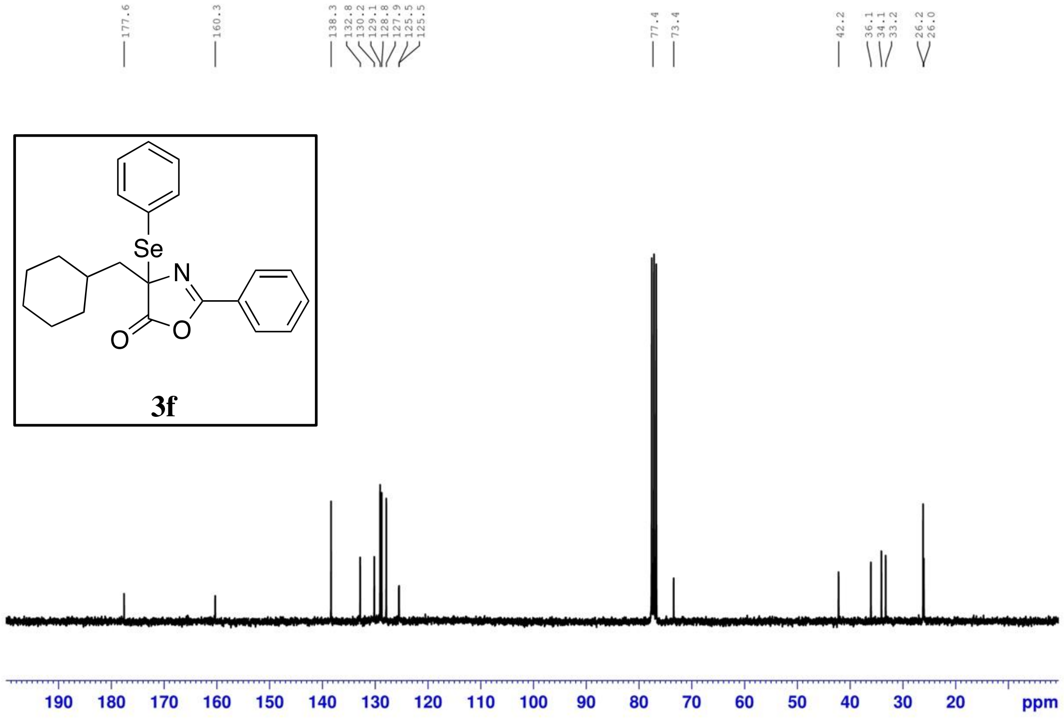

NMR spectra of compound **3g**

^{1}H-NMR (300 MHz, $CDCl_3$, 298 K)

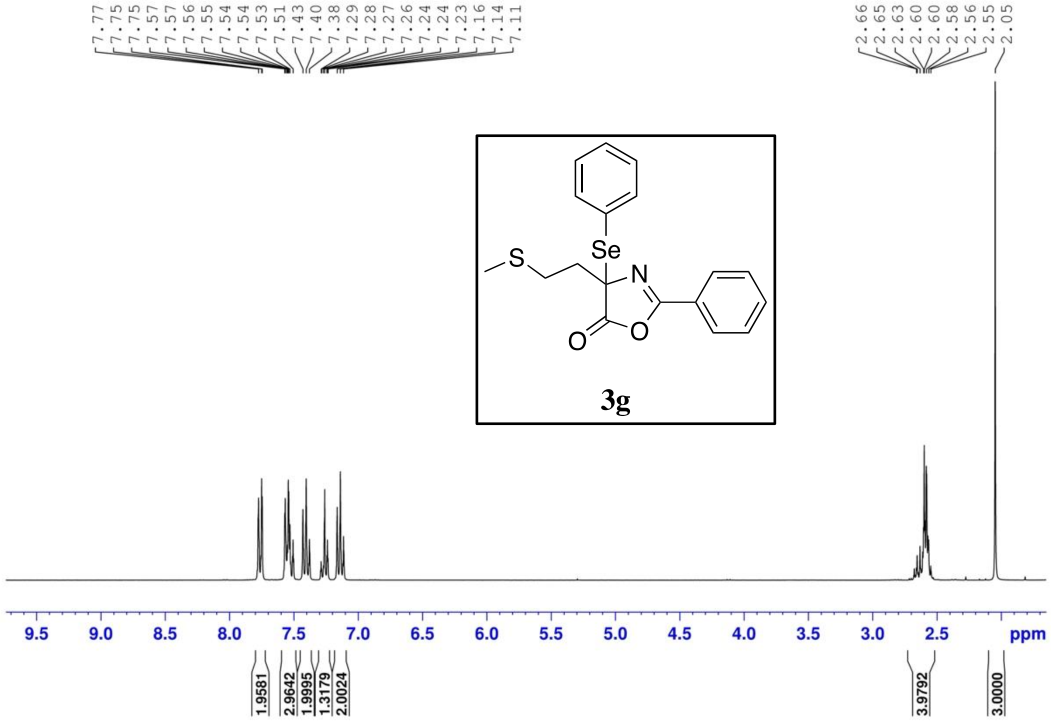

^{13}C-NMR (75 MHz, $CDCl_3$, 298 K)

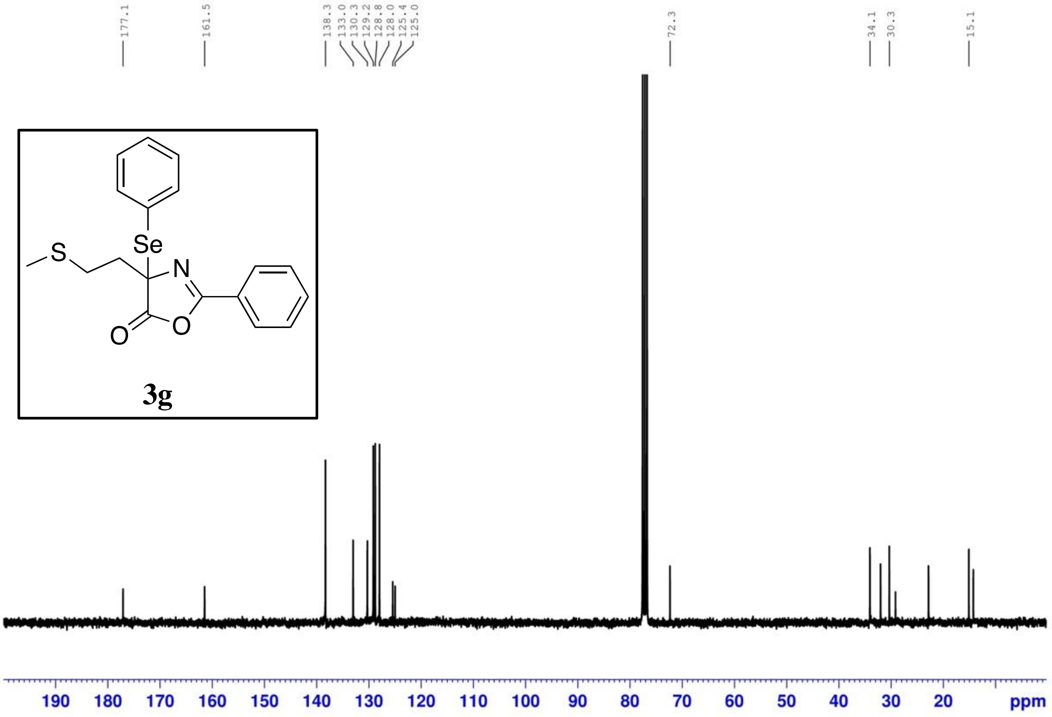

NMR spectra of compound **3h**

^{1}H-NMR (300 MHz, $CDCl_3$, 298 K)

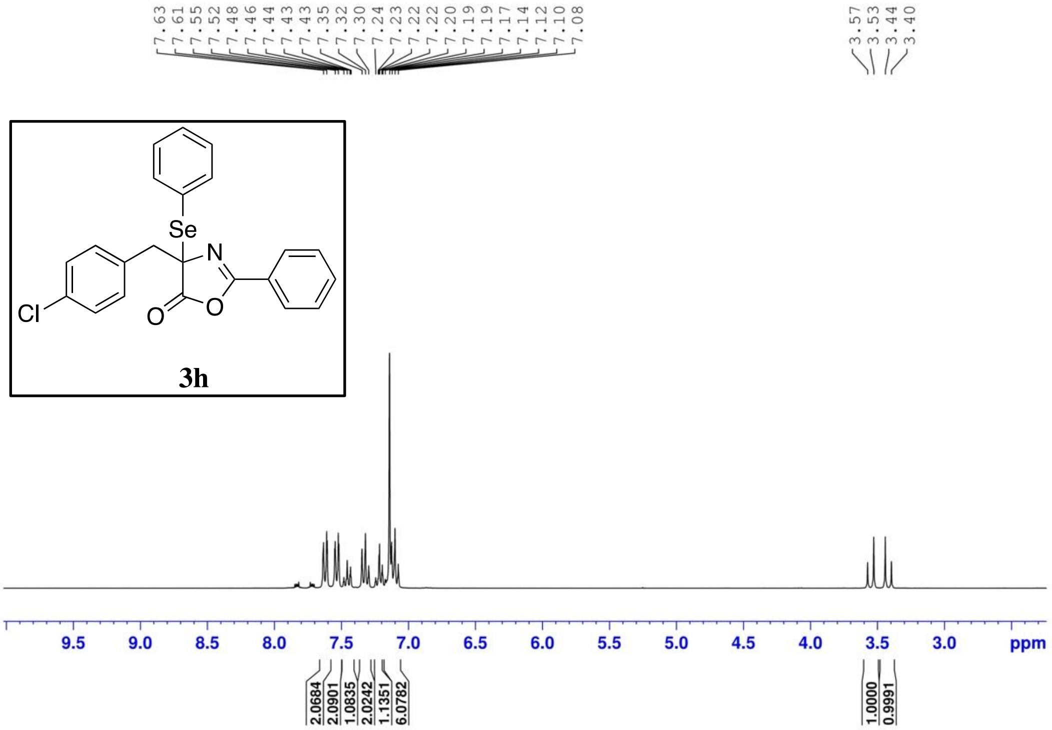

^{13}C-NMR (75 MHz, $CDCl_3$, 298 K)

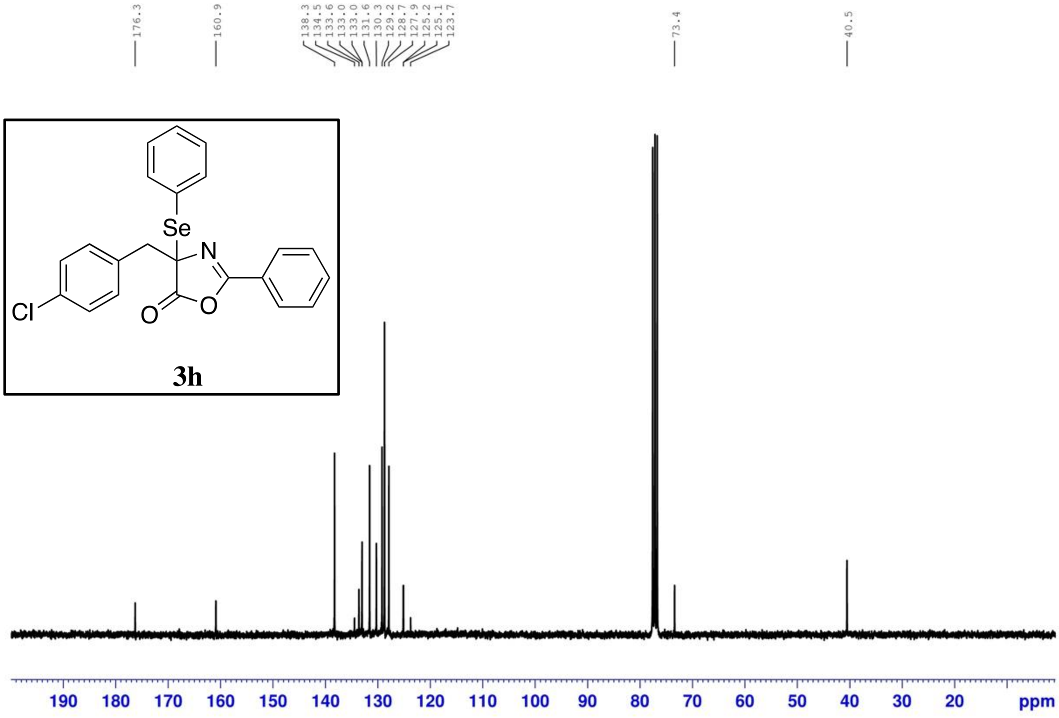

NMR spectra of compound **3i**

[1]**H-NMR** (300 MHz, $CDCl_3$, 298 K)

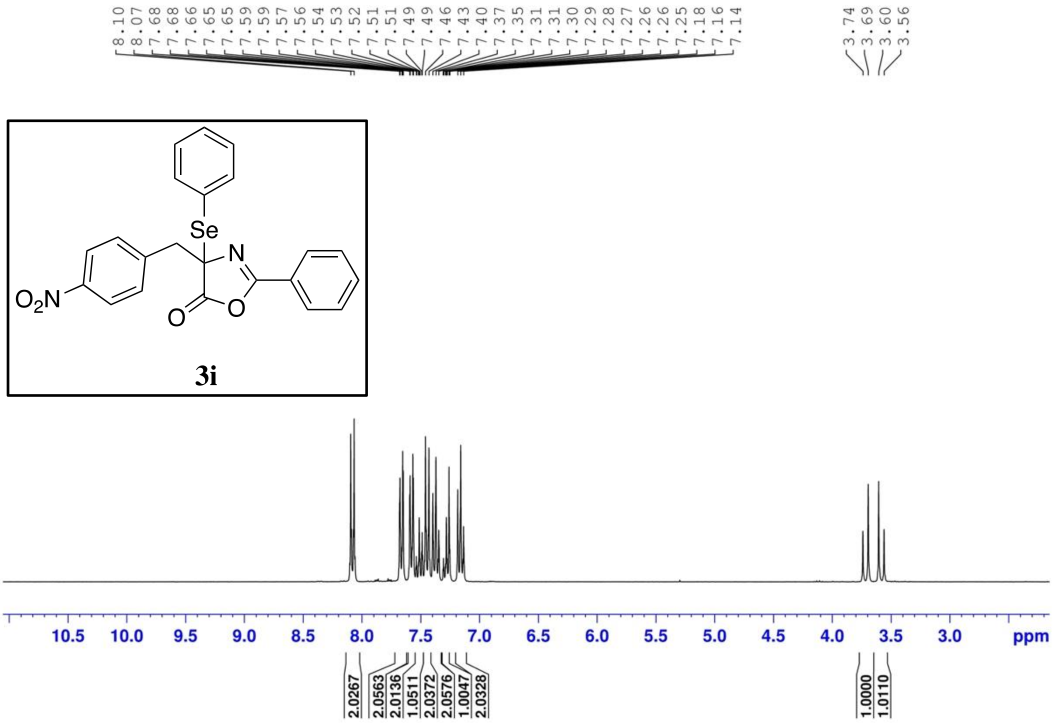

[13]**C-NMR** (75 MHz, $CDCl_3$, 298 K)

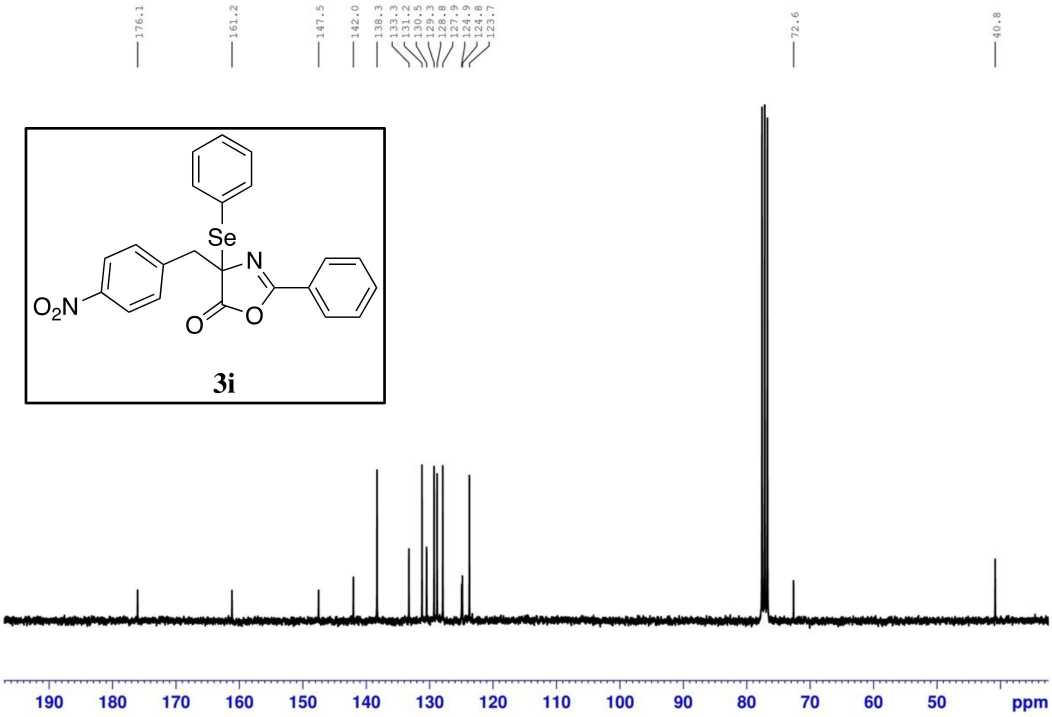

NMR spectra of compound **3j**

^{1}**H-NMR** (700 MHz, $CDCl_3$, 298 K)

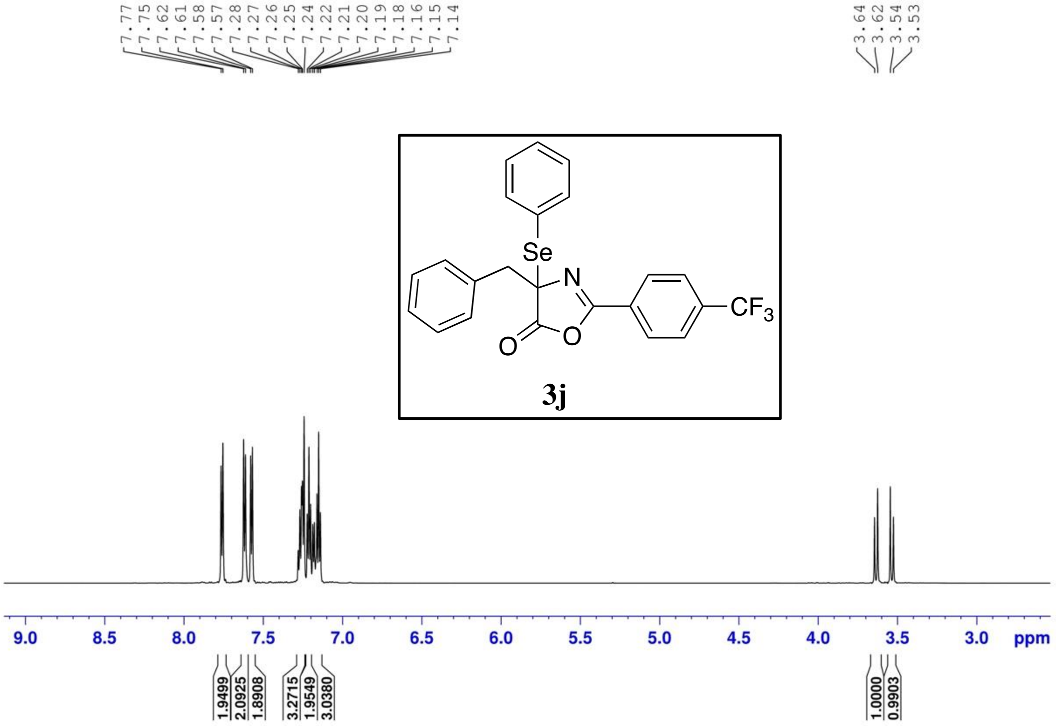

^{13}**C-NMR** (176 MHz, $CDCl_3$, 298 K)

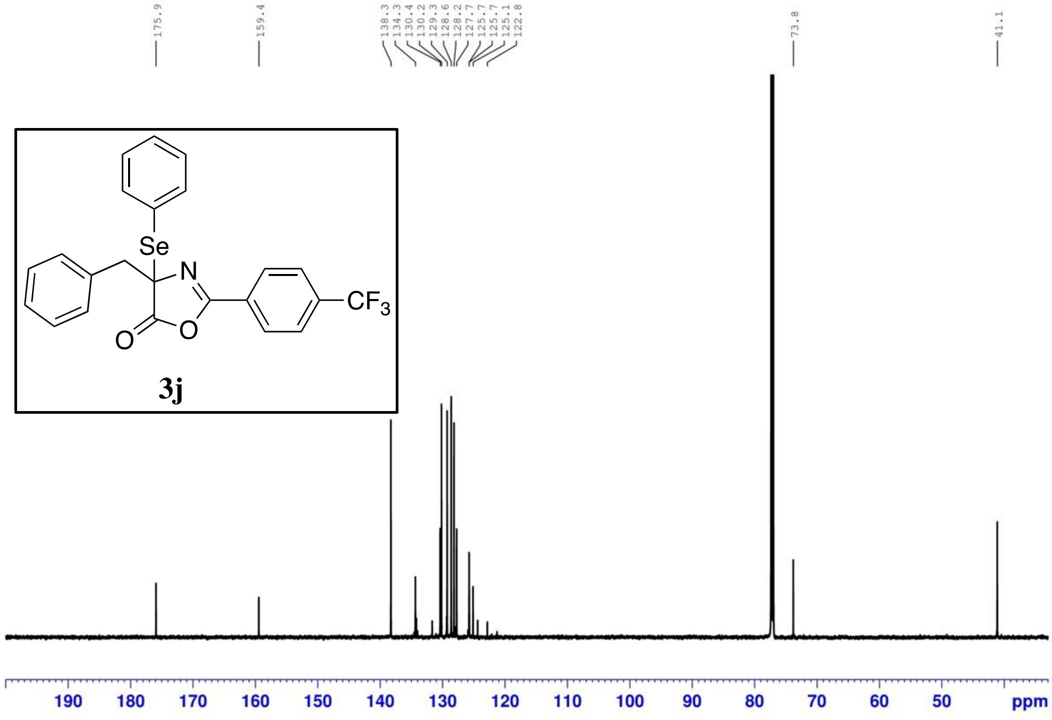

^{19}F-NMR (471 MHz, $CDCl_3$, 298 K)

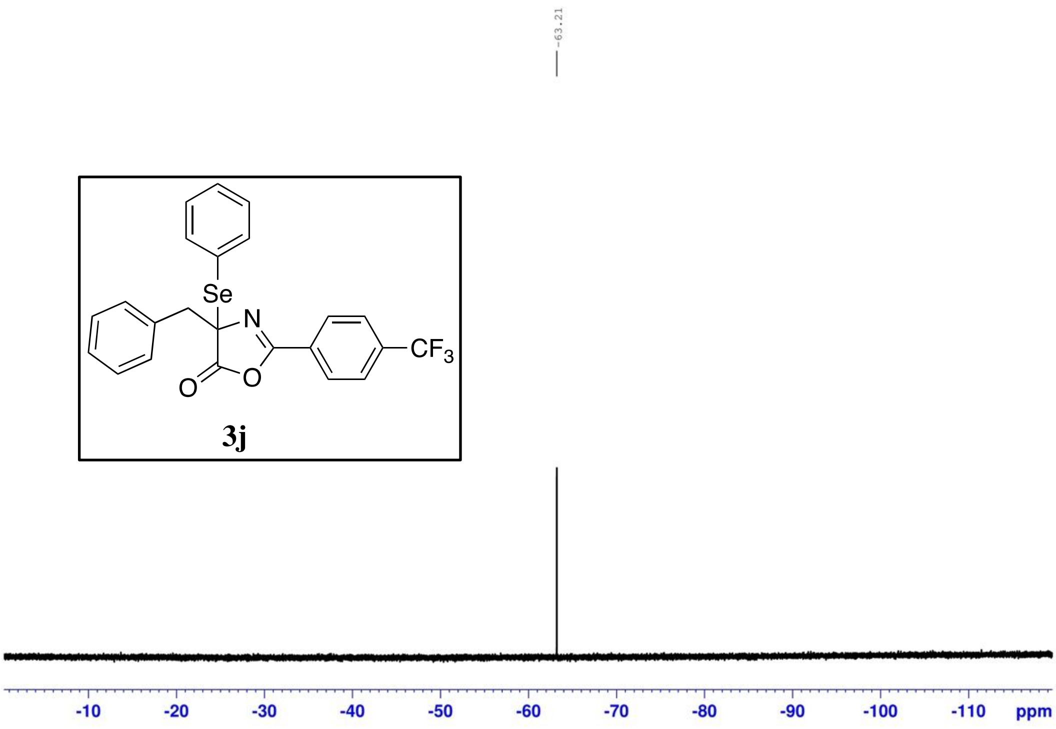

NMR spectra of compound **3k**

[1]**H-NMR** (300 MHz, $CDCl_3$, 298 K)

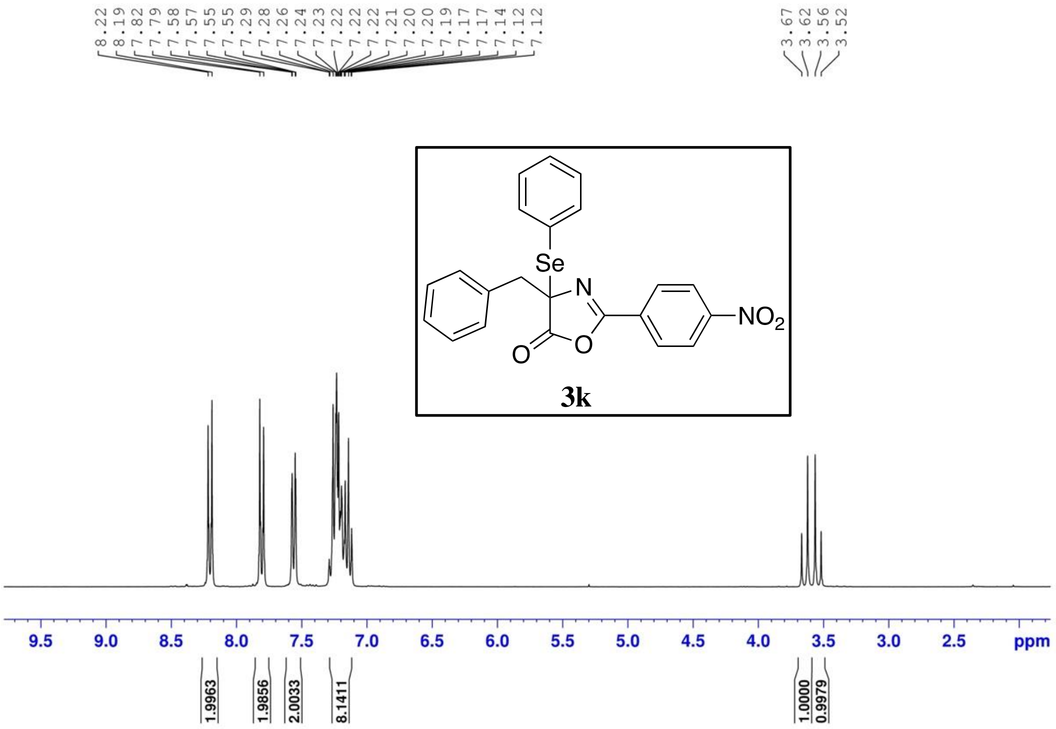

[13]**C-NMR** (75 MHz, $CDCl_3$, 298 K)

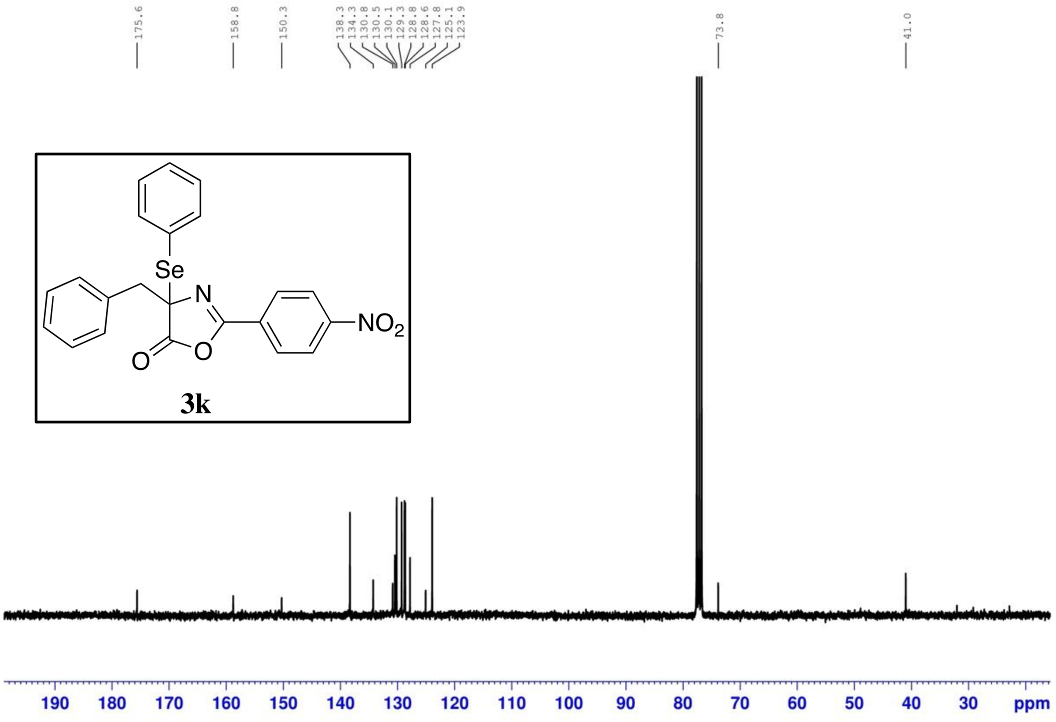

NMR spectra of compound **3l**

[1]**H-NMR** (300 MHz, $CDCl_3$, 298 K)

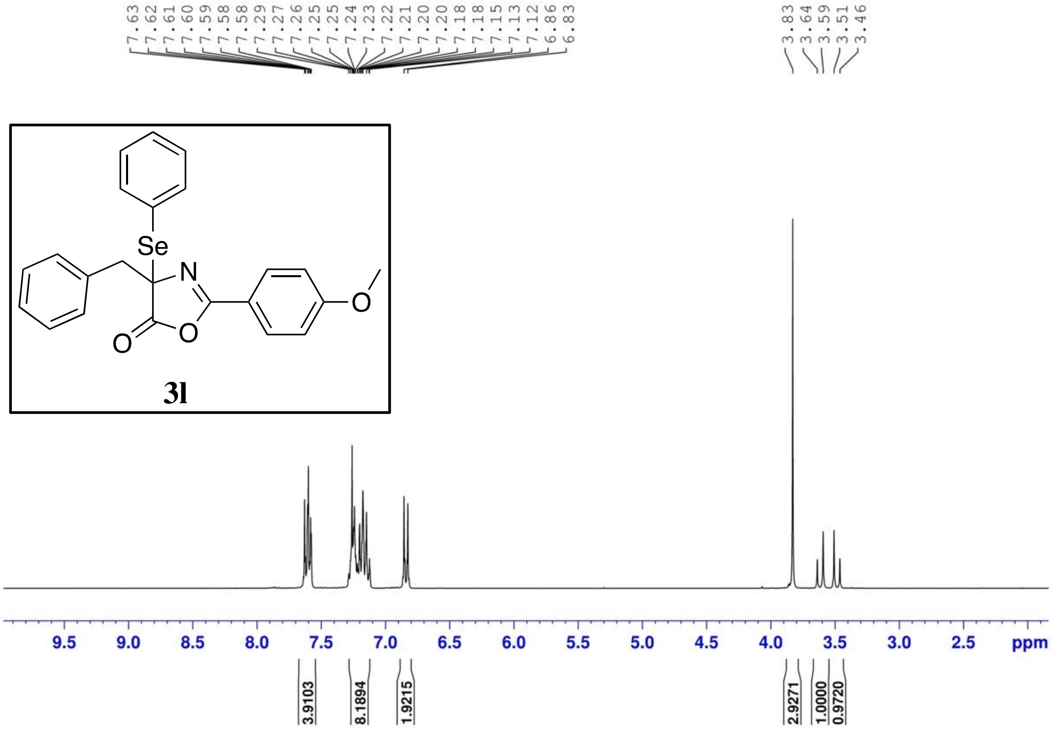

[13]**C-NMR** (75 MHz, $CDCl_3$, 298 K)

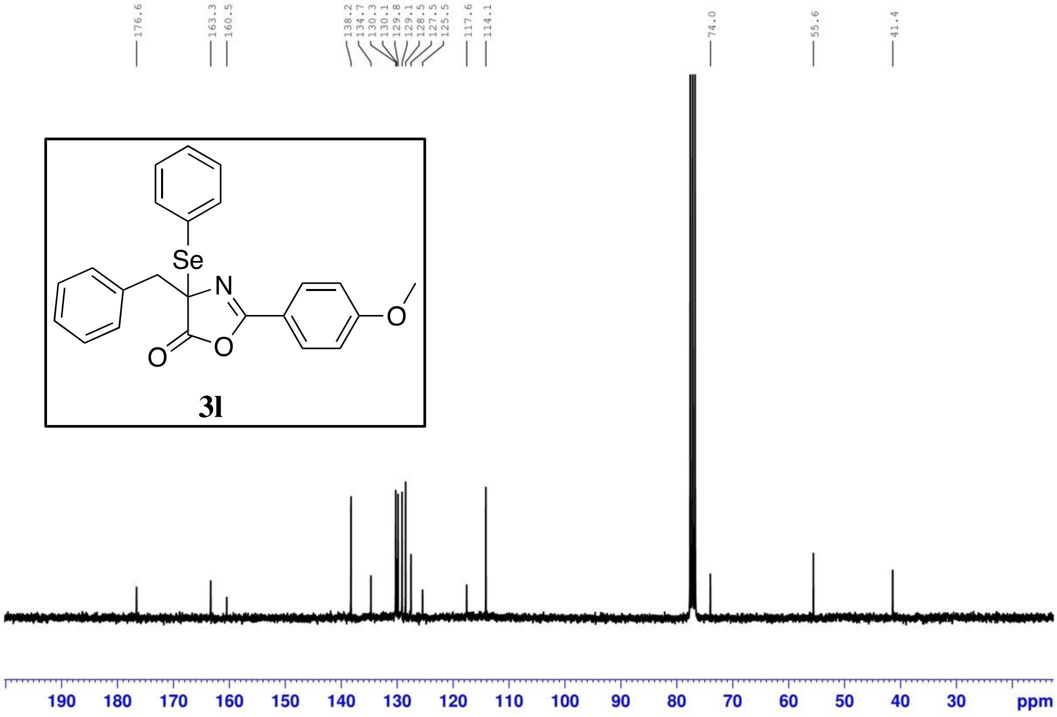

NMR spectra of compound **3m**

^{1}**H-NMR** (300 MHz, $CDCl_3$, 298 K)

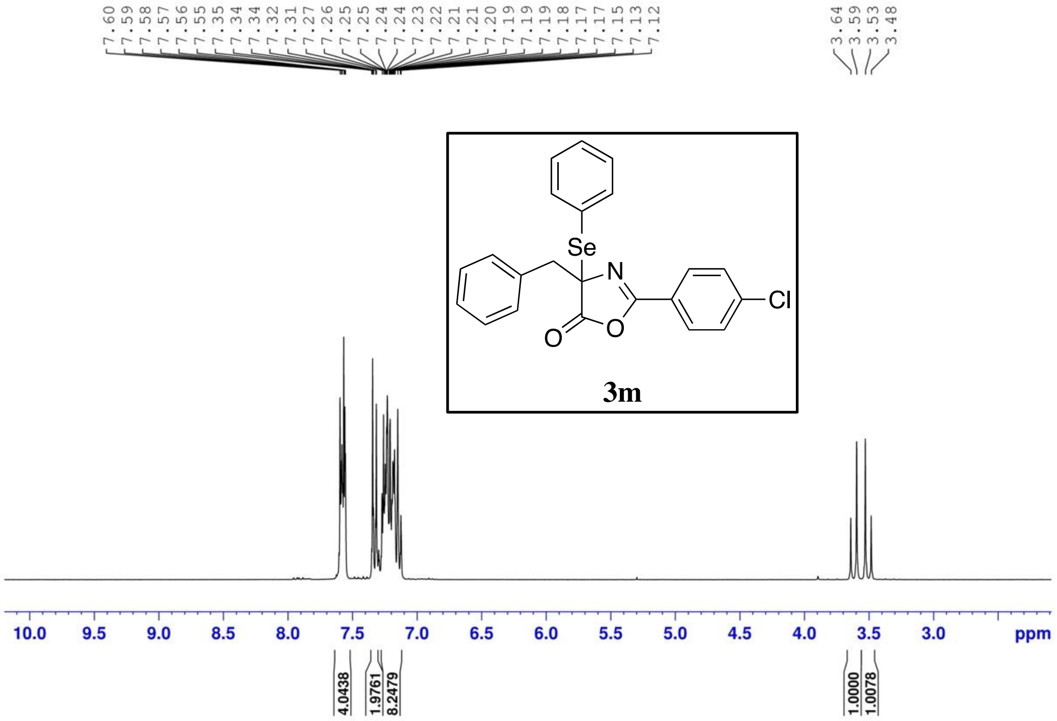

^{13}**C-NMR** (75 MHz, $CDCl_3$, 298 K)

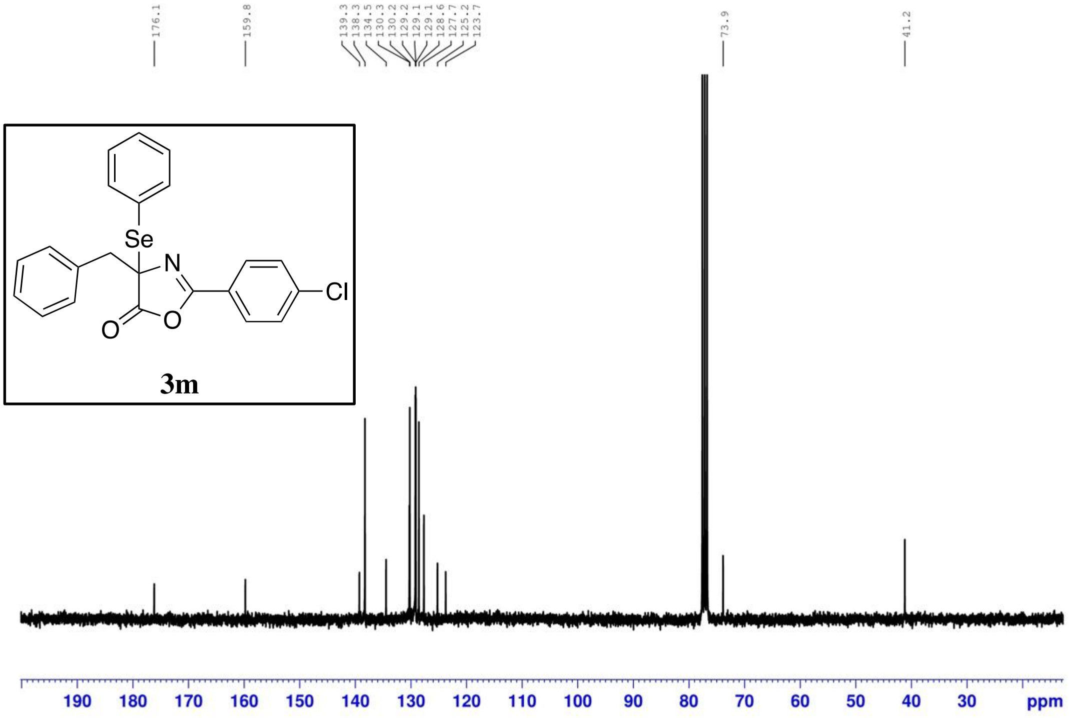

NMR spectra of compound **3n**

^{1}H-NMR (300 MHz, $CDCl_3$, 298 K)

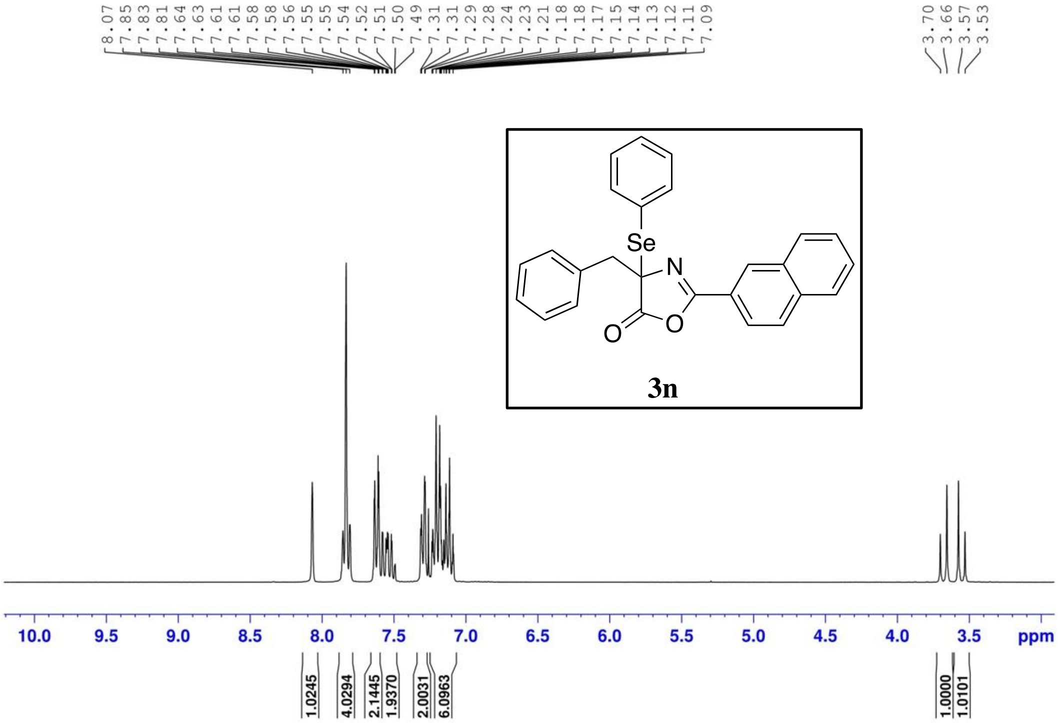

^{13}C-NMR (75 MHz, $CDCl_3$, 298 K)

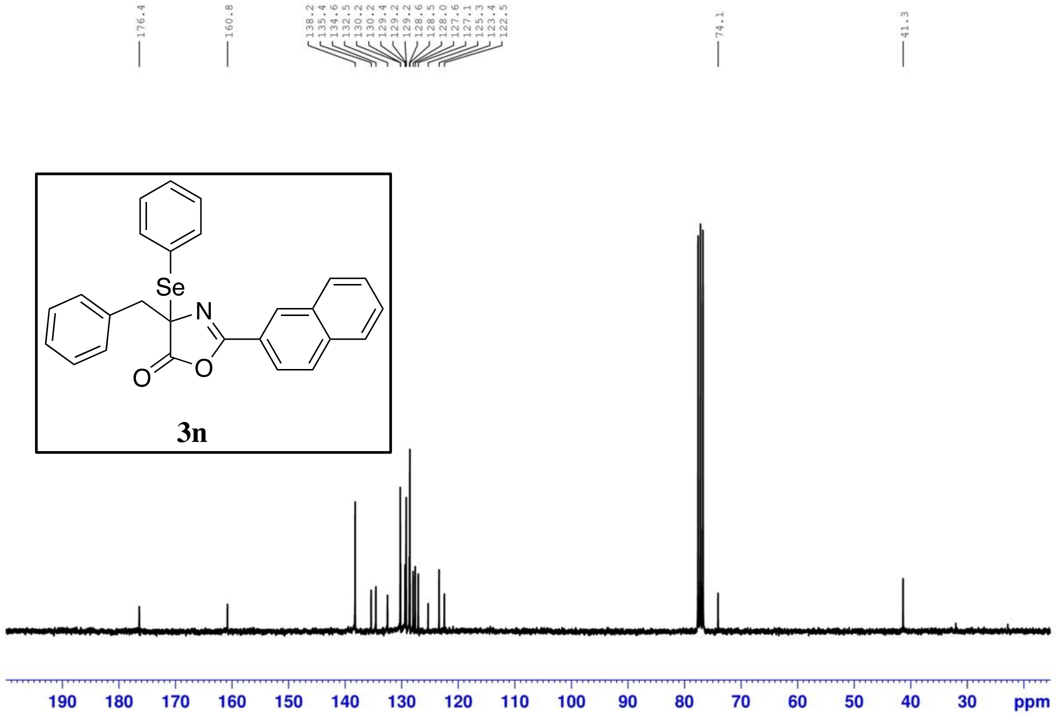

NMR spectra of compound **3o**

^{1}H-NMR (500 MHz, $CDCl_3$, 298 K)

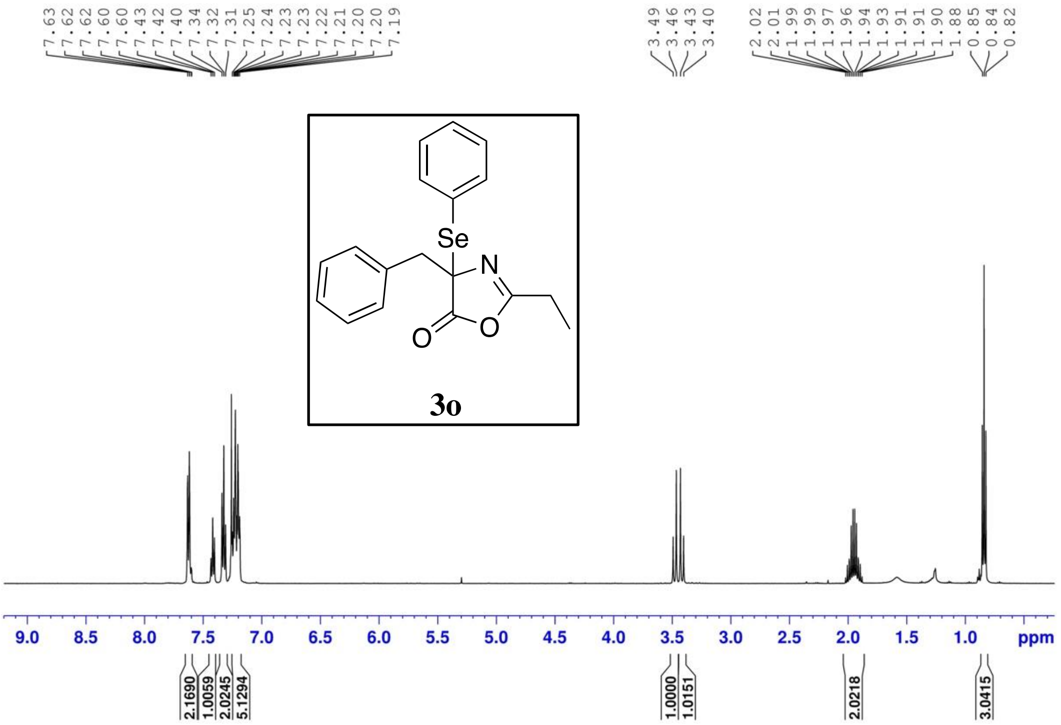

^{13}C-NMR (126 MHz, $CDCl_3$, 298 K)

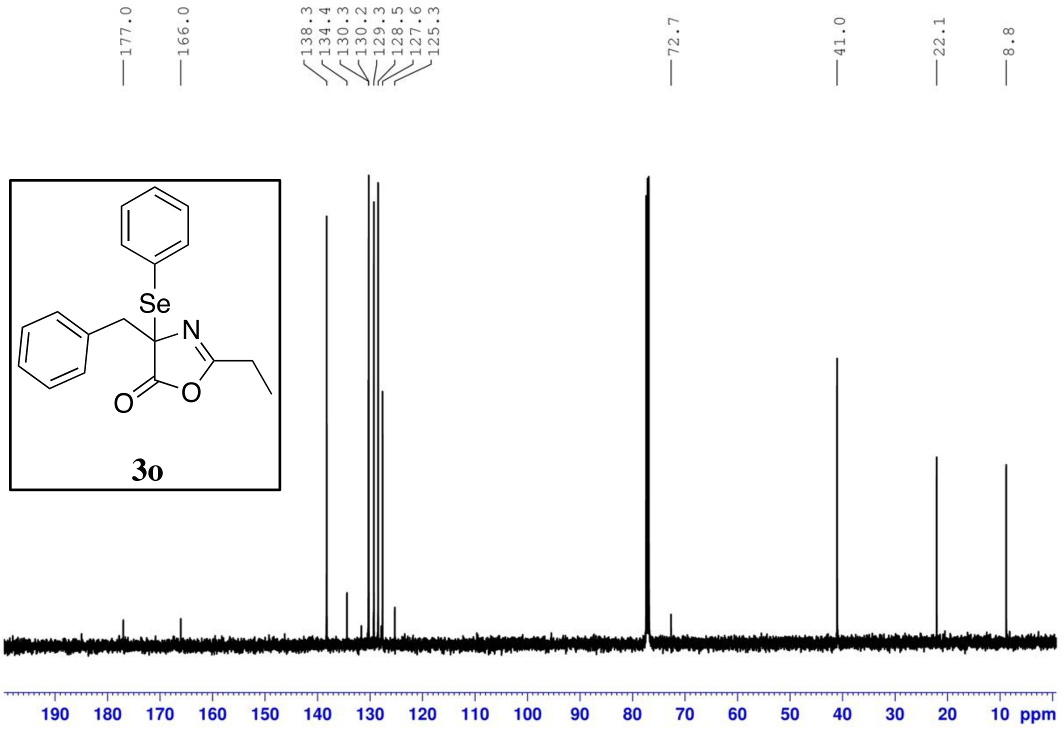

NMR spectra of compound **3p**

^{1}H-NMR (500 MHz, $CDCl_3$, 298 K)

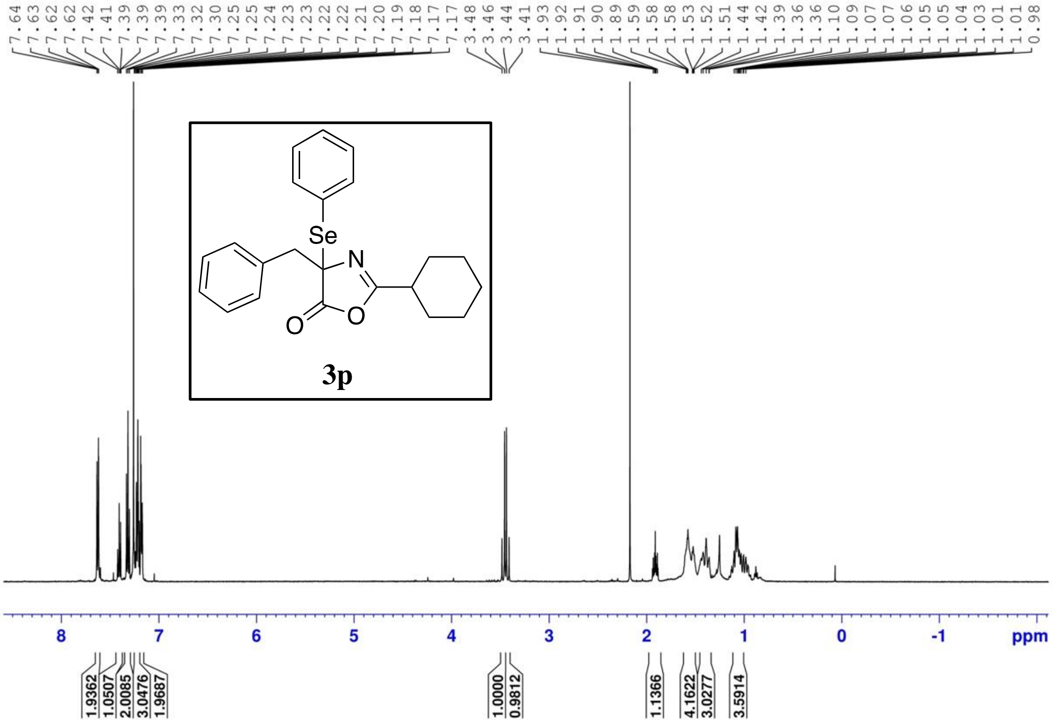

NMR spectra of compound **3q**

^{1}H-NMR (300 MHz, $CDCl_3$, 298 K)

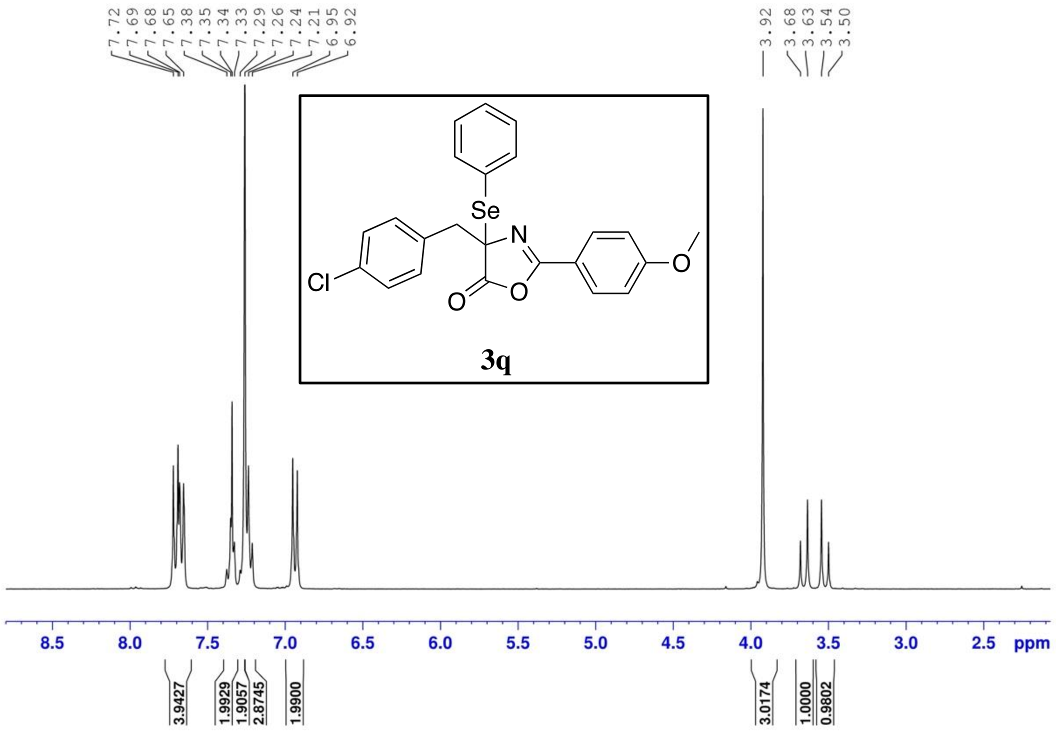

^{13}C-NMR (75 MHz, $CDCl_3$, 298 K)

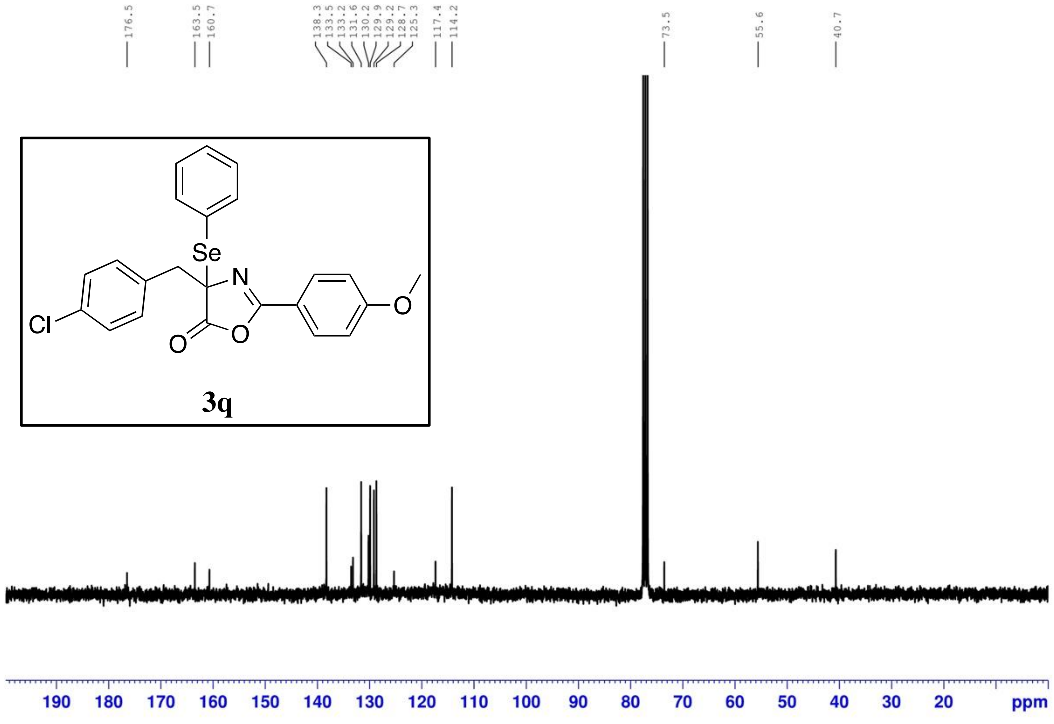

NMR spectra of compound **3r**

^{1}H-NMR (300 MHz, $CDCl_3$, 298 K)

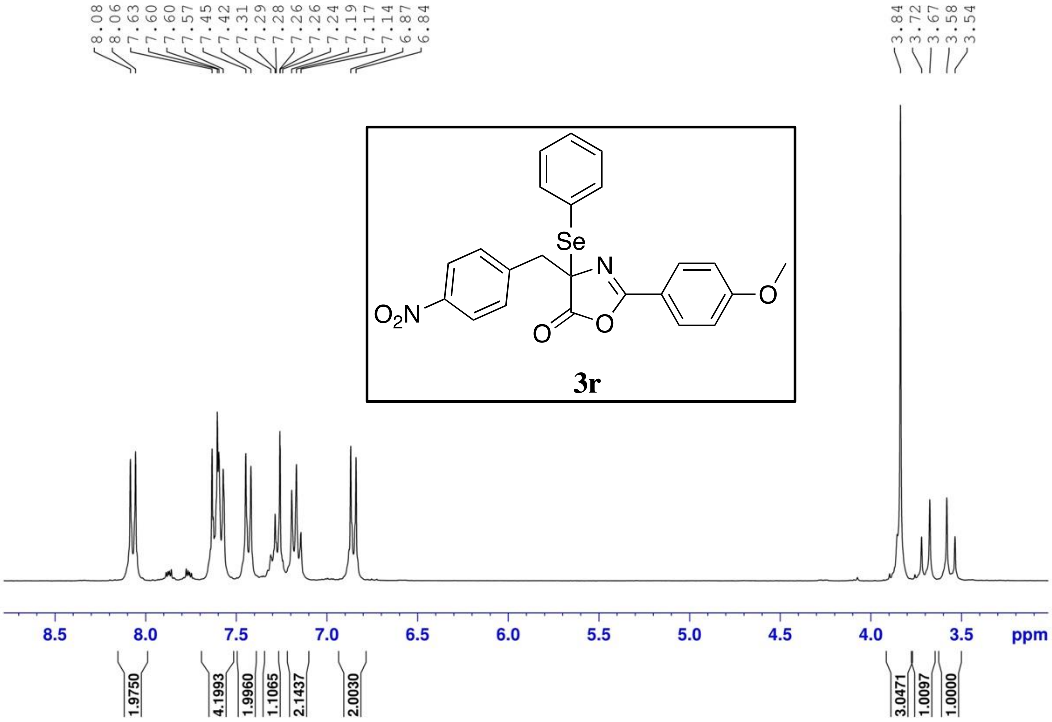

^{13}C-NMR (75 MHz, $CDCl_3$, 298 K)

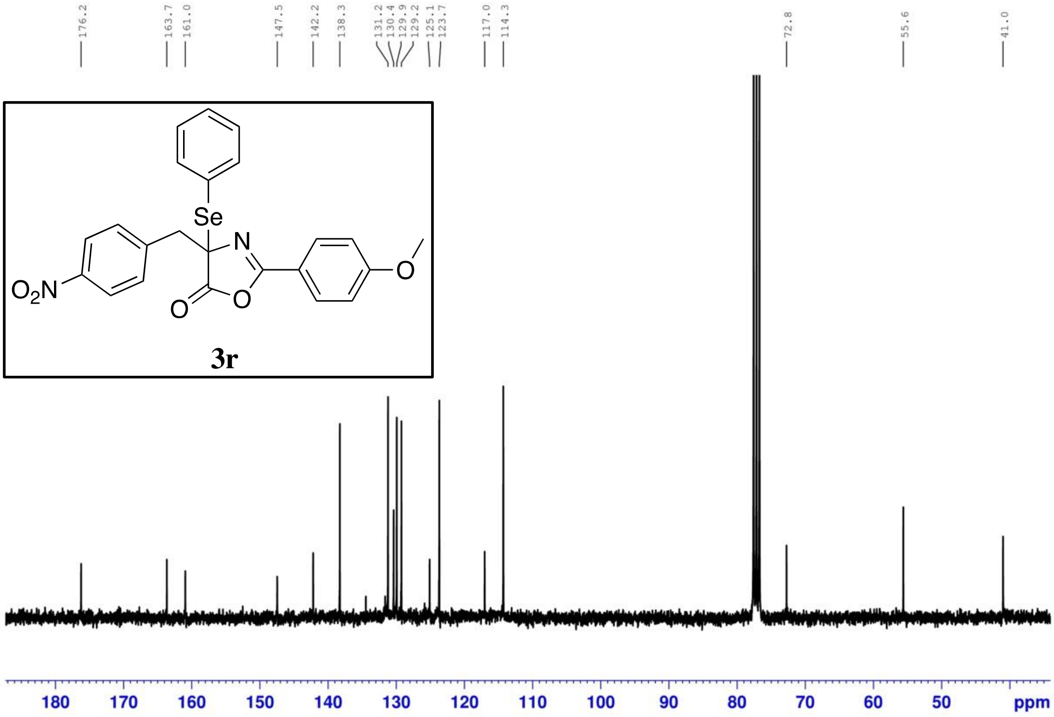

NMR spectra of compound **3s**

¹H-NMR (300 MHz, $CDCl_3$, 298 K)

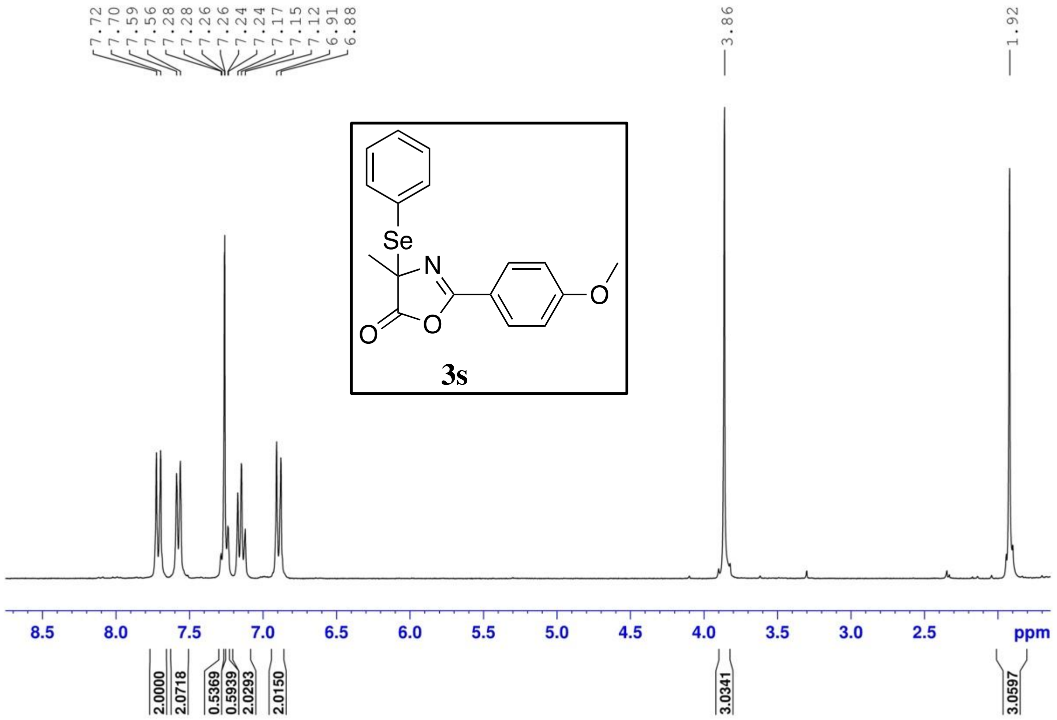

NMR spectra of compound **3t**

^{1}H-NMR (300 MHz, $CDCl_3$, 298 K)

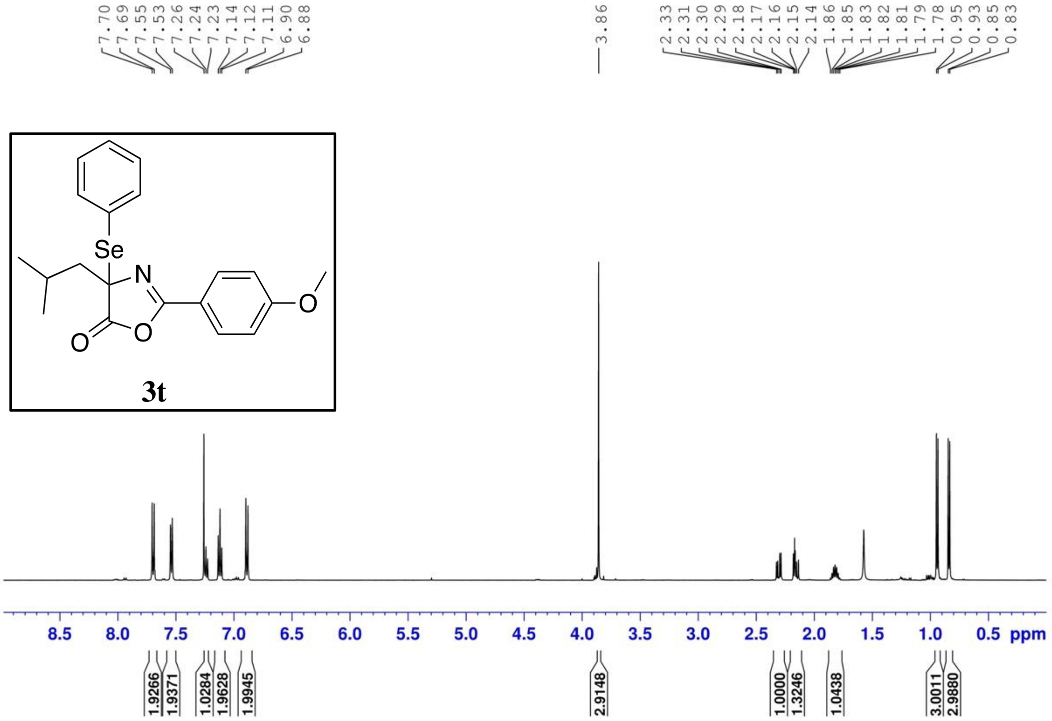

^{13}C-NMR (126 MHz, $CDCl_3$, 298 K)

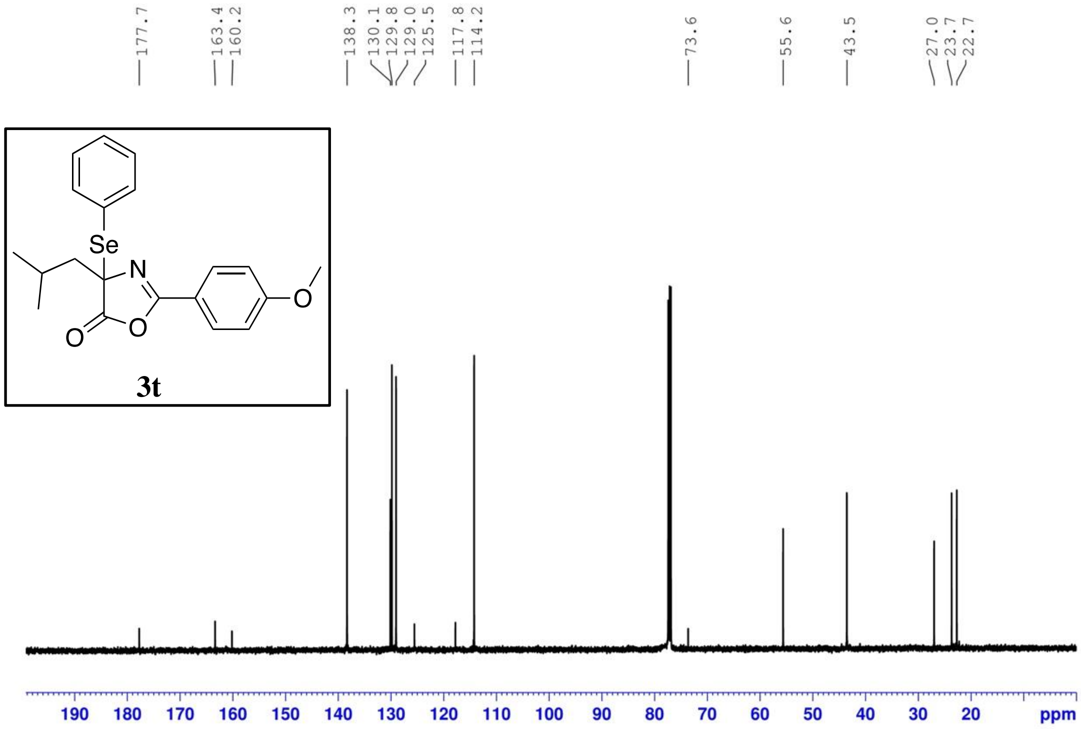

NMR spectra of compound **3u**

^{1}**H-NMR** (300 MHz, $CDCl_3$, 298 K)

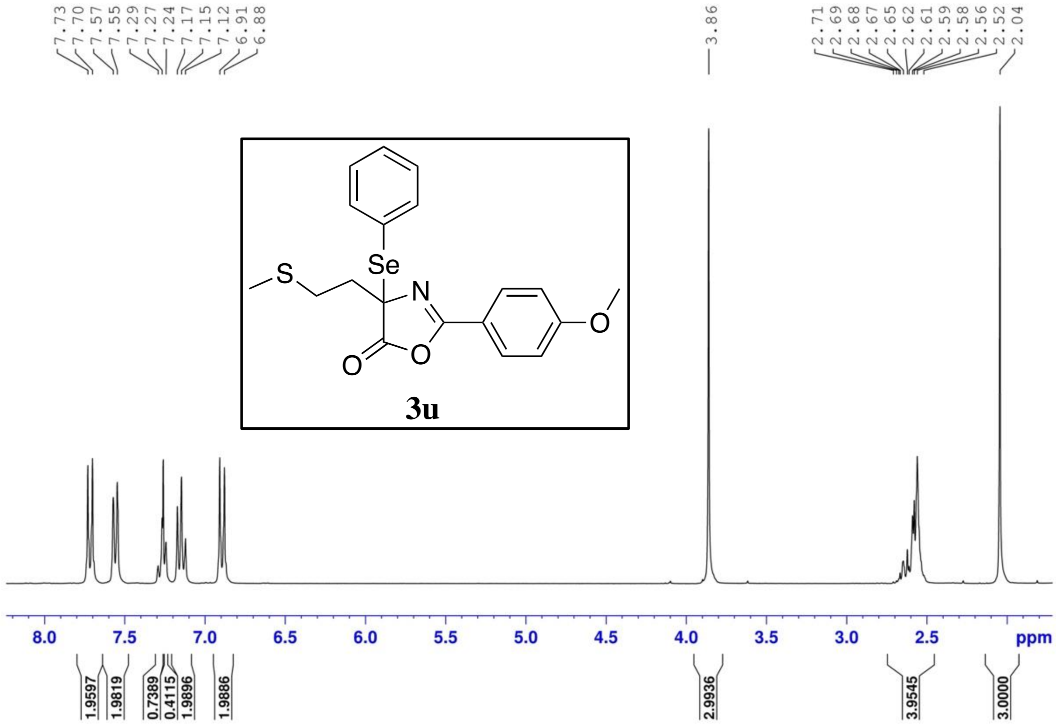

^{13}**C-NMR** (75 MHz, $CDCl_3$, 298 K)

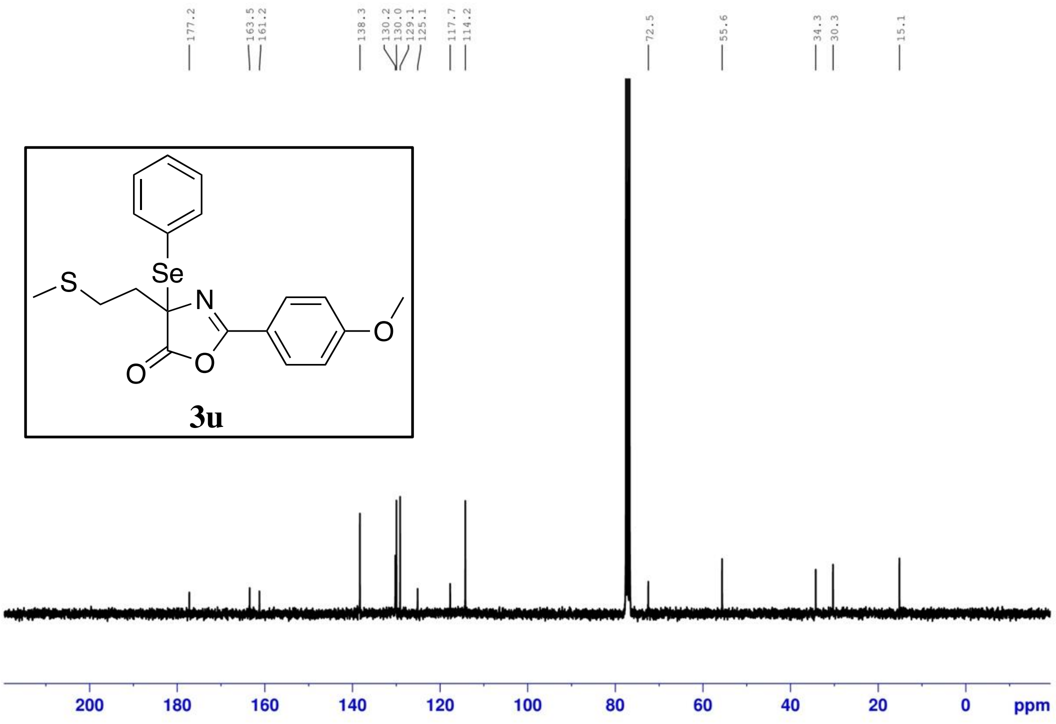

NMR spectra of compound **3v**

^{1}H-NMR (300 MHz, $CDCl_3$, 298 K)

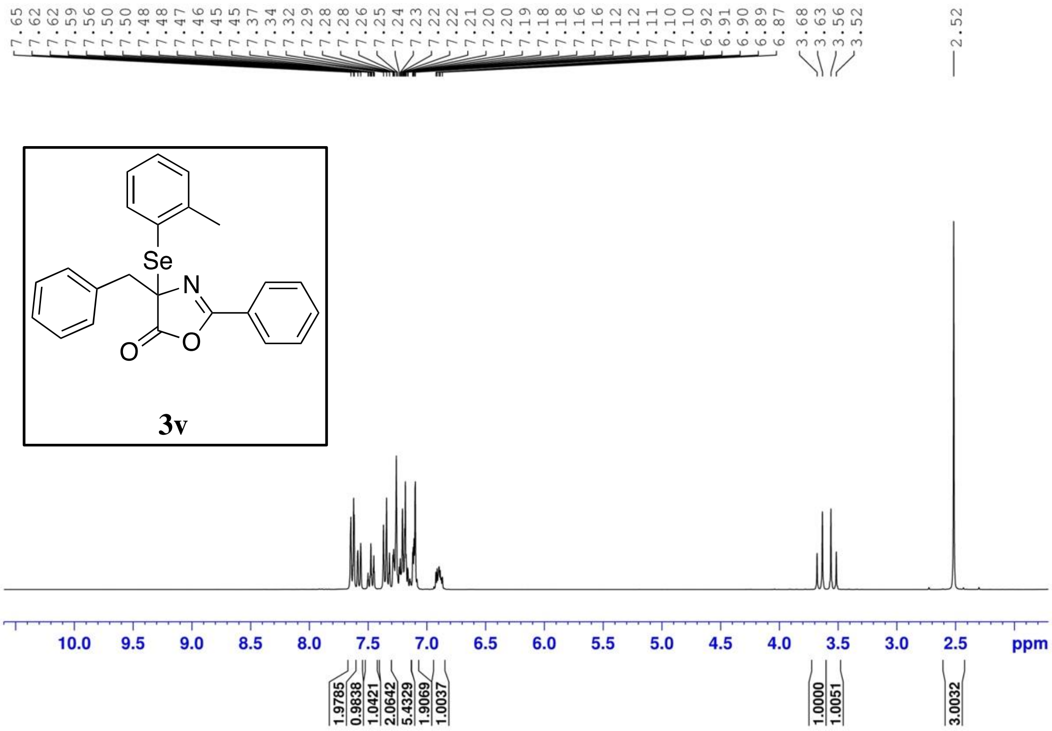

^{13}C-NMR (75 MHz, $CDCl_3$, 298 K)

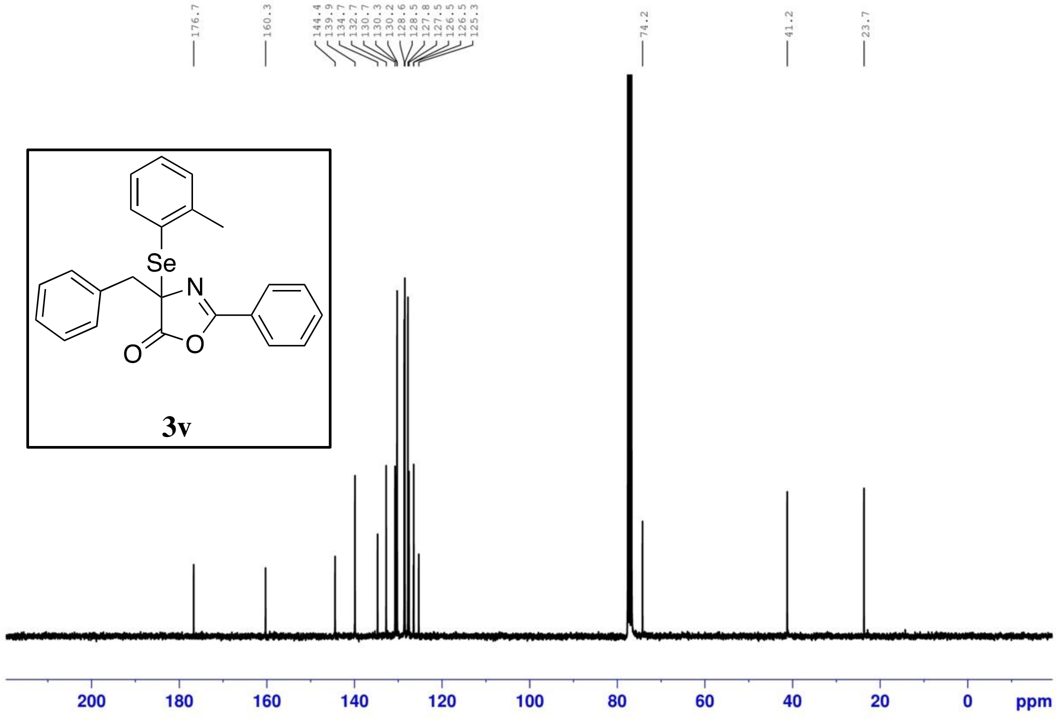

NMR spectra of compound **3w**

^{1}H-NMR (300 MHz, $CDCl_3$, 298 K)

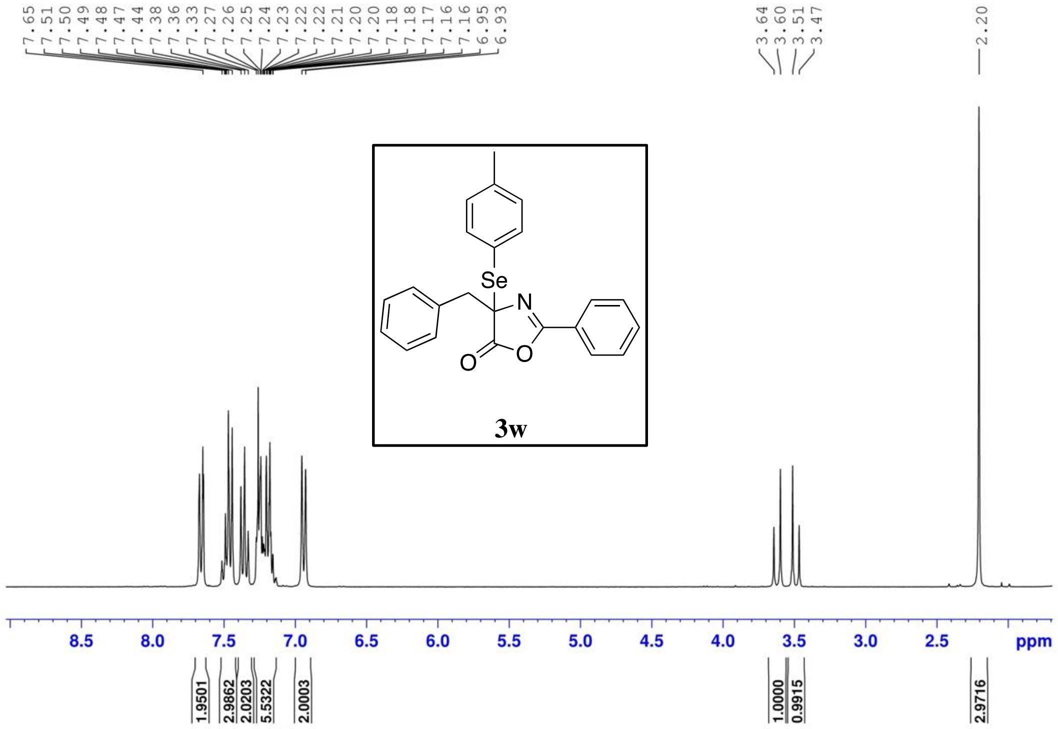

^{13}C-NMR (75 MHz, $CDCl_3$, 298 K)

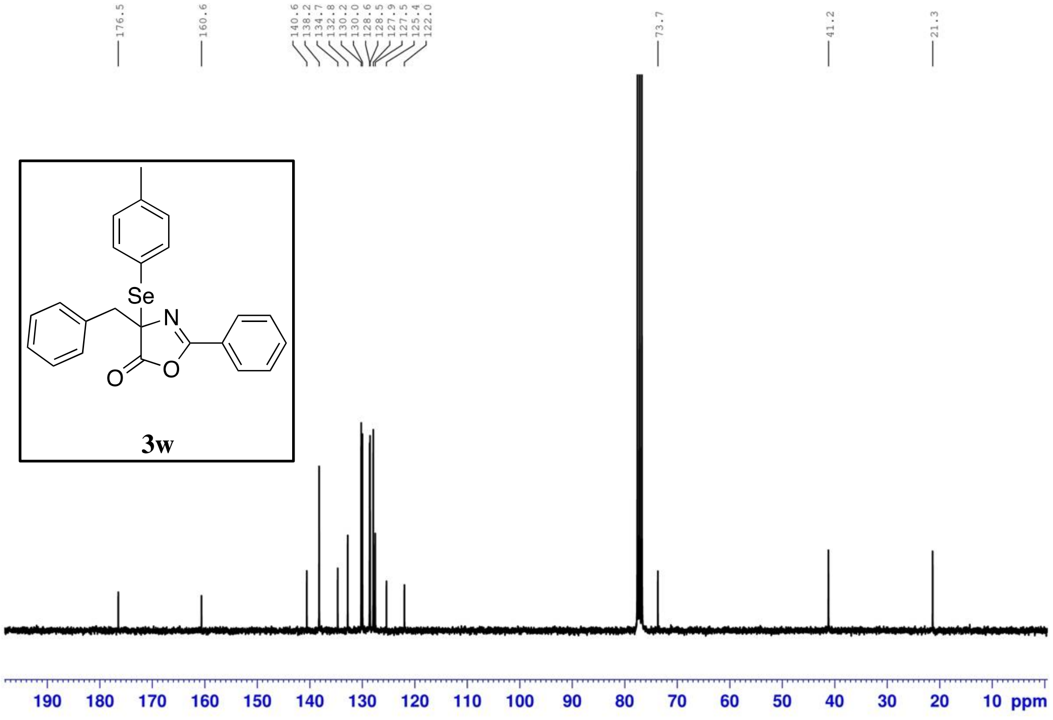

NMR spectra of compound **3x**

^{1}H-NMR (300 MHz, $CDCl_3$, 298 K)

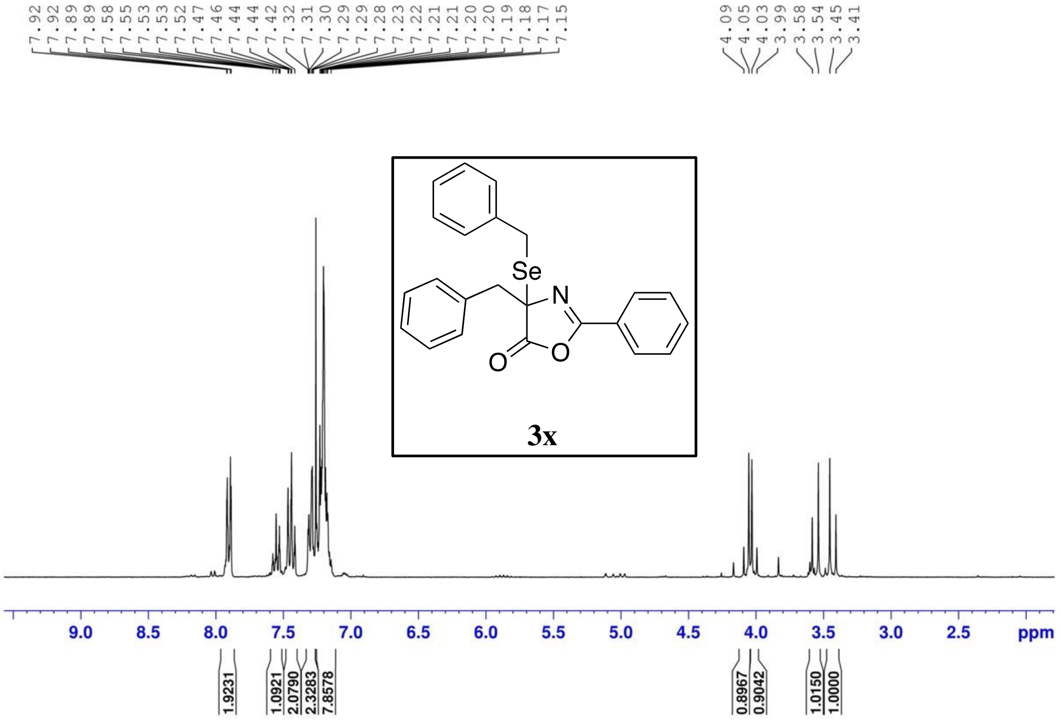

^{13}C-NMR (75 MHz, $CDCl_3$, 298 K)

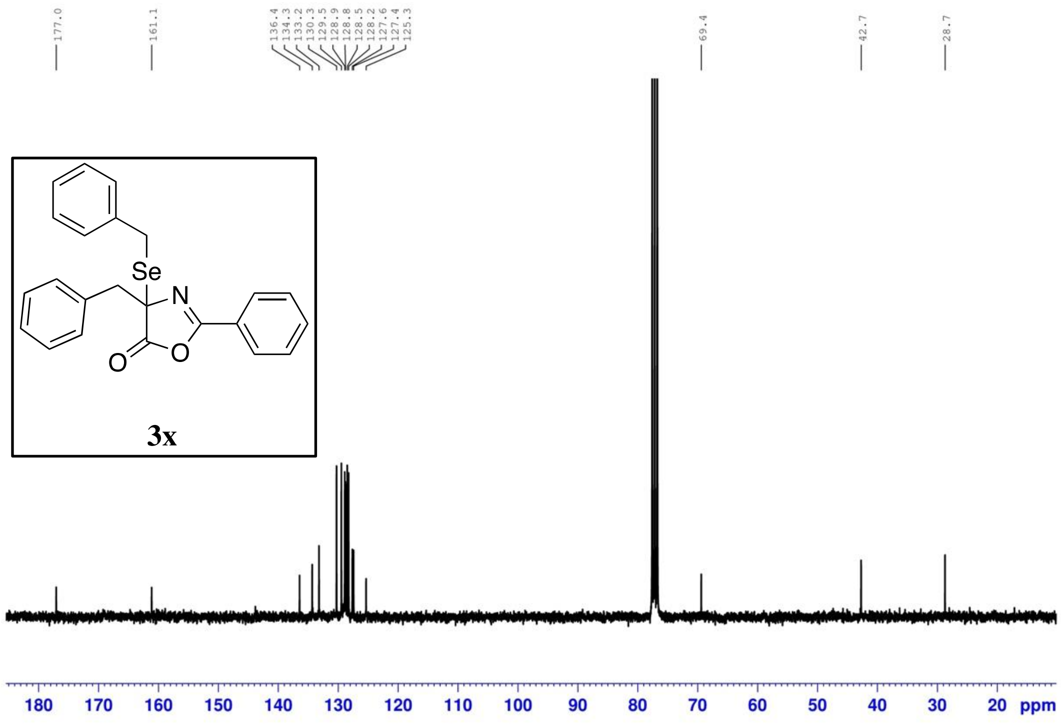

NMR spectra of compound **4a**

¹H-NMR (300 MHz, $CDCl_3$, 298 K)

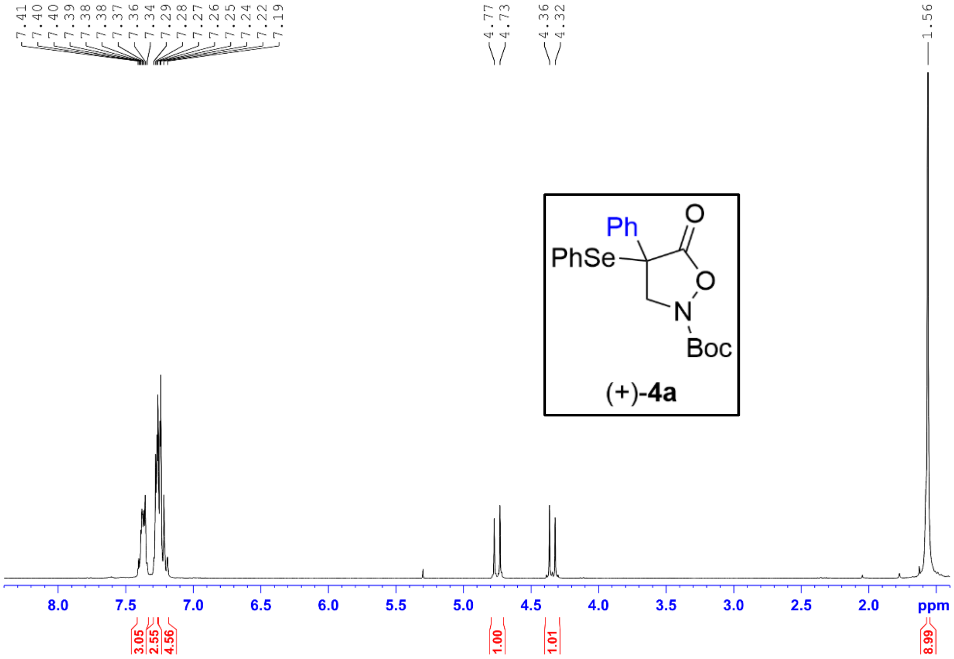

¹³C-NMR (75 MHz, $CDCl_3$, 298 K)

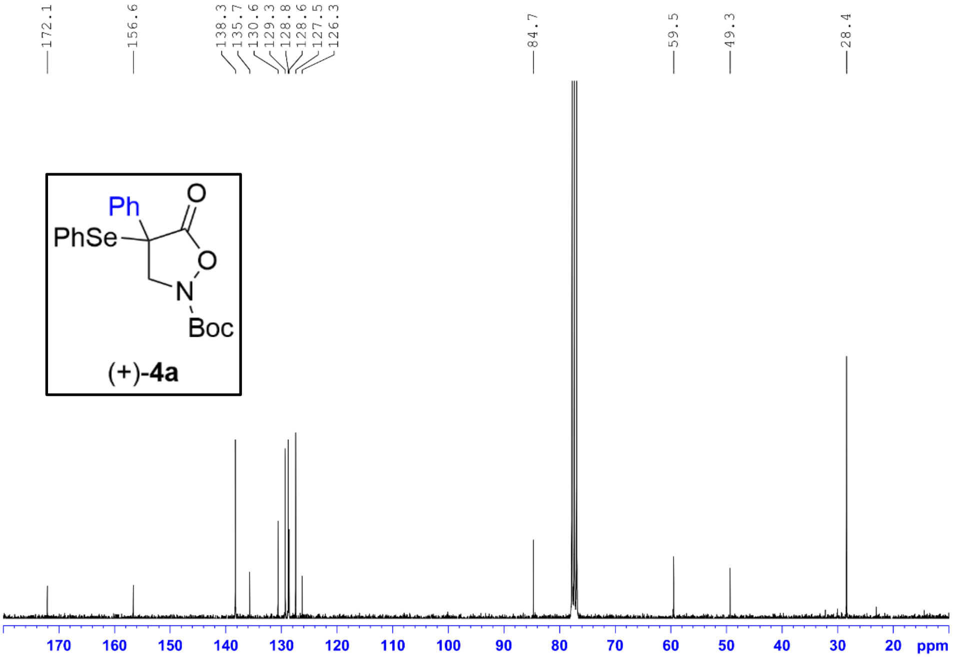

NMR spectra of compound **4b**

^{1}H-NMR (300 MHz, $CDCl_3$, 298 K)

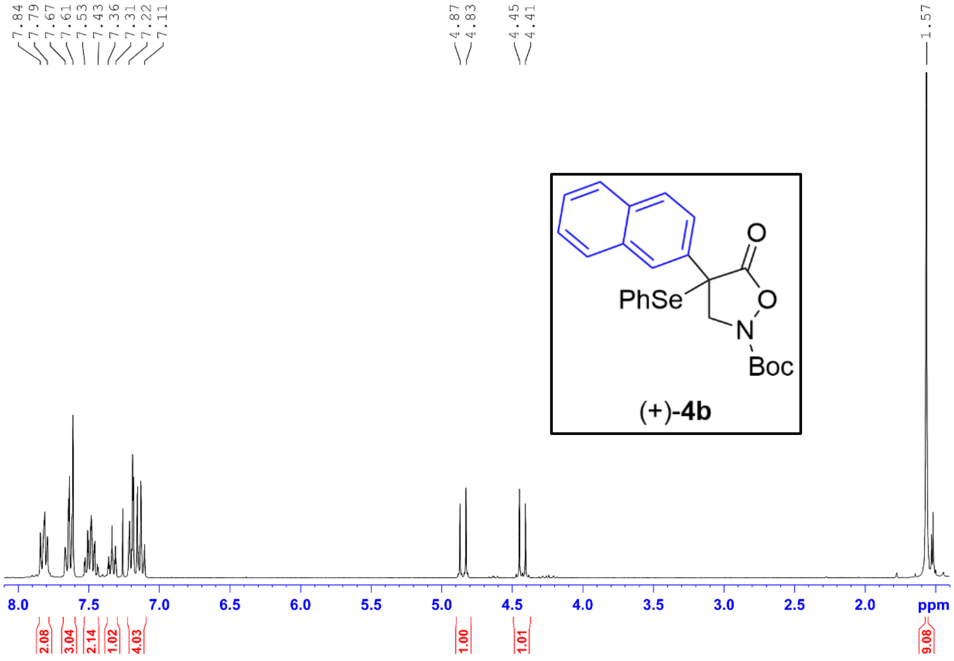

^{13}C-NMR (75 MHz, $CDCl_3$, 298 K)

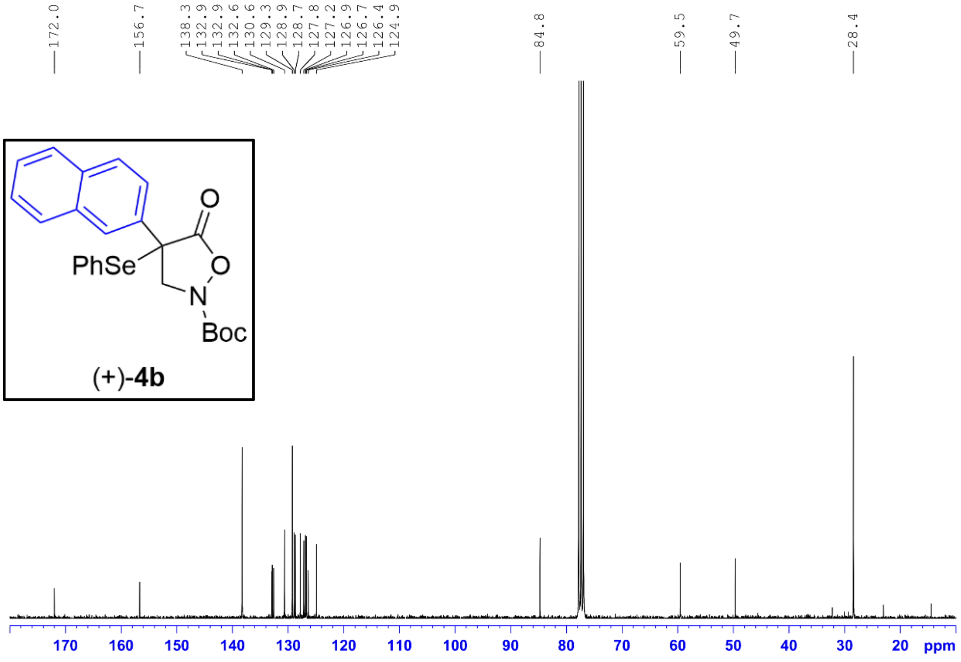

NMR spectra of compound **4c**

^{1}H-NMR (300 MHz, $CDCl_3$, 298 K)

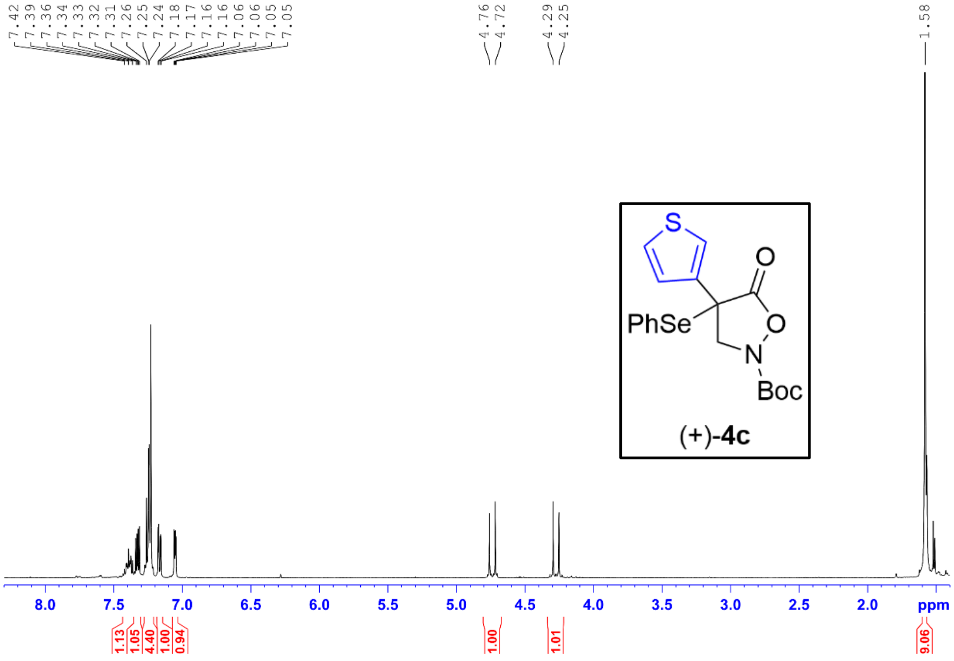

^{13}C-NMR (75 MHz, $CDCl_3$, 298 K)

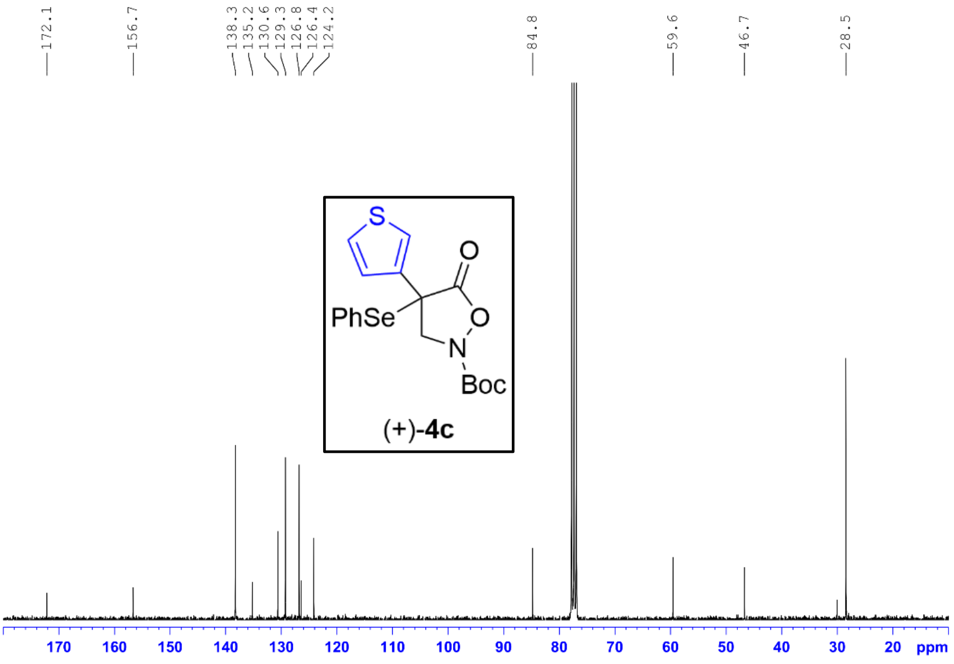

NMR spectra of compound **4d**

^{1}H-NMR (300 MHz, $CDCl_3$, 298 K)

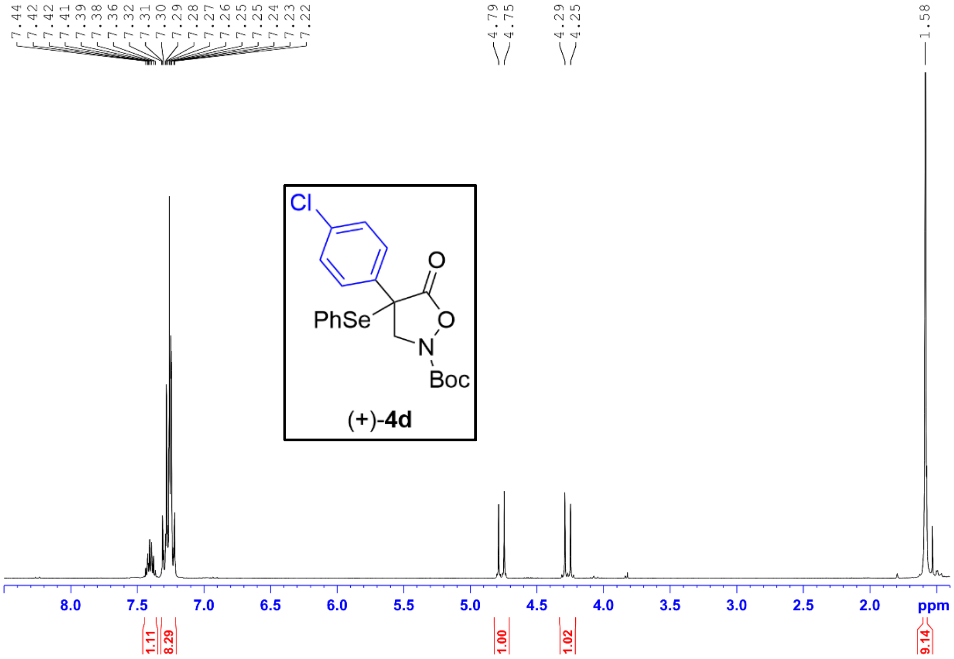

^{13}C-NMR (75 MHz, $CDCl_3$, 298 K)

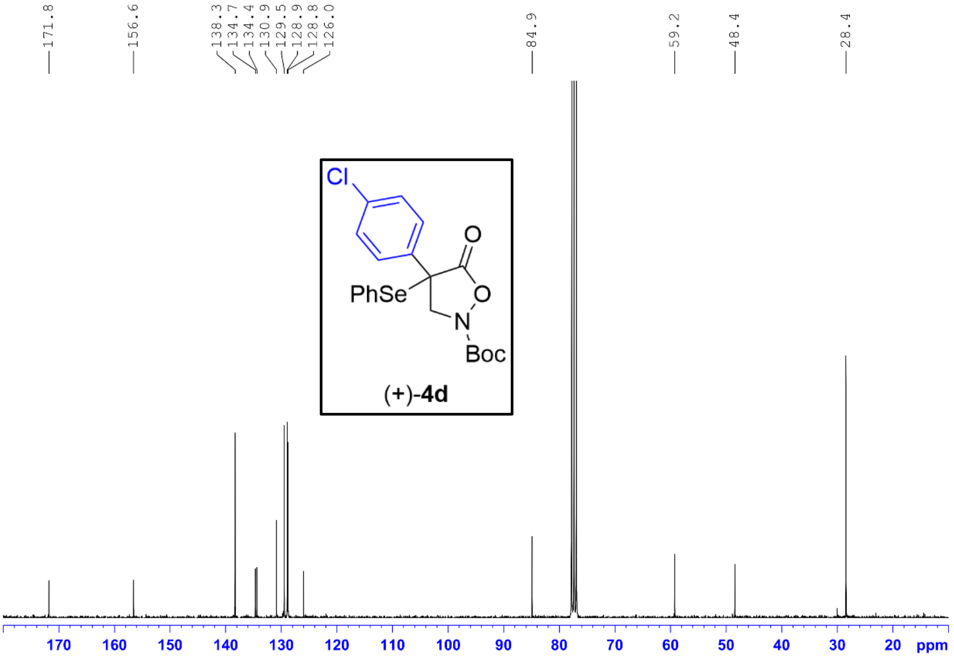

NMR spectra of compound **4e**

^{1}H-NMR (300 MHz, $CDCl_3$, 298 K)

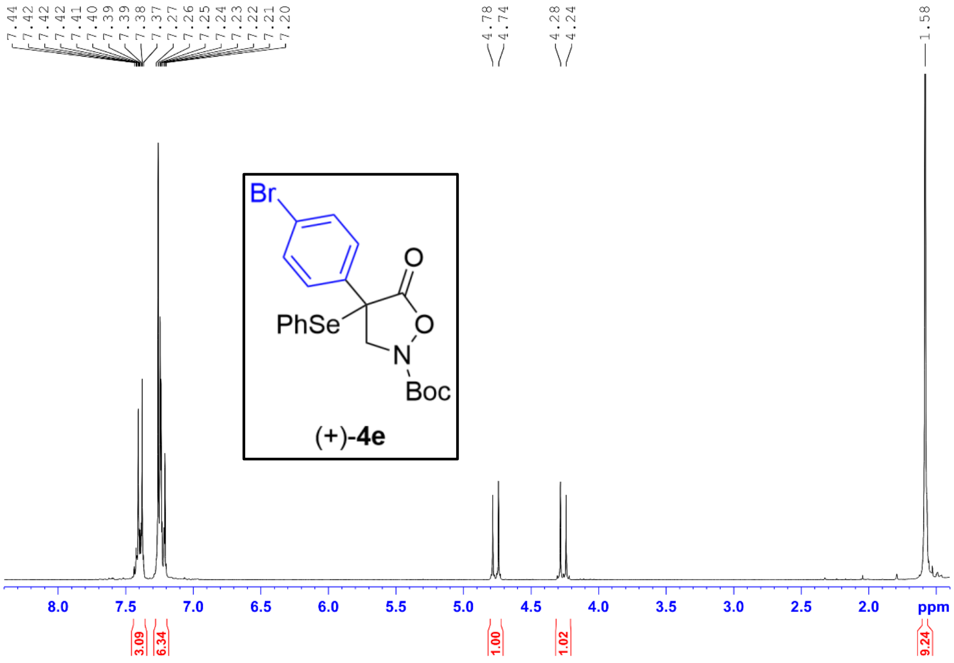

^{13}C-NMR (75 MHz, $CDCl_3$, 298 K)

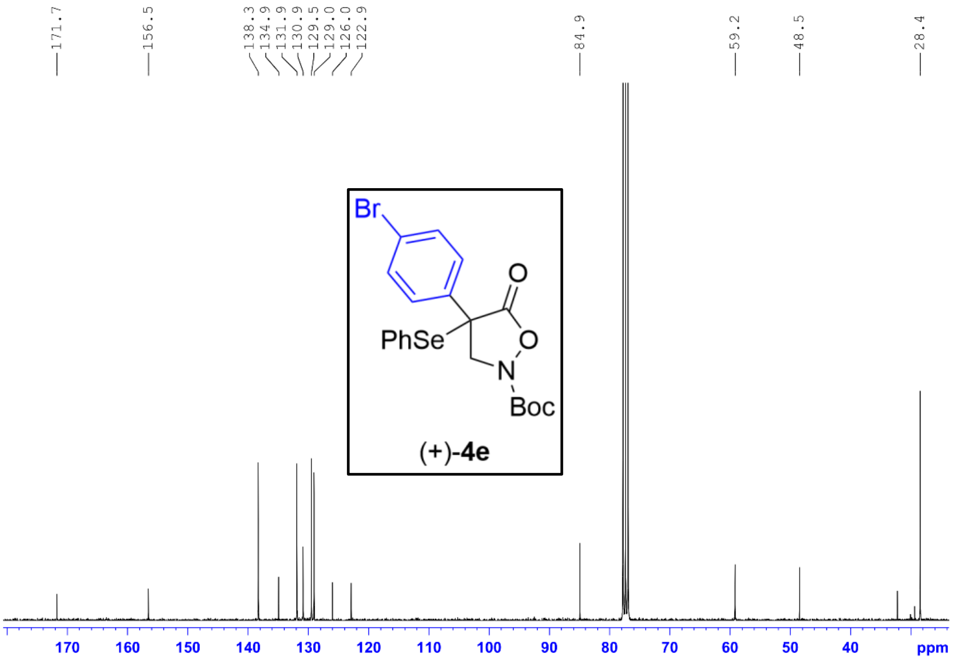

NMR spectra of compound **4f**

^{1}**H-NMR** (300 MHz, $CDCl_3$, 298 K)

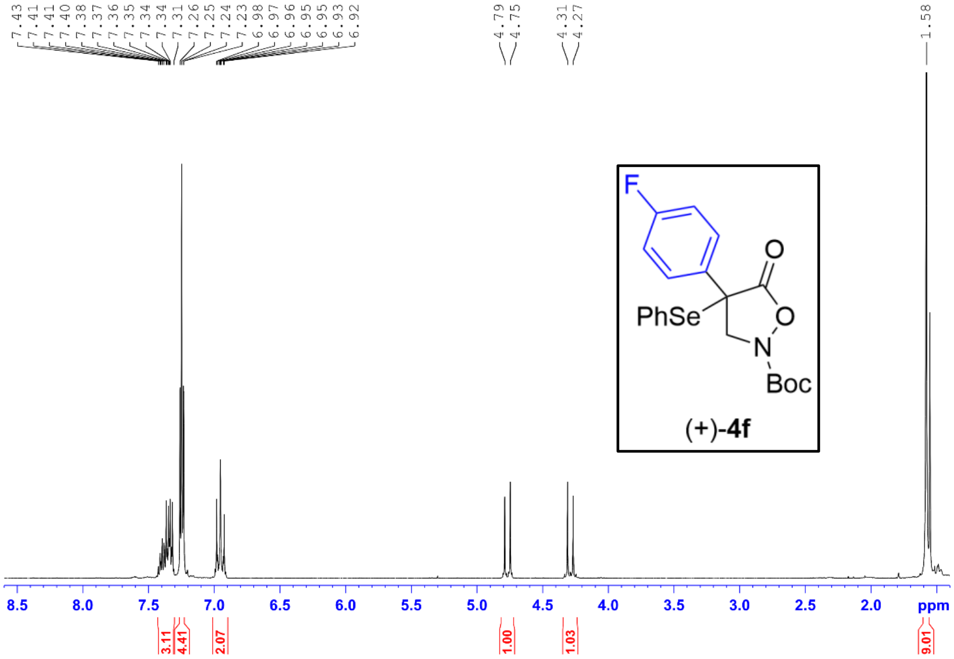

^{13}**C-NMR** (75 MHz, $CDCl_3$, 298 K)

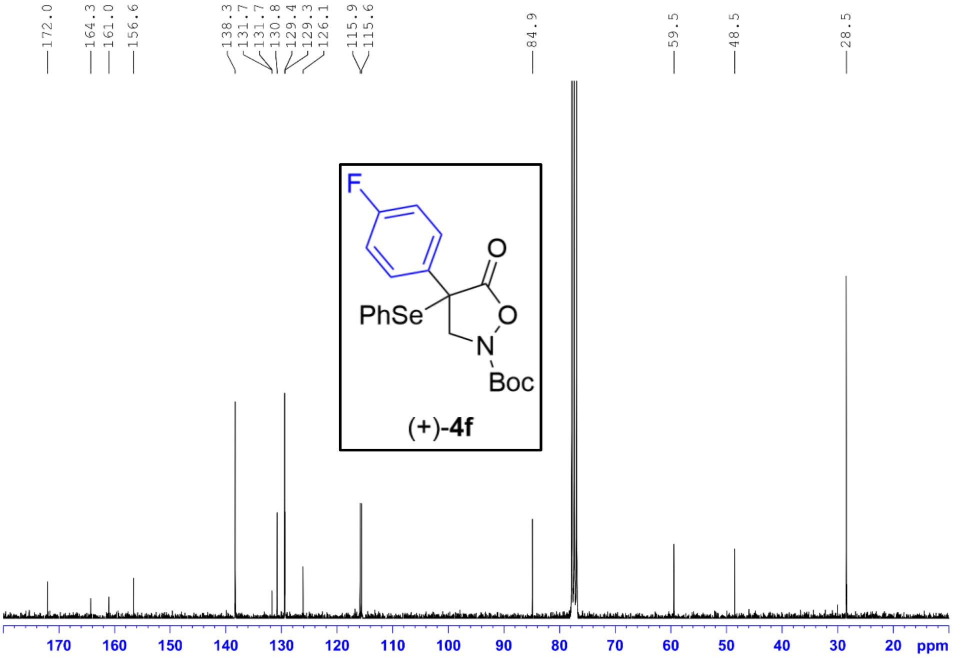

^{19}F-NMR (282 MHz, $CDCl_3$, 298 K)

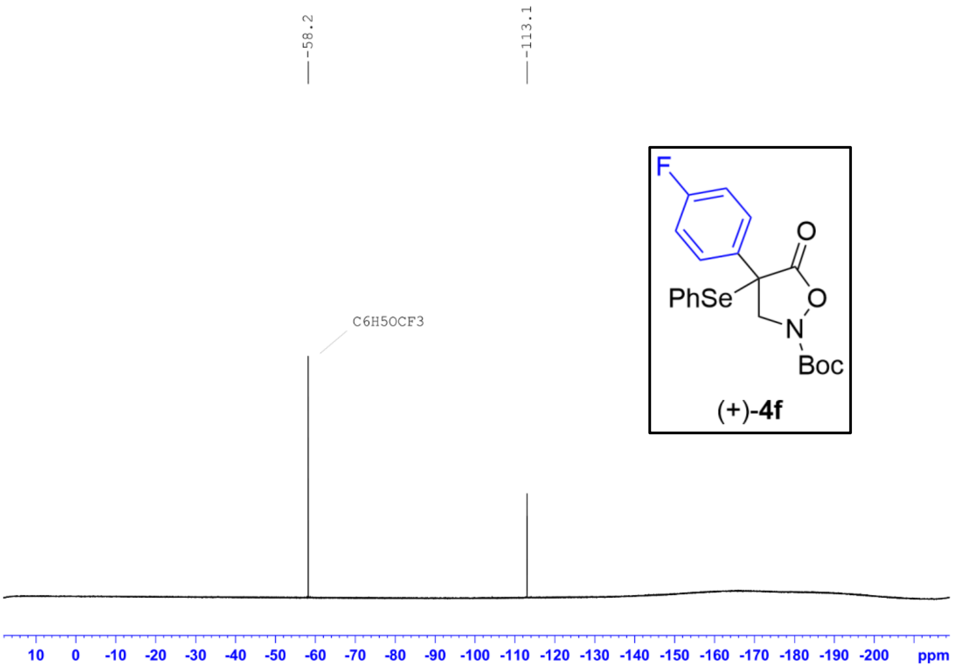

NMR spectra of compound **4g**

^{1}H-NMR (300 MHz, $CDCl_3$, 298 K)

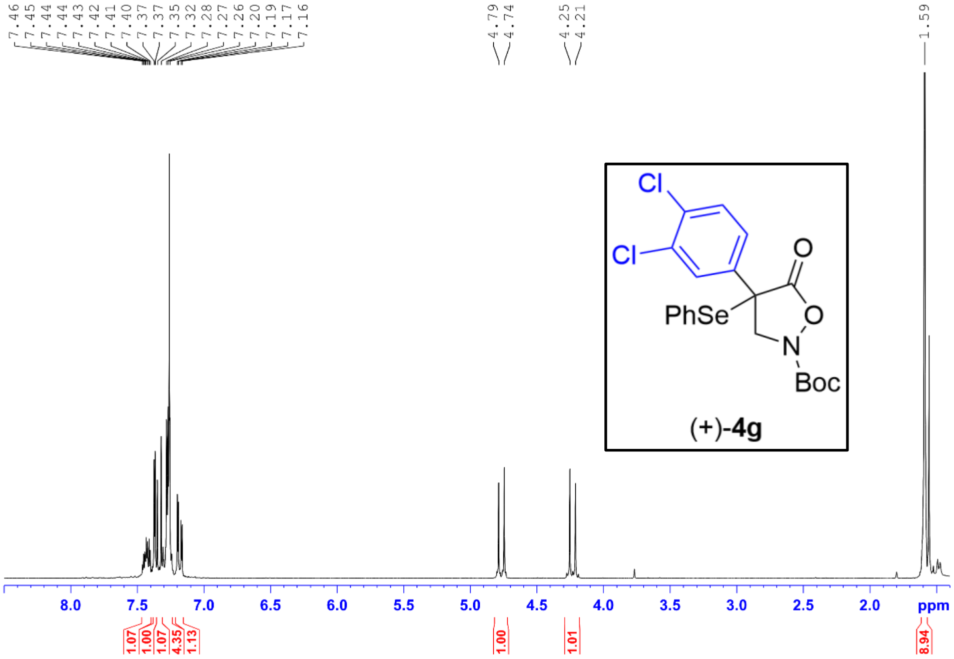

^{13}C-NMR (75 MHz, $CDCl_3$, 298 K)

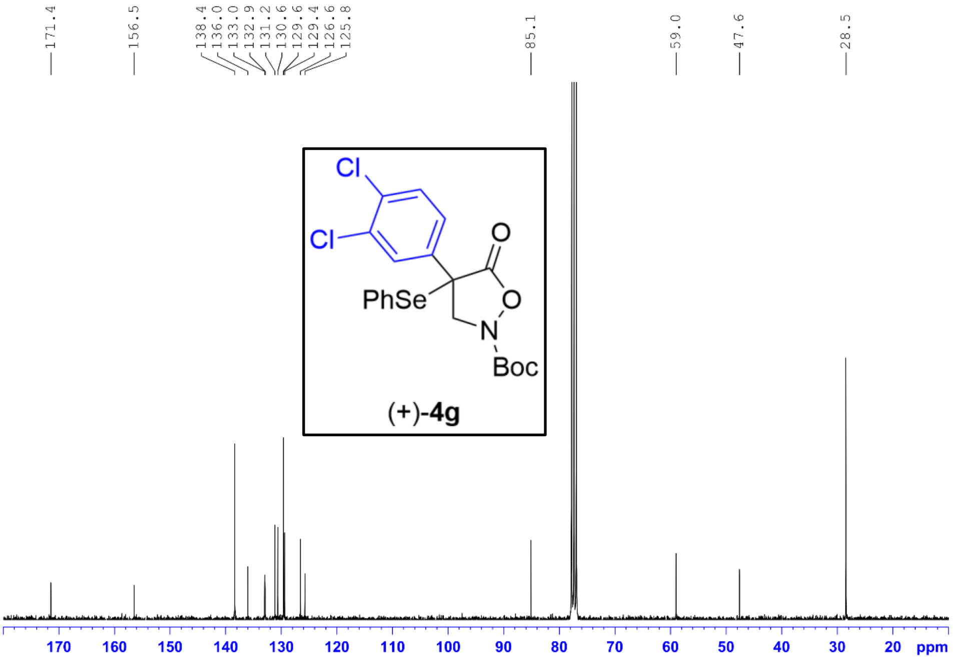

NMR spectra of compound **4i**

^{1}H-NMR (500 MHz, $CDCl_3$, 298 K)

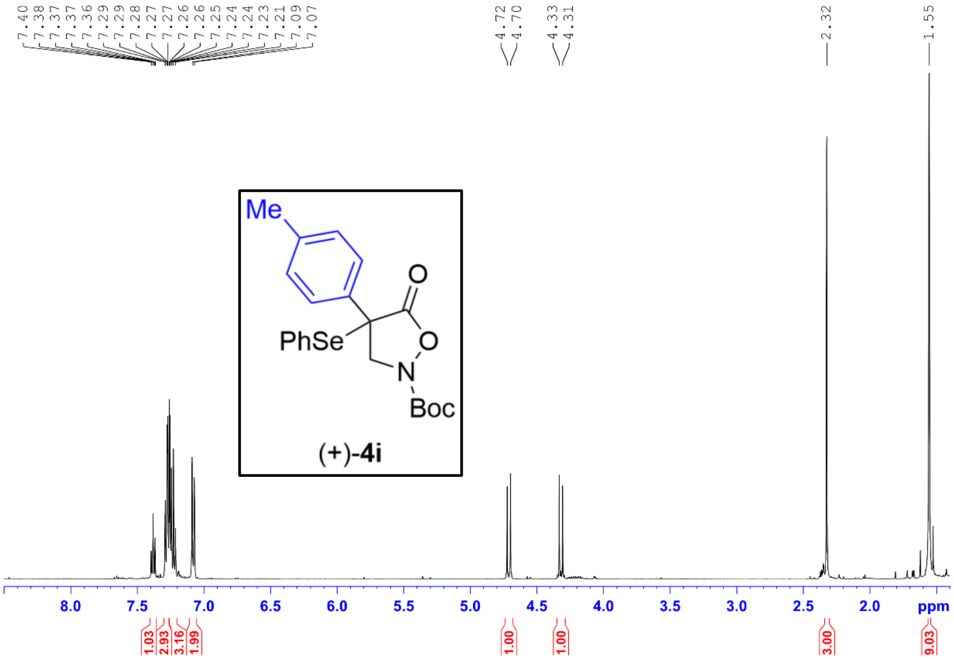

^{13}C-NMR (126 MHz, $CDCl_3$, 298 K)

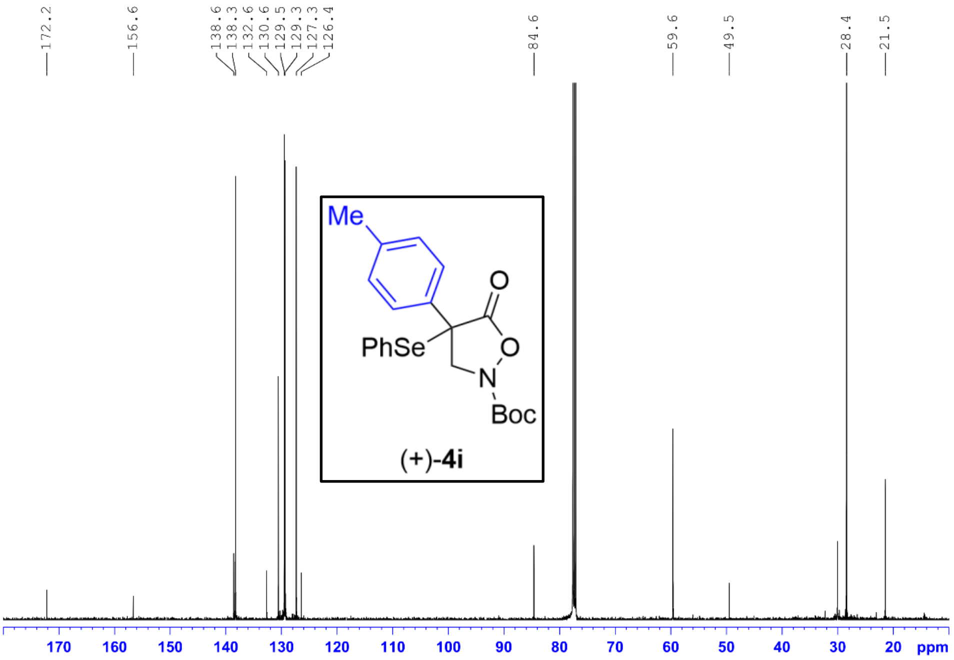

NMR spectra of compound **4j**

^{1}H-NMR (500 MHz, $CDCl_3$, 298 K)

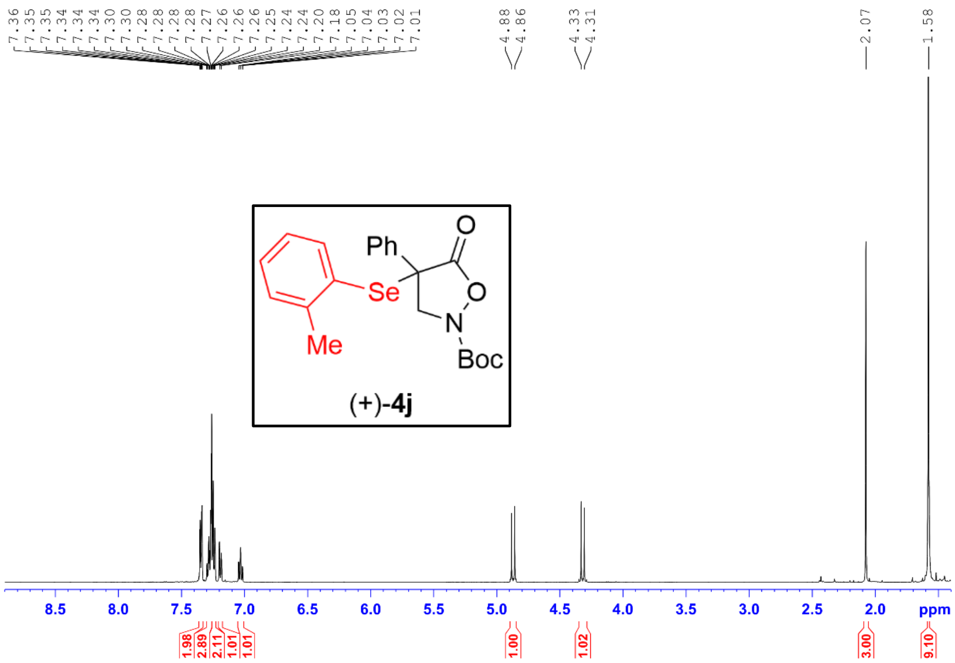

^{13}C-NMR (126 MHz, $CDCl_3$, 298 K)

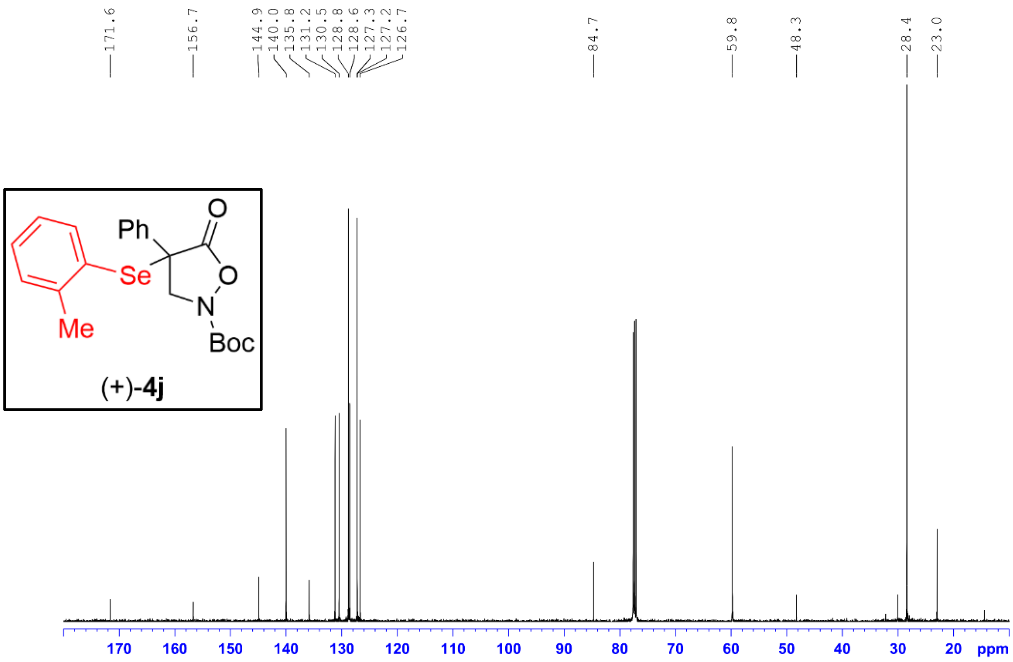

NMR spectra of compound **4k**

^{1}H-NMR (300 MHz, $CDCl_3$, 298 K)

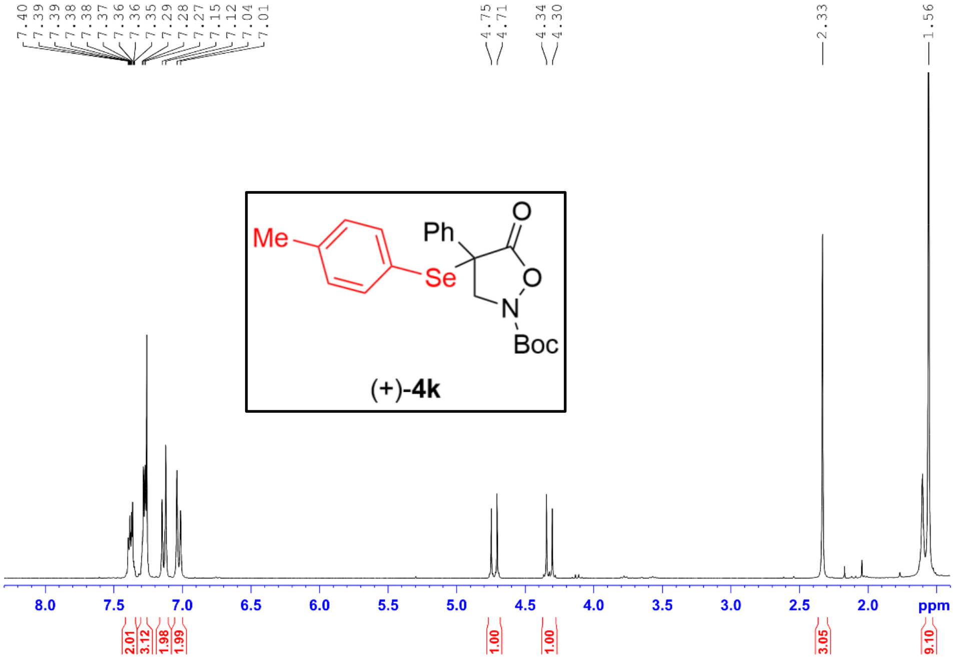

^{13}C-NMR (75 MHz, $CDCl_3$, 298 K)

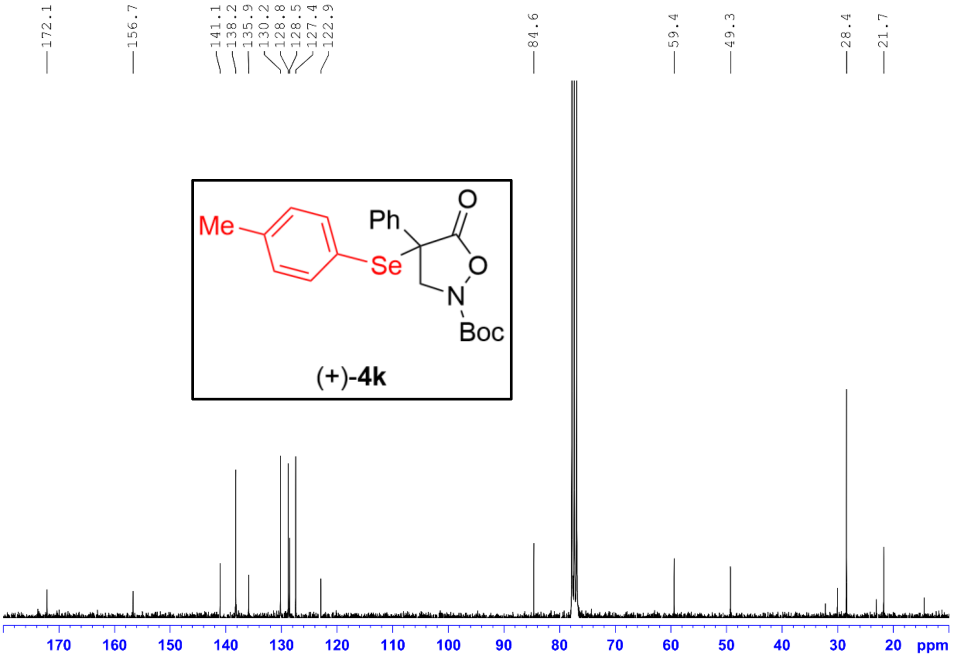

NMR spectra of compound **4l**

^{1}H-NMR (500 MHz, $CDCl_3$, 298 K)

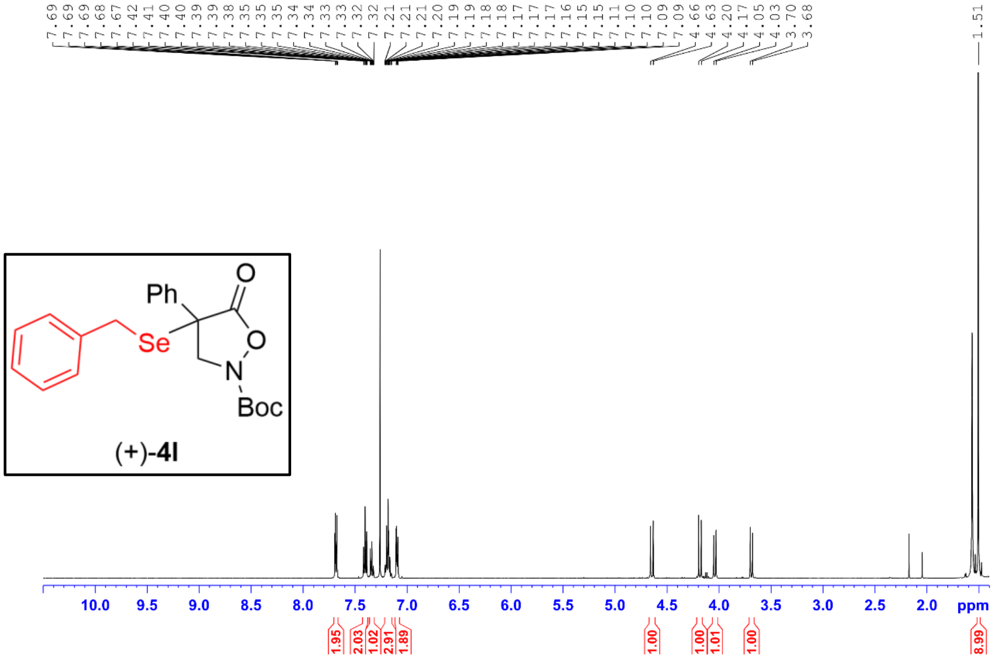

^{13}C-NMR (126 MHz, $CDCl_3$, 298 K)

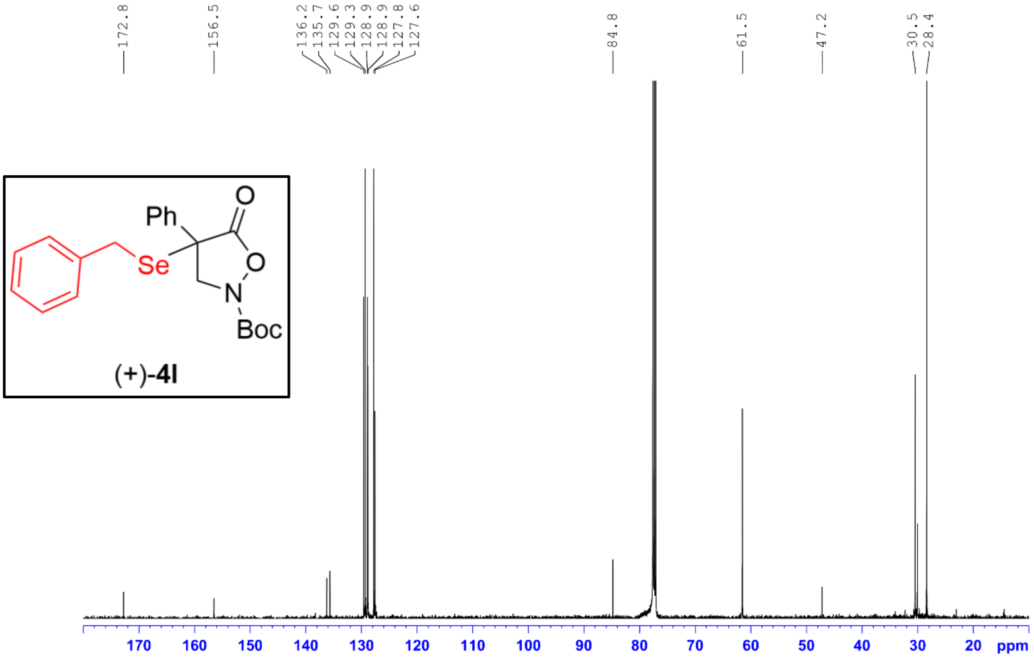

NMR spectra of compound **6**

^{1}H-NMR (300 MHz, $CDCl_3$, 298 K)

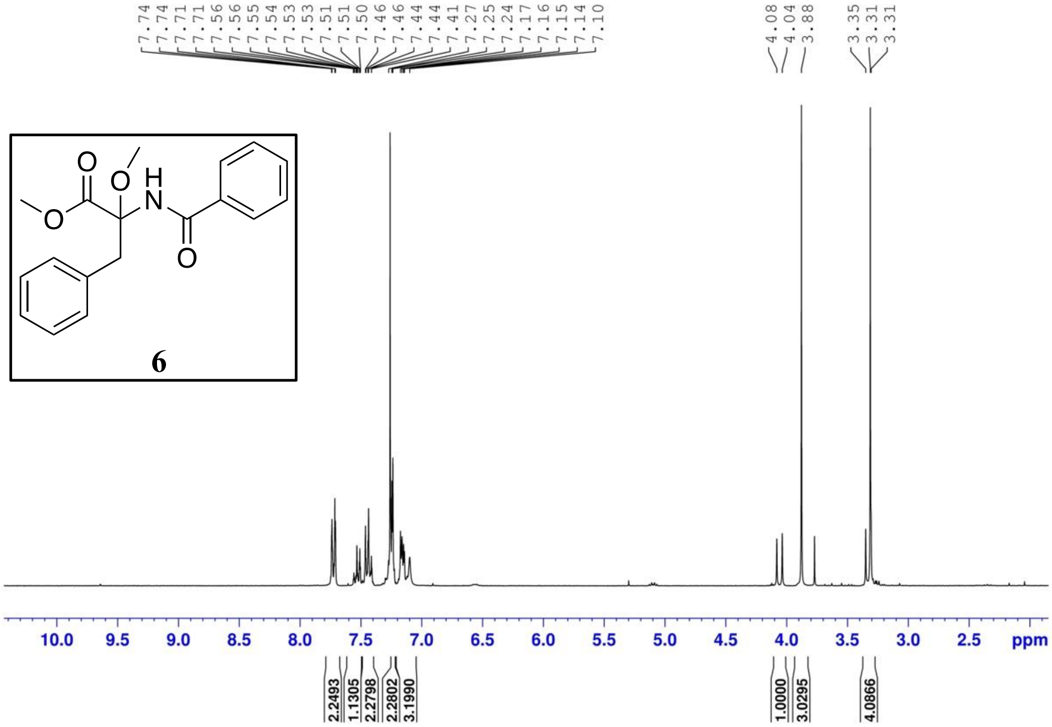

^{13}C-NMR (75 MHz, $CDCl_3$, 298 K)

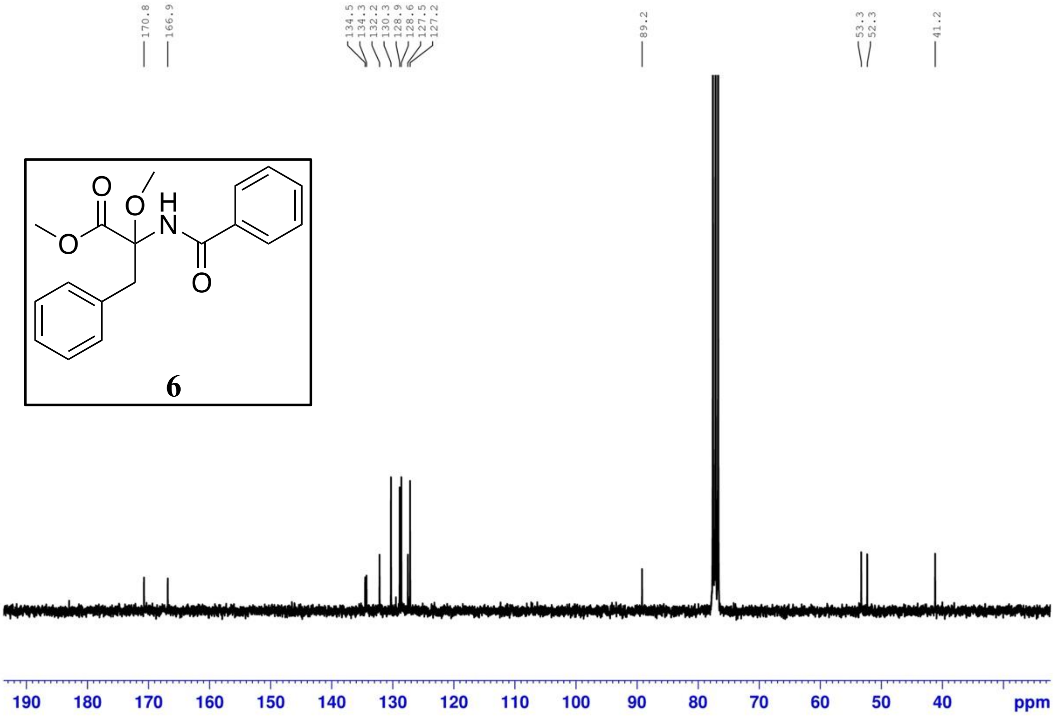

NMR spectra of compound **8**

^{1}H-NMR (300 MHz, $CDCl_3$, 298 K)

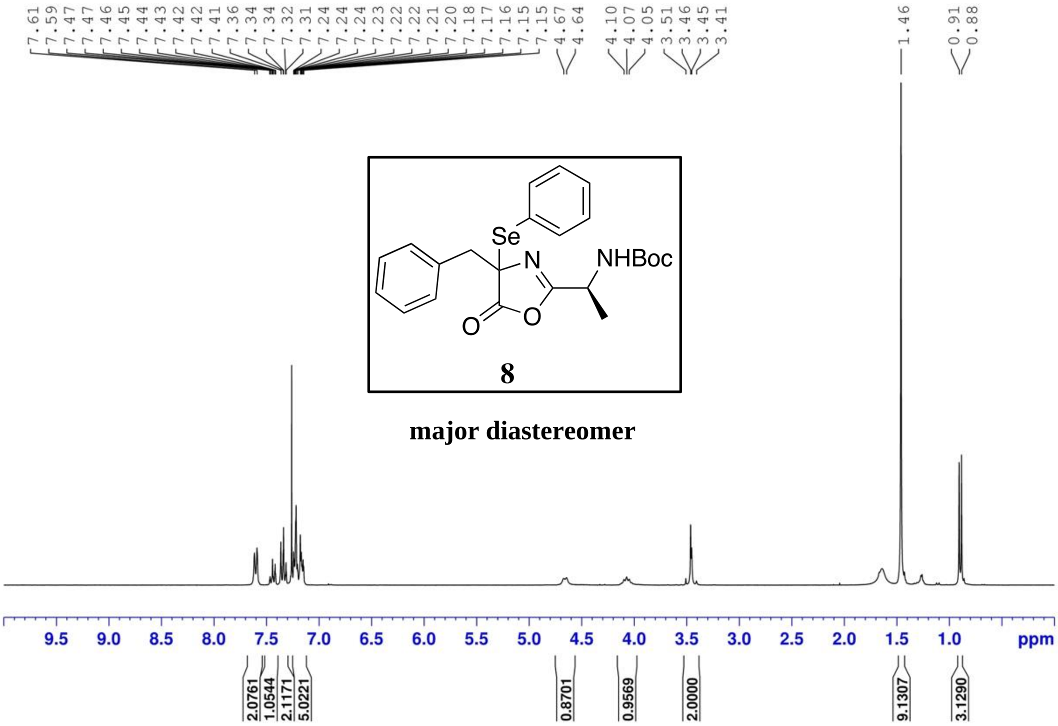

^{1}H-NMR (300 MHz, $CDCl_3$, 298 K)

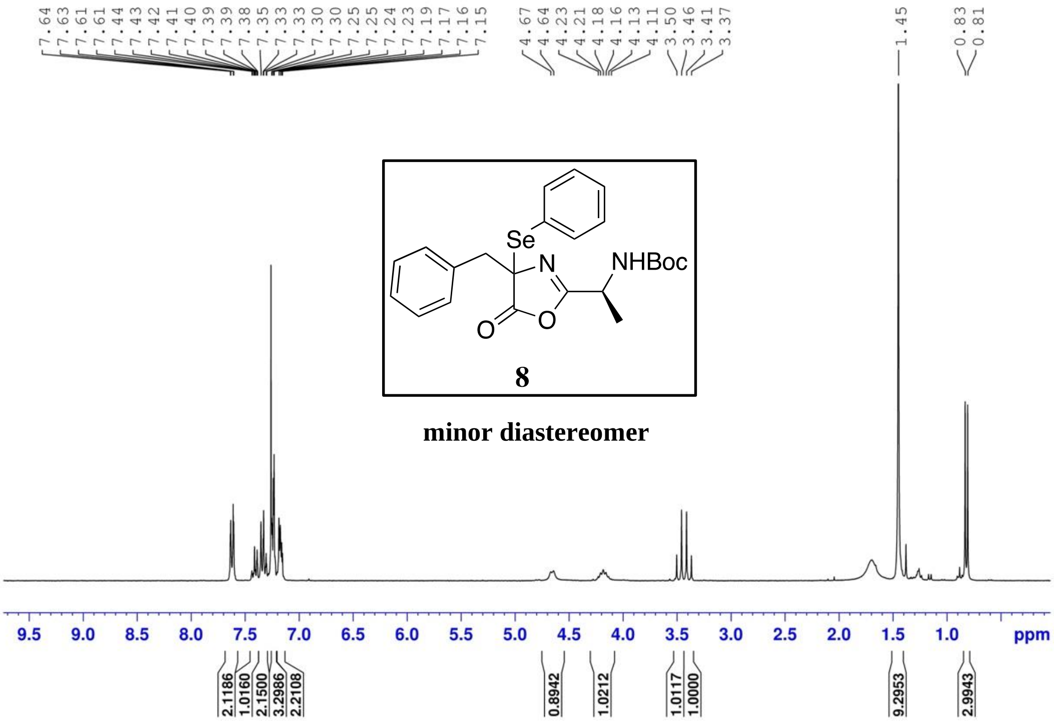

NMR spectra of compound **9a**

^{1}H-NMR (300 MHz, $CDCl_3$, 298 K)

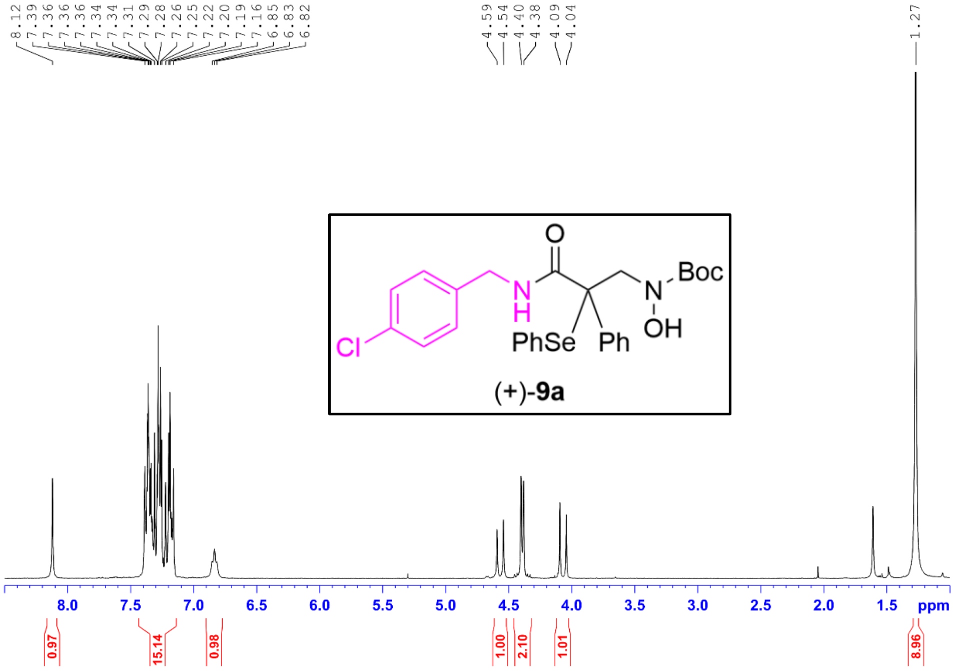

^{13}C-NMR (75 MHz, $CDCl_3$, 298 K)

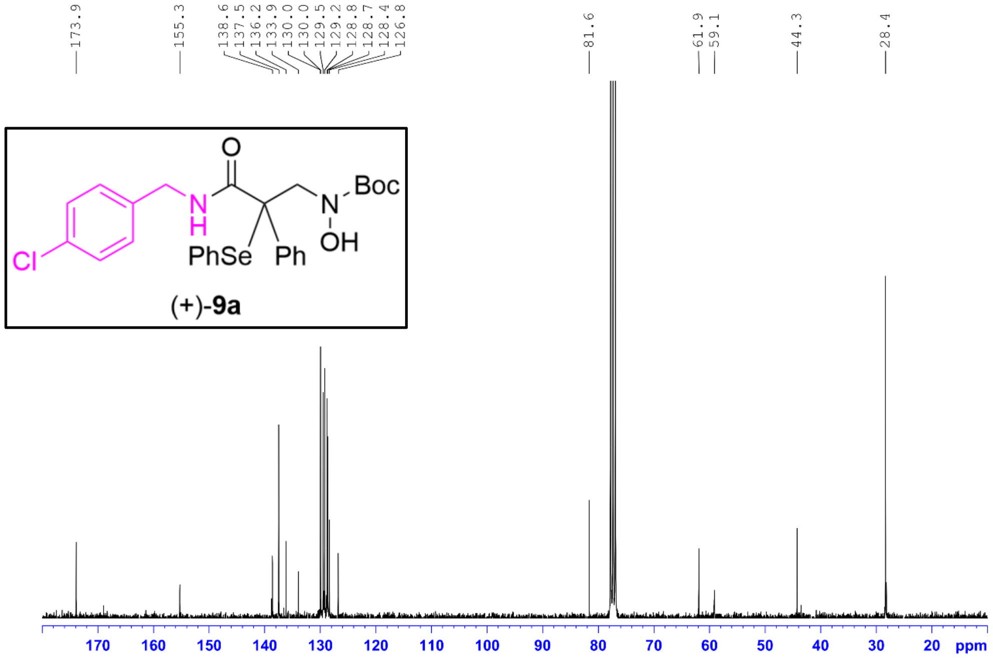

NMR spectra of compound **9b**

^{1}H-NMR (500 MHz, $CDCl_3$, 298 K)

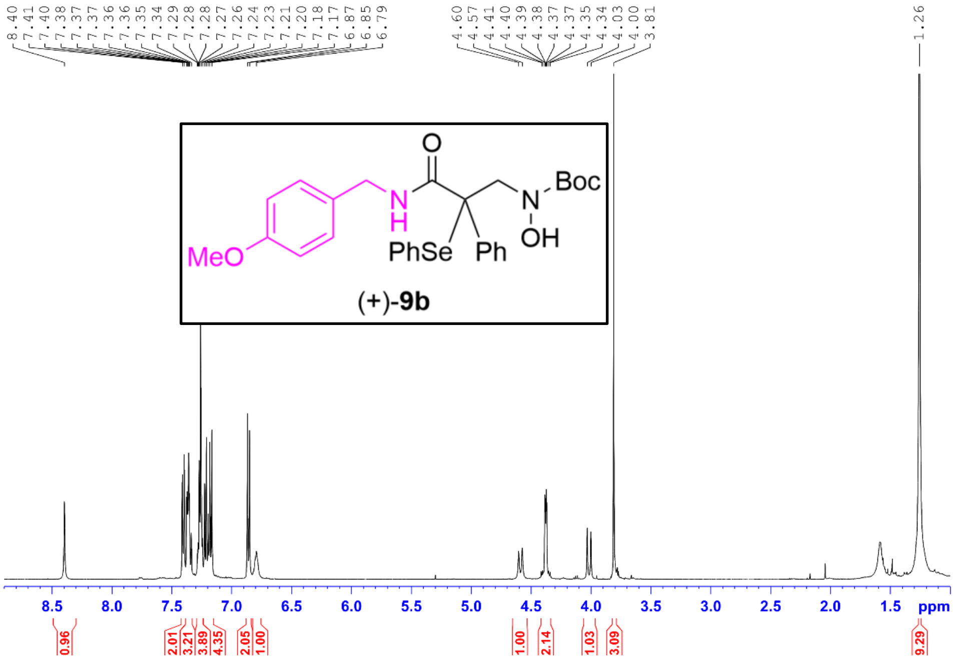

^{13}C-NMR (126 MHz, $CDCl_3$, 298 K)

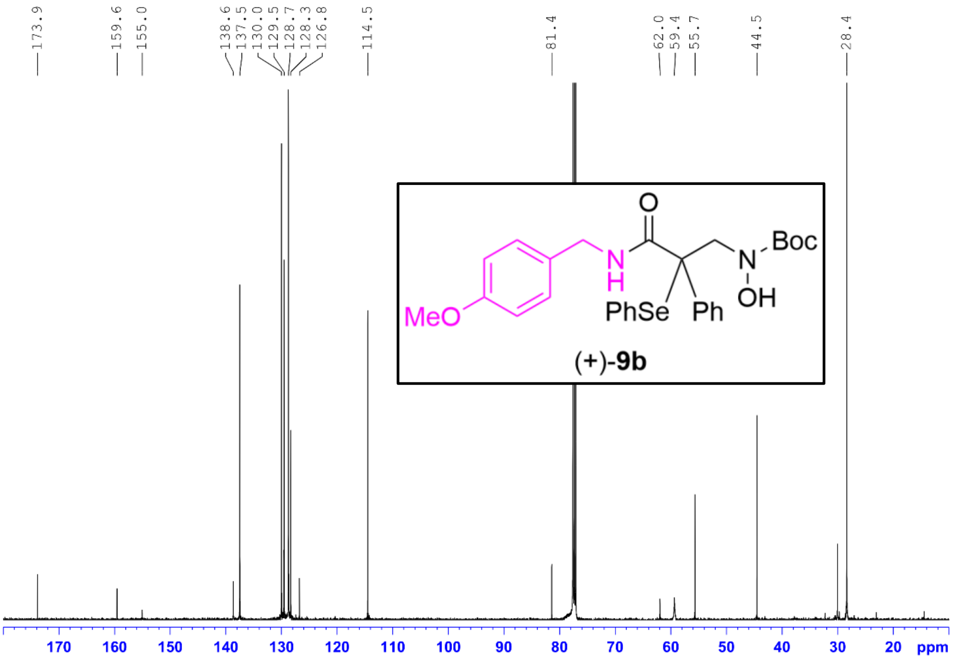

NMR spectra of compound **10**

^{1}H-NMR (300 MHz, $CDCl_3$, 298 K)

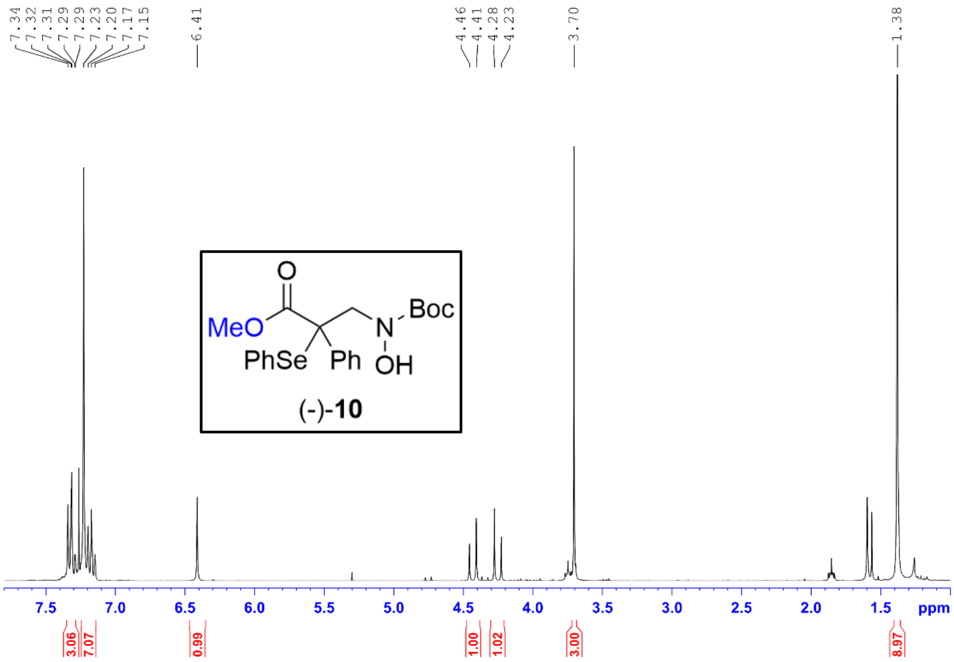

^{13}C-NMR (176 MHz, $CDCl_3$, 298 K)

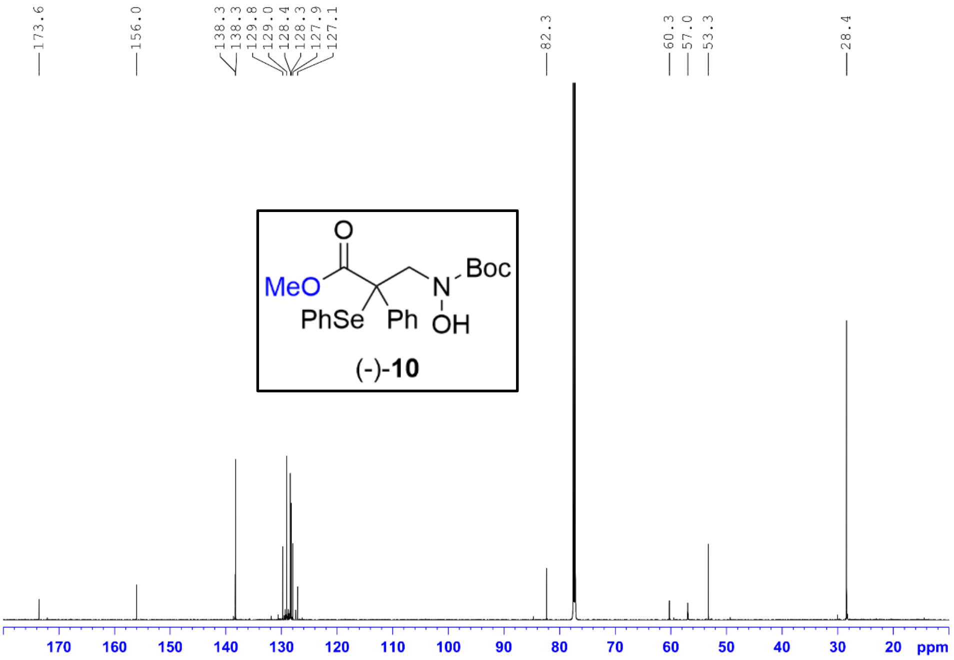

NMR spectra of compound **11**

^{1}H-NMR (300 MHz, $CDCl_3$, 298 K)

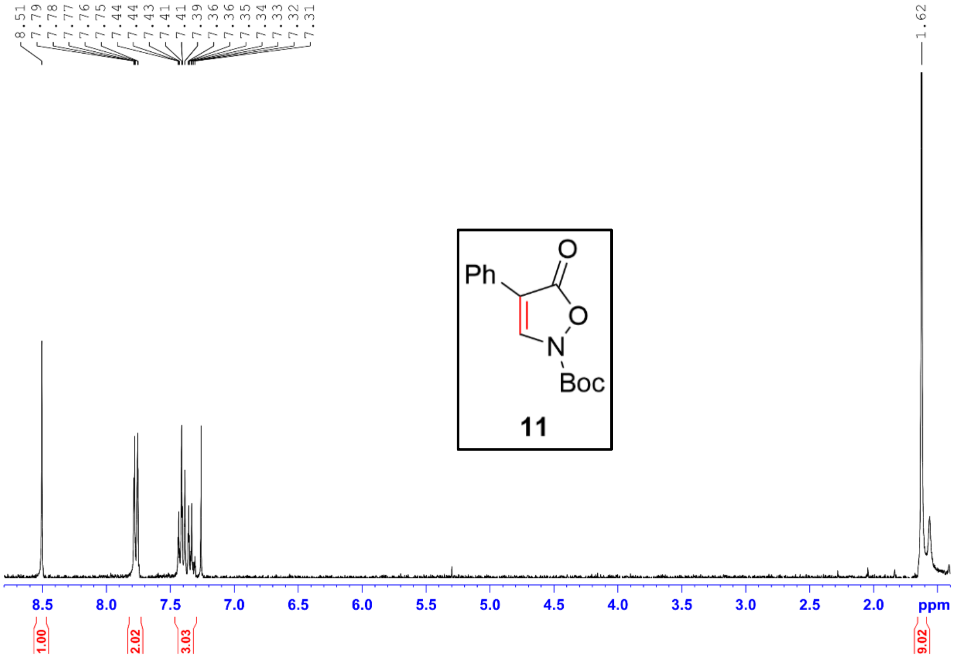

^{13}C-NMR (75 MHz, $CDCl_3$, 298 K)

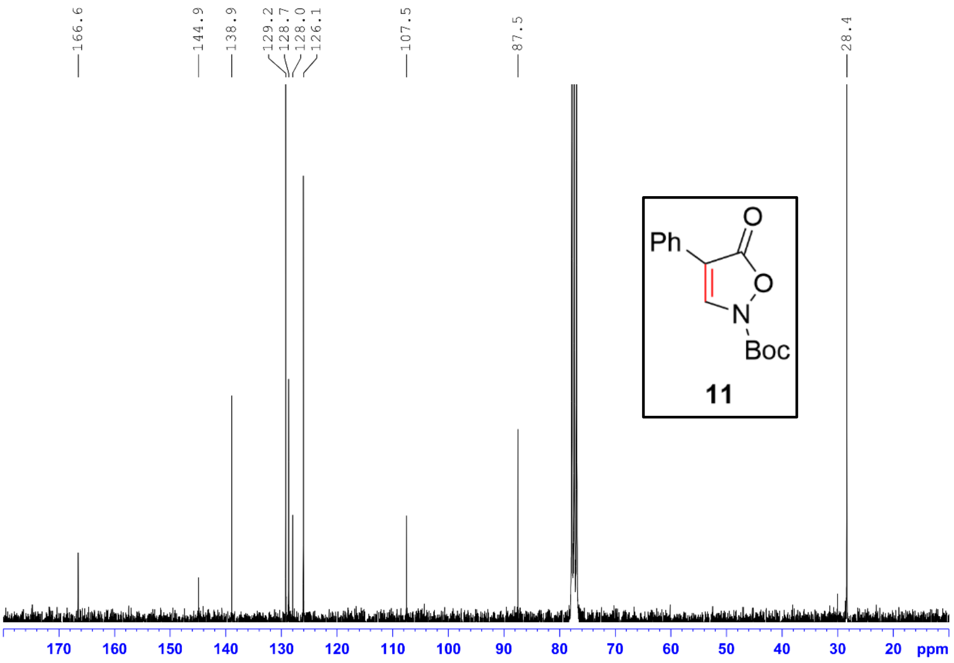

10.HPLC Traces of Racemic and Enantioenriched Selenation Products

HPLC traces of **3a** (Chiralpak AD-H, *n*-hexane:*i*-PrOH = 20:1, flow rate 0.3 mL/min, 10 °C, λ = 220 nm)

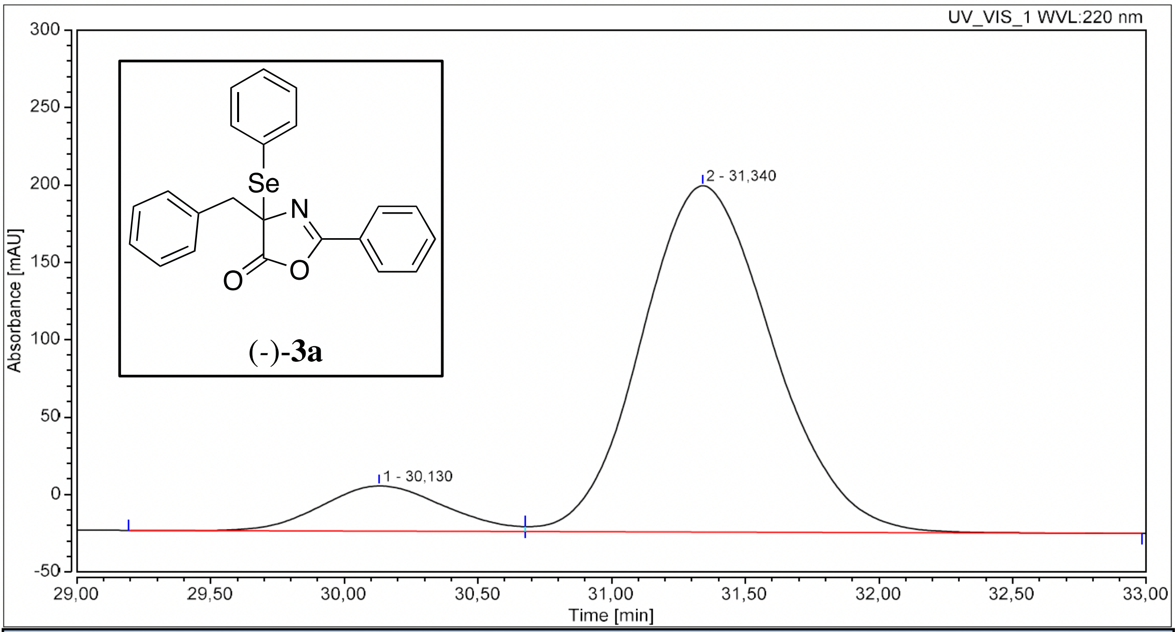

Integration Results							
No.	Peak Name	Retention Time min	Area mAU*min	Height mAU	Relative Area %	Relative Height %	Amount n.a.
1		30,130	15,593	29,159	10,92	11,53	n.a.
2		31,340	127,209	223,740	89,08	88,47	n.a.
**Total:**			**142,802**	**252,898**	**100,00**	**100,00**	

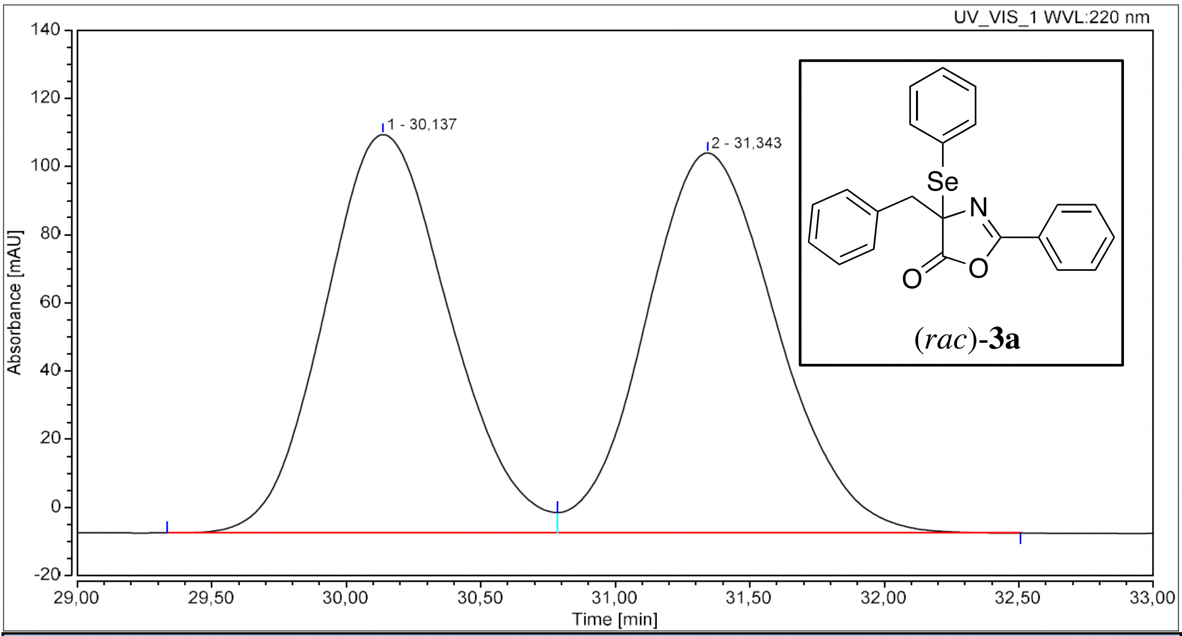

Integration Results							
No.	Peak Name	Retention Time min	Area mAU*min	Height mAU	Relative Area %	Relative Height %	Amount n.a.
1		30,137	62,977	116,797	49,95	51,17	n.a.
2		31,343	63,115	111,475	50,05	48,83	n.a.
**Total:**			**126,093**	**228,272**	**100,00**	**100,00**	

HPLC traces of **3c** (Chiralpak AD-H, *n*-hexane:*i*-PrOH = 20:1, flow rate 0.3 mL/min, 10 °C, λ = 220 nm)

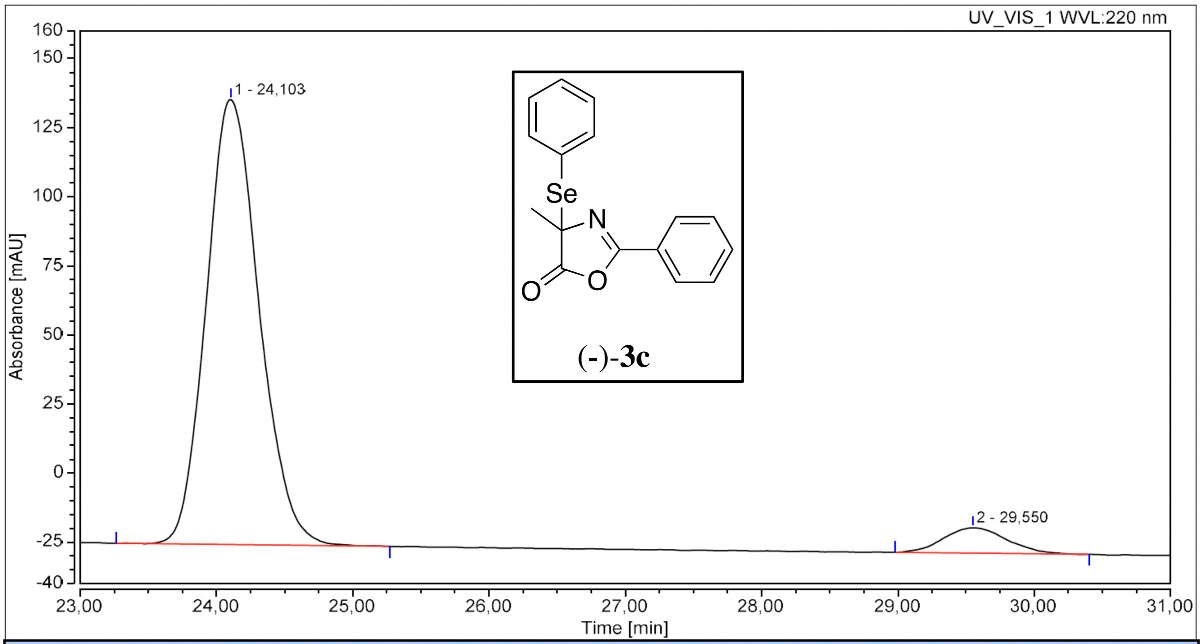

Integration Results							
No.	Peak Name	Retention Time min	Area mAU*min	Height mAU	Relative Area %	Relative Height %	Amount n.a.
1		24,103	72,320	161,061	93,59	94,61	n.a.
2		29,550	4,957	9,183	6,41	5,39	n.a.
**Total:**			**77,277**	**170,244**	**100,00**	**100,00**	

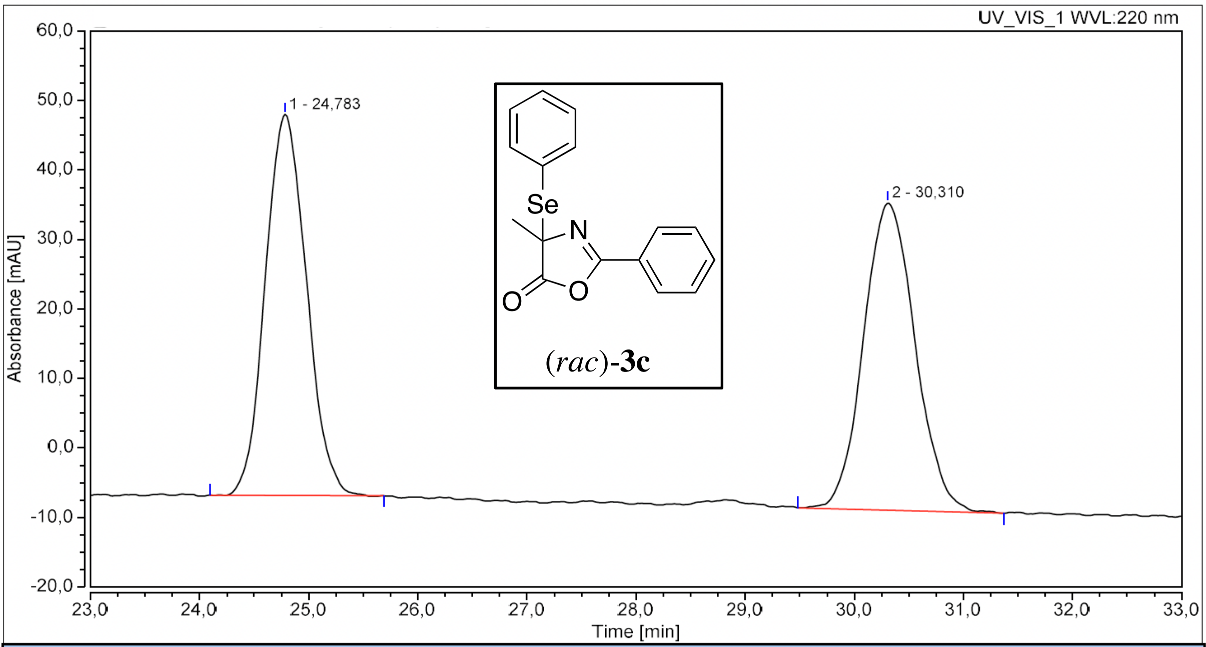

Integration Results							
No.	Peak Name	Retention Time min	Area mAU*min	Height mAU	Relative Area %	Relative Height %	Amount n.a.
1		24,783	23,942	54,827	49,75	55,36	n.a.
2		30,310	24,183	44,204	50,25	44,64	n.a.
**Total:**			**48,125**	**99,030**	**100,00**	**100,00**	

HPLC traces of **3d** (Chiralpak AD-H, *n*-hexane:*i*-PrOH = 20:1, flow rate 0.3 mL/min, 10 °C, λ = 220 nm)

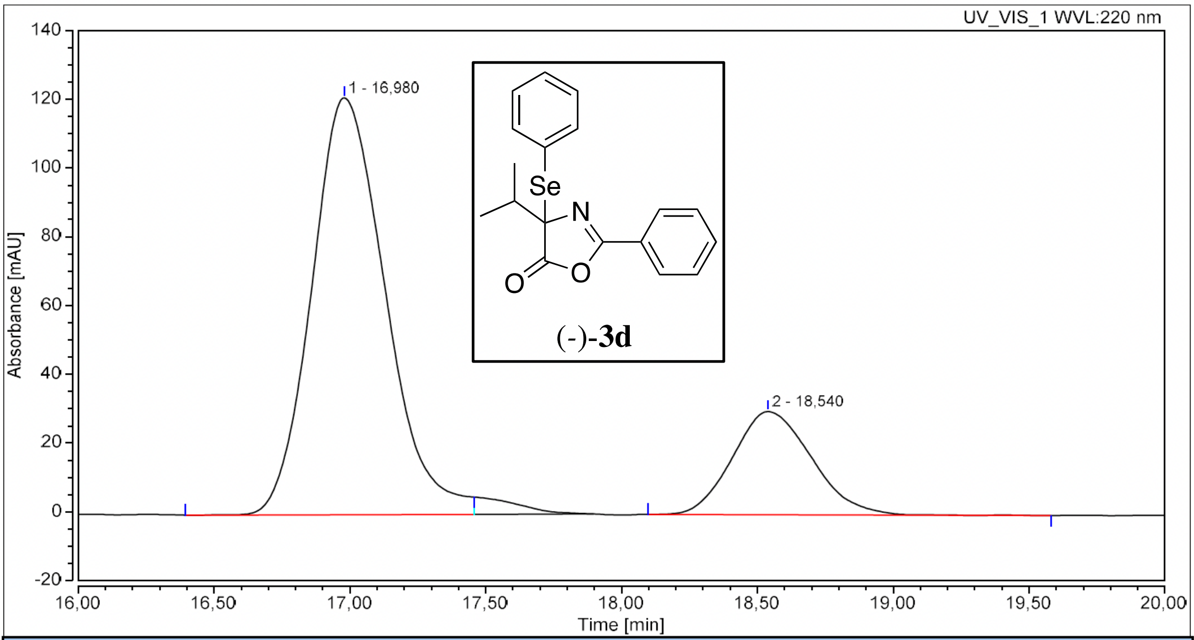

Integration Results

No.	Peak Name	Retention Time min	Area mAU*min	Height mAU	Relative Area %	Relative Height %	Amount n.a.
1		16,980	37,992	121,260	78,91	80,16	n.a.
2		18,540	10,152	30,013	21,09	19,84	n.a.
**Total:**			**48,144**	**151,273**	**100,00**	**100,00**	

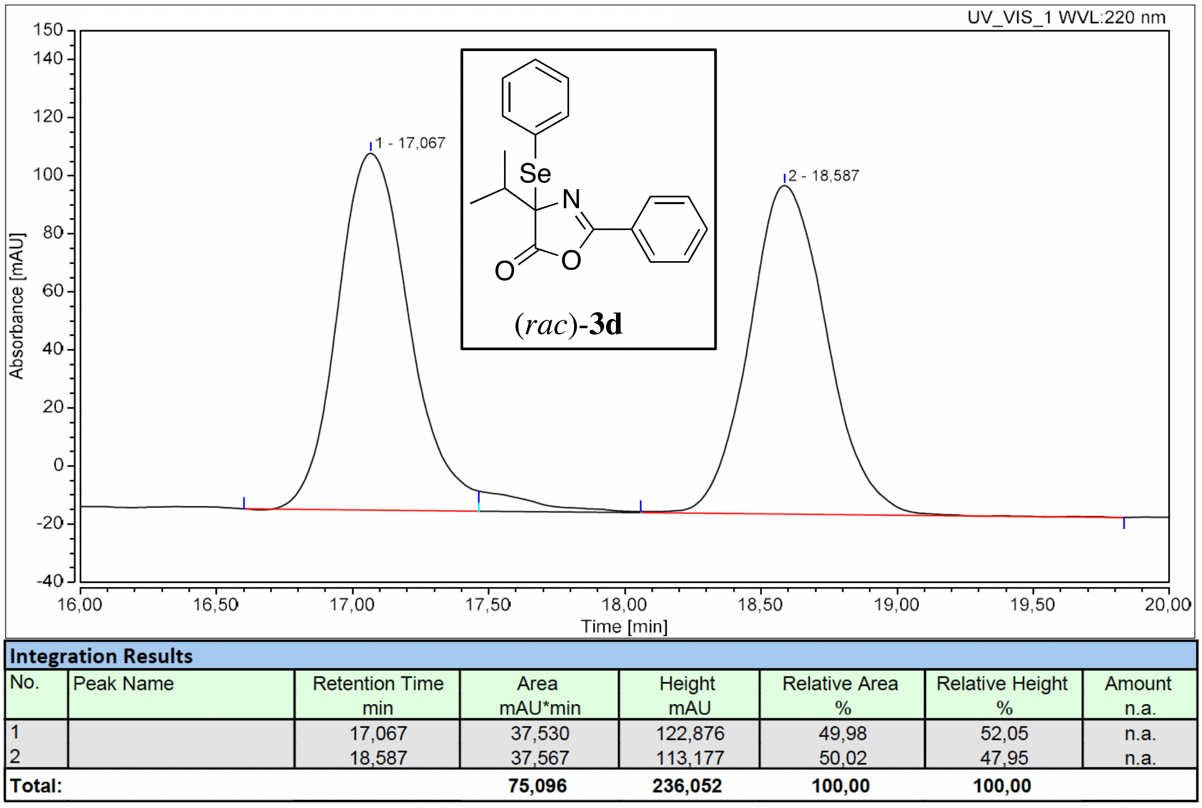

Integration Results

No.	Peak Name	Retention Time min	Area mAU*min	Height mAU	Relative Area %	Relative Height %	Amount n.a.
1		17,067	37,530	122,876	49,98	52,05	n.a.
2		18,587	37,567	113,177	50,02	47,95	n.a.
**Total:**			**75,096**	**236,052**	**100,00**	**100,00**	

HPLC traces of **3e** (YMC Chiral Art Cellulose SB, *n*-hexane:*i*-PrOH = 80:1, flow rate 0.2 mL/min, 10 °C, λ = 220 nm)

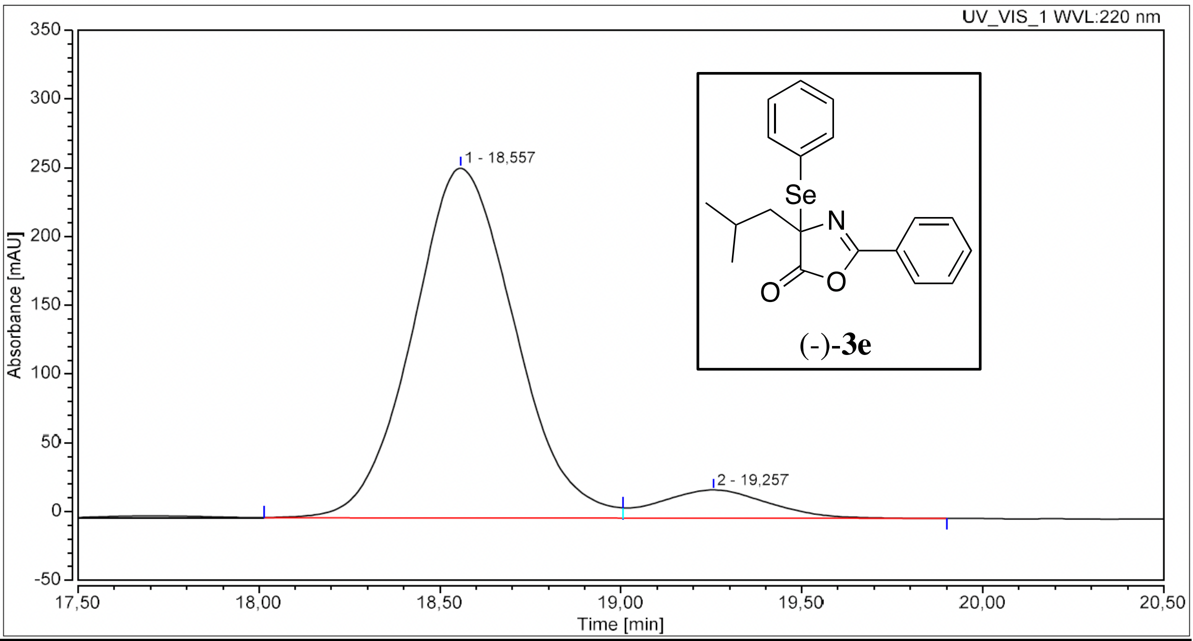

Integration Results							
No.	Peak Name	Retention Time min	Area mAU*min	Height mAU	Relative Area %	Relative Height %	Amount n.a.
1		18,557	83,280	254,286	92,17	92,53	n.a.
2		19,257	7,079	20,532	7,83	7,47	n.a.
**Total:**			**90,360**	**274,817**	**100,00**	**100,00**	

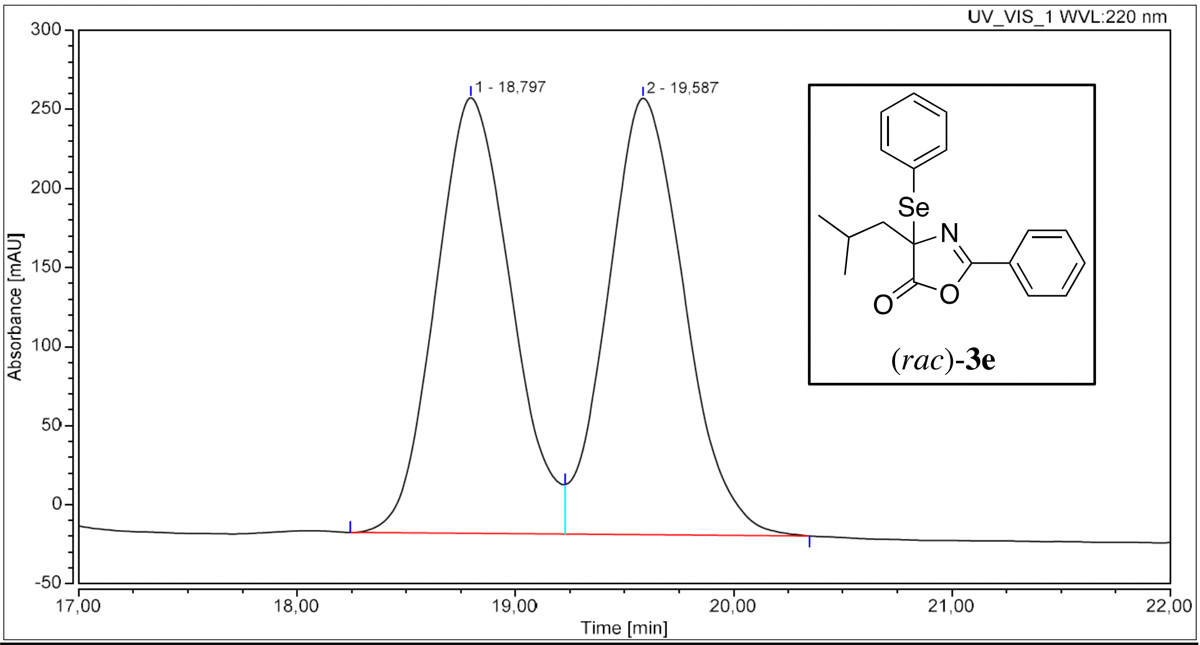

Integration Results							
No.	Peak Name	Retention Time min	Area mAU*min	Height mAU	Relative Area %	Relative Height %	Amount n.a.
1		18,797	110,241	275,508	49,49	49,95	n.a.
2		19,587	112,502	276,073	50,51	50,05	n.a.
**Total:**			**222,743**	**551,581**	**100,00**	**100,00**	

HPLC traces of **3f** (Chiralpak AD-H, *n*-hexane:*i*-PrOH = 20:1, flow rate 0.7 mL/min, 10 °C, λ = 220 nm)

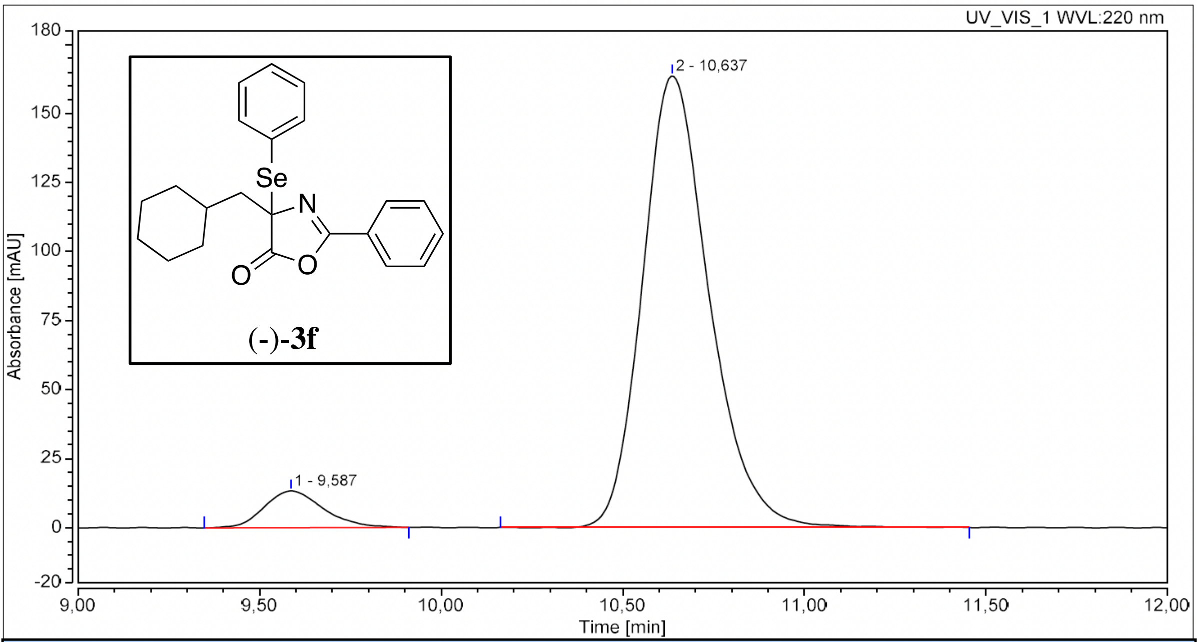

Integration Results							
No.	Peak Name	Retention Time min	Area mAU*min	Height mAU	Relative Area %	Relative Height %	Amount n.a.
1		9,587	2,515	13,315	6,74	7,52	n.a.
2		10,637	34,823	163,633	93,26	92,48	n.a.
Total:			37,339	176,948	100,00	100,00	

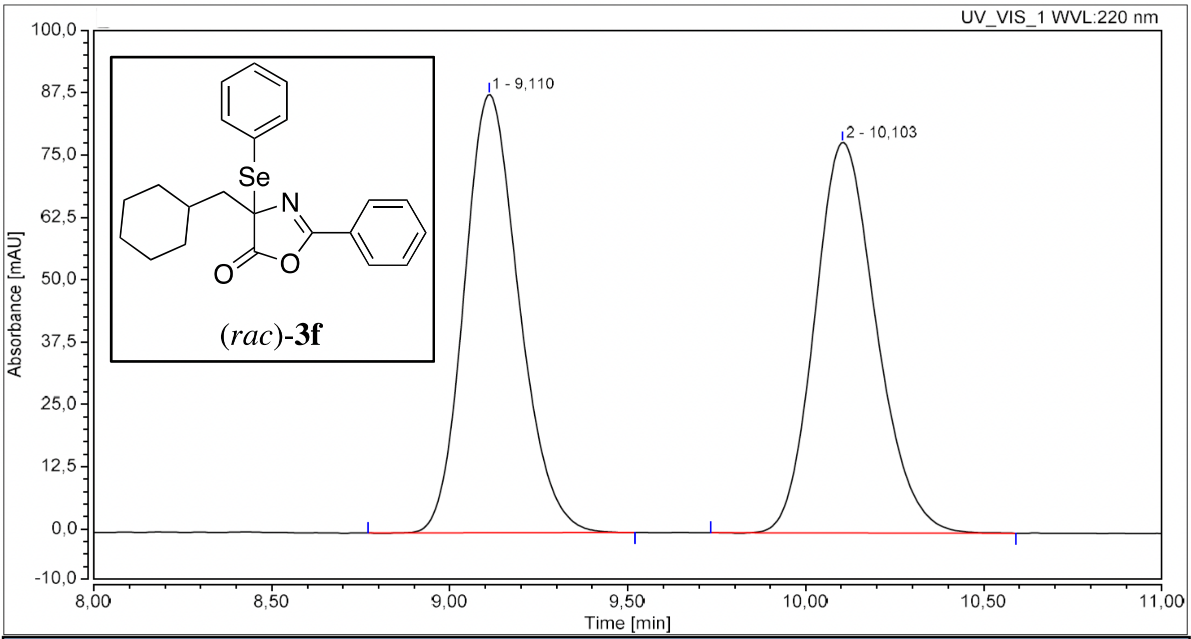

Integration Results							
No.	Peak Name	Retention Time min	Area mAU*min	Height mAU	Relative Area %	Relative Height %	Amount n.a.
1		9,110	15,674	87,913	49,98	52,89	n.a.
2		10,103	15,689	78,311	50,02	47,11	n.a.
Total:			31,363	166,224	100,00	100,00	

HPLC traces of **3g** (Chiralpak AD-H, *n*-hexane:*i*-PrOH = 20:1, flow rate 0.3 mL/min, 10 °C, λ = 220 nm)

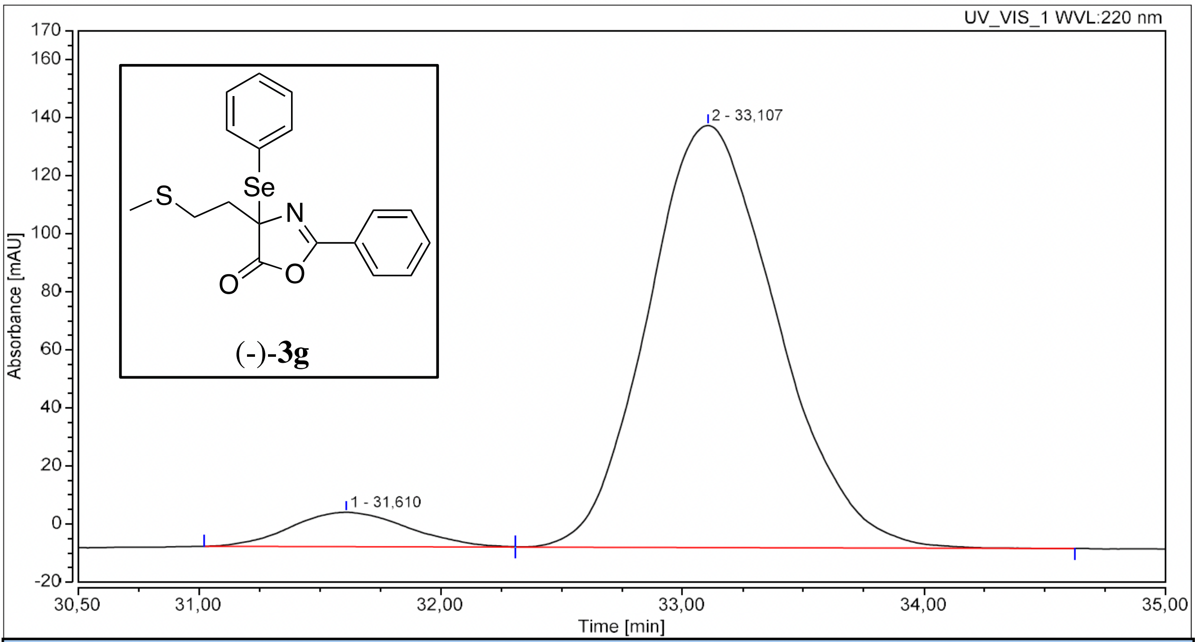

Integration Results							
No.	Peak Name	Retention Time min	Area mAU*min	Height mAU	Relative Area %	Relative Height %	Amount n.a.
1		31,610	6,619	11,814	6,84	7,51	n.a.
2		33,107	90,106	145,434	93,16	92,49	n.a.
**Total:**			**96,725**	**157,248**	**100,00**	**100,00**	

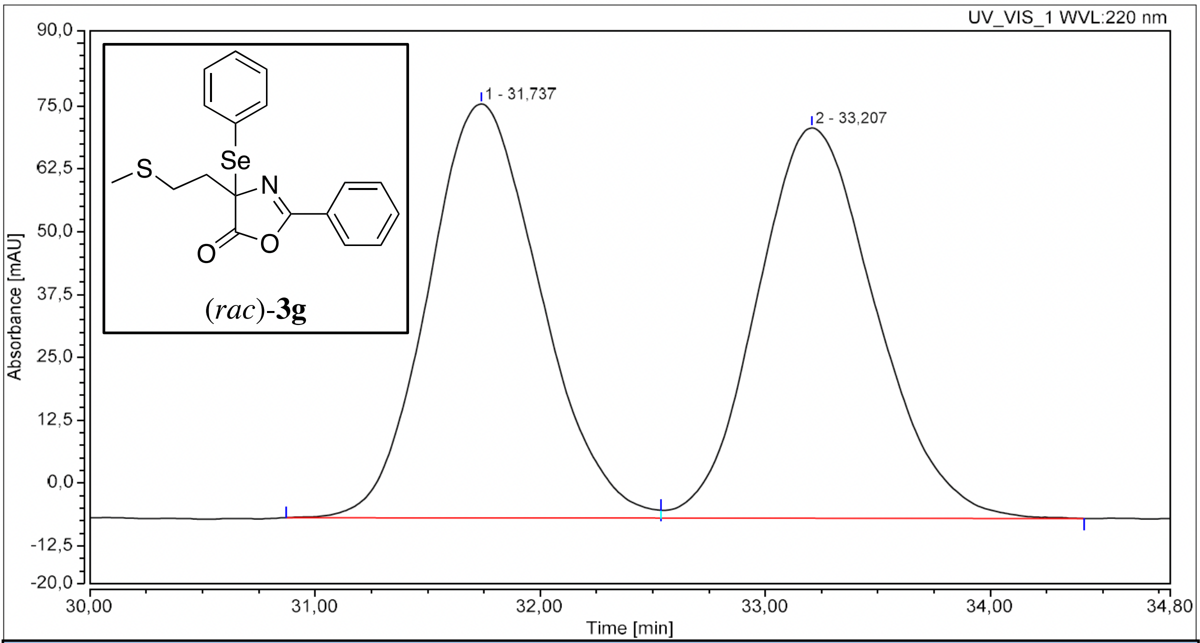

Integration Results							
No.	Peak Name	Retention Time min	Area mAU*min	Height mAU	Relative Area %	Relative Height %	Amount n.a.
1		31,737	47,567	82,317	50,03	51,47	n.a.
2		33,207	47,516	77,622	49,97	48,53	n.a.
**Total:**			**95,084**	**159,939**	**100,00**	**100,00**	

HPLC traces of **3h** (Chiralpak AD-H, *n*-hexane:*i*-PrOH = 20:1, flow rate 0.3 mL/min, 10 °C, λ = 220 nm)

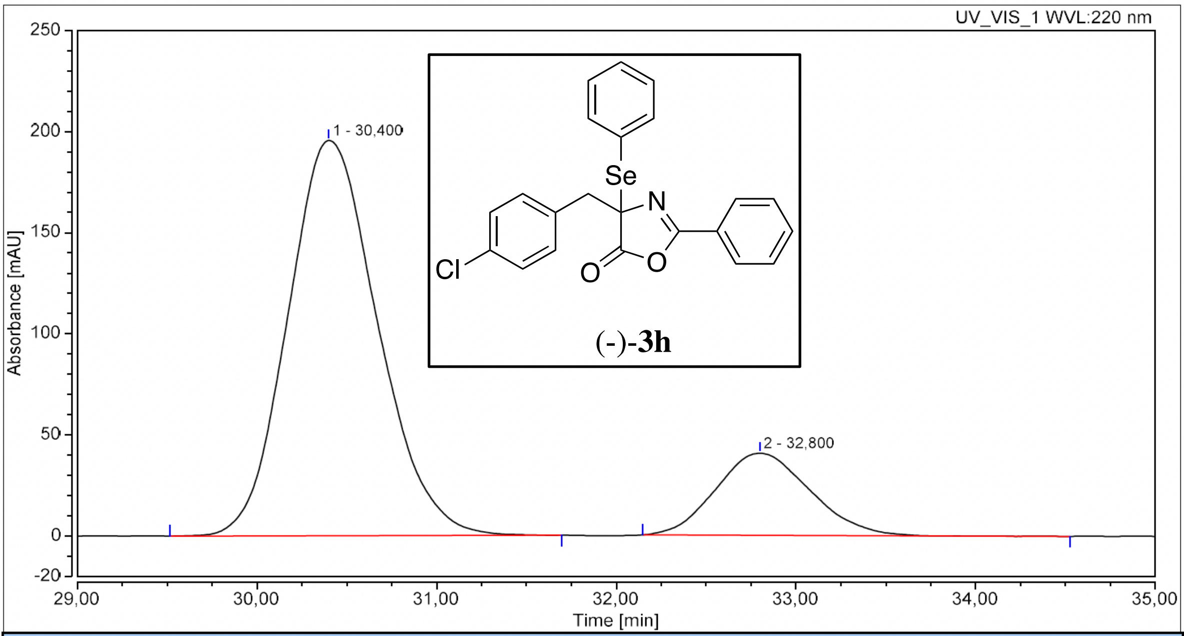

Integration Results							
No.	Peak Name	Retention Time min	Area mAU*min	Height mAU	Relative Area %	Relative Height %	Amount n.a.
1		30,400	113,049	195,421	82,14	82,83	n.a.
2		32,800	24,573	40,520	17,86	17,17	n.a.
**Total:**			**137,622**	**235,941**	**100,00**	**100,00**	

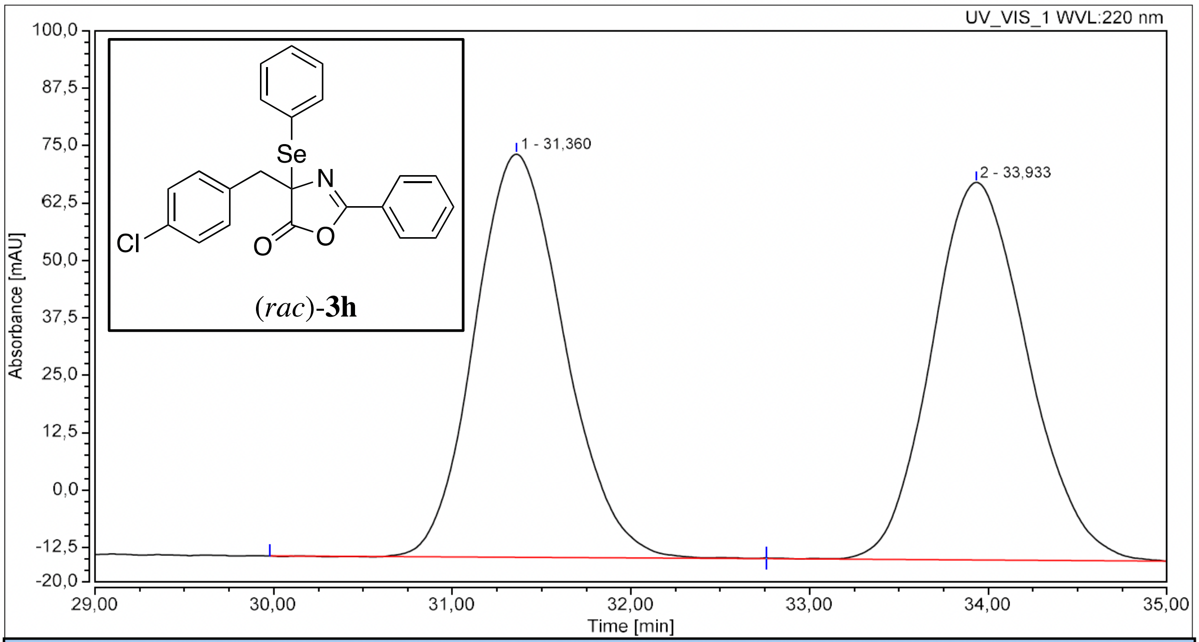

Integration Results							
No.	Peak Name	Retention Time min	Area mAU*min	Height mAU	Relative Area %	Relative Height %	Amount n.a.
1		31,360	51,060	87,776	49,91	51,63	n.a.
2		33,933	51,237	82,219	50,09	48,37	n.a.
**Total:**			**102,296**	**169,996**	**100,00**	**100,00**	

HPLC traces of **3i** (Chiralpak AD-H, *n*-hexane:*i*-PrOH = 20:1, flow rate 0.7 mL/min, 10 °C, λ = 220 nm)

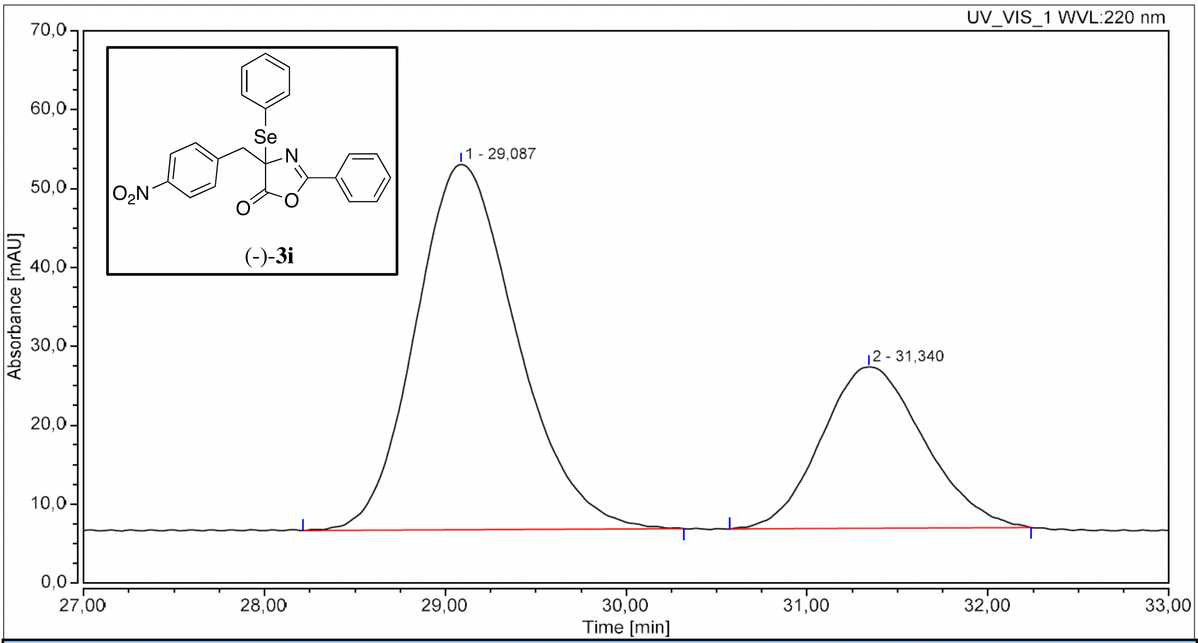

Integration Results

No.	Peak Name	Retention Time min	Area mAU*min	Height mAU	Relative Area %	Relative Height %	Amount n.a.
1		29,087	30,118	46,288	69,13	69,36	n.a.
2		31,340	13,451	20,449	30,87	30,64	n.a.
**Total:**			**43,568**	**66,737**	**100,00**	**100,00**	

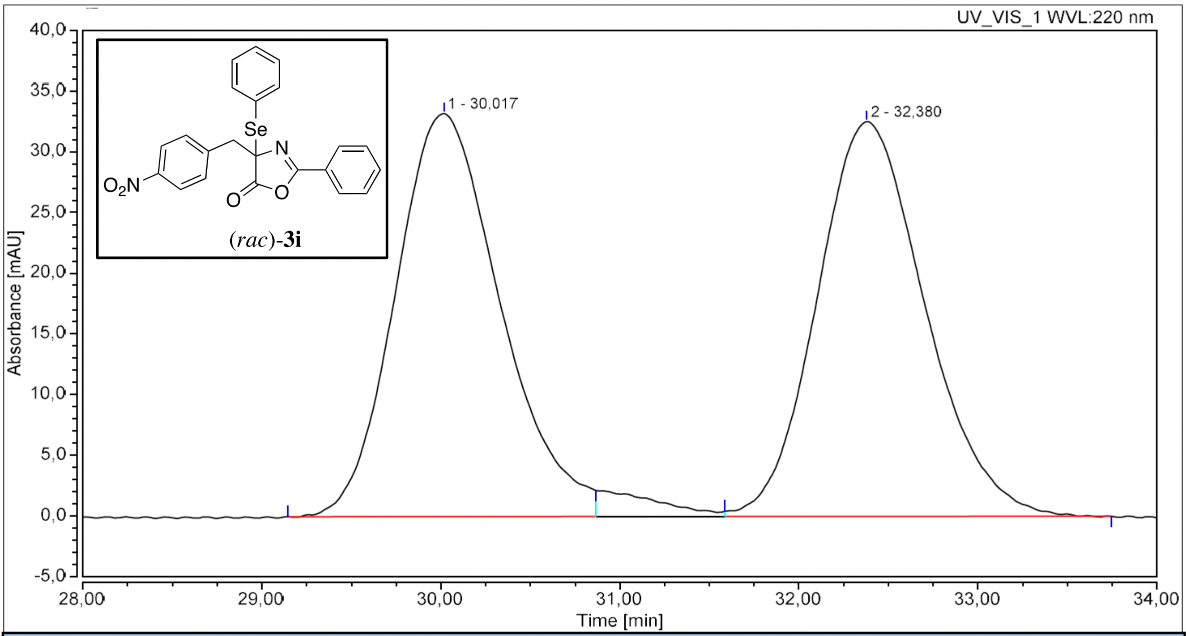

Integration Results

No.	Peak Name	Retention Time min	Area mAU*min	Height mAU	Relative Area %	Relative Height %	Amount n.a.
1		30,017	22,463	33,199	49,78	50,52	n.a.
2		32,380	22,664	32,516	50,22	49,48	n.a.
**Total:**			**45,127**	**65,715**	**100,00**	**100,00**	

HPLC traces of **3j** (Chiralpak AD-H, *n*-hexane:*i*-PrOH = 20:1, flow rate 0.3 mL/min, 10 °C, λ = 220 nm)

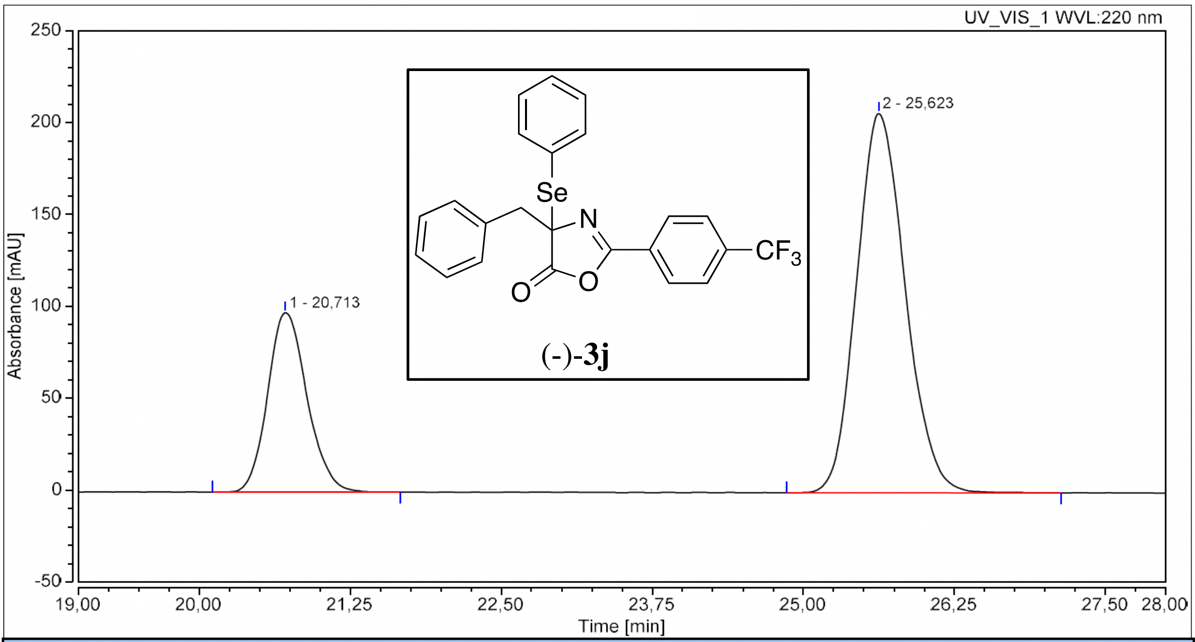

Integration Results							
No.	Peak Name	Retention Time min	Area mAU*min	Height mAU	Relative Area %	Relative Height %	Amount n.a.
1		20,713	36,934	97,656	27,73	32,11	n.a.
2		25,623	96,240	206,429	72,27	67,89	n.a.
**Total:**			**133,174**	**304,085**	**100,00**	**100,00**	

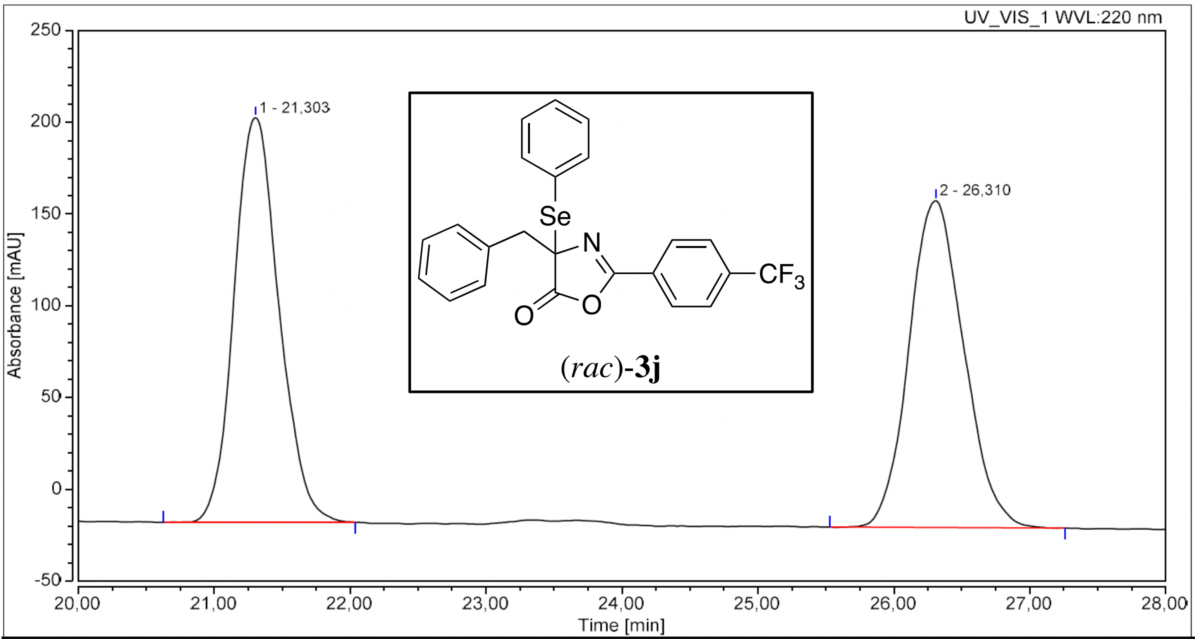

Integration Results							
No.	Peak Name	Retention Time min	Area mAU*min	Height mAU	Relative Area %	Relative Height %	Amount n.a.
1		21,303	84,446	220,547	50,35	55,32	n.a.
2		26,310	83,278	178,111	49,65	44,68	n.a.
**Total:**			**167,724**	**398,659**	**100,00**	**100,00**	

HPLC traces of **3k** (Chiralpak AD-H, *n*-hexane:*i*-PrOH = 20:1, flow rate 0.7 mL/min, 10 °C, λ = 220 nm)

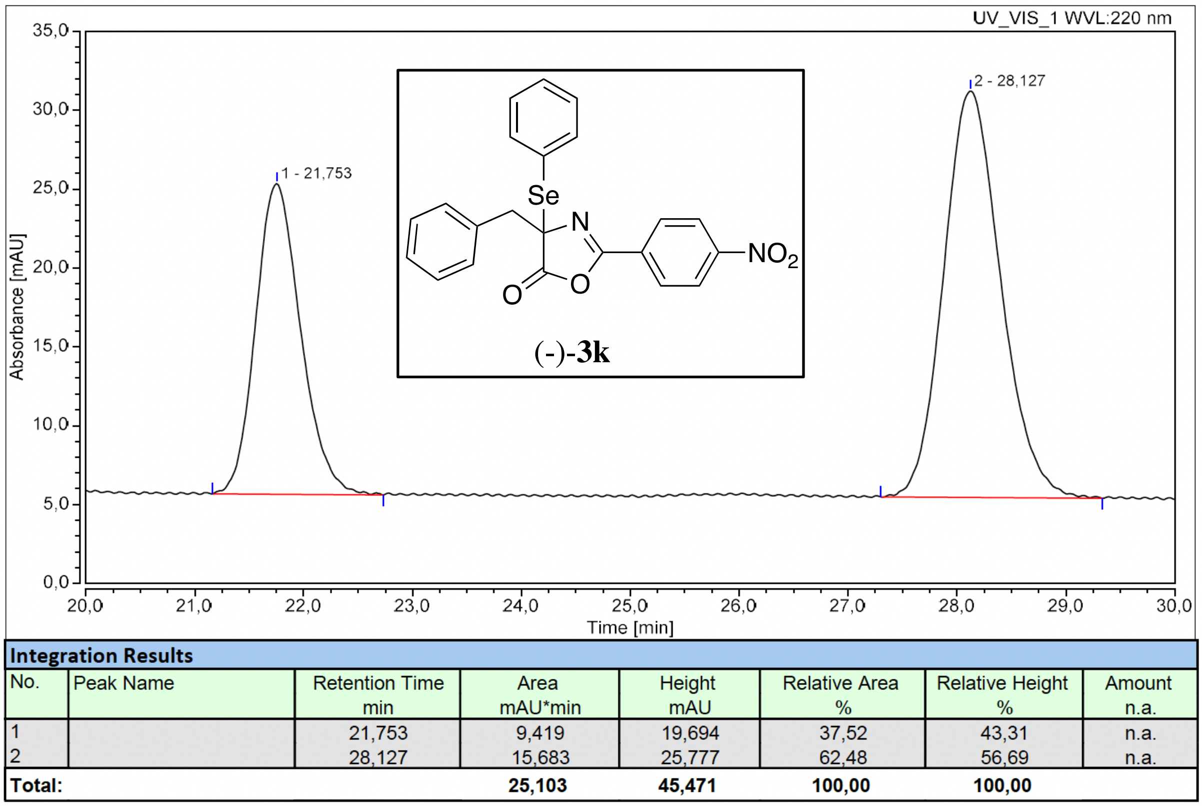

Integration Results							
No.	Peak Name	Retention Time min	Area mAU*min	Height mAU	Relative Area %	Relative Height %	Amount n.a.
1		21,753	9,419	19,694	37,52	43,31	n.a.
2		28,127	15,683	25,777	62,48	56,69	n.a.
**Total:**			**25,103**	**45,471**	**100,00**	**100,00**	

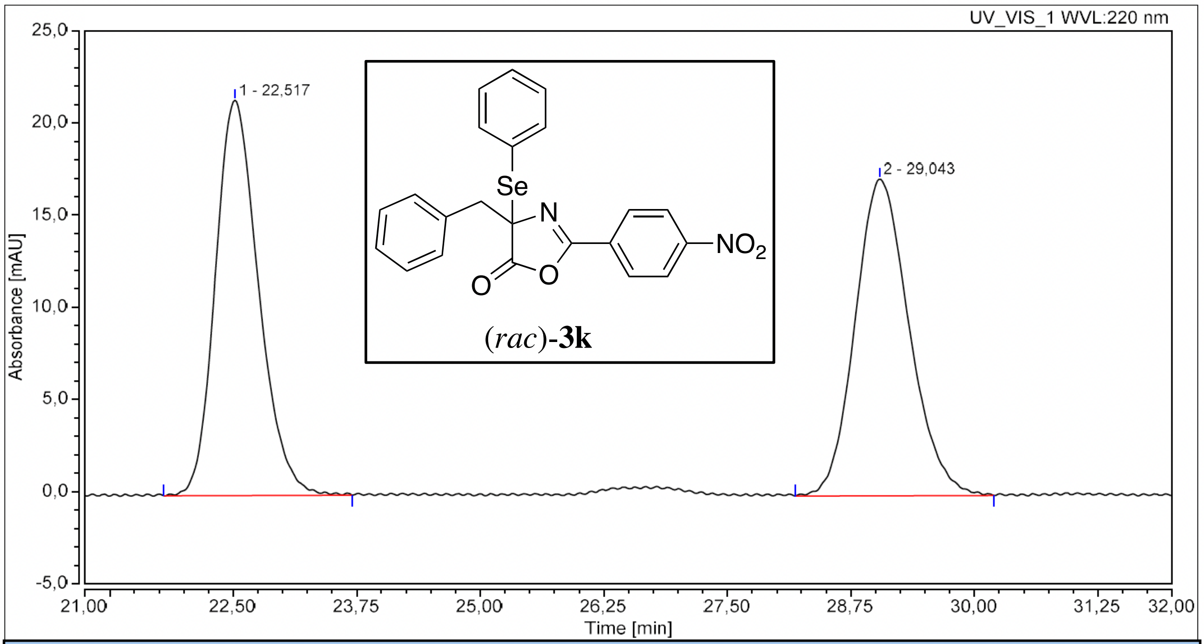

Integration Results							
No.	Peak Name	Retention Time min	Area mAU*min	Height mAU	Relative Area %	Relative Height %	Amount n.a.
1		22,517	10,868	21,443	49,93	55,50	n.a.
2		29,043	10,900	17,193	50,07	44,50	n.a.
**Total:**			**21,768**	**38,636**	**100,00**	**100,00**	

HPLC traces of **3l** (Chiralpak AD-H, *n*-hexane:*i*-PrOH = 20:1, flow rate 0.7 mL/min, 10 °C, λ = 270 nm)

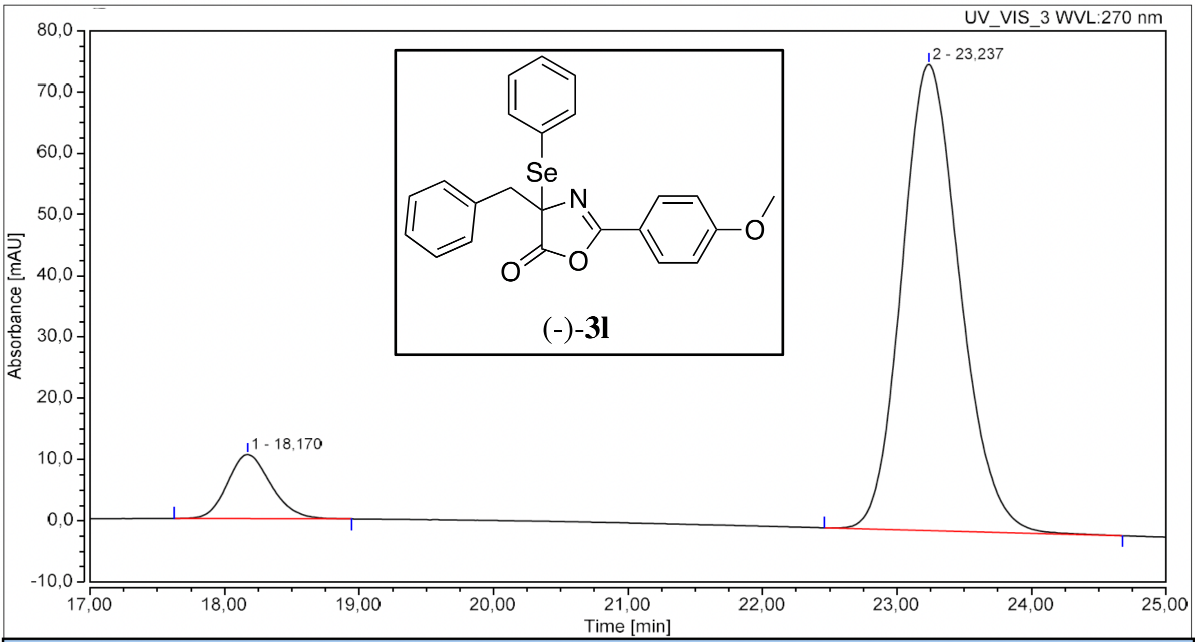

Integration Results

No.	Peak Name	Retention Time min	Area mAU*min	Height mAU	Relative Area %	Relative Height %	Amount n.a.
1		18,170	3,917	10,460	9,32	12,08	n.a.
2		23,237	38,109	76,121	90,68	87,92	n.a.
**Total:**			**42,026**	**86,580**	**100,00**	**100,00**	

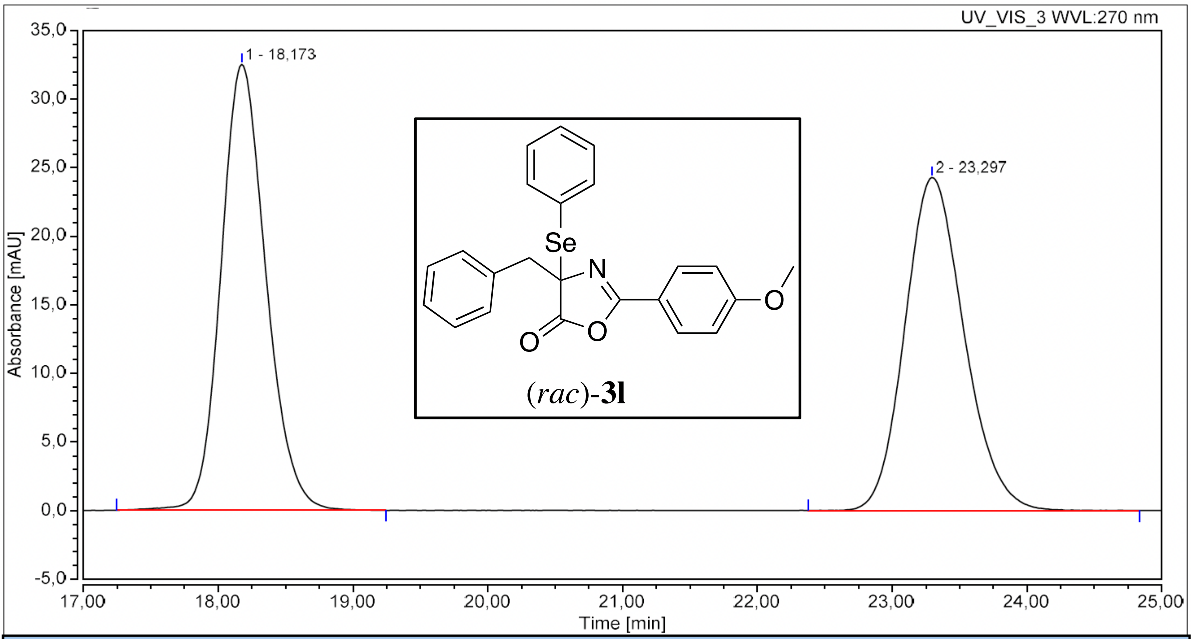

Integration Results

No.	Peak Name	Retention Time min	Area mAU*min	Height mAU	Relative Area %	Relative Height %	Amount n.a.
1		18,173	12,577	32,504	50,43	57,24	n.a.
2		23,297	12,361	24,280	49,57	42,76	n.a.
**Total:**			**24,939**	**56,784**	**100,00**	**100,00**	

HPLC traces of **3m** (Chiralpak AD-H, *n*-hexane:*i*-PrOH = 20:1, flow rate 0.3 mL/min, 10 °C, λ = 220 nm)

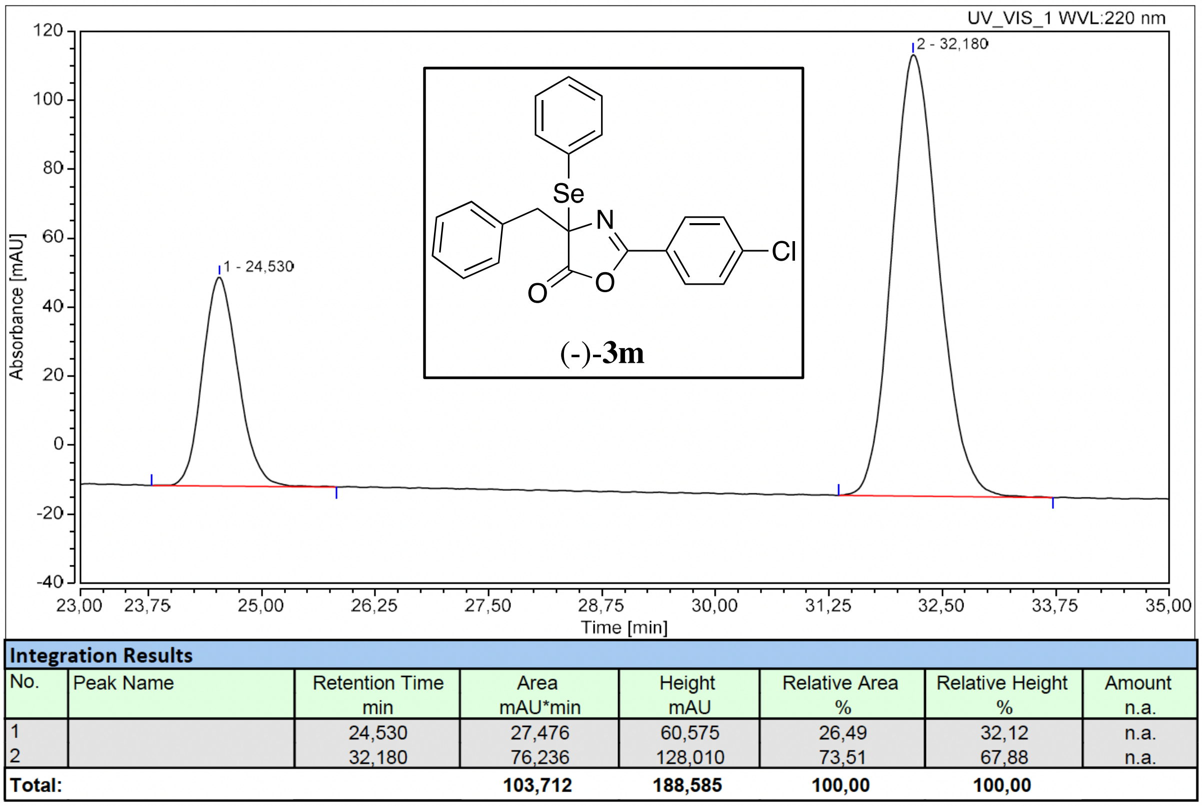

Integration Results

No.	Peak Name	Retention Time min	Area mAU*min	Height mAU	Relative Area %	Relative Height %	Amount n.a.
1		24,530	27,476	60,575	26,49	32,12	n.a.
2		32,180	76,236	128,010	73,51	67,88	n.a.
**Total:**			**103,712**	**188,585**	**100,00**	**100,00**	

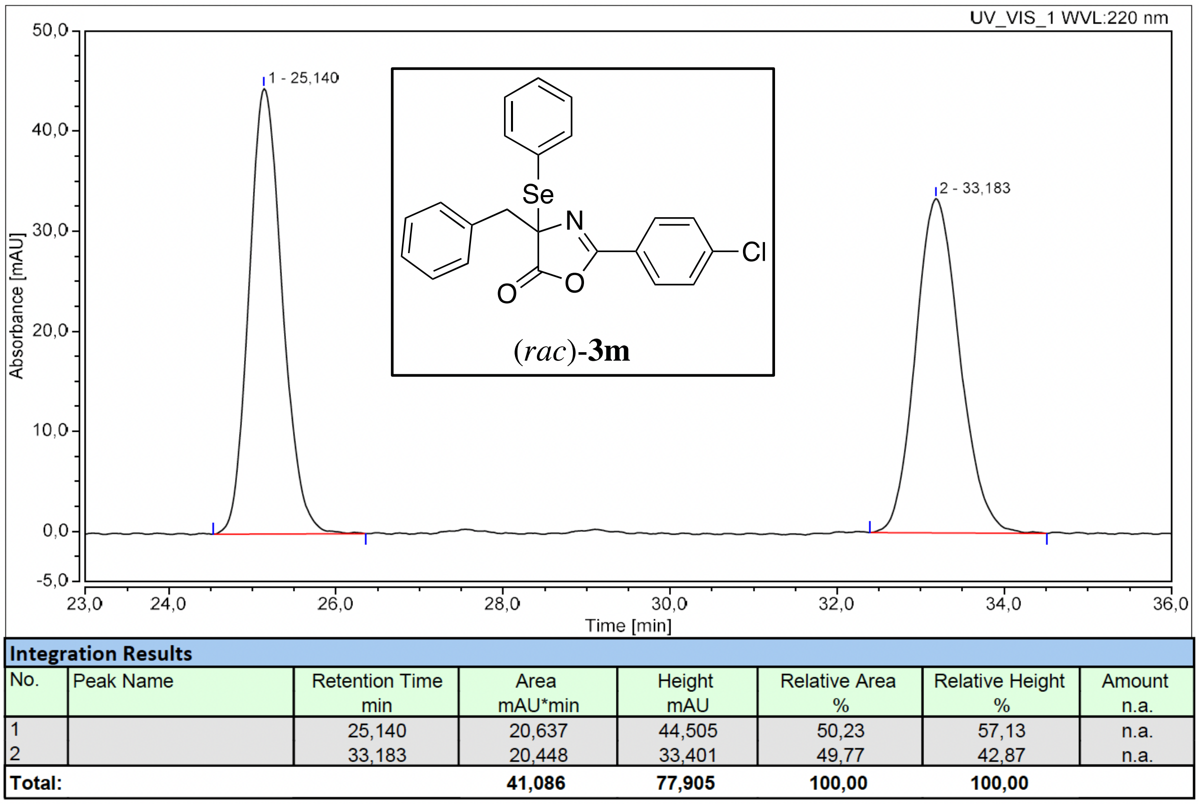

Integration Results

No.	Peak Name	Retention Time min	Area mAU*min	Height mAU	Relative Area %	Relative Height %	Amount n.a.
1		25,140	20,637	44,505	50,23	57,13	n.a.
2		33,183	20,448	33,401	49,77	42,87	n.a.
**Total:**			**41,086**	**77,905**	**100,00**	**100,00**	

HPLC traces of **3n** (Chiralpak AD-H, *n*-hexane:*i*-PrOH = 20:1, flow rate 0.7 mL/min, 10 °C, λ = 220 nm)

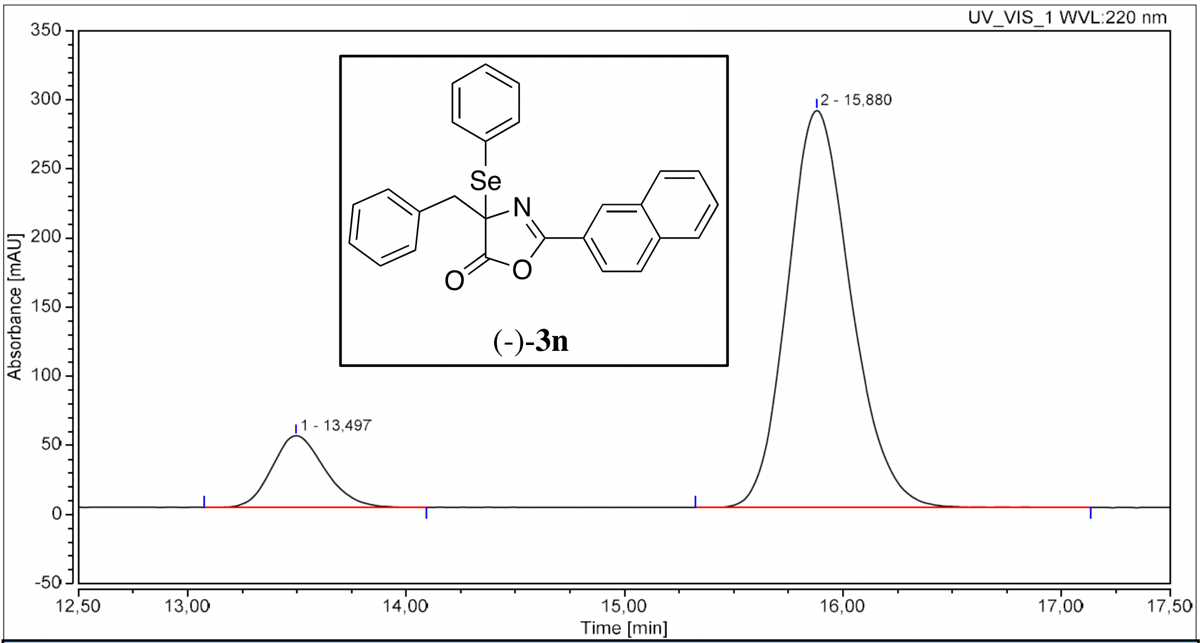

Integration Results							
No.	Peak Name	Retention Time min	Area mAU*min	Height mAU	Relative Area %	Relative Height %	Amount n.a.
1		13,497	14,606	51,935	13,08	15,31	n.a.
2		15,880	97,096	287,316	86,92	84,69	n.a.
**Total:**			**111,702**	**339,251**	**100,00**	**100,00**	

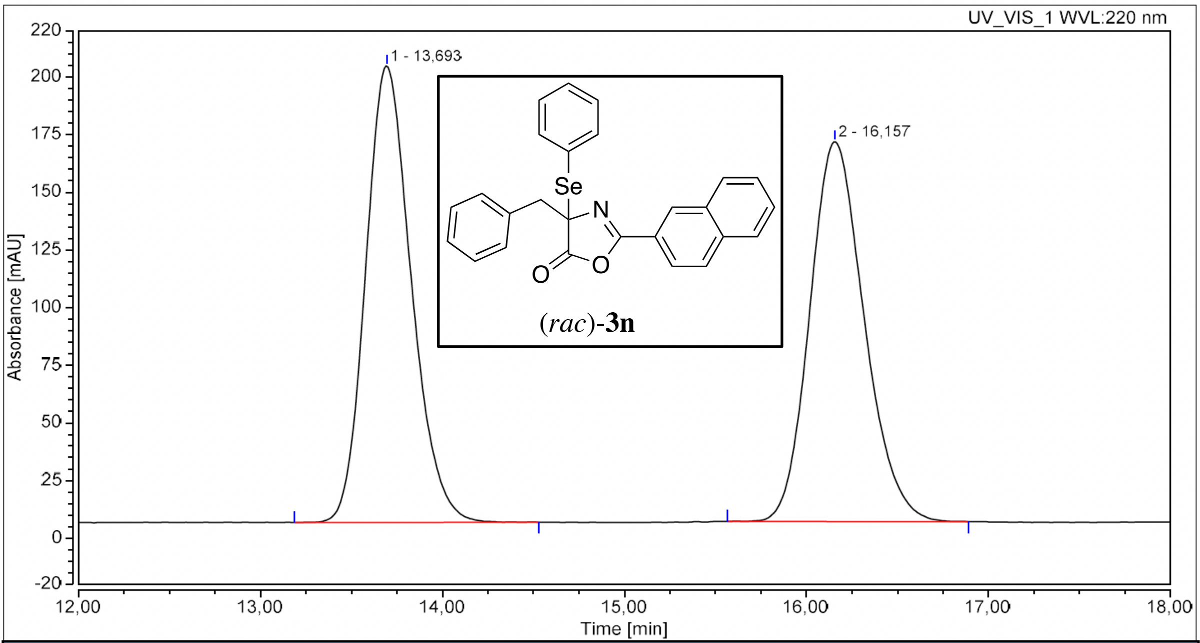

Integration Results							
No.	Peak Name	Retention Time min	Area mAU*min	Height mAU	Relative Area %	Relative Height %	Amount n.a.
1		13,693	57,412	197,897	50,15	54,57	n.a.
2		16,157	57,057	164,739	49,85	45,43	n.a.
**Total:**			**114,469**	**362,636**	**100,00**	**100,00**	

HPLC traces of **3o** (Chiralpak AD-H, *n*-hexane:*i*-PrOH = 20:1, flow rate 0.3 mL/min, 10 °C, λ = 220 nm)

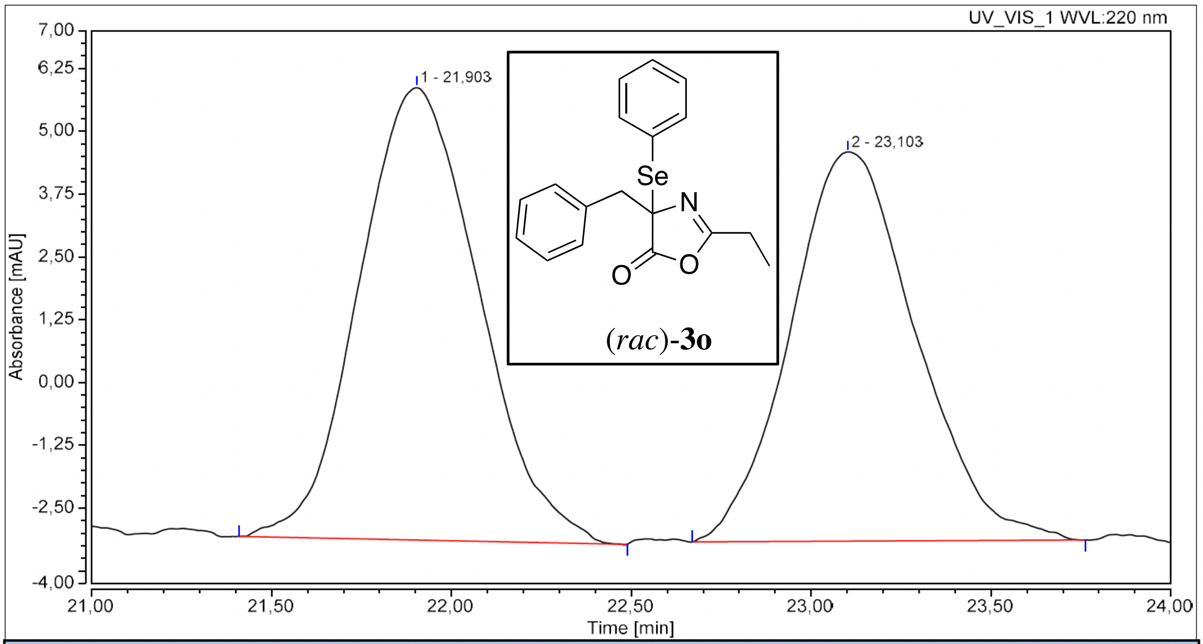

Integration Results							
No.	Peak Name	Retention Time min	Area mAU*min	Height mAU	Relative Area %	Relative Height %	Amount n.a.
1		21,903	3,458	9,000	52,54	53,74	n.a.
2		23,103	3,123	7,746	47,46	46,26	n.a.
**Total:**			**6,581**	**16,745**	**100,00**	**100,00**	

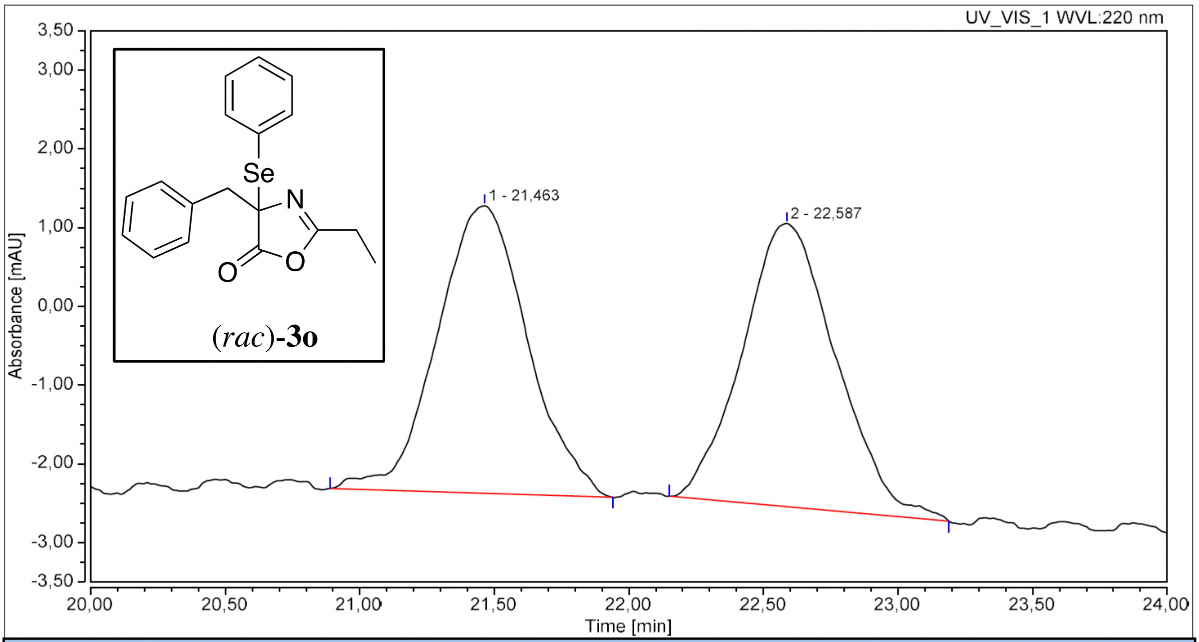

Integration Results							
No.	Peak Name	Retention Time min	Area mAU*min	Height mAU	Relative Area %	Relative Height %	Amount n.a.
1		21,463	1,419	3,649	49,49	50,36	n.a.
2		22,587	1,449	3,597	50,51	49,64	n.a.
**Total:**			**2,868**	**7,246**	**100,00**	**100,00**	

HPLC traces of **3p** (Chiralpak AD-H, *n*-hexane:*i*-PrOH = 20:1, flow rate 0.3 mL/min, 10 °C, λ = 220 nm)

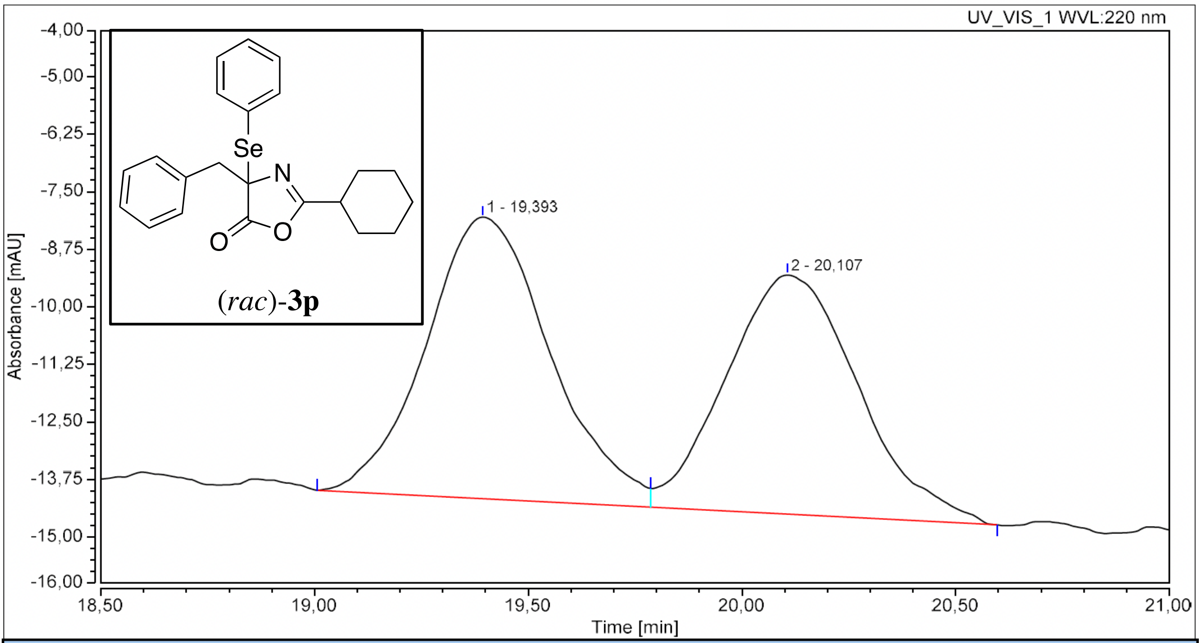

**Integration Results**							
No.	Peak Name	Retention Time min	Area mAU*min	Height mAU	Relative Area %	Relative Height %	Amount n.a.
1		19,393	2,059	6,119	52,55	54,08	n.a.
2		20,107	1,859	5,195	47,45	45,92	n.a.
**Total:**			**3,918**	**11,313**	**100,00**	**100,00**	

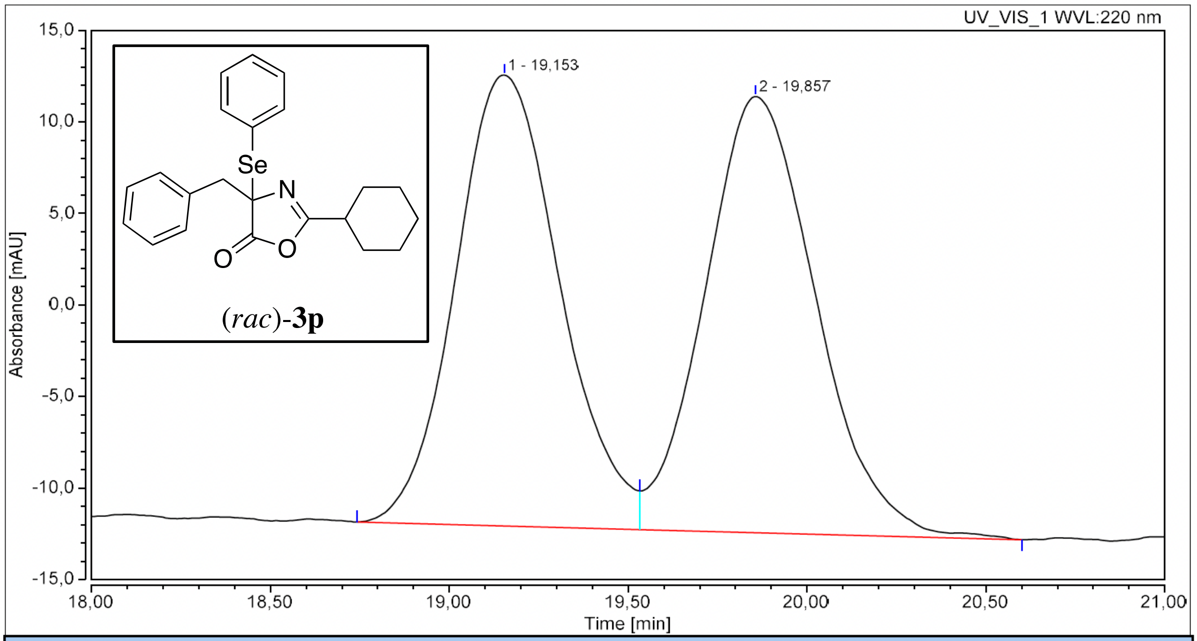

**Integration Results**							
No.	Peak Name	Retention Time min	Area mAU*min	Height mAU	Relative Area %	Relative Height %	Amount n.a.
1		19,153	8,326	24,641	49,27	50,83	n.a.
2		19,857	8,571	23,840	50,73	49,17	n.a.
**Total:**			**16,897**	**48,482**	**100,00**	**100,00**	

HPLC traces of **3q** (Chiralpak AD-H, *n*-hexane:*i*-PrOH = 20:1, flow rate 0.7 mL/min, 10 °C, λ = 220 nm)

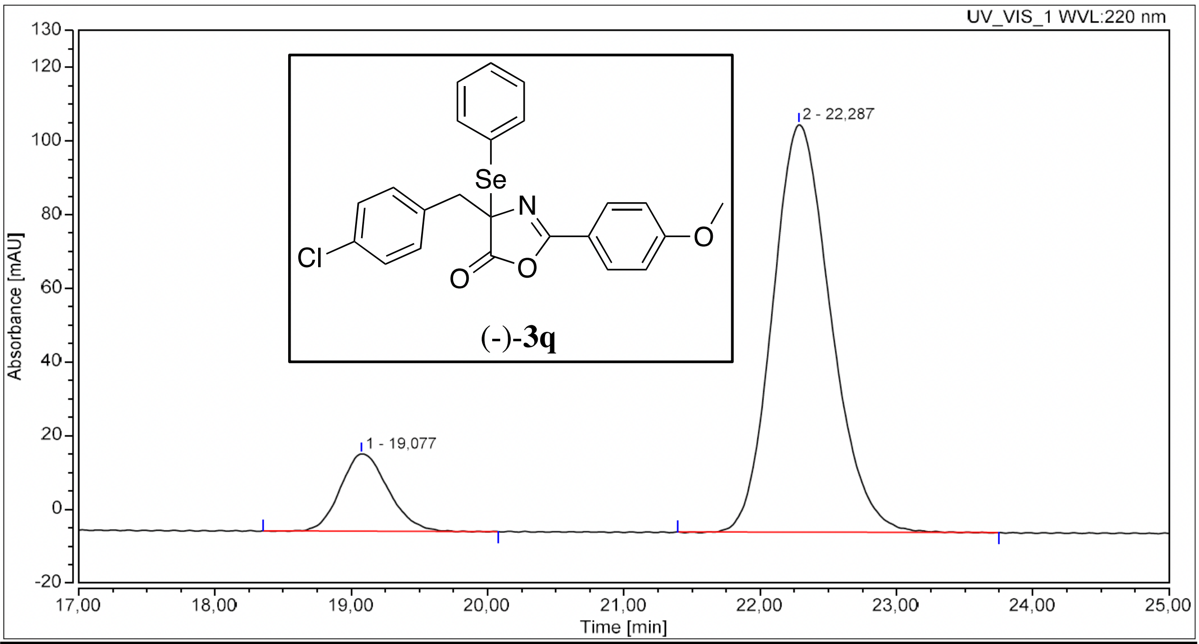

Integration Results							
No.	Peak Name	Retention Time min	Area mAU*min	Height mAU	Relative Area %	Relative Height %	Amount n.a.
1		19,077	8,522	20,974	13,50	15,94	n.a.
2		22,287	54,584	110,590	86,50	84,06	n.a.
**Total:**			**63,106**	**131,565**	**100,00**	**100,00**	

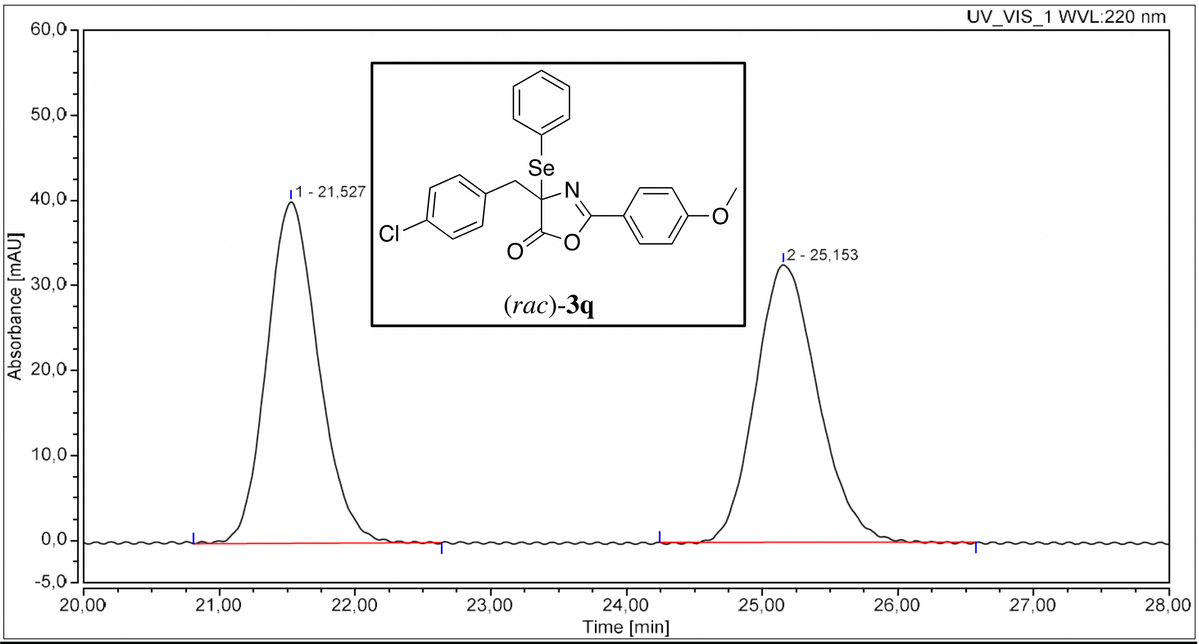

Integration Results							
No.	Peak Name	Retention Time min	Area mAU*min	Height mAU	Relative Area %	Relative Height %	Amount n.a.
1		21,527	17,653	40,156	50,48	55,17	n.a.
2		25,153	17,318	32,632	49,52	44,83	n.a.
**Total:**			**34,971**	**72,787**	**100,00**	**100,00**	

HPLC traces of **3r** (Chiralpak AD-H, *n*-hexane:*i*-PrOH = 20:1, flow rate 1.2 mL/min, 10 °C, λ = 220 nm)

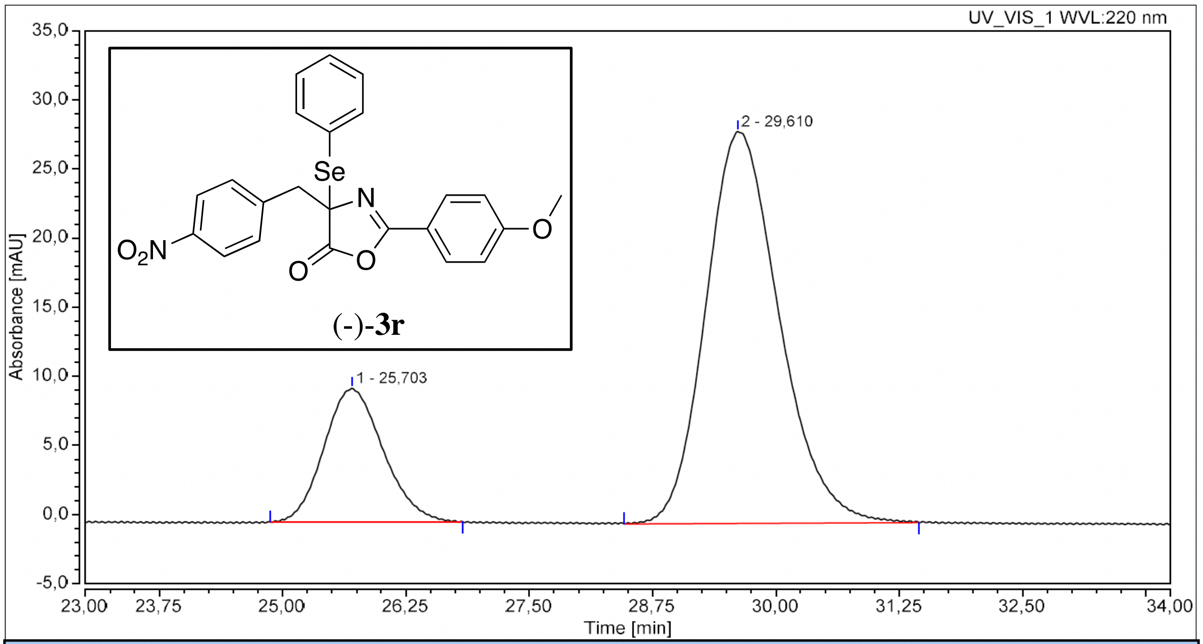

Integration Results							
No.	Peak Name	Retention Time min	Area mAU*min	Height mAU	Relative Area %	Relative Height %	Amount n.a.
1		25,703	6,700	9,661	21,46	25,41	n.a.
2		29,610	24,514	28,360	78,54	74,59	n.a.
**Total:**			**31,214**	**38,021**	**100,00**	**100,00**	

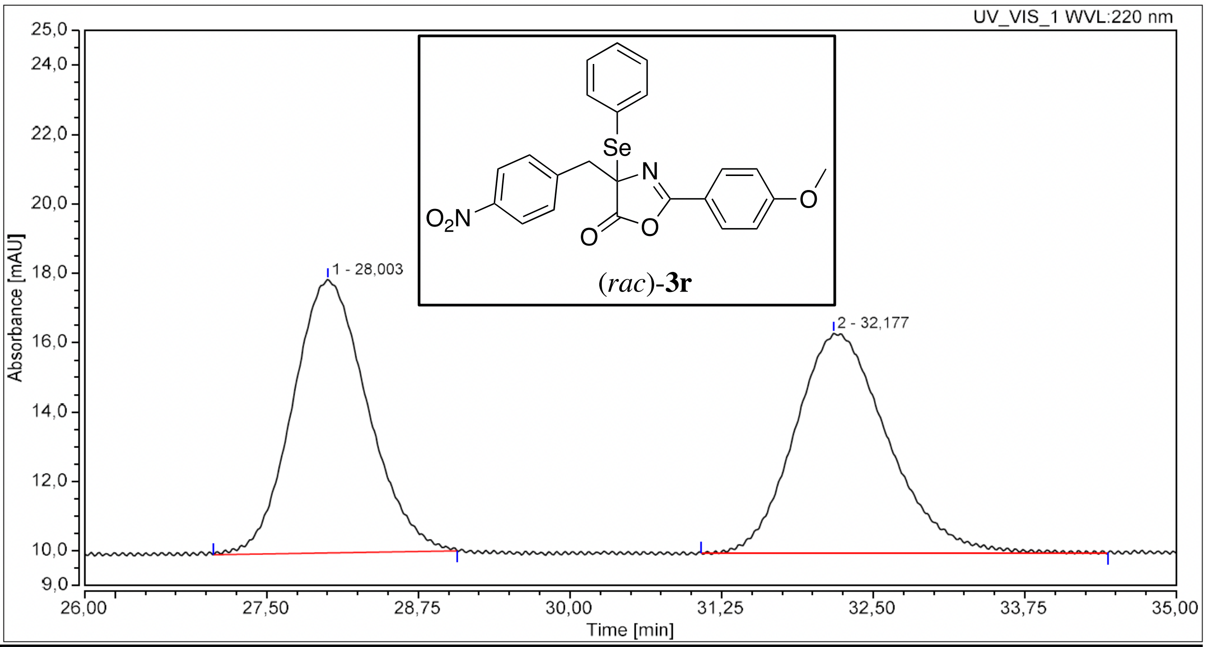

Integration Results							
No.	Peak Name	Retention Time min	Area mAU*min	Height mAU	Relative Area %	Relative Height %	Amount n.a.
1		28,003	5,619	7,883	50,01	55,45	n.a.
2		32,177	5,618	6,332	49,99	44,55	n.a.
**Total:**			**11,238**	**14,215**	**100,00**	**100,00**	

HPLC traces of **3s** (Chiralpak AD-H, *n*-hexane:*i*-PrOH = 20:1, flow rate 0.7 mL/min, 10 °C, λ = 220 nm)

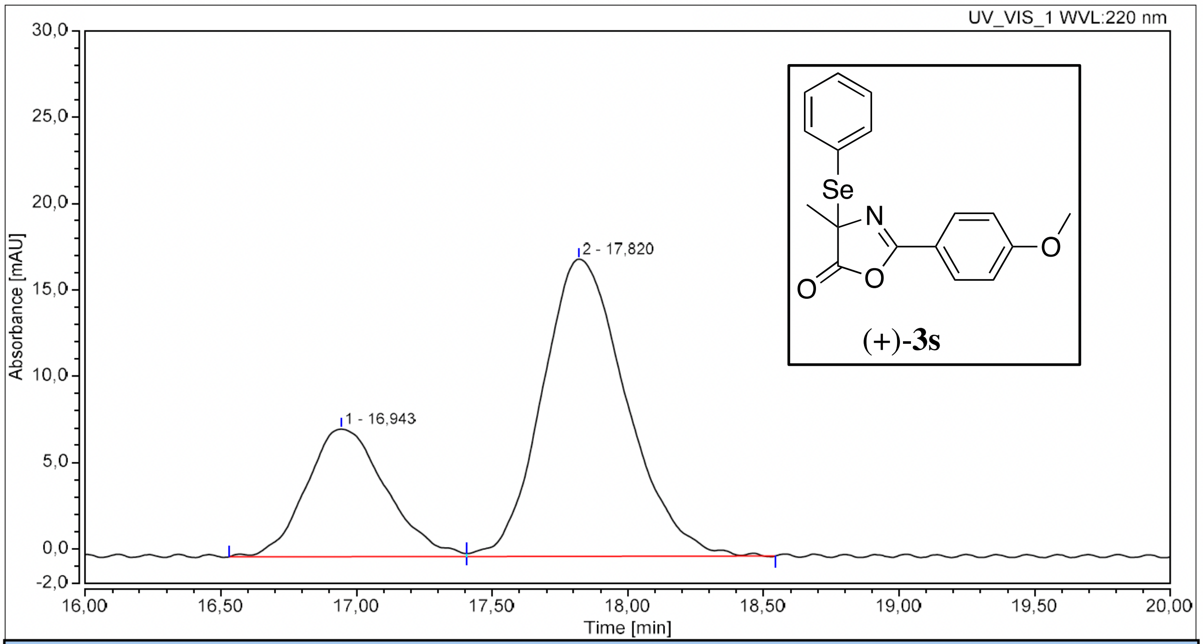

Integration Results							
No.	Peak Name	Retention Time min	Area mAU*min	Height mAU	Relative Area %	Relative Height %	Amount n.a.
1		16,943	2,521	7,377	28,96	30,00	n.a.
2		17,820	6,183	17,216	71,04	70,00	n.a.
**Total:**			**8,704**	**24,593**	**100,00**	**100,00**	

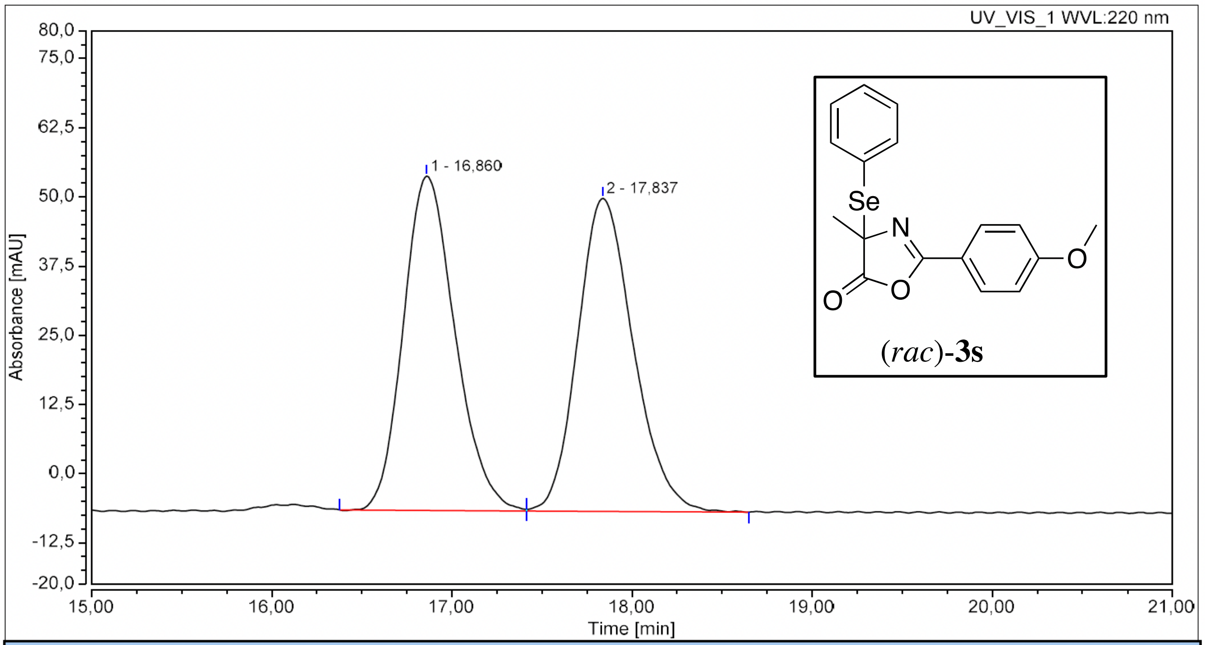

Integration Results							
No.	Peak Name	Retention Time min	Area mAU*min	Height mAU	Relative Area %	Relative Height %	Amount n.a.
1		16,860	20,093	60,401	49,84	51,65	n.a.
2		17,837	20,222	56,543	50,16	48,35	n.a.
**Total:**			**40,316**	**116,945**	**100,00**	**100,00**	

HPLC traces of **3t** (Chiralpak AD-H, *n*-hexane:*i*-PrOH = 20:1, flow rate 0.7 mL/min, 10 °C, λ = 220 nm)

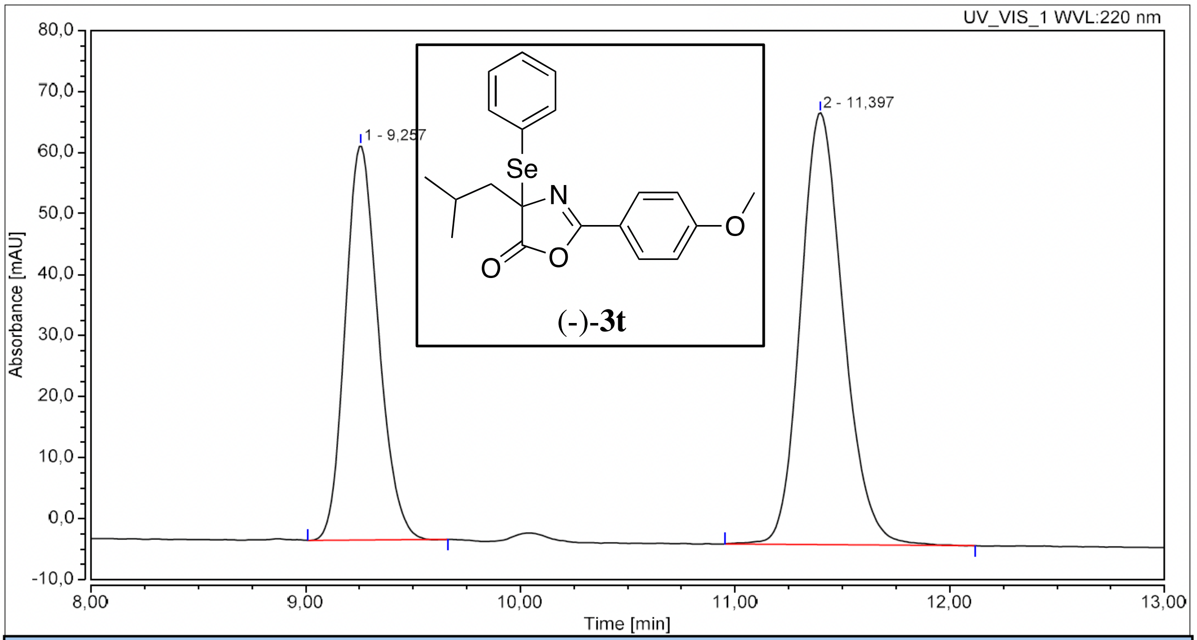

Integration Results

No.	Peak Name	Retention Time min	Area mAU*min	Height mAU	Relative Area %	Relative Height %	Amount n.a.
1		9,257	11,549	64,640	41,36	47,72	n.a.
2		11,397	16,372	70,804	58,64	52,28	n.a.
**Total:**			**27,922**	**135,443**	**100,00**	**100,00**	

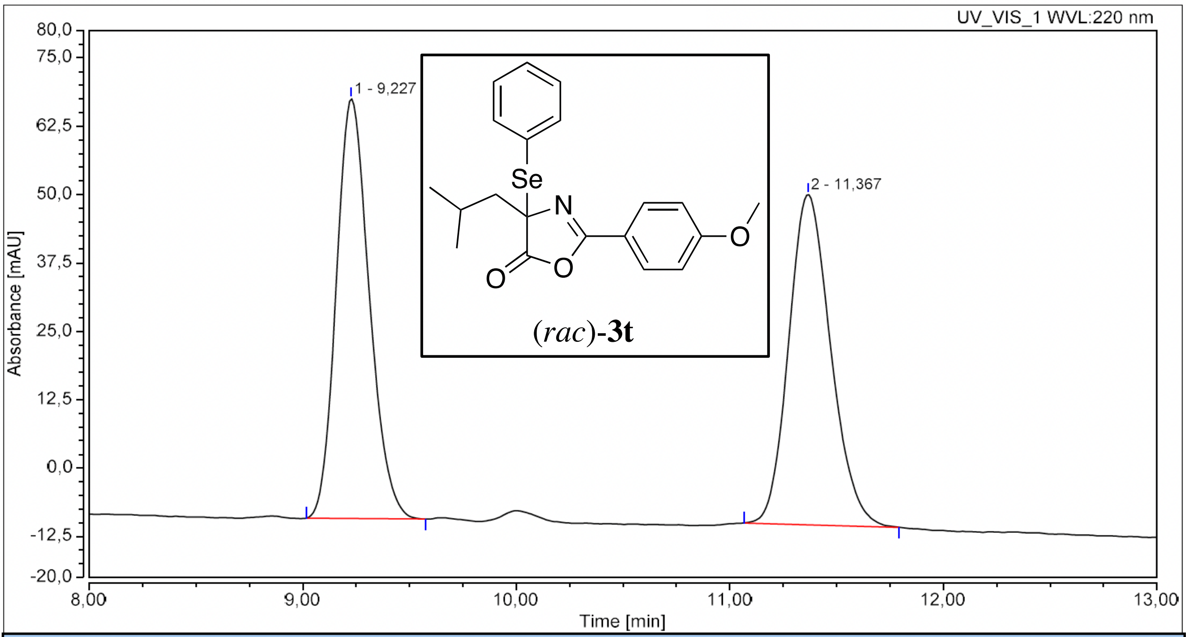

Integration Results

No.	Peak Name	Retention Time min	Area mAU*min	Height mAU	Relative Area %	Relative Height %	Amount n.a.
1		9,227	13,754	76,764	50,01	55,96	n.a.
2		11,367	13,749	60,424	49,99	44,04	n.a.
**Total:**			**27,503**	**137,188**	**100,00**	**100,00**	

HPLC traces of **3u** (Chiralpak AD-H, *n*-hexane:*i*-PrOH = 20:1, flow rate 0.7 mL/min, 10 °C, λ = 220 nm)

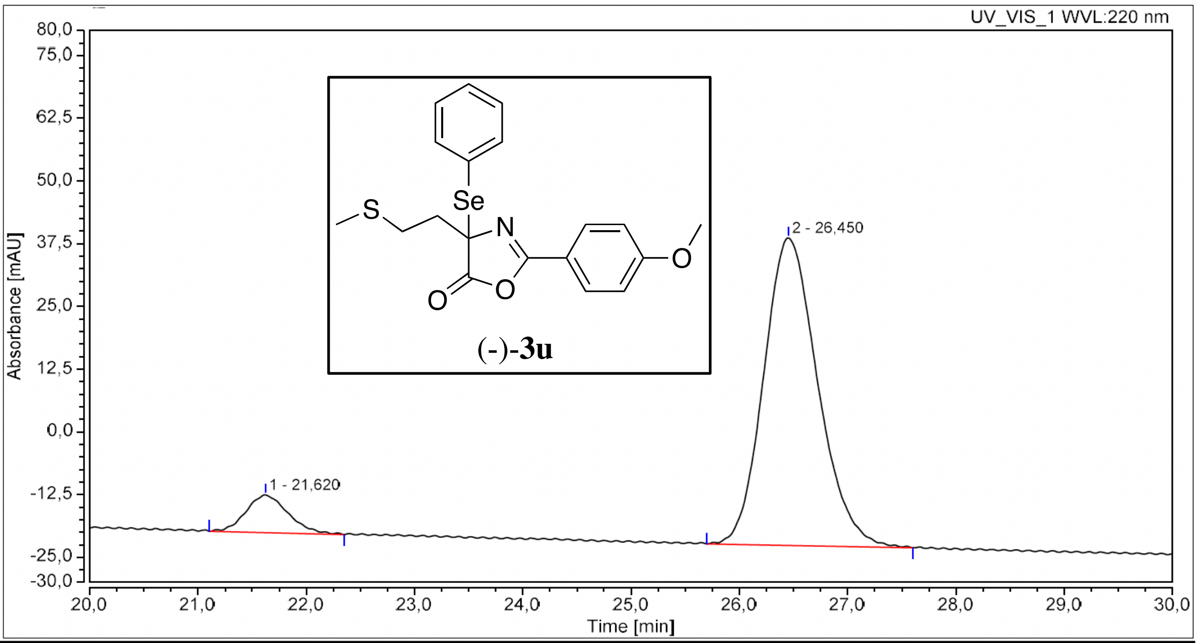

Integration Results							
No.	Peak Name	Retention Time<br>min	Area<br>mAU*min	Height<br>mAU	Relative Area<br>%	Relative Height<br>%	Amount<br>n.a.
1		21,620	3,374	7,523	8,70	10,93	n.a.
2		26,450	35,418	61,311	91,30	89,07	n.a.
**Total:**			**38,792**	**68,834**	**100,00**	**100,00**	

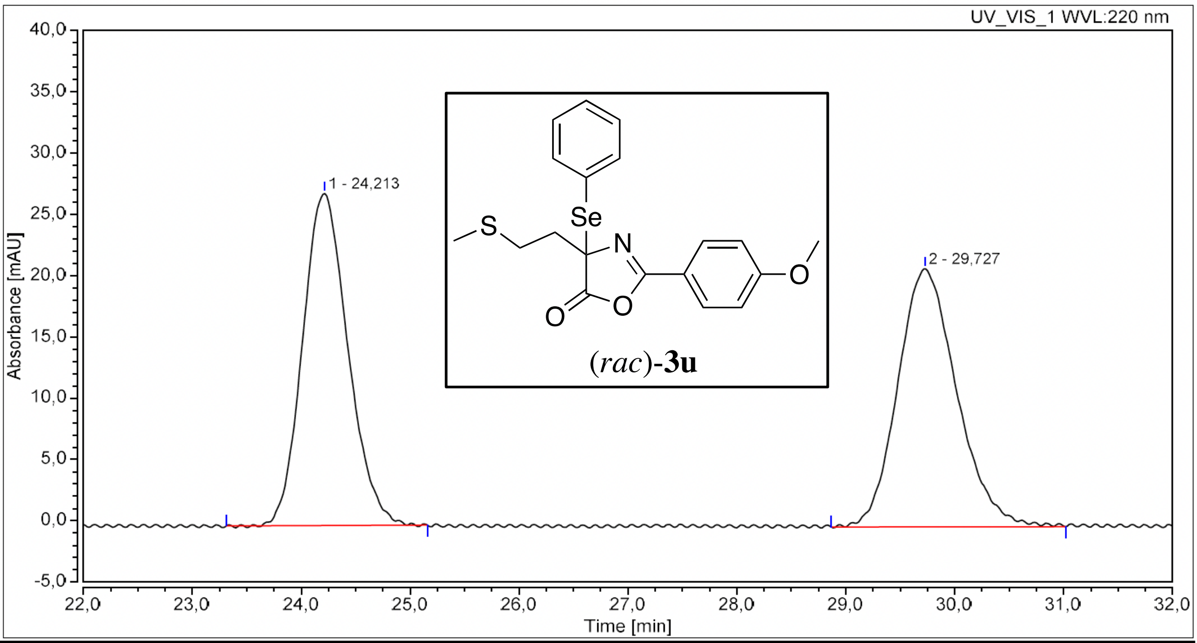

Integration Results							
No.	Peak Name	Retention Time<br>min	Area<br>mAU*min	Height<br>mAU	Relative Area<br>%	Relative Height<br>%	Amount<br>n.a.
1		24,213	13,040	27,096	49,74	56,27	n.a.
2		29,727	13,175	21,057	50,26	43,73	n.a.
**Total:**			**26,214**	**48,153**	**100,00**	**100,00**	

HPLC traces of **3v** (YMC Chiral Art Cellulose SB, *n*-hexane:*i*-PrOH = 20:1, flow rate 0.3 mL/min, 10 °C, λ = 270 nm)

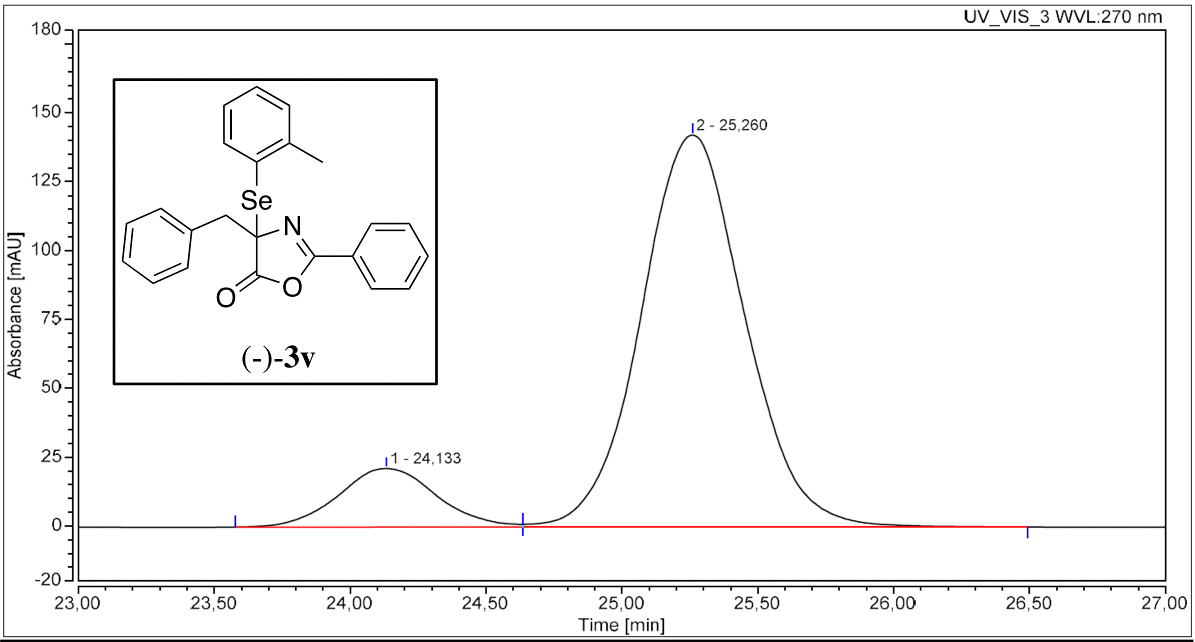

Integration Results							
No.	Peak Name	Retention Time min	Area mAU*min	Height mAU	Relative Area %	Relative Height %	Amount n.a.
1		24,133	8,884	21,240	12,37	12,99	n.a.
2		25,260	62,930	142,288	87,63	87,01	n.a.
**Total:**			**71,814**	**163,528**	**100,00**	**100,00**	

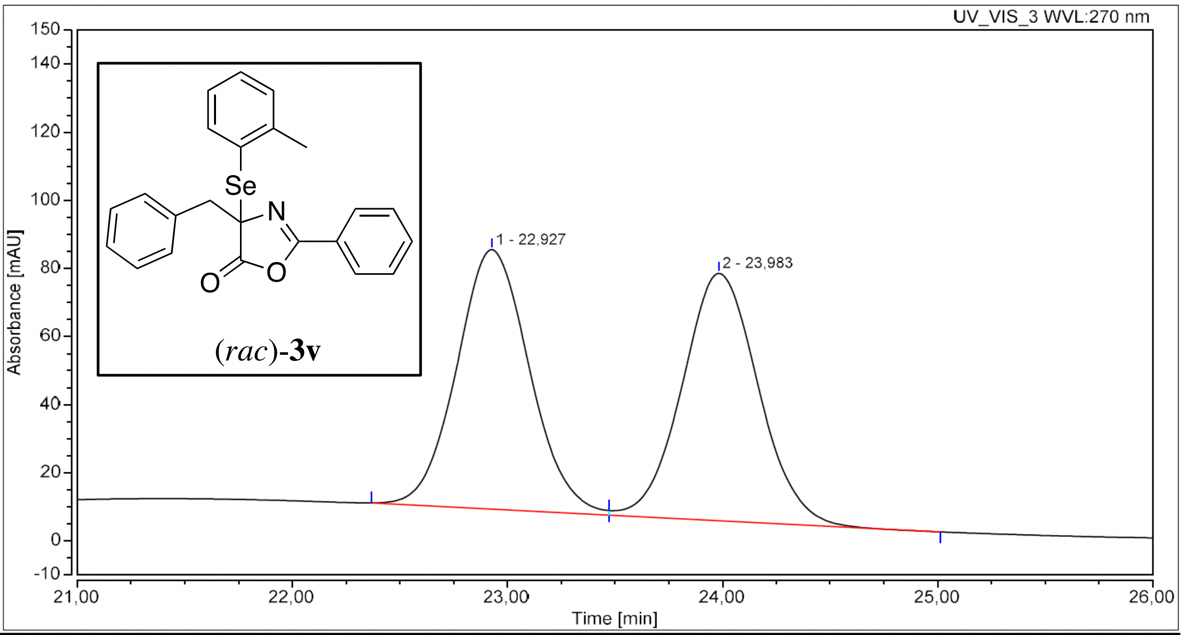

Integration Results							
No.	Peak Name	Retention Time min	Area mAU*min	Height mAU	Relative Area %	Relative Height %	Amount n.a.
1		22,927	29,853	76,220	50,06	51,22	n.a.
2		23,983	29,787	72,598	49,94	48,78	n.a.
**Total:**			**59,640**	**148,817**	**100,00**	**100,00**	

HPLC traces of **3w** (Chiralpak AD-H, *n*-hexane:*i*-PrOH = 20:1, flow rate 0.3 mL/min, 10 °C, λ = 220 nm)

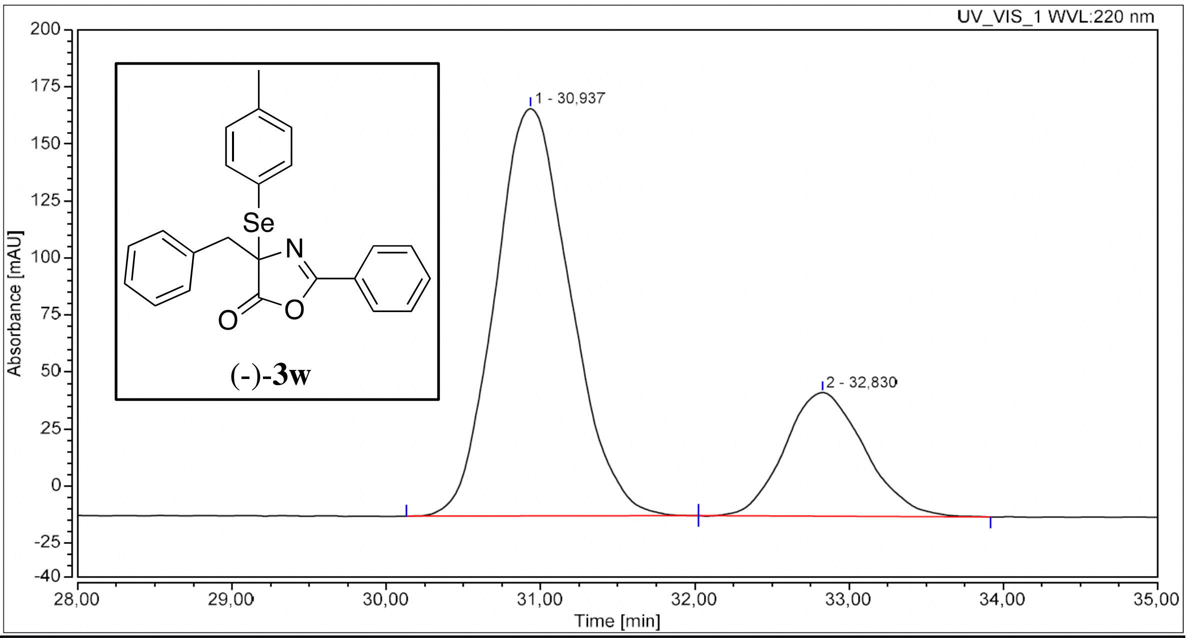

Integration Results							
No.	Peak Name	Retention Time min	Area mAU*min	Height mAU	Relative Area %	Relative Height %	Amount n.a.
1		30,937	101,149	178,550	75,65	76,70	n.a.
2		32,830	32,557	54,245	24,35	23,30	n.a.
**Total:**			**133,706**	**232,795**	**100,00**	**100,00**	

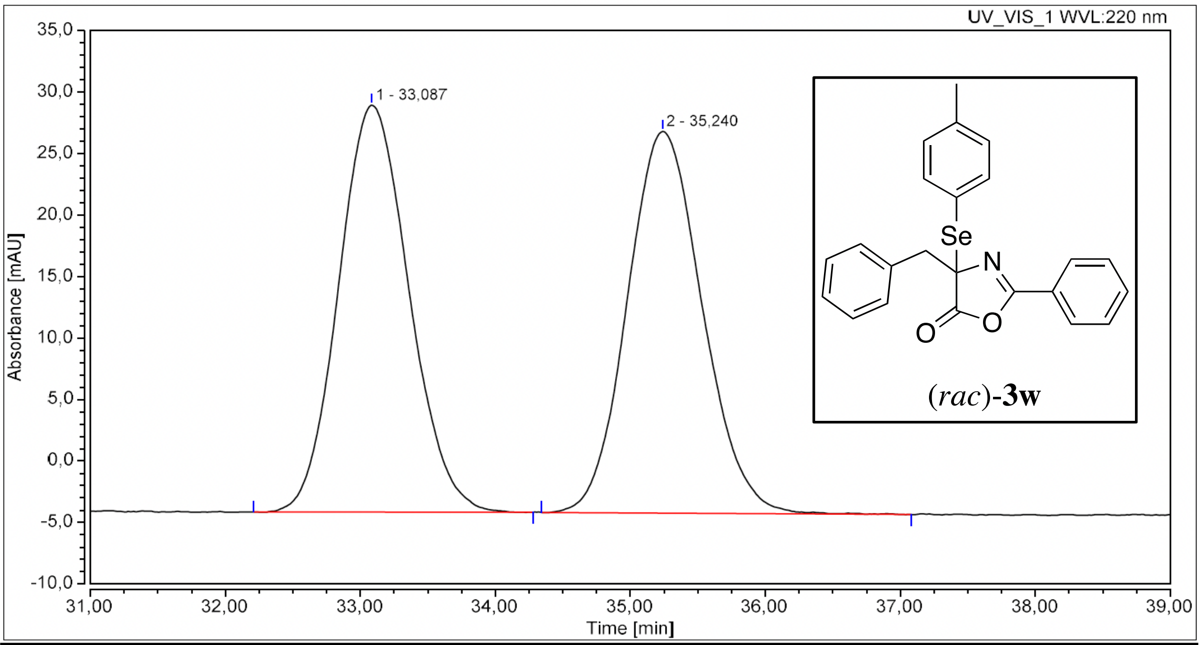

Integration Results							
No.	Peak Name	Retention Time min	Area mAU*min	Height mAU	Relative Area %	Relative Height %	Amount n.a.
1		33,087	19,899	33,075	49,94	51,59	n.a.
2		35,240	19,948	31,031	50,06	48,41	n.a.
**Total:**			**39,847**	**64,106**	**100,00**	**100,00**	

HPLC traces of **3x** (Chiralpak AD-H, *n*-hexane:*i*-PrOH = 20:1, flow rate 0.3 mL/min, 10 °C, λ = 220 nm)

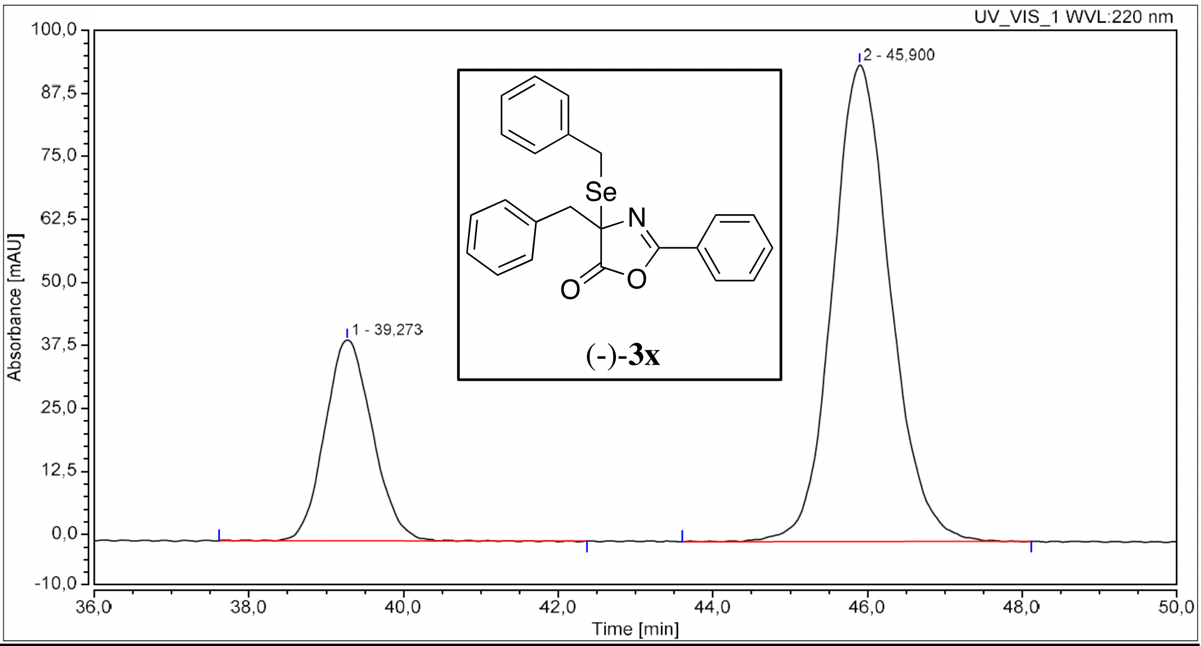

Integration Results							
No.	Peak Name	Retention Time min	Area mAU*min	Height mAU	Relative Area %	Relative Height %	Amount n.a.
1		39,273	28,368	39,823	25,71	29,63	n.a.
2		45,900	81,976	94,563	74,29	70,37	n.a.
**Total:**			**110,344**	**134,385**	**100,00**	**100,00**	

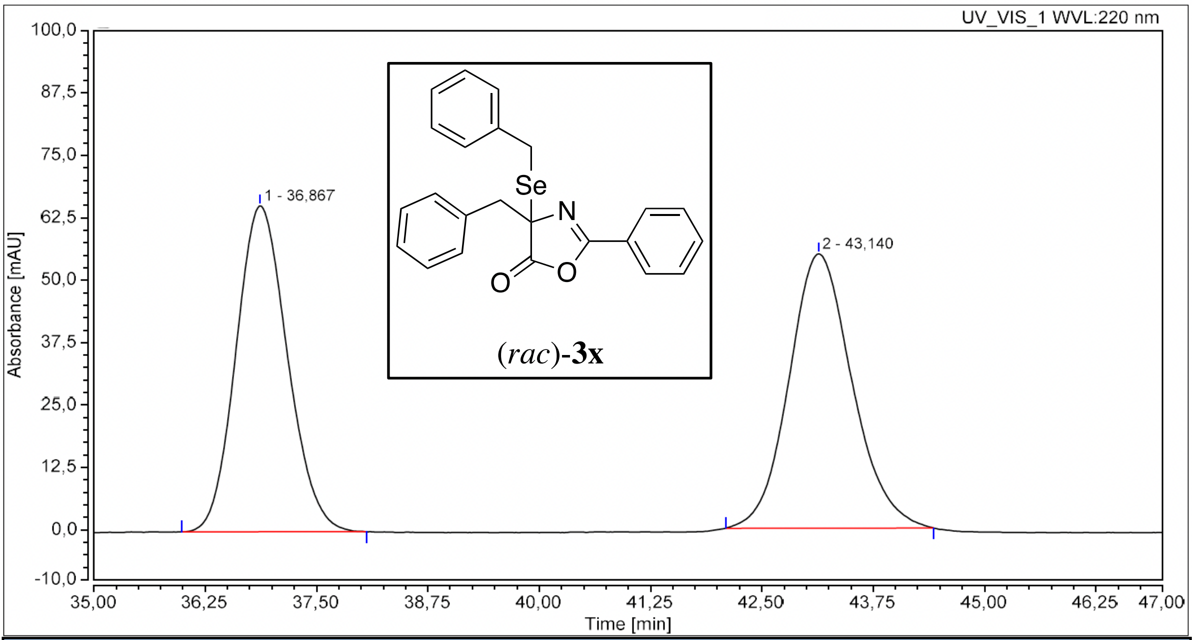

Integration Results							
No.	Peak Name	Retention Time min	Area mAU*min	Height mAU	Relative Area %	Relative Height %	Amount n.a.
1		36,867	43,568	65,307	49,14	54,31	n.a.
2		43,140	45,088	54,939	50,86	45,69	n.a.
**Total:**			**88,657**	**120,245**	**100,00**	**100,00**	

HPLC traces of **4a** (Chiralpak AD-H, *n*-hexane:*i*-PrOH = 20:1, flow rate 1.0 mL/min, 20 °C, λ = 254 nm)

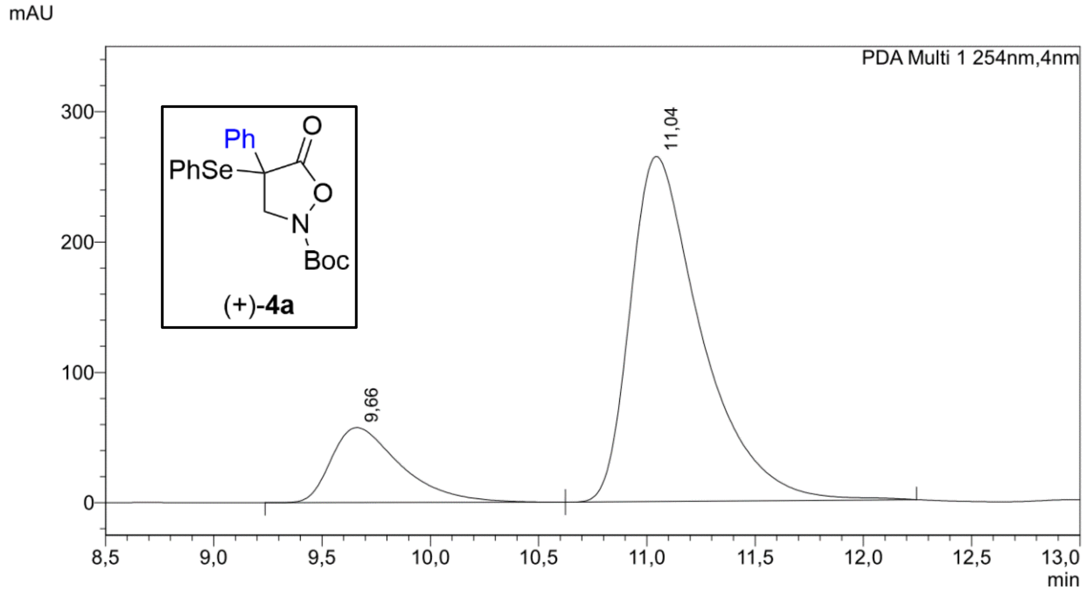

Peak Table

PDA Ch1 254nm

Peak#	Ret. Time	Area	Area%
1	9,66	1282010	17,45
2	11,04	6065478	82,55
Total		7347487	100,00

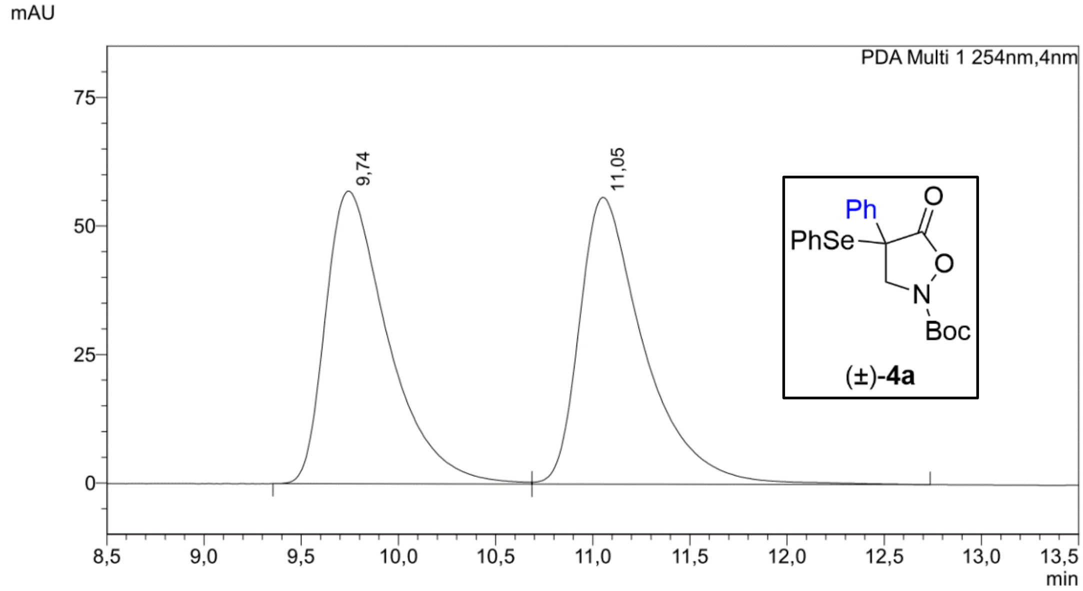

Peak Table

PDA Ch1 254nm

Peak#	Ret. Time	Area	Area%
1	9,74	1277340	49,66
2	11,05	1294957	50,34
Total		2572297	100,00

HPLC traces of **4b** (Chiralpak AD-H, *n*-hexane:*i*-PrOH = 20:1, flow rate 1.0 mL/min, 20 °C, λ = 254 nm)

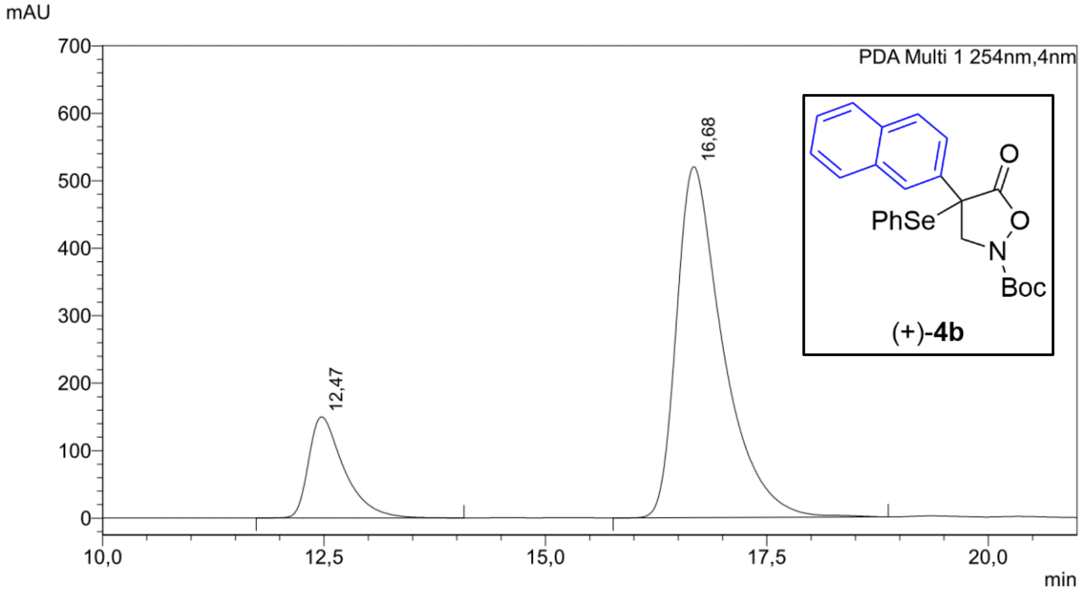

Peak Table

PDA Ch1 254nm

Peak#	Ret. Time	Area	Area%
1	12,47	4169553	17,97
2	16,68	19033478	82,03
Total		23203031	100,00

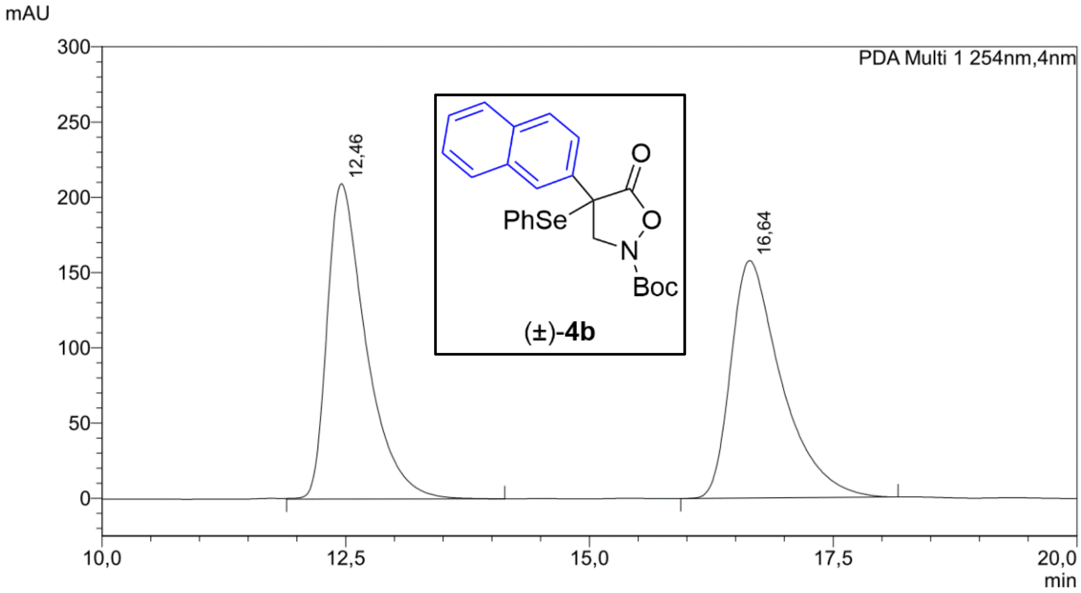

Peak Table

PDA Ch1 254nm

Peak#	Ret. Time	Area	Area%
1	12,46	5822250	50,46
2	16,64	5715966	49,54
Total		11538216	100,00

HPLC traces of **4c** (Chiralpak AD-H, *n*-hexane:*i*-PrOH = 20:1, flow rate 1.0 mL/min, 20 °C, λ = 254 nm)

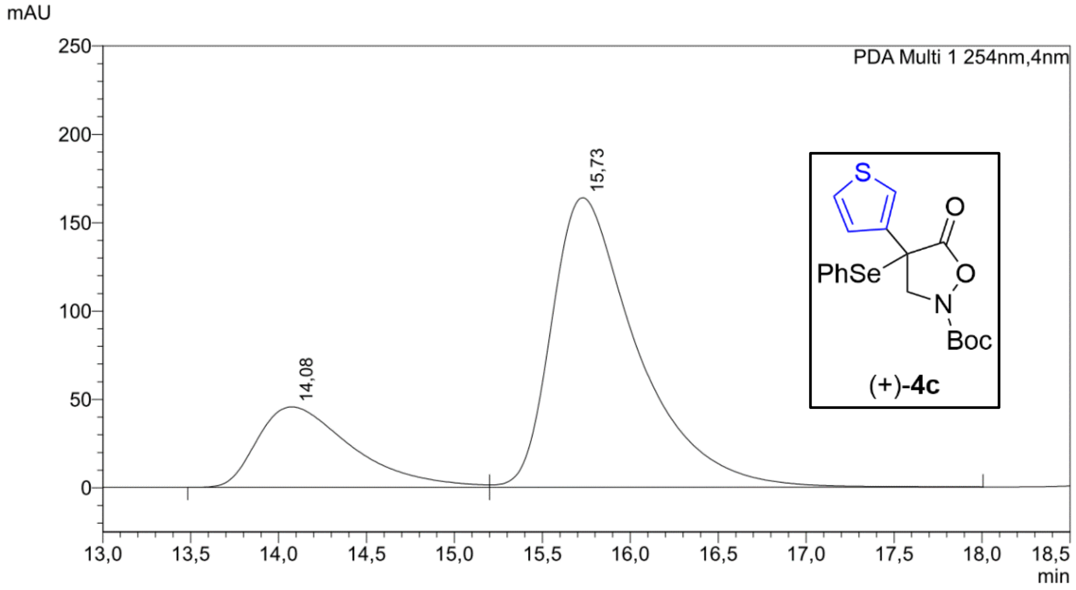

Peak Table

PDA Ch1 254nm

Peak#	Ret. Time	Area	Area%
1	14,08	1708423	23,54
2	15,73	5549356	76,46
Total		7257779	100,00

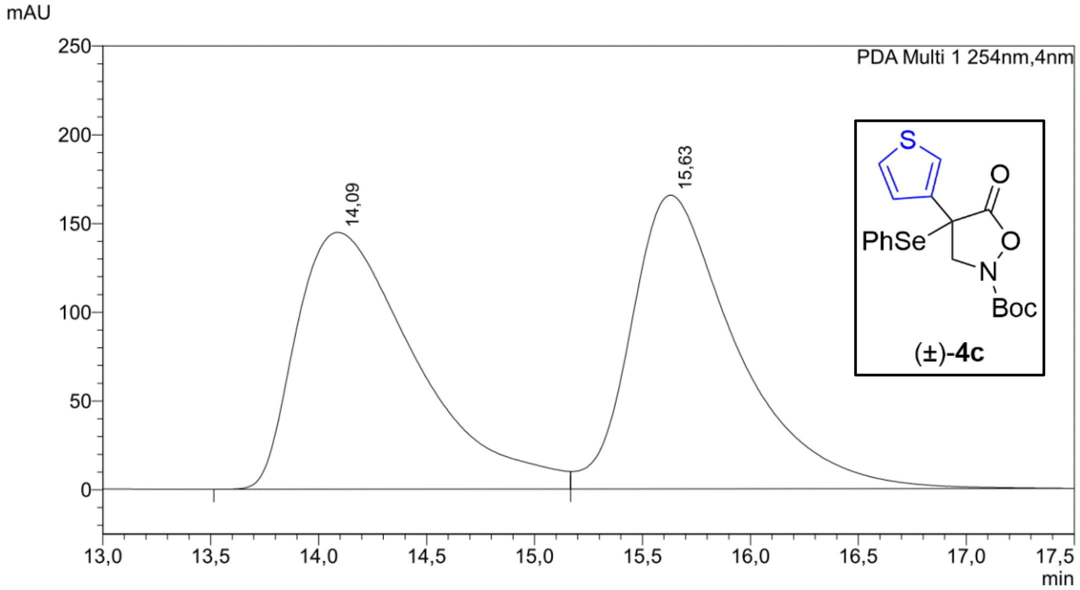

Peak Table

PDA Ch1 254nm

Peak#	Ret. Time	Area	Area%
1	14,09	5615054	49,71
2	15,63	5680643	50,29
Total		11295697	100,00

HPLC traces of **4d** (Chiralpak AD-H, *n*-hexane:*i*-PrOH = 20:1, flow rate 1.0 mL/min, 20 °C, λ = 254 nm)

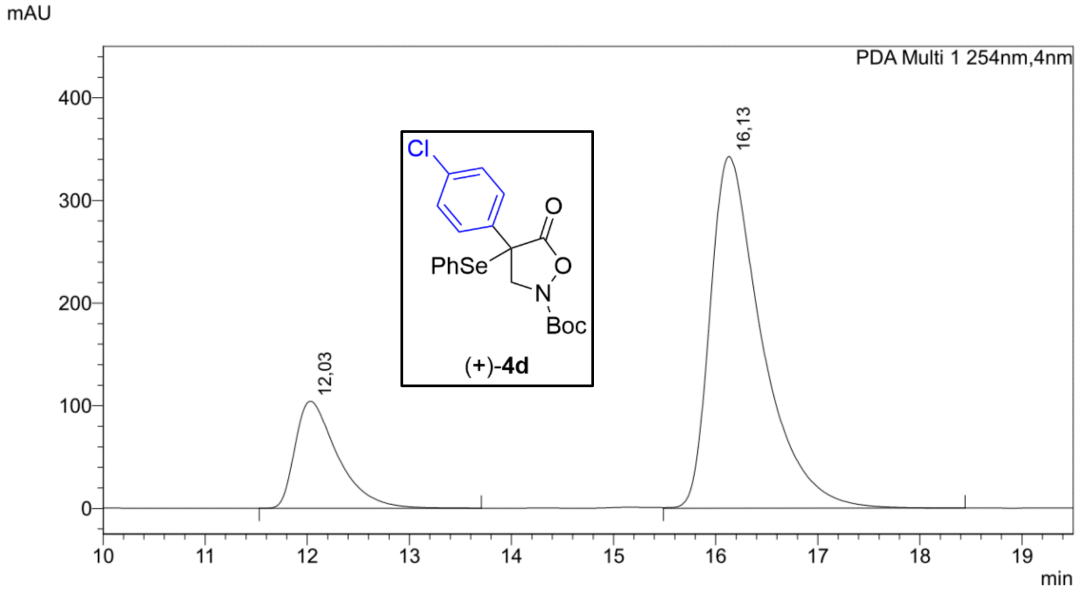

Peak Table

PDA Ch1 254nm

Peak#	Ret. Time	Area	Area%
1	12,03	3020050	20,07
2	16,13	12029062	79,93
Total		15049112	100,00

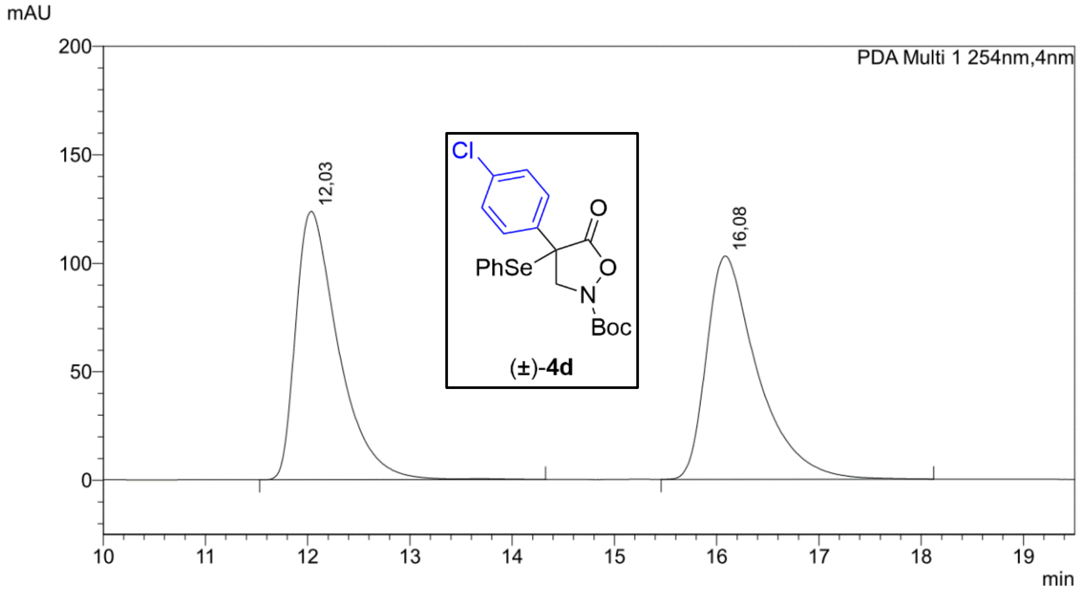

Peak Table

PDA Ch1 254nm

Peak#	Ret. Time	Area	Area%
1	12,03	3589177	50,06
2	16,08	3580893	49,94
Total		7170070	100,00

HPLC traces of **4e** (Chiralpak AD-H, *n*-hexane:*i*-PrOH = 20:1, flow rate 1.0 mL/min, 20 °C, λ = 254 nm)

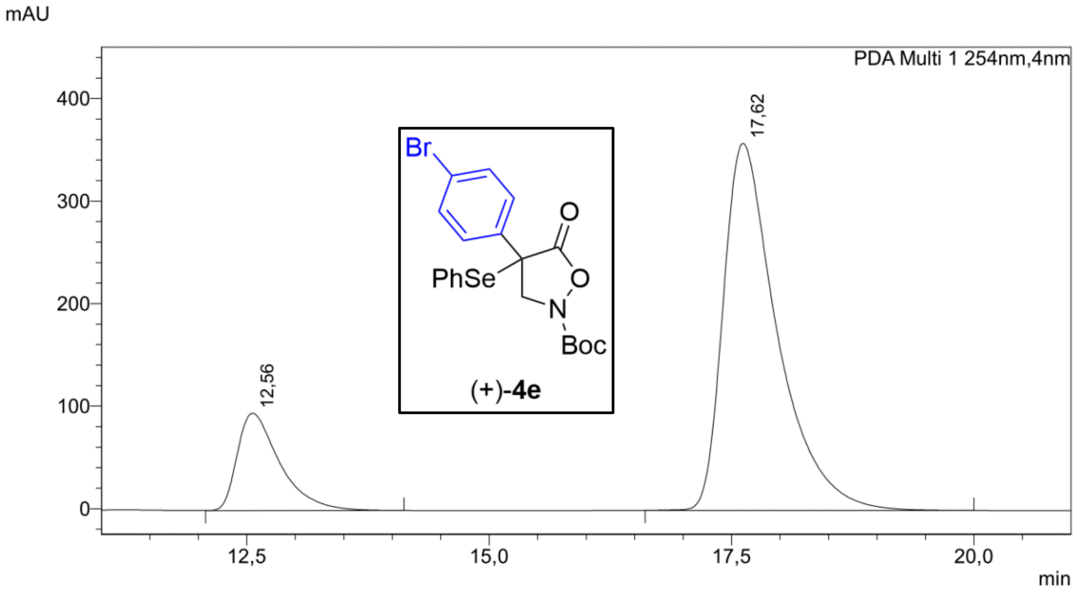

Peak Table

PDA Ch1 254nm

Peak#	Ret. Time	Area	Area%
1	12,56	2901606	17,29
2	17,62	13884221	82,71
Total		16785827	100,00

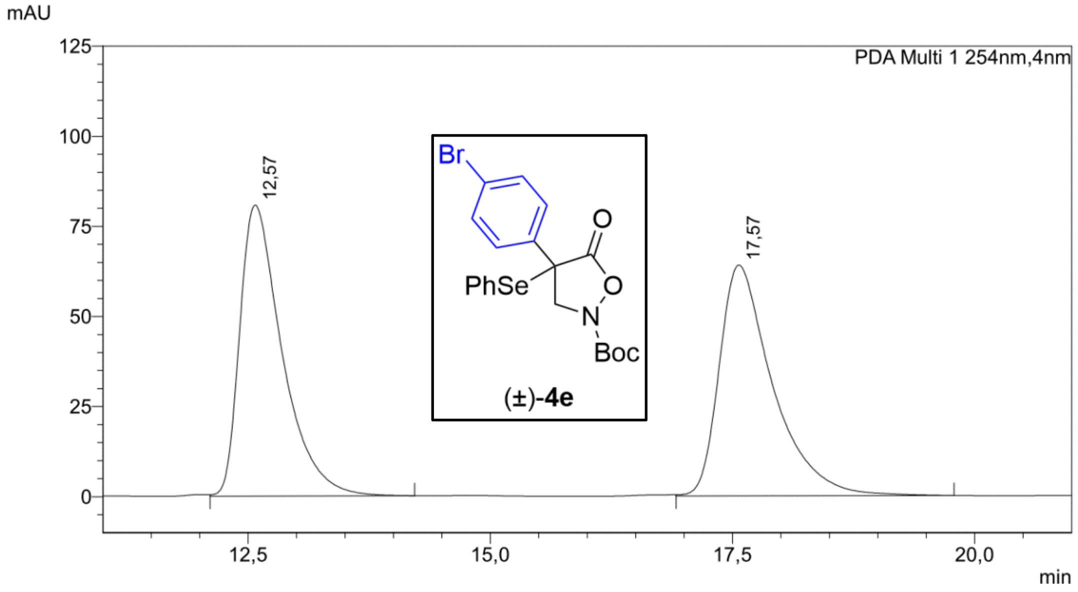

Peak Table

PDA Ch1 254nm

Peak#	Ret. Time	Area	Area%
1	12,57	2453924	49,97
2	17,57	2457074	50,03
Total		4910998	100,00

HPLC traces of **4f** (Chiralpak AD-H, *n*-hexane:*i*-PrOH = 20:1, flow rate 1.0 mL/min, 20 °C, λ = 254 nm)

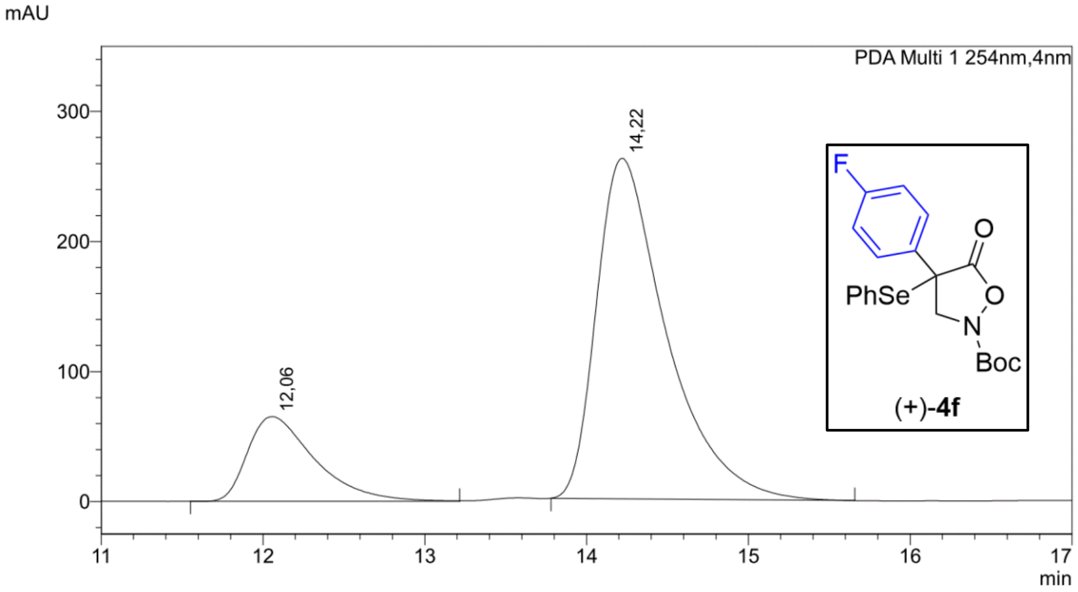

Peak Table

PDA Ch1 254nm

Peak#	Ret. Time	Area	Area%
1	12,06	1879654	19,26
2	14,22	7878156	80,74
Total		9757810	100,00

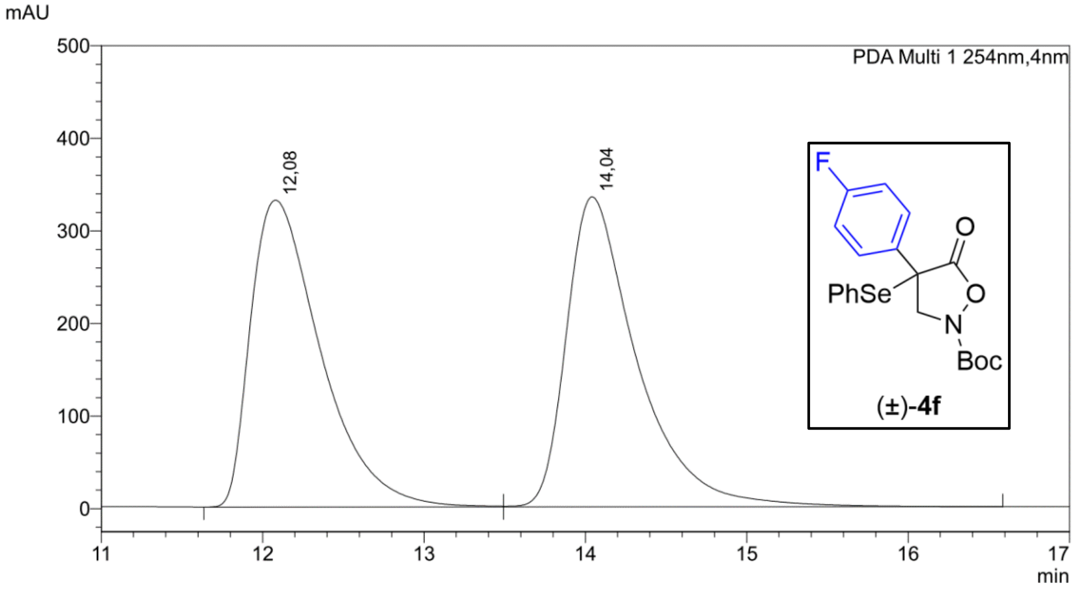

Peak Table

PDA Ch1 254nm

Peak#	Ret. Time	Area	Area%
1	12,08	10007146	49,53
2	14,04	10198474	50,47
Total		20205620	100,00

HPLC traces of **4g** (Chiralpak AD-H, *n*-hexane:*i*-PrOH = 20:1, flow rate 1.0 mL/min, 20 °C, λ = 254 nm)

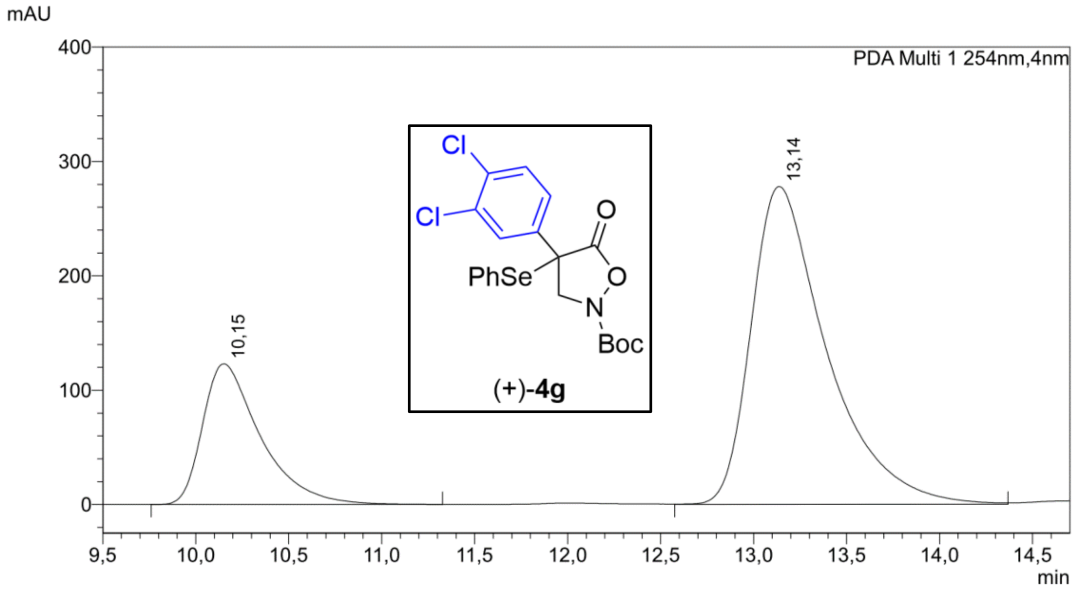

Peak Table

PDA Ch1 254nm

Peak#	Ret. Time	Area	Area%
1	10,15	2654198	25,22
2	13,14	7869025	74,78
Total		10523223	100,00

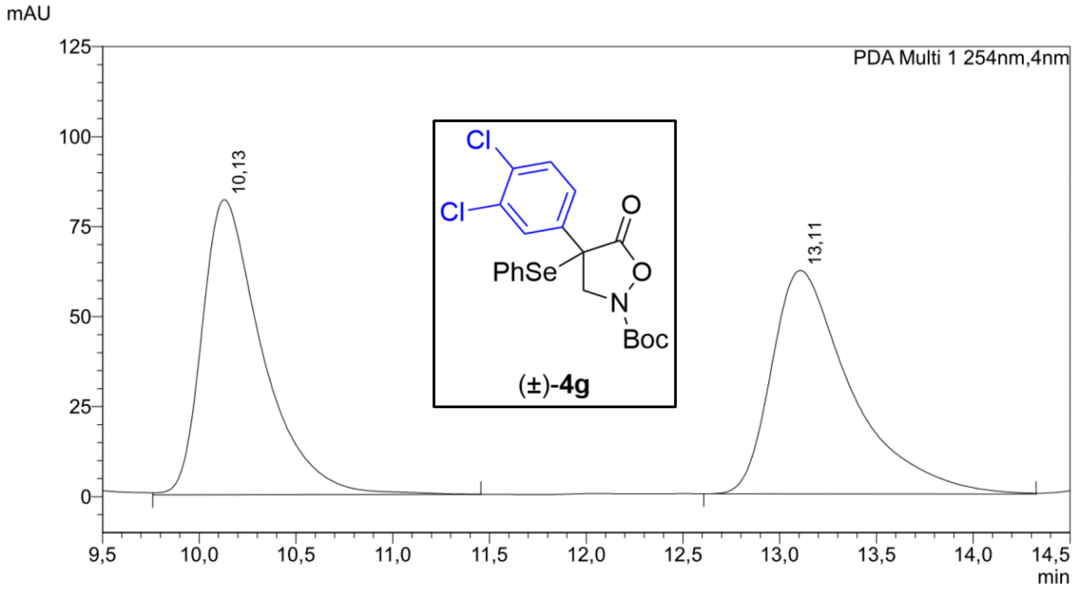

Peak Table

PDA Ch1 254nm

Peak#	Ret. Time	Area	Area%
1	10,13	1775586	49,89
2	13,11	1783726	50,11
Total		3559312	100,00

HPLC traces of **4i** (Chiralpak AD-H, *n*-hexane:*i*-PrOH = 20:1, flow rate 1.0 mL/min, 20 °C, λ = 254 nm)

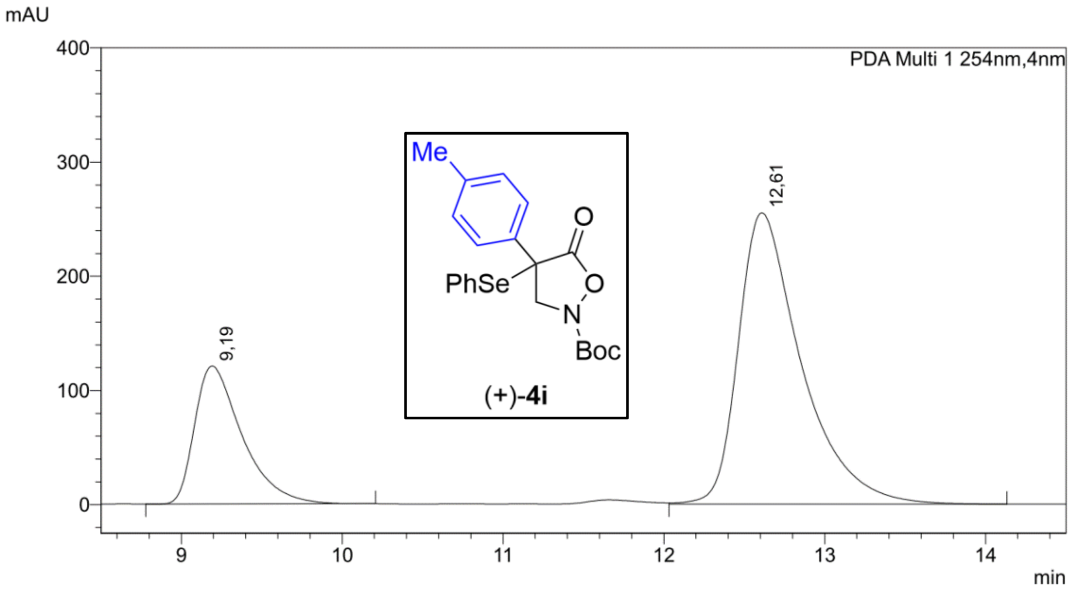

Peak Table

PDA Ch1 254nm

Peak#	Ret. Time	Area	Area%
1	9,19	2471161	26,27
2	12,61	6934762	73,73
Total		9405924	100,00

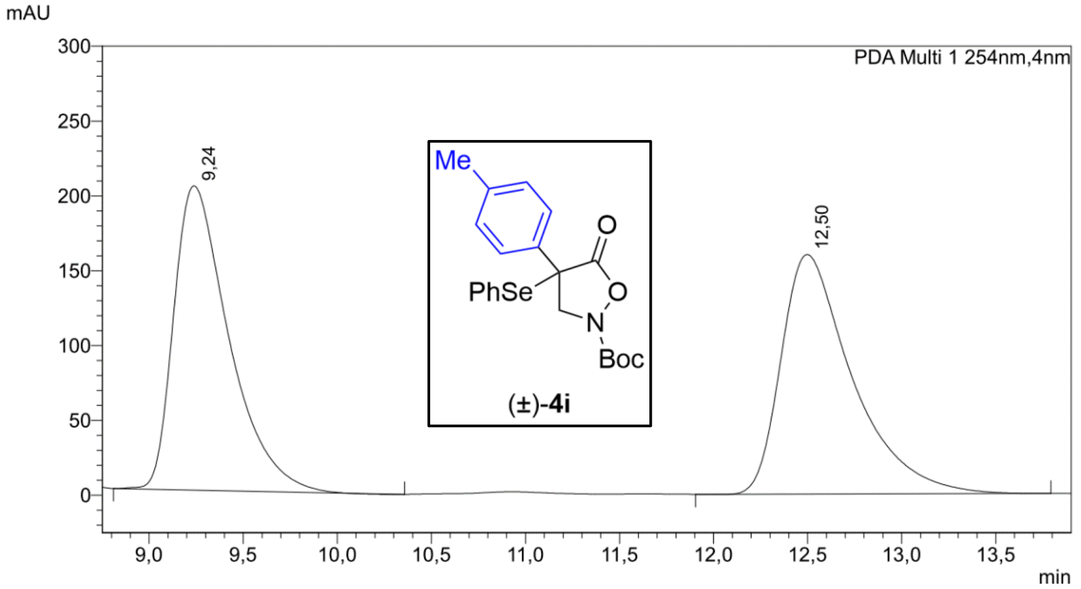

Peak Table

PDA Ch1 254nm

Peak#	Ret. Time	Area	Area%
1	9,24	4142184	49,61
2	12,50	4207357	50,39
Total		8349541	100,00

HPLC traces of **4j** (Chiralpak AD-H, *n*-hexane:*i*-PrOH = 20:1, flow rate 1.0 mL/min, 10 °C, λ = 240 nm)

Chromatogram

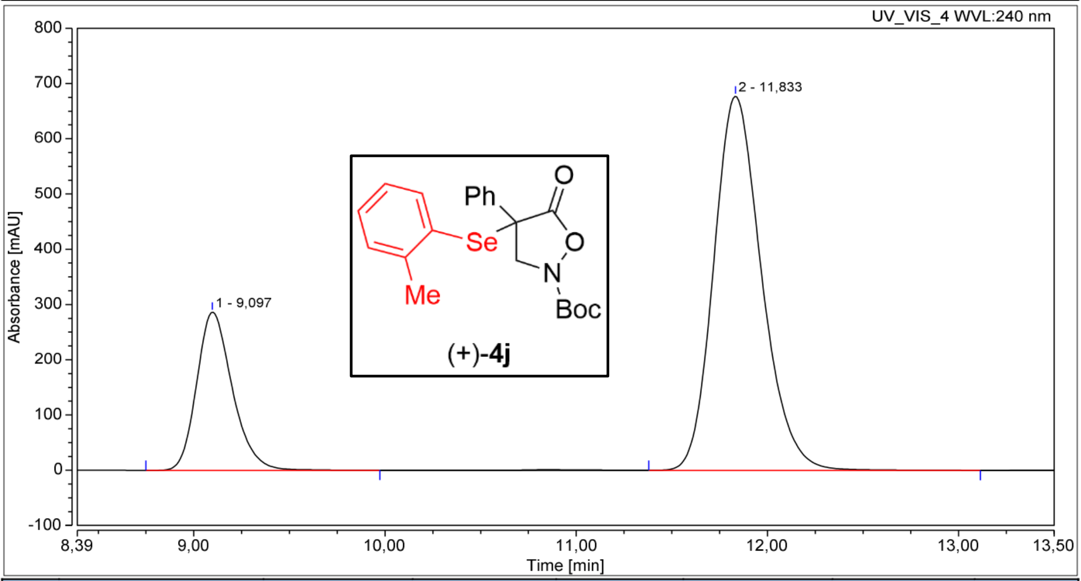

Integration Results

No.	Peak Name	Retention Time min	Area mAU*min	Height mAU	Relative Area %	Relative Height %	Amount n.a.
1		9,097	61,918	286,428	23,99	29,72	n.a.
2		11,833	196,195	677,375	76,01	70,28	n.a.
**Total:**			**258,113**	**963,803**	**100,00**	**100,00**	

Chromatogram

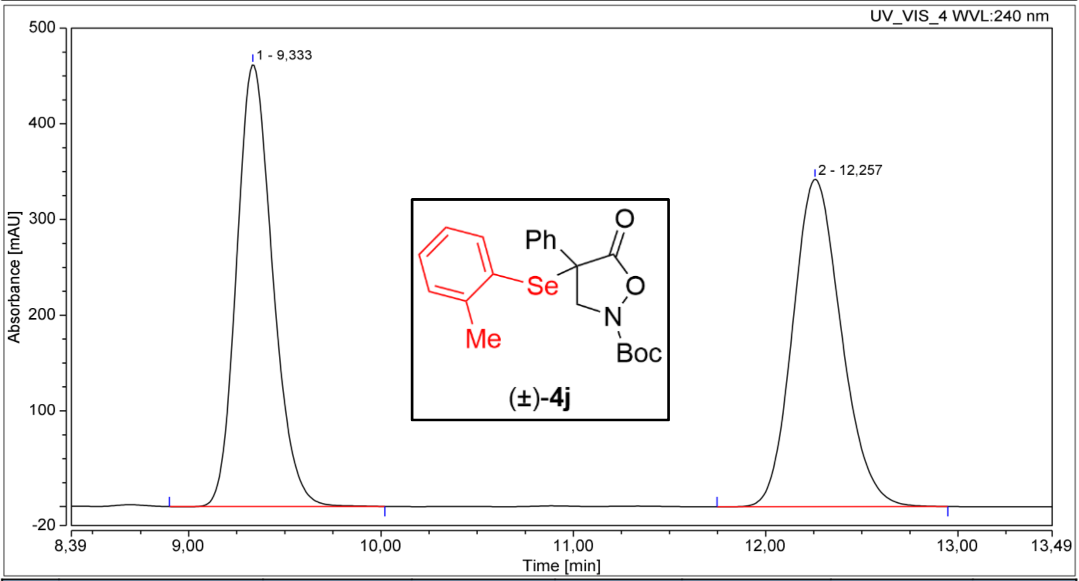

Integration Results

No.	Peak Name	Retention Time min	Area mAU*min	Height mAU	Relative Area %	Relative Height %	Amount n.a.
1		9,333	100,147	462,081	50,01	57,45	n.a.
2		12,257	100,091	342,201	49,99	42,55	n.a.
**Total:**			**200,238**	**804,282**	**100,00**	**100,00**	

HPLC traces of **4k** (Chiralpak AD-H, *n*-hexane:*i*-PrOH = 20:1, flow rate 1.0 mL/min, 10 °C, λ = 240 nm)

Chromatogram

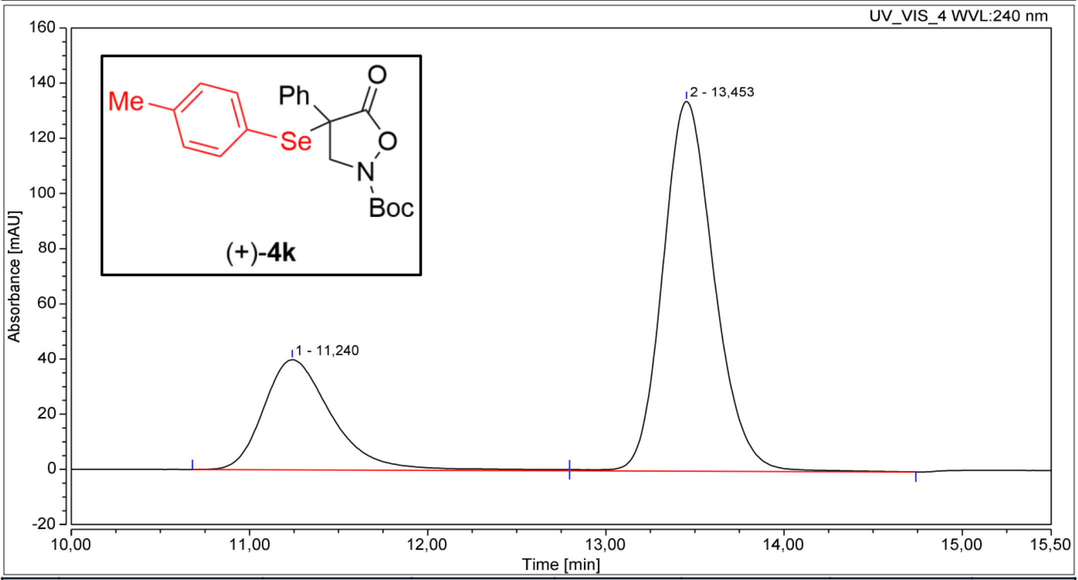

Integration Results

No.	Peak Name	Retention Time min	Area mAU*min	Height mAU	Relative Area %	Relative Height %	Amount n.a.
1		11,240	17,794	39,914	28,45	22,94	n.a.
2		13,453	44,761	134,054	71,55	77,06	n.a.
**Total:**			**62,555**	**173,969**	**100,00**	**100,00**	

Chromatogram

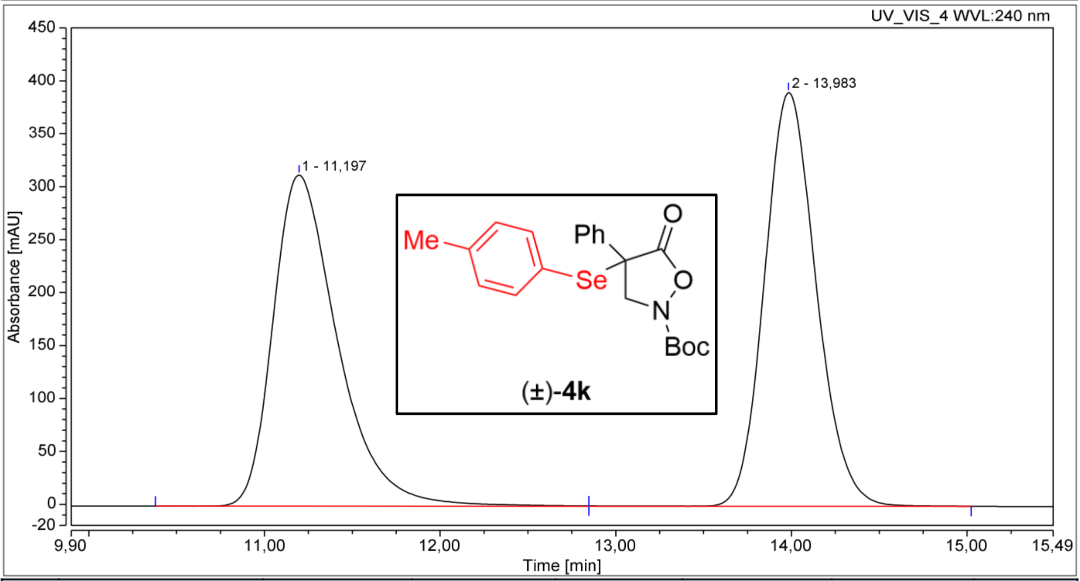

Integration Results

No.	Peak Name	Retention Time min	Area mAU*min	Height mAU	Relative Area %	Relative Height %	Amount n.a.
1		11,197	132,957	312,599	49,95	44,44	n.a.
2		13,983	133,225	390,767	50,05	55,56	n.a.
**Total:**			**266,182**	**703,366**	**100,00**	**100,00**	

HPLC traces of 4l (Chiralpak OD-H, *n*-hexane:*i*-PrOH = 20:1, flow rate 1.0 mL/min, 10 °C, λ = 220 nm)

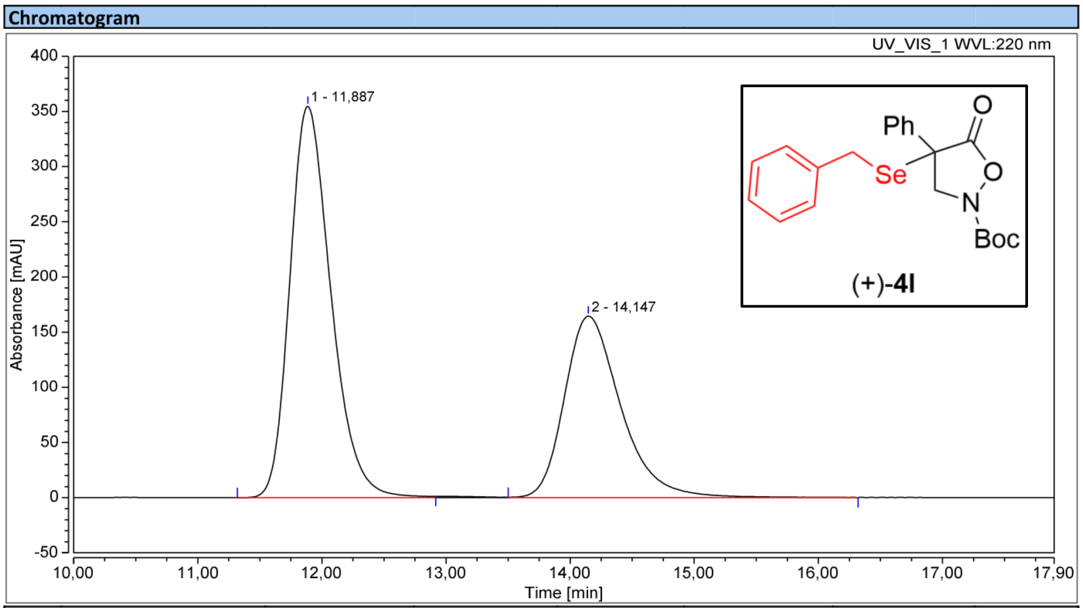

Integration Results							
No.	Peak Name	Retention Time min	Area mAU*min	Height mAU	Relative Area %	Relative Height %	Amount n.a.
1		11,887	136,043	354,763	61,18	68,34	n.a.
2		14,147	86,316	164,360	38,82	31,66	n.a.
**Total:**			**222,359**	**519,124**	**100,00**	**100,00**	

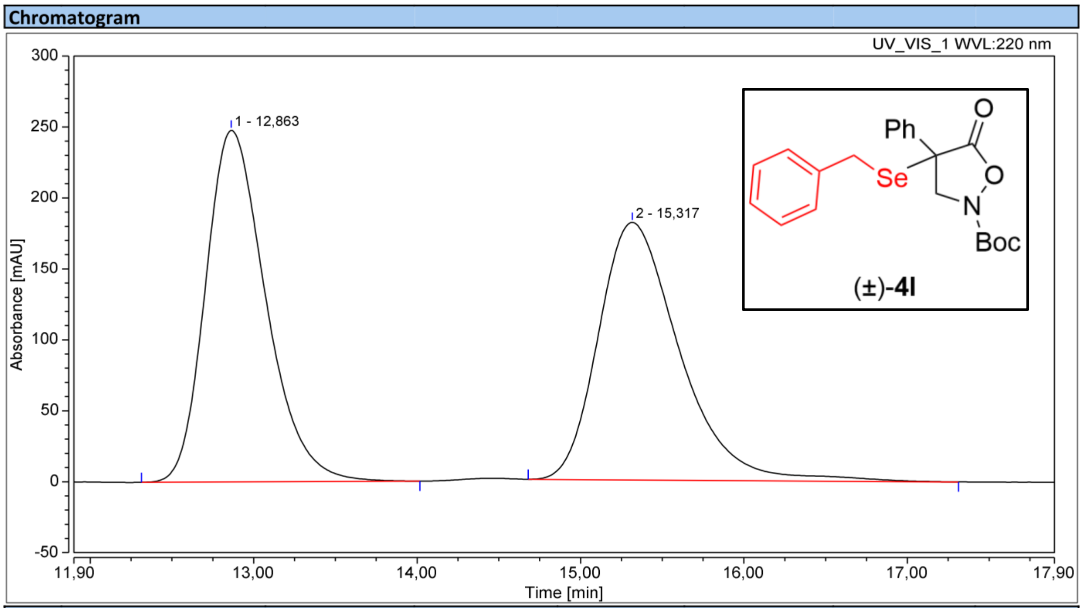

Integration Results							
No.	Peak Name	Retention Time min	Area mAU*min	Height mAU	Relative Area %	Relative Height %	Amount n.a.
1		12,863	105,874	247,867	50,28	57,72	n.a.
2		15,317	104,703	181,527	49,72	42,28	n.a.
**Total:**			**210,577**	**429,393**	**100,00**	**100,00**	

HPLC traces of **6** (Chiralpak AD-H, *n*-hexane:*i*-PrOH = 20:1, flow rate 0.9 mL/min, 10 °C, λ = 220 nm)

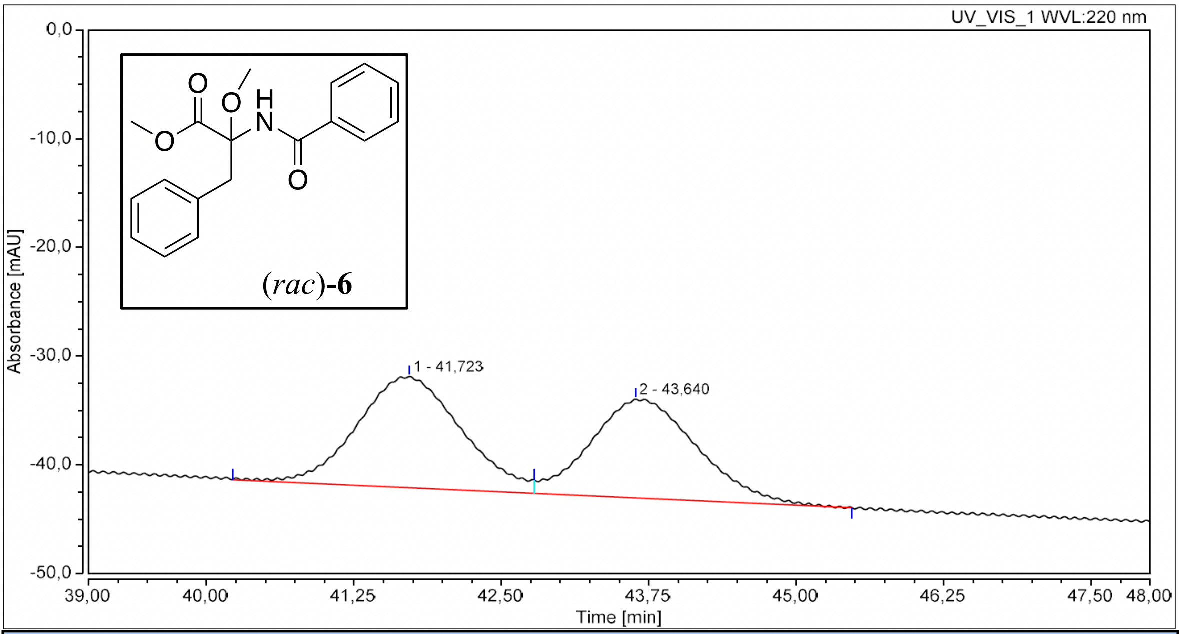

Integration Results

No.	Peak Name	Retention Time min	Area mAU*min	Height mAU	Relative Area %	Relative Height %	Amount n.a.
1		41,723	10,451	10,251	52,50	53,01	n.a.
2		43,640	9,456	9,085	47,50	46,99	n.a.
**Total:**			**19,908**	**19,336**	**100,00**	**100,00**	

HPLC traces of **9a** (Chiralpak OD-H, *n*-hexane:*i*-PrOH = 2:1, flow rate 0.5 mL/min, 20 °C, λ = 254 nm)

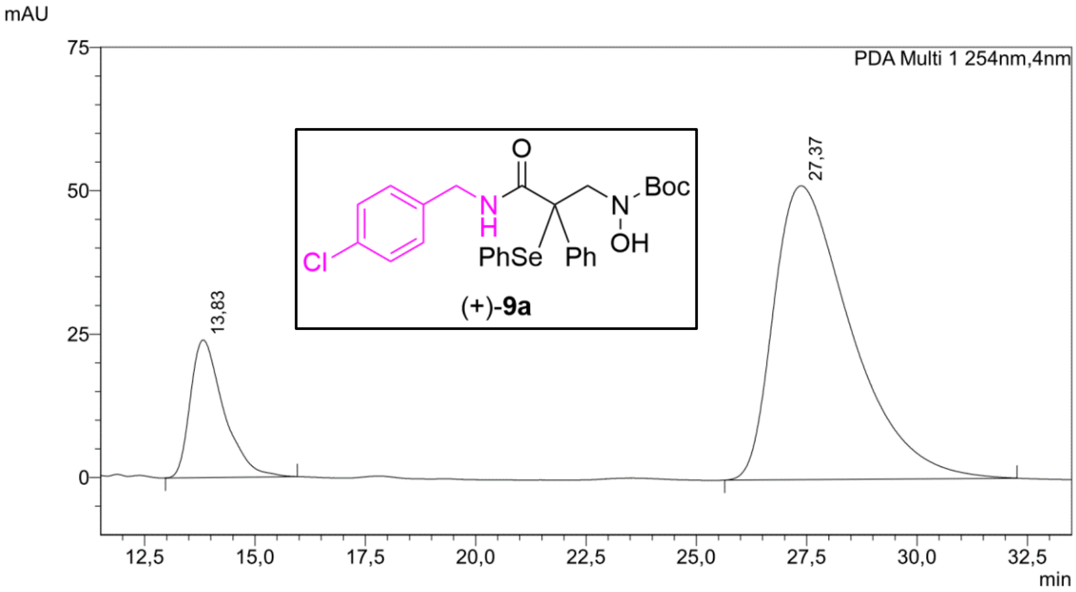

Peak Table

PDA Ch1 254nm

Peak#	Ret. Time	Area	Area%
1	13,83	1264252	16,81
2	27,37	6256611	83,19
Total		7520863	100,00

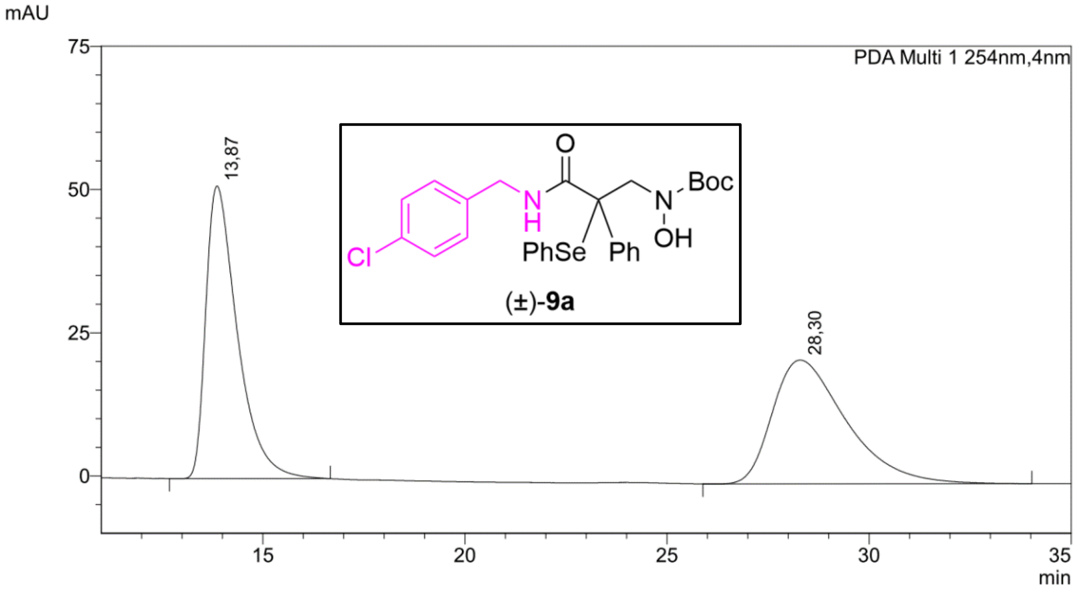

Peak Table

PDA Ch1 254nm

Peak#	Ret. Time	Area	Area%
1	13,87	2774971	50,27
2	28,30	2744791	49,73
Total		5519762	100,00

HPLC traces of **9b** (Chiralpak OD-H, *n*-hexane:*i*-PrOH = 2:1, flow rate 0.5 mL/min, 20 °C, λ = 254 nm)

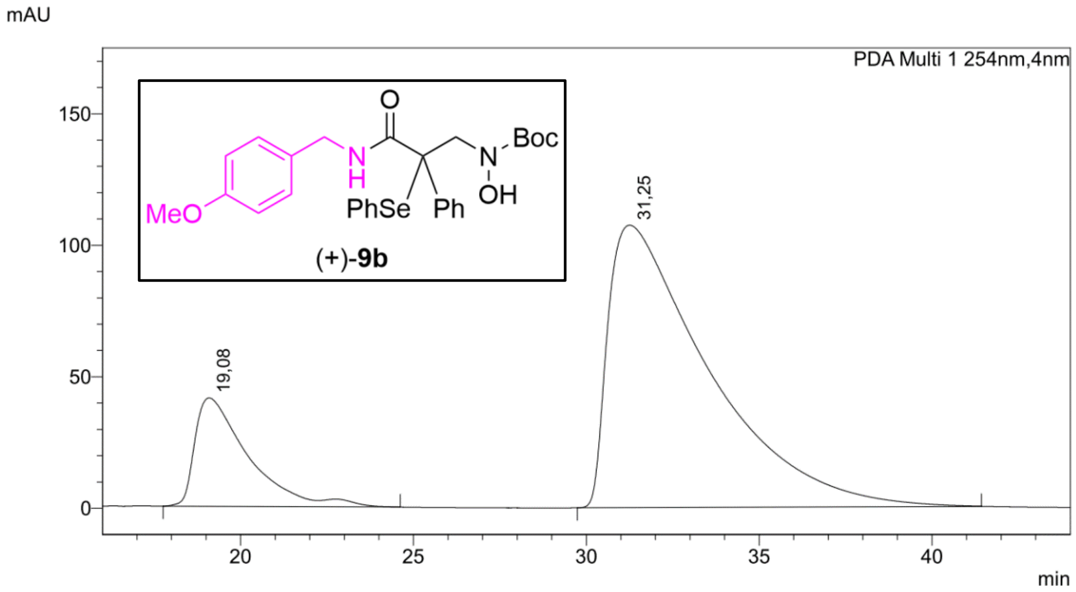

Peak Table

PDA Ch1 254nm

Peak#	Ret. Time	Area	Area%
1	19,08	4458318	17,04
2	31,25	21703836	82,96
Total		26162154	100,00

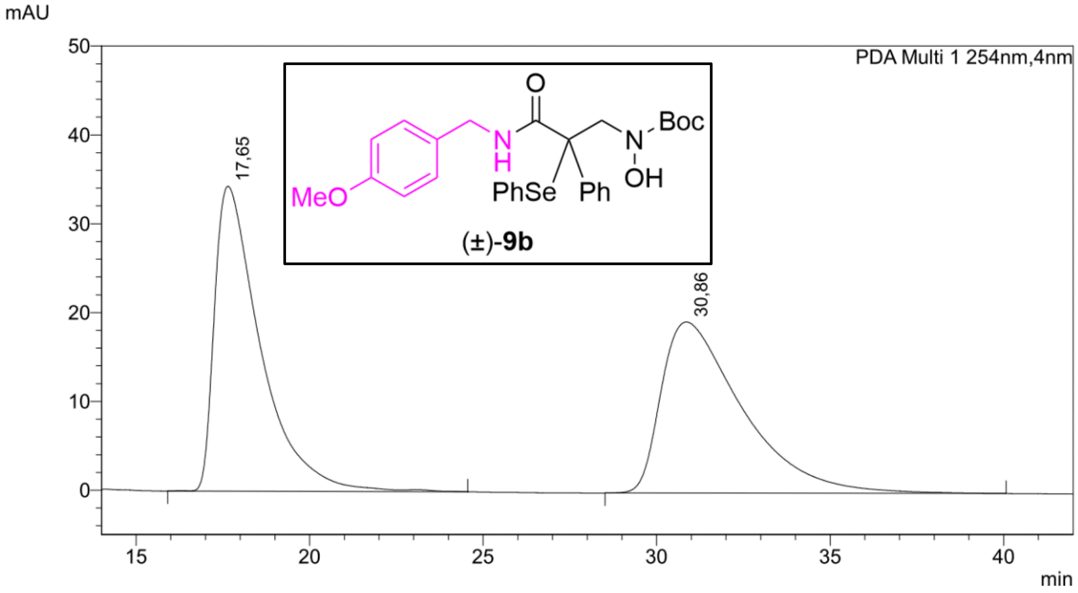

Peak Table

PDA Ch1 254nm

Peak#	Ret. Time	Area	Area%
1	17,65	3164909	50,19
2	30,86	3140800	49,81
Total		6305709	100,00

HPLC traces of **10** (YMC Chiral ART Cellulose-SB, *n*-hexane:*i*-PrOH = 4:1, flow rate 0.5 mL/min, 20 °C, λ = 254 nm)

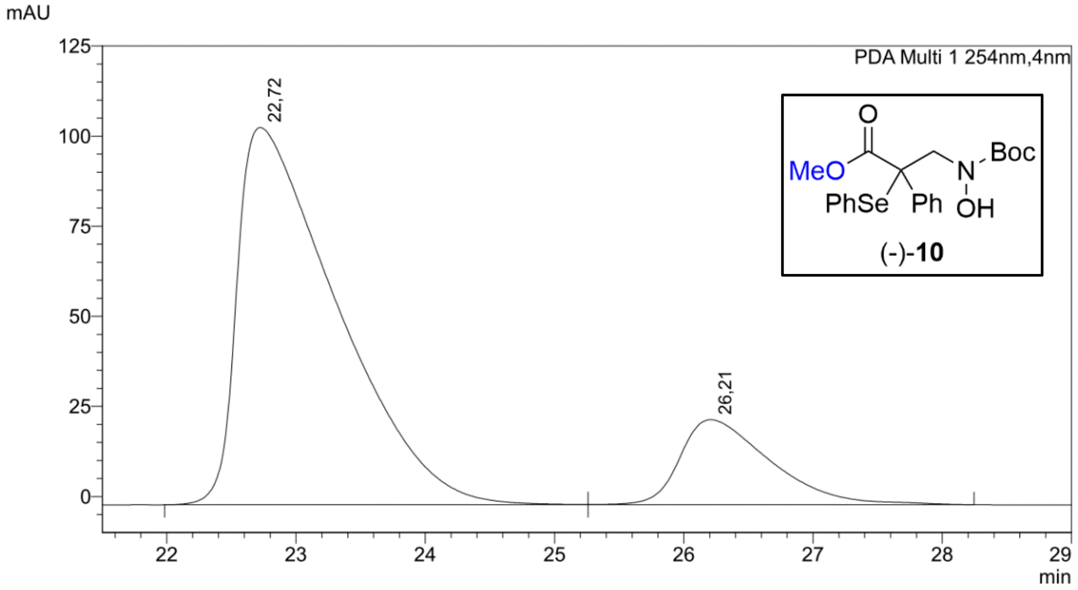

Peak Table

PDA Ch1 254nm

Peak#	Ret. Time	Area	Area%
1	22,72	5628121	83,07
2	26,21	1147292	16,93
Total		6775414	100,00

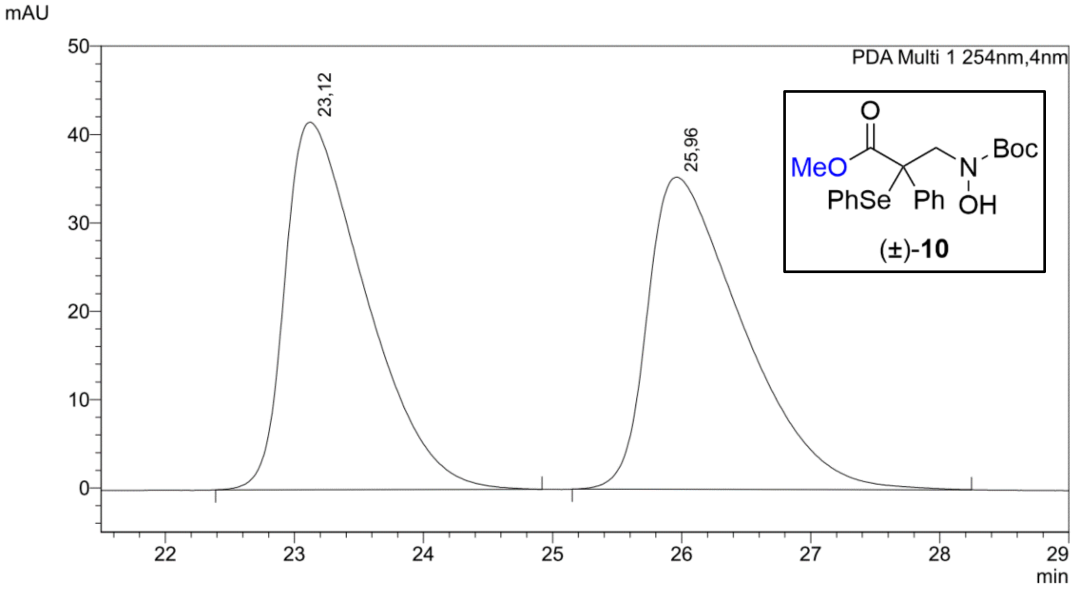

Peak Table

PDA Ch1 254nm

Peak#	Ret. Time	Area	Area%
1	23,12	1816104	49,96
2	25,96	1818670	50,04
Total		3634774	100,00

11. HRMS Data

HRMS spectrum of **3a** (ESI-QTOF, MeOH) *m/z*: $[M+H]^+$ calculated for $C_{23}H_{17}NO_2Se$, 408.0498; found, 408.0500.

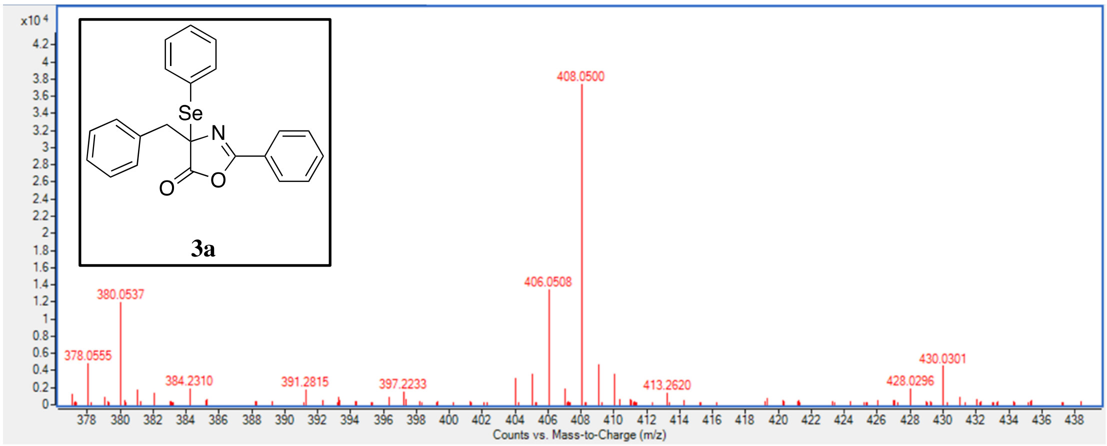

HRMS spectrum of **3c** (ESI-QTOF, MeOH) *m/z*: $[M+H]^+$ calculated for $C_{16}H_{14}NO_2Se$, 332.0185; found, 332.0187.

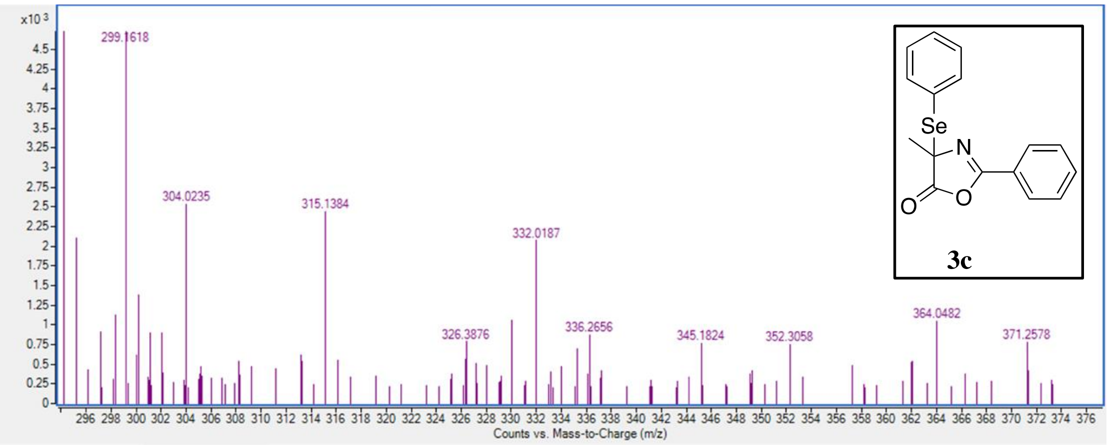

HRMS spectrum of **3d** (ESI-QTOF, MeOH) *m/z*: $[M+H]^+$ calculated for $C_{18}H_{18}NO_2Se$, 360.0498; found, 360.0501.

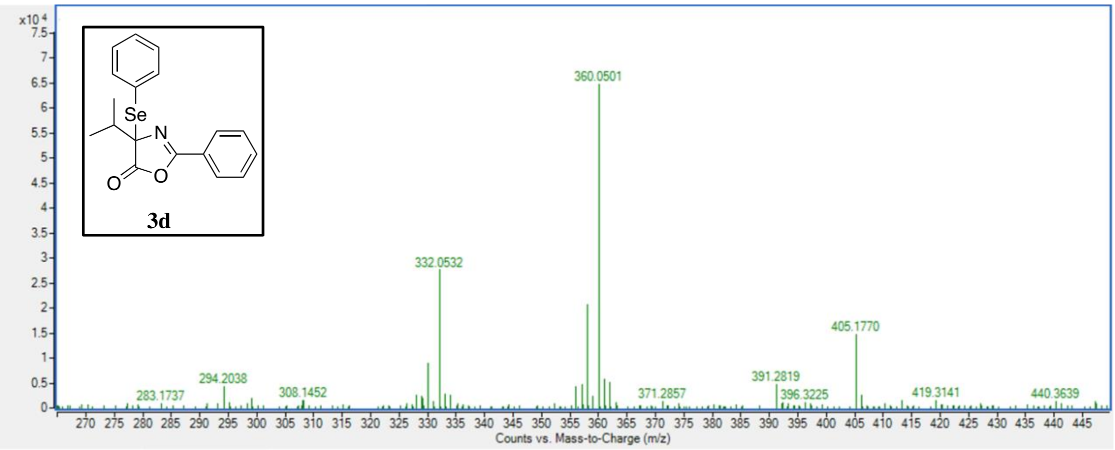

HRMS spectrum of **3e** (ESI-QTOF, MeOH) *m/z*: $[M+H]^+$ calculated for $C_{19}H_{20}NO_2Se$, 374.0654; found, 374.0657.

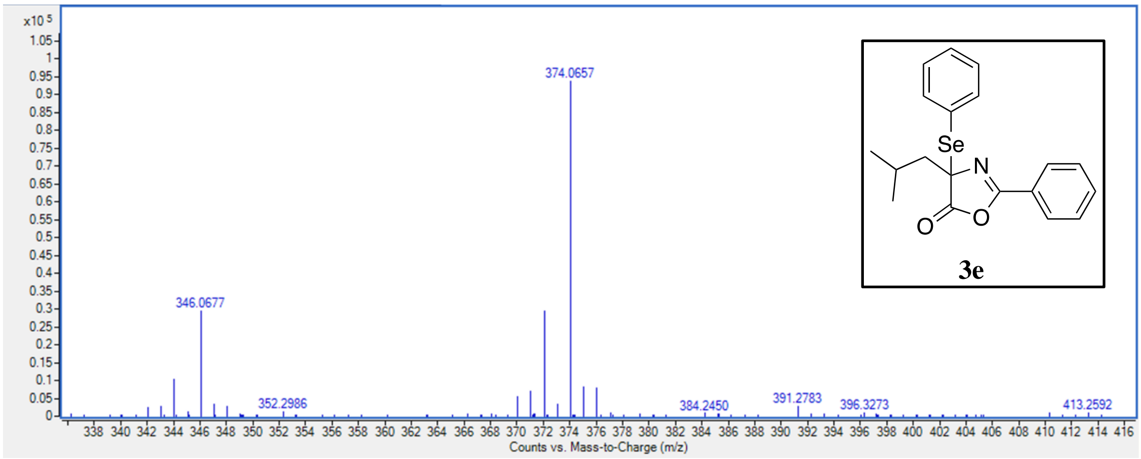

HRMS spectrum of **3f** (ESI-QTOF, MeOH) *m/z*: $[M+H]^+$ calculated for $C_{22}H_{24}NO_2Se$, 414.0967; found, 414.0965.

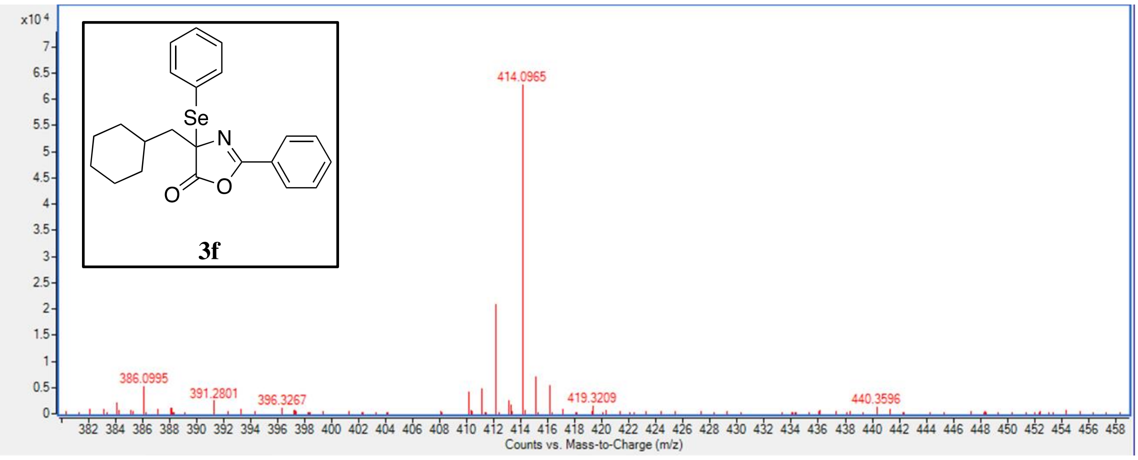

HRMS spectrum of **3g** (ESI-QTOF, MeOH) *m/z*: $[M+H]^+$ calculated for $C_{18}H_{18}NO_2SSe$, 392.0218; found, 392.0219.

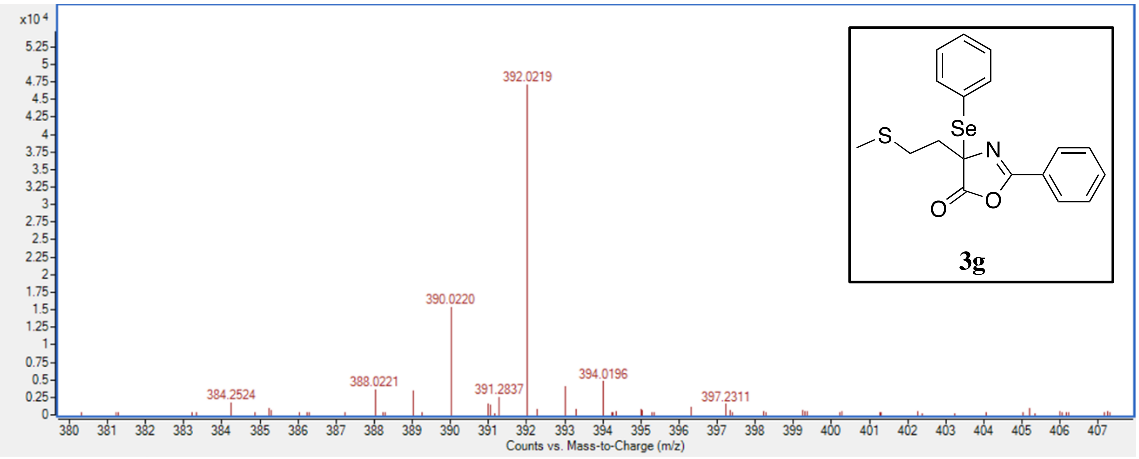

HRMS spectrum of **3h** (ESI-QTOF, MeOH) *m/z*: $[M+H]^+$ calculated for $C_{22}H_{17}ClNO_2Se$, 442.0108; found, 442.0106.

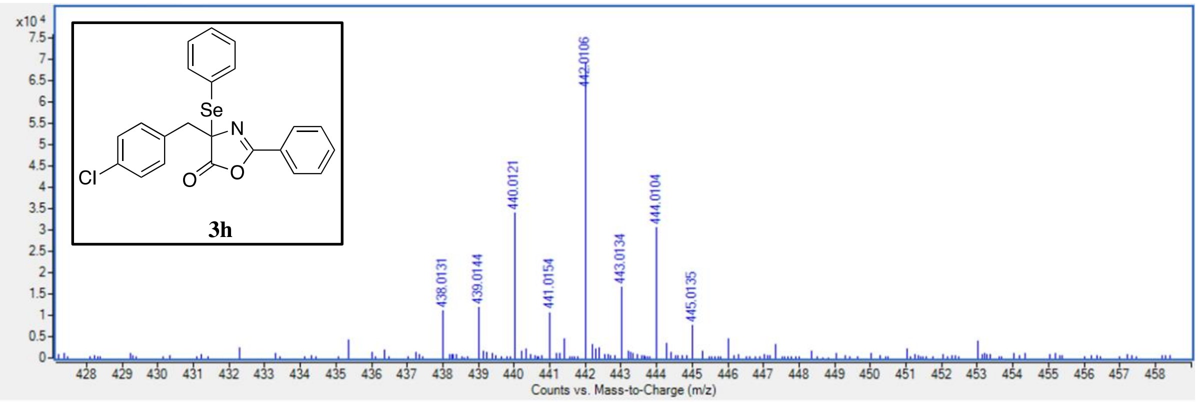

HRMS spectrum of **3i** (ESI-QTOF, MeOH) *m/z*: $[M+H]^+$ calculated for $C_{22}H_{17}N_2O_4Se$, 453.0348; found, 453.0352.

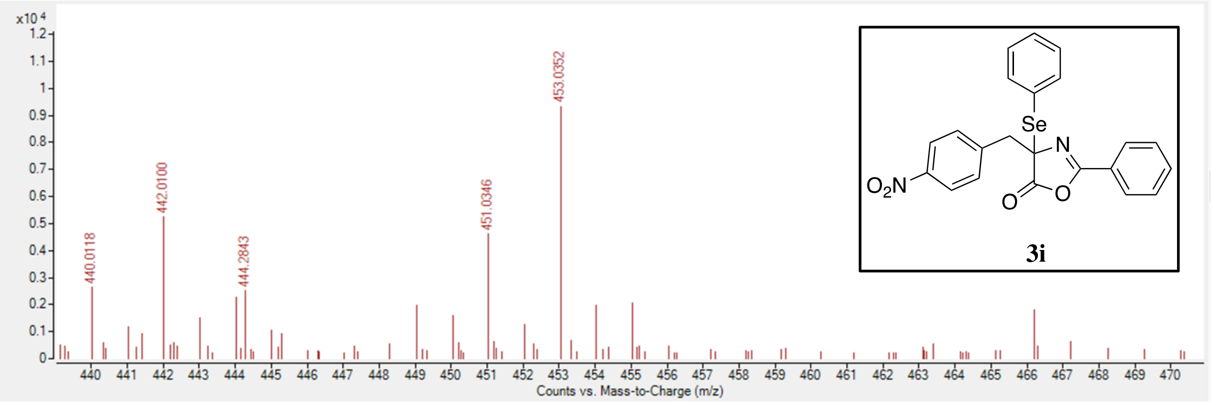

HRMS spectrum of **3j** (ESI-QTOF, MeOH) *m/z*: $[M+H]^+$ calculated for $C_{23}H_{17}F_3NO_2Se$, 476.0371; found, 476.0377.

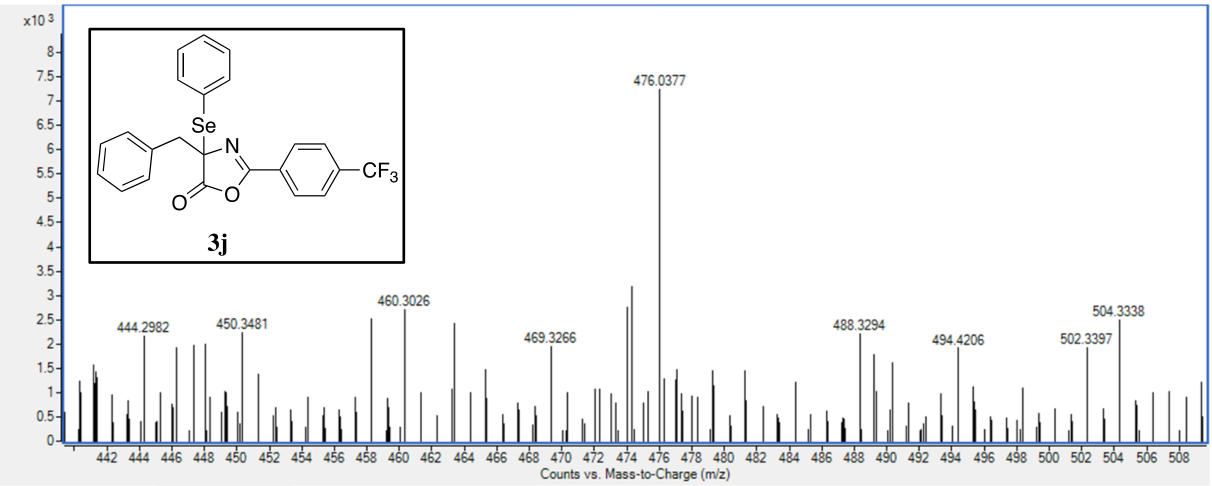

HRMS spectrum of **3k** (ESI-QTOF, MeOH) *m/z*: $[M+H]^+$ calculated for $C_{22}H_{17}N_2O_4Se$, 453.0348; found, 453.0343.

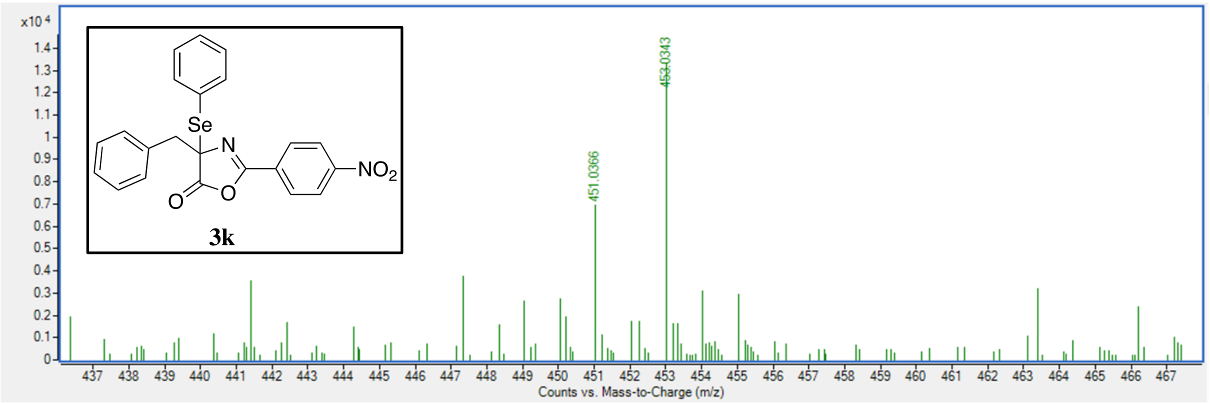

HRMS spectrum of **3l** (ESI-QTOF, MeOH) *m/z*: $[M+H]^+$ calculated for $C_{23}H_{20}NO_3Se$, 438.0603; found, 438.0605.

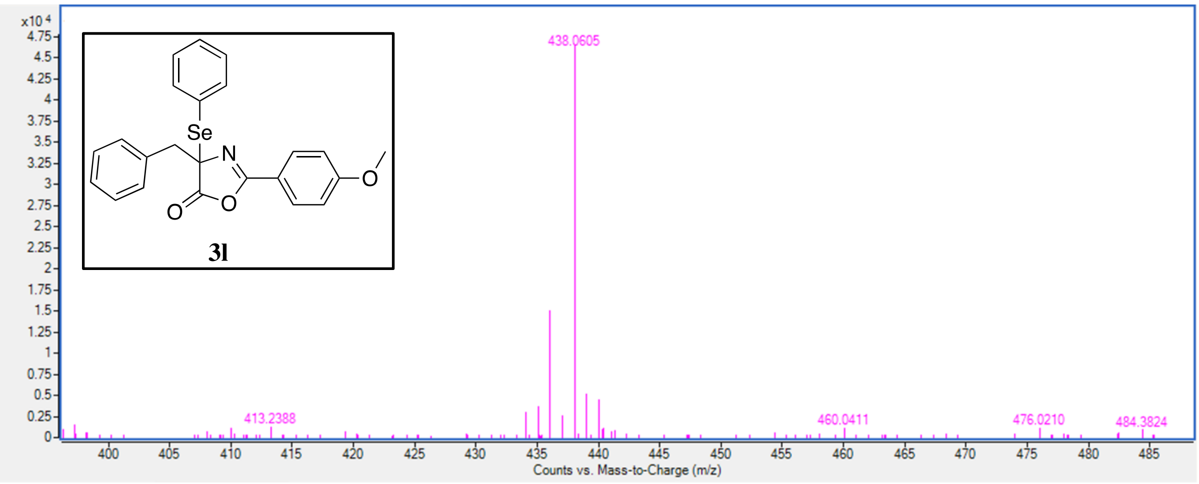

HRMS spectrum of **3m** (ESI-QTOF, MeOH) *m/z*: $[M+H]^+$ calculated for $C_{22}H_{17}ClNO_2Se$, 442.0108; found, 442.0108.

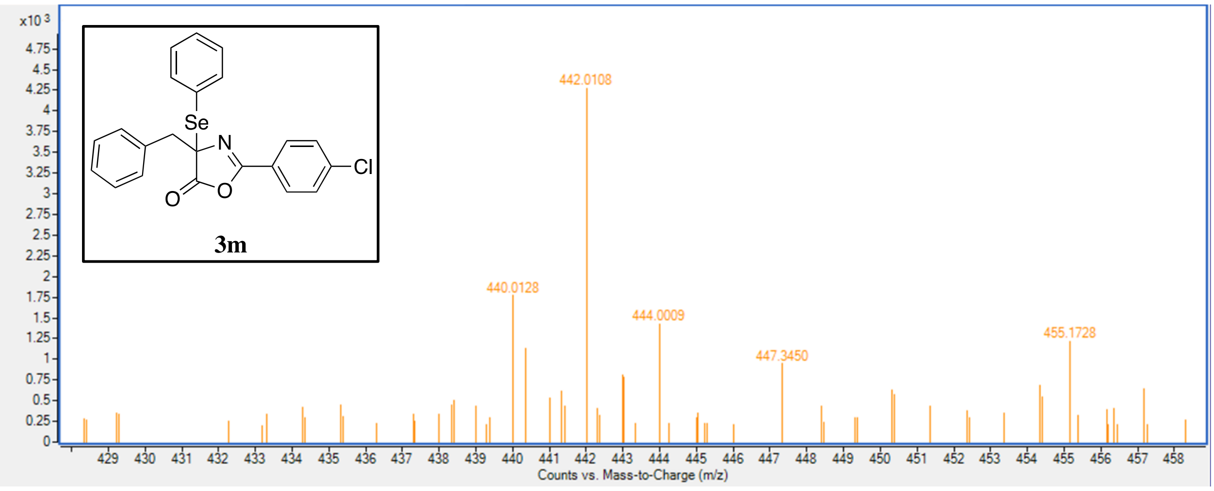

HRMS spectrum of **3n** (ESI-QTOF, MeOH) *m/z*: $[M+H]^+$ calculated for $C_{26}H_{20}NO_2Se$, 458.0654; found, 458.0649.

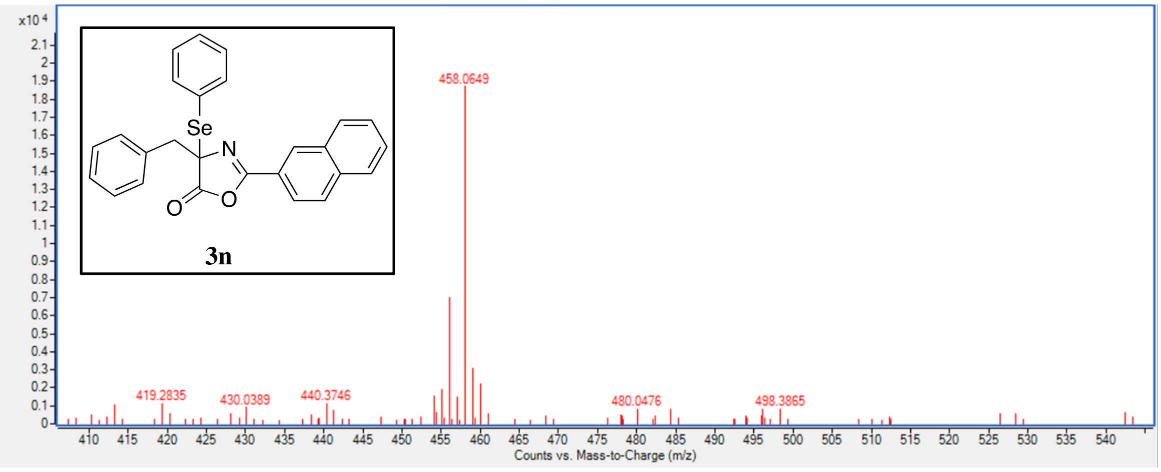

HRMS spectrum of **3p** (ESI-QTOF, MeOH) *m/z*: $[M+H]^+$ calculated for $C_{22}H_{24}NO_2Se$, 414.0967; found, 414.0963.

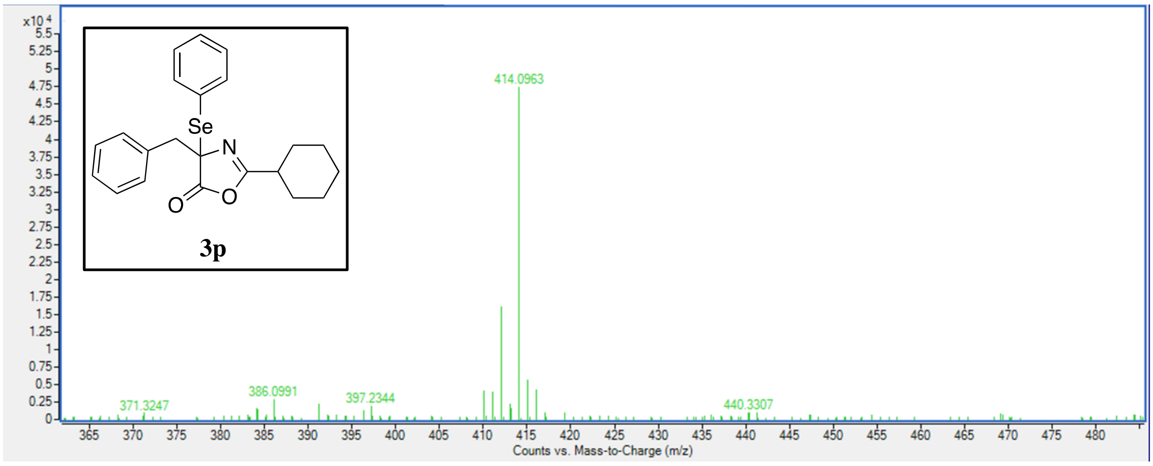

HRMS spectrum of **3q** (ESI-QTOF, MeOH) *m/z*: $[M+H]^+$ calculated for $C_{23}H_{19}ClNO_3Se$, 472.0213; found, 472.0205.

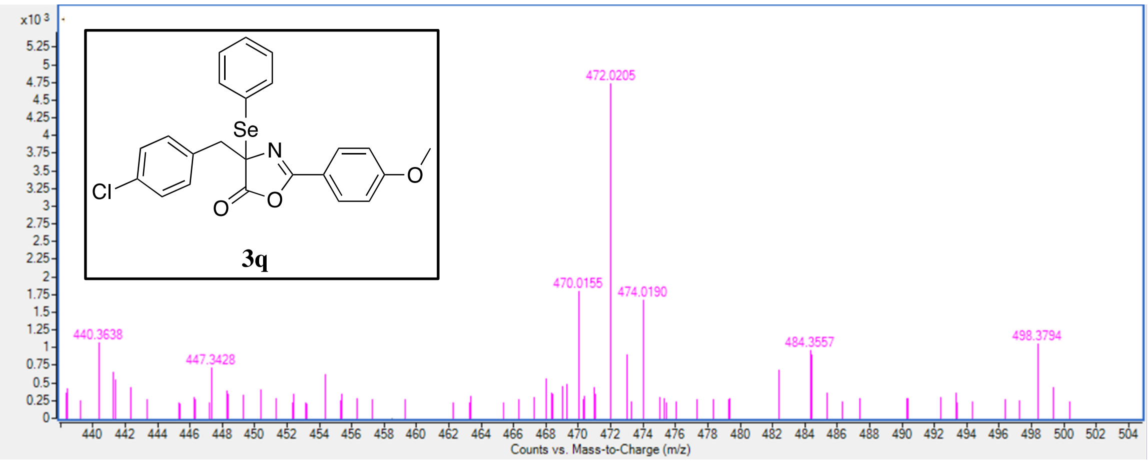

HRMS spectrum of **3r** (ESI-QTOF, MeOH) *m/z*: $[M+H]^+$ calculated for $C_{23}H_{19}N_2O_5Se$, 483.0454; found, 483.0462.

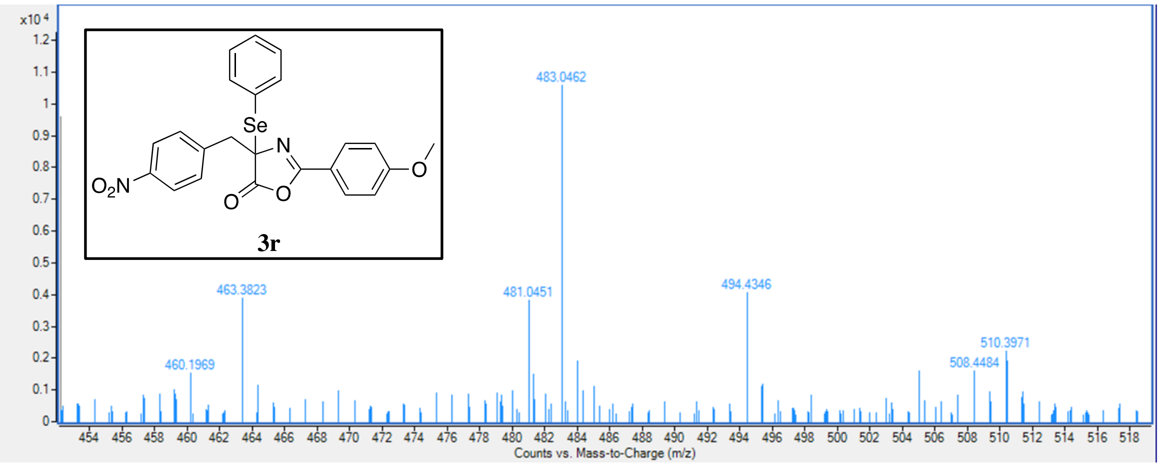

HRMS spectrum of **3s** (ESI-QTOF, MeOH) *m/z*: $[M+H]^+$ calculated for $C_{17}H_{16}NO_3Se$, 362.0290; found, 362.0294.

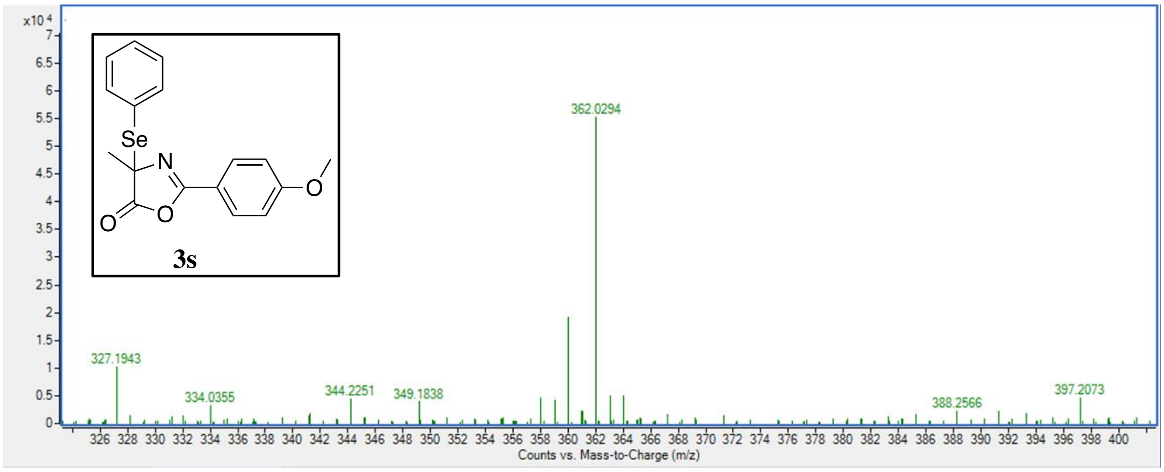

HRMS spectrum of **3t** (ESI-QTOF, MeOH) *m/z*: $[M+H]^+$ calculated for $C_{20}H_{22}NO_3Se$, 404.0760; found, 404.0770.

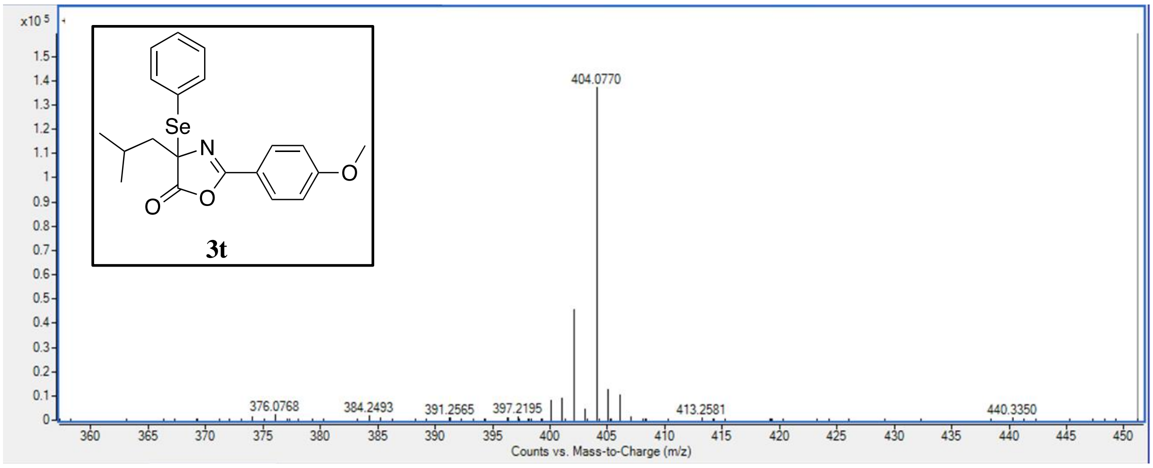

HRMS spectrum of **3u** (ESI-QTOF, MeOH) *m/z*: $[M+H]^+$ calculated for $C_{19}H_{20}NO_3SSe$, 422.0324; found, 422.0324.

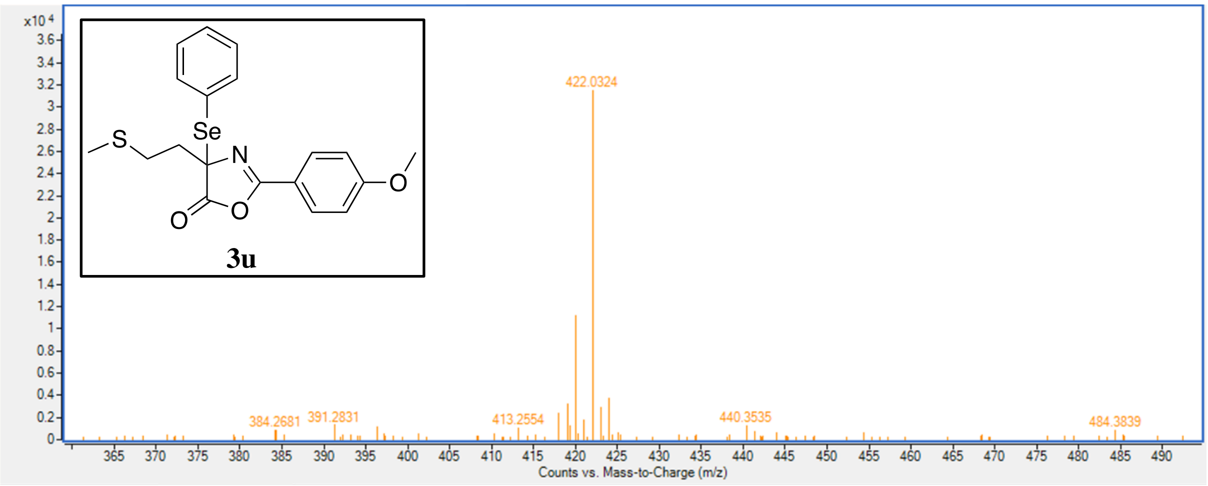

HRMS spectrum of **3v** (ESI-QTOF, MeOH) *m/z*: $[M+H]^+$ calculated for $C_{23}H_{20}NO_2Se$, 422.0654; found, 422.0650.

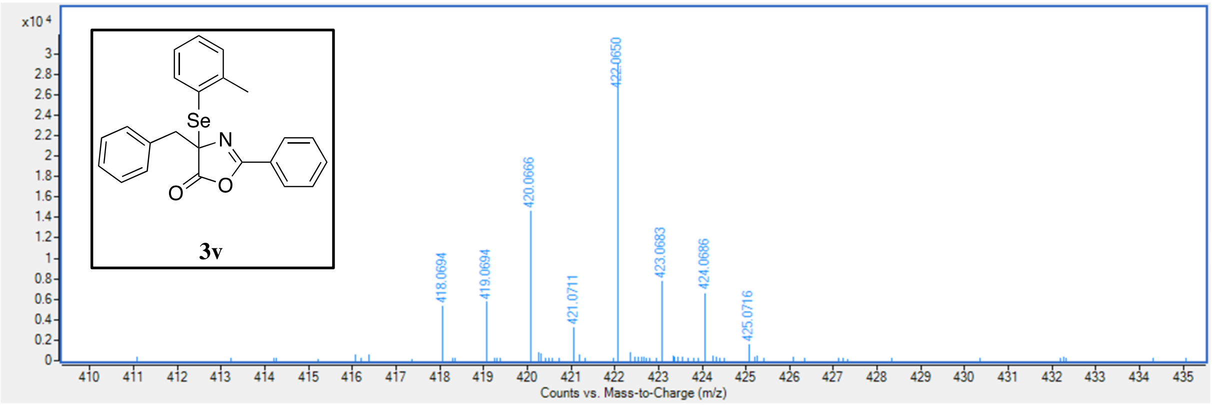

HRMS spectrum of **3w** (ESI-QTOF, MeOH) *m/z*: $[M+H]^+$ calculated for $C_{23}H_{20}NO_2Se$, 422.0654; found, 422.0658.

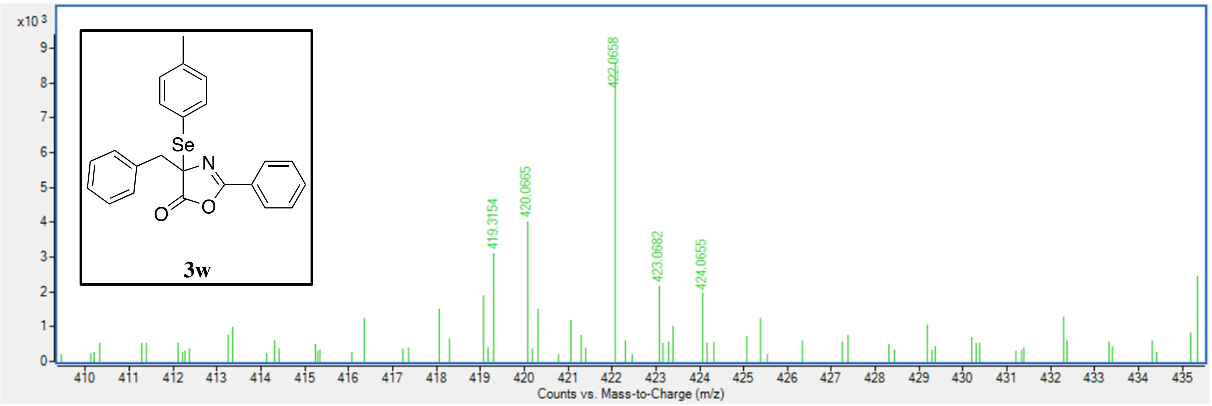

HRMS spectrum of **3x** (ESI-QTOF, MeOH) *m/z*: $[M+H]^+$ calculated for $C_{23}H_{20}NO_2Se$, 422.0654; found, 422.0652.

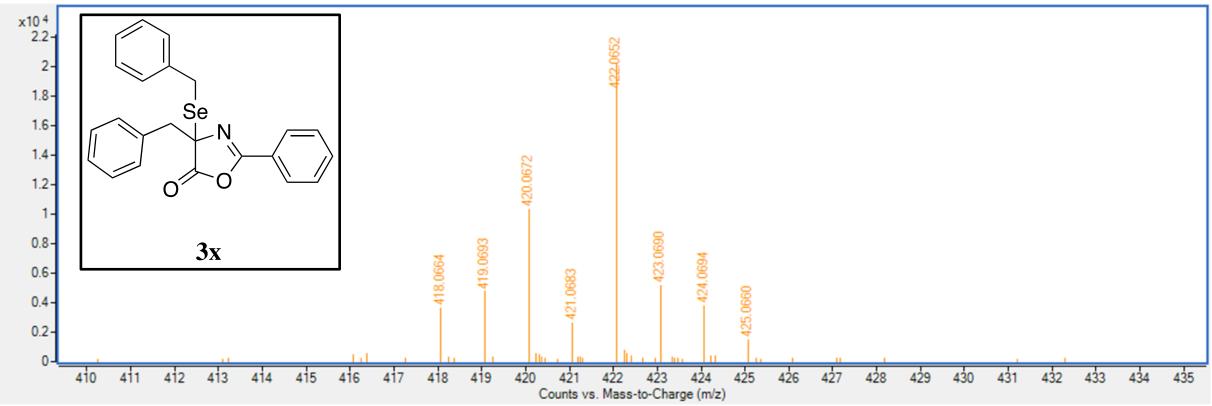

HRMS spectrum of **4a** (ESI-QTOF, MeOH) *m/z*: $[M+NH_4]^+$ calculated for $C_{20}H_{25}N_2O_4Se^+$, 437.0974; found, 437.0973.

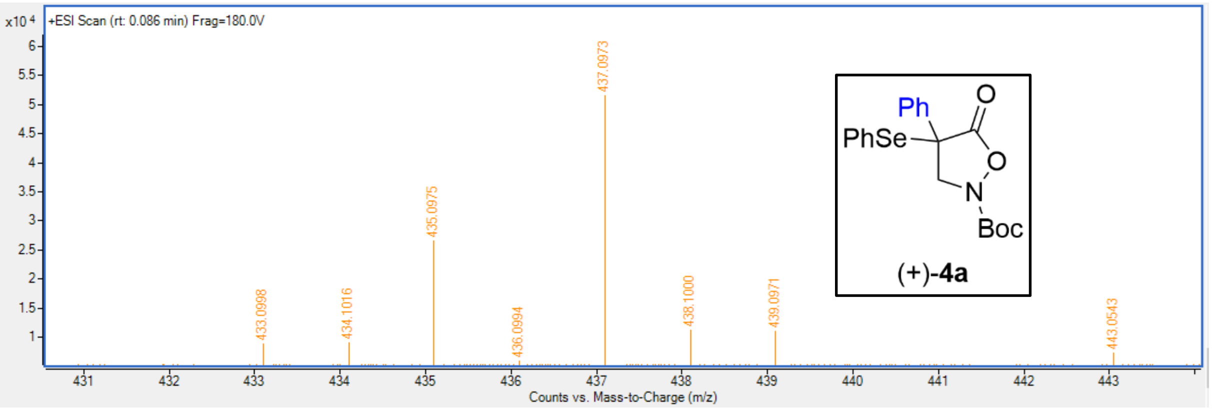

HRMS spectrum of **4b** (ESI-QTOF, MeOH) *m/z*: $[M+NH_4]^+$ calculated for $C_{24}H_{27}N_2O_4Se^+$, 487.1131; found, 487.1129.

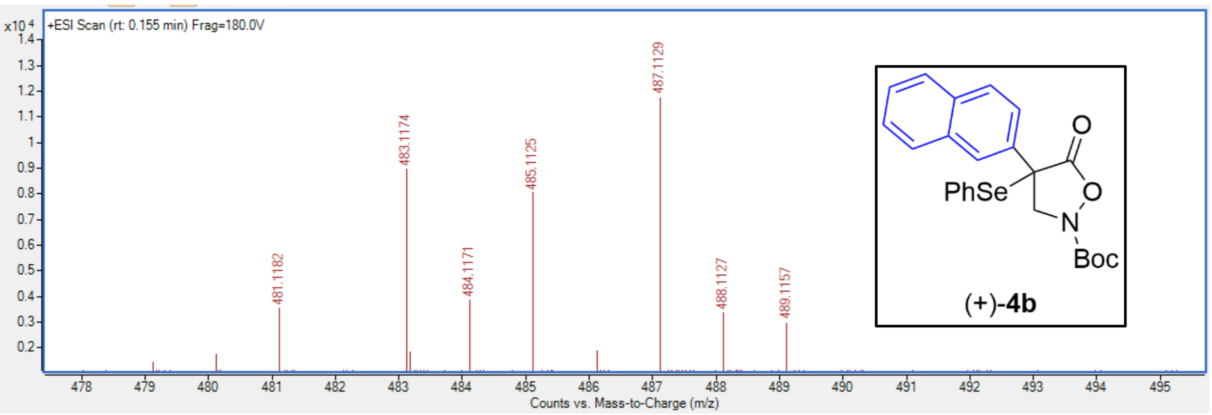

HRMS spectrum of **4c** (ESI-QTOF, MeOH) *m/z*: $[M+NH_4]^+$ calculated for $C_{18}H_{23}N_2O_4SSe^+$, 443.0538; found, 443.0538.

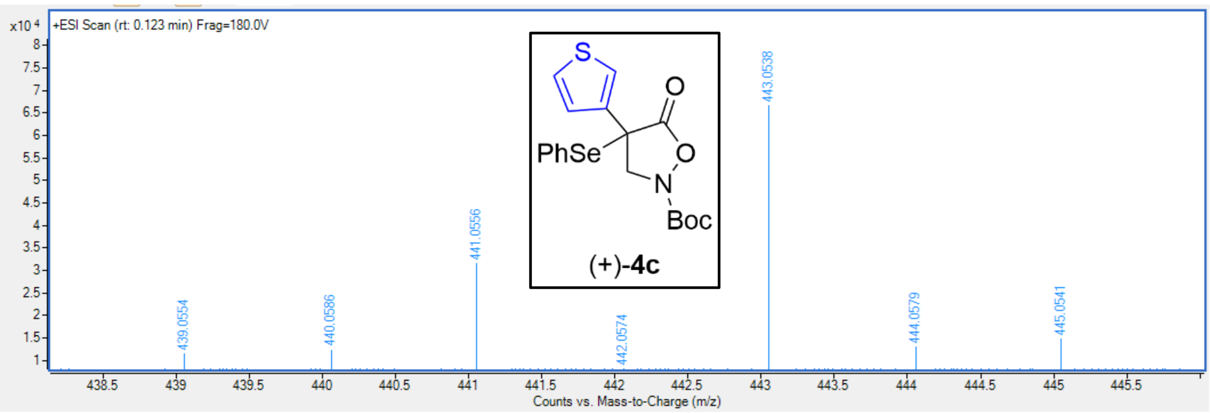

HRMS spectrum of **4d** (ESI-QTOF, MeOH) *m/z*: $[M+NH_4]^+$ calculated for $C_{20}H_{24}ClN_2O_4Se^+$, 471.0584; found, 471.0582.

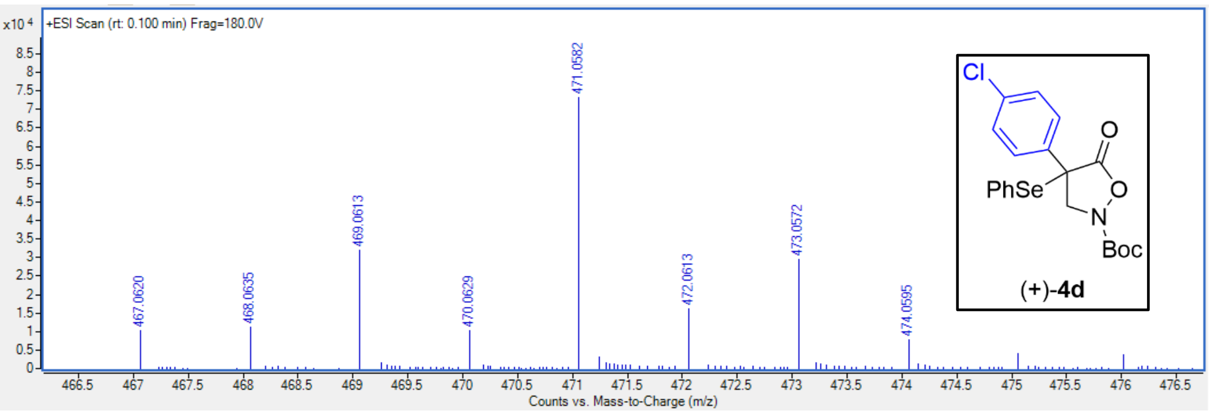

HRMS spectrum of **4e** (ESI-QTOF, MeOH) *m/z*: $[M+NH_4]^+$ calculated for $C_{20}H_{24}BrN_2O_4Se^+$, 515.0079; found, 515.0079.

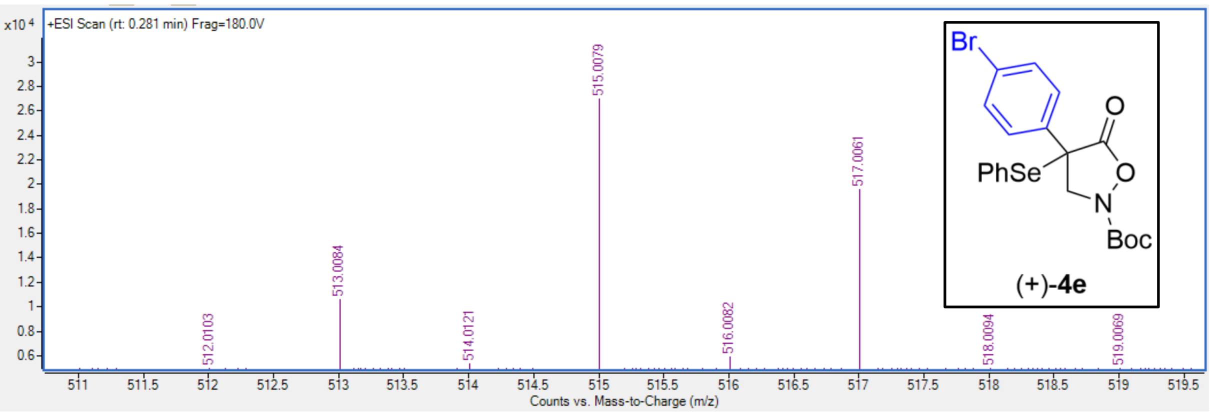

HRMS spectrum of **4f** (ESI-QTOF, MeOH) *m/z*: $[M+NH_4]^+$ calculated for $C_{20}H_{24}FN_2O_4Se^+$, 455.0880; found, 455.0881.

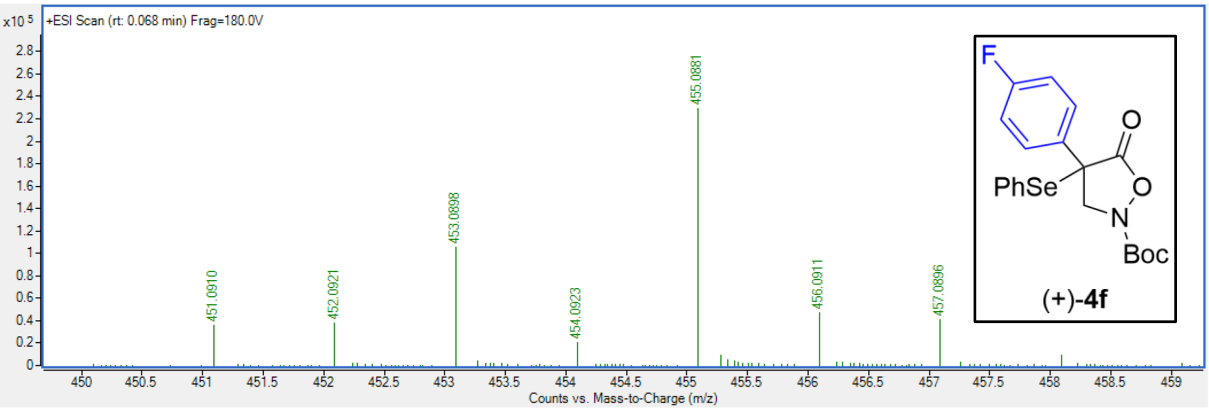

HRMS spectrum of **4g** (ESI-QTOF, MeOH) *m/z*: $[M+NH_4]^+$ calculated for $C_{20}H_{23}Cl_2N_2O_4Se^+$, 505.0195; found, 505.0195.

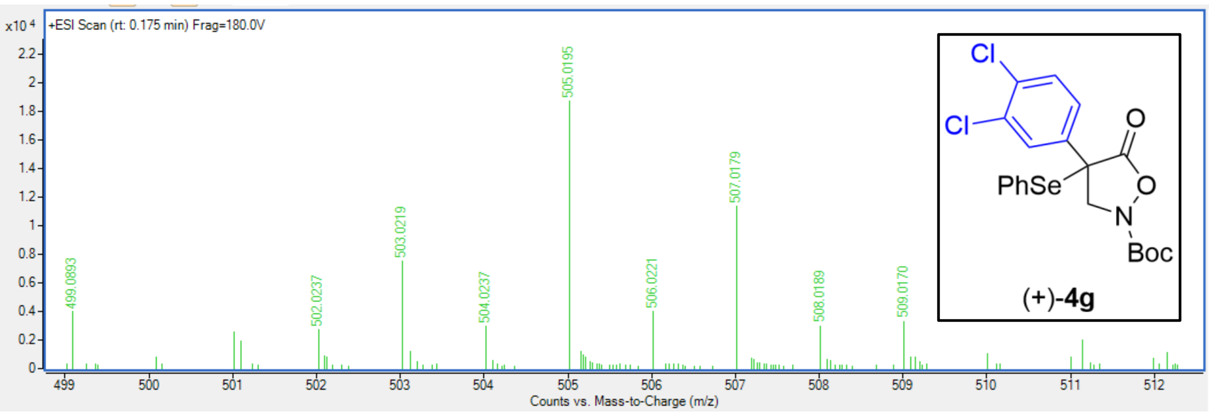

HRMS spectrum of **4i** (ESI-QTOF, MeOH) *m/z*: $[M+NH_4]^+$ calculated for $C_{21}H_{27}N_2O_4Se^+$, 451.1131; found, 451.1128.

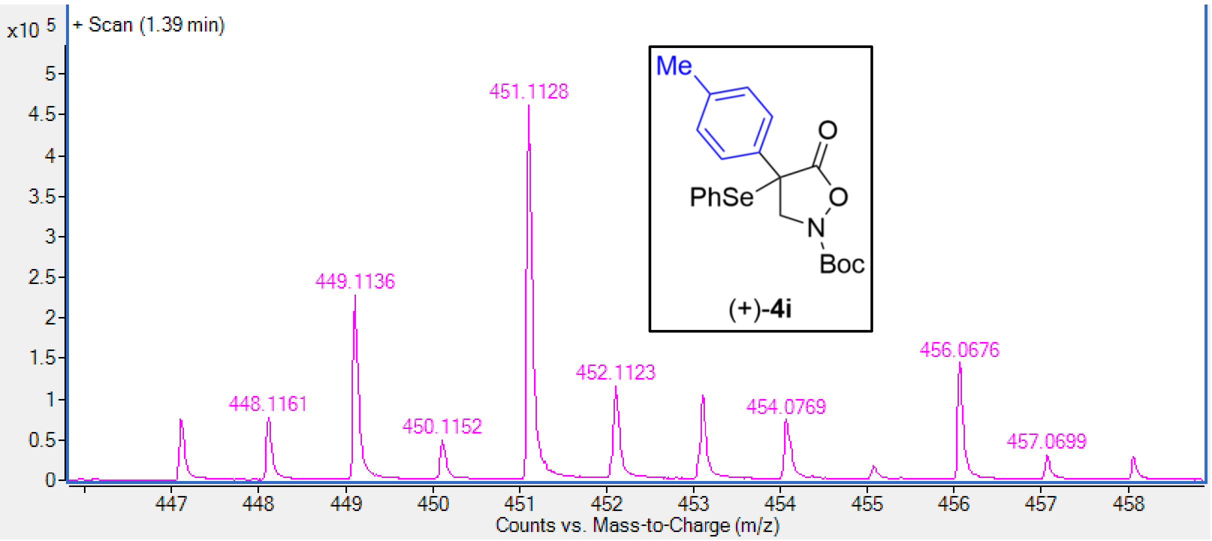

HRMS spectrum of **4j** (ESI-QTOF, MeOH) *m/z*: $[M+NH_4]^+$ calculated for $C_{21}H_{27}N_2O_4Se^+$, 451.1131; found, 451.1125.

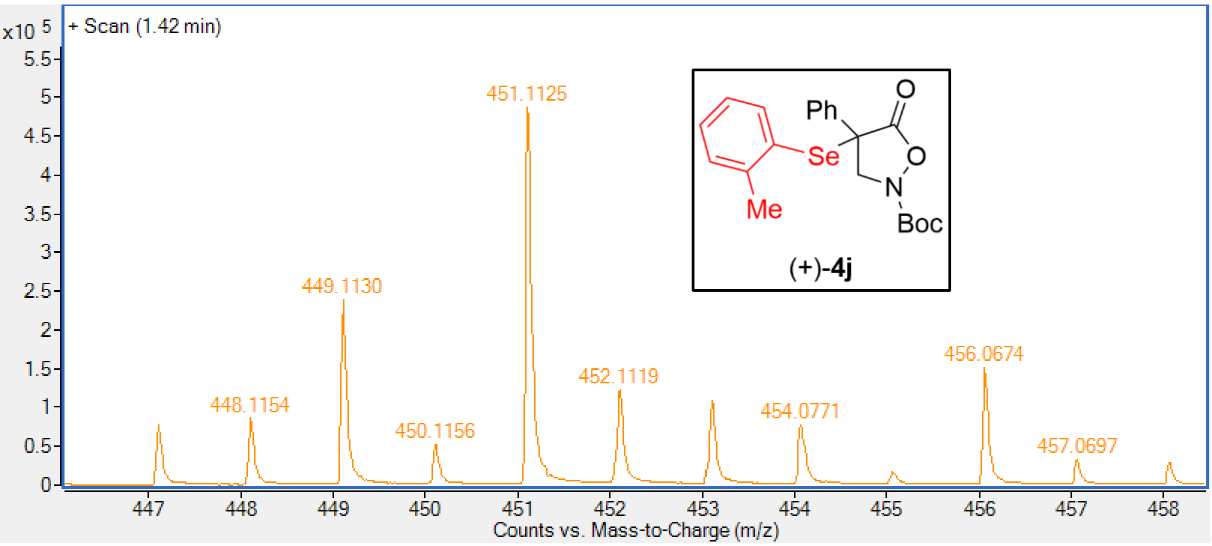

HRMS spectrum of **4k** (ESI-QTOF, MeOH) *m/z*: $[M+NH_4]^+$ calculated for $C_{21}H_{27}N_2O_4Se^+$, 451.1131; found, 451.1129.

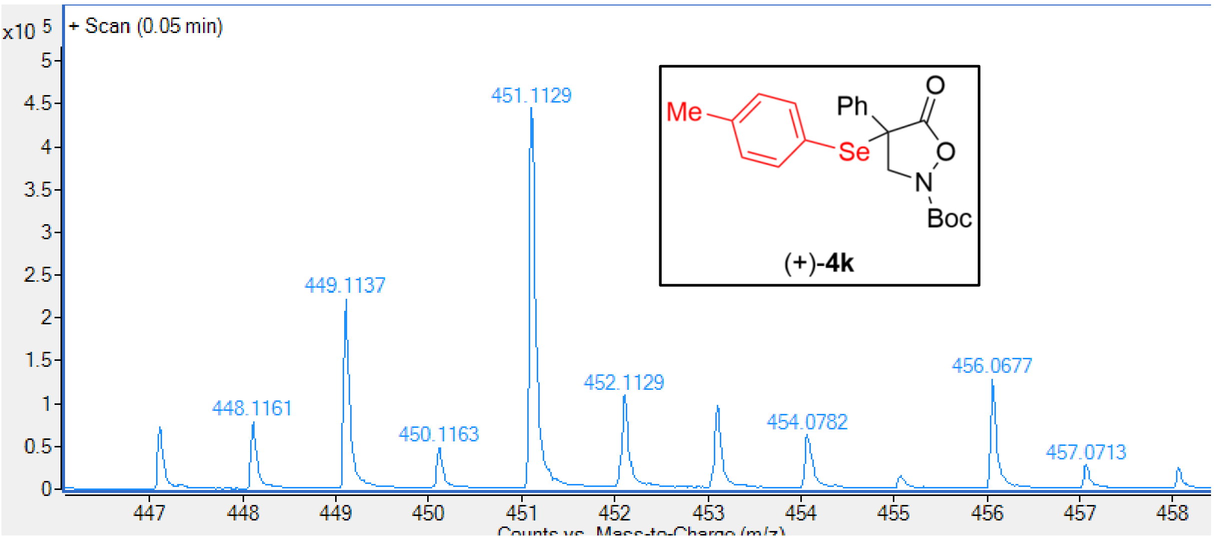

HRMS spectrum of **4l** (ESI-QTOF, MeOH) *m/z*: $[M+NH_4]^+$ calculated for $C_{21}H_{27}N_2O_4Se^+$, 451.1131; found, 451.1133.

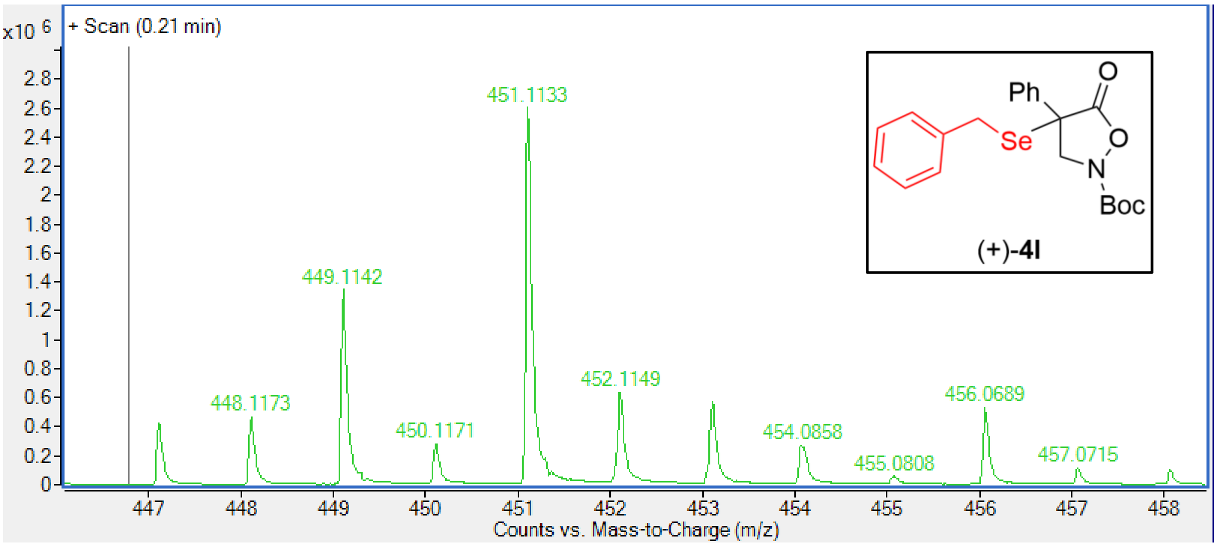

HRMS spectrum of **6** (ESI-QTOF, MeOH) *m/z*: $[M+Na]^+$ calculated for $C_{18}H_{19}NO_4Na$, 336.1206; found, 336.1207.

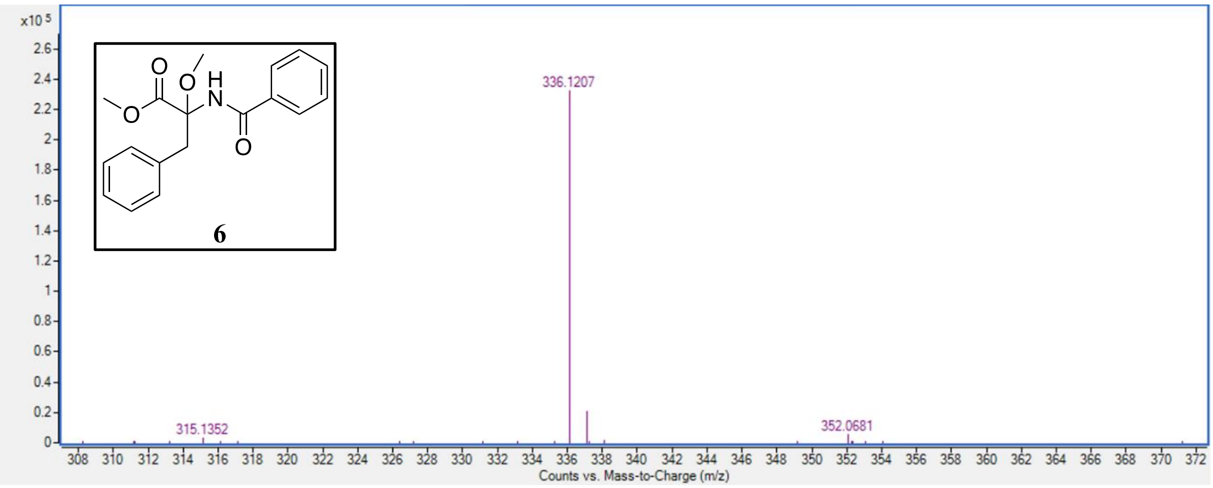

HRMS spectrum of **8** (ESI-QTOF, MeOH) *m/z*: $[M+H]^+$ calculated for $C_{23}H_{27}N_2O_4Se$, 475.1131; found, 475.1126.

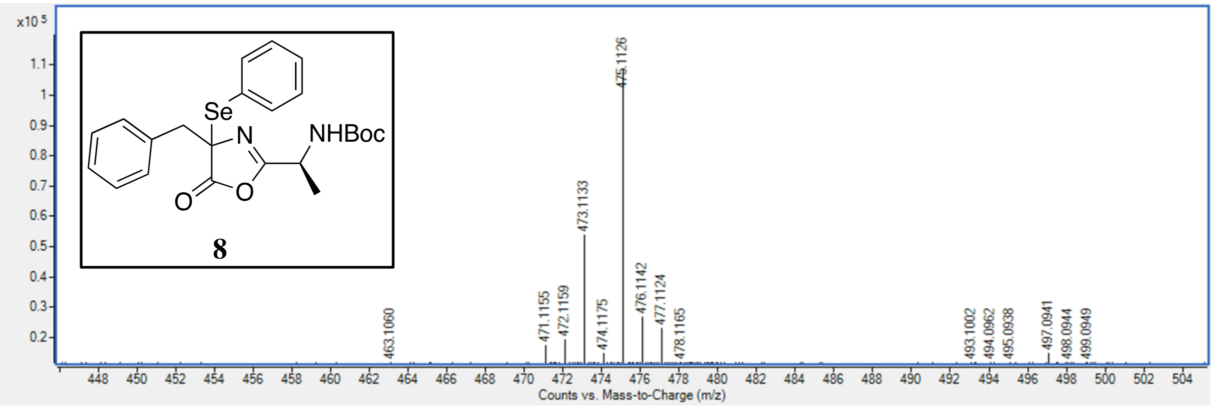

HRMS spectrum of **9a** (ESI-QTOF, MeOH) *m/z*: $[M+Na]^+$ calculated for $C_{27}H_{29}ClN_2NaO_4Se^+$, 583.0873; found, 583.0875.

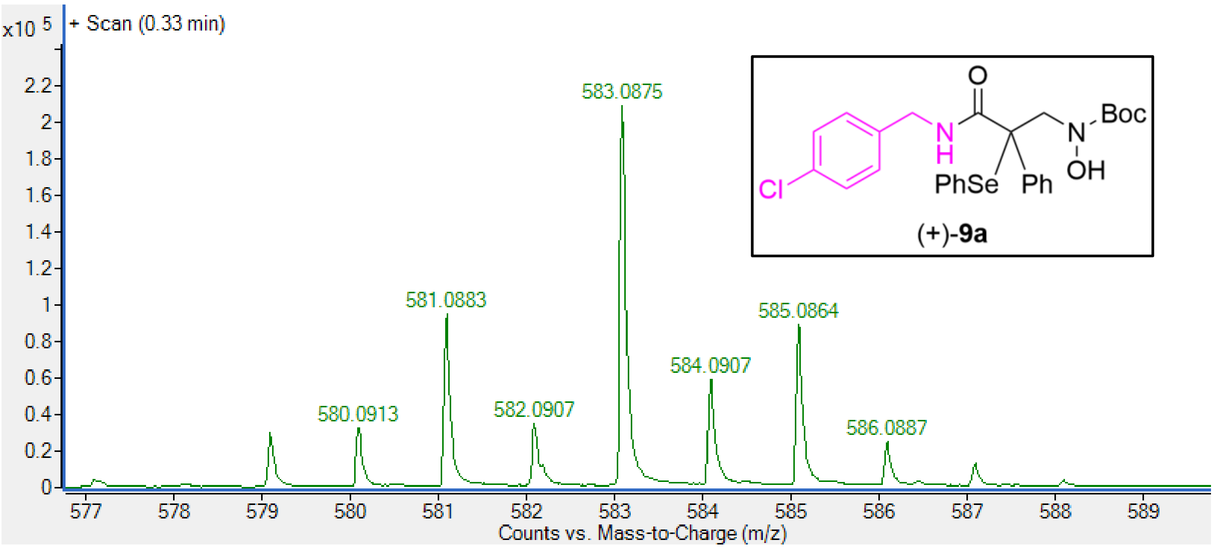

HRMS spectrum of **9b** (ESI-QTOF, MeOH) *m/z*: $[M+Na]^+$ calculated for $C_{28}H_{32}N_2NaO_5Se^+$, 579.1369; found, 579.1364.

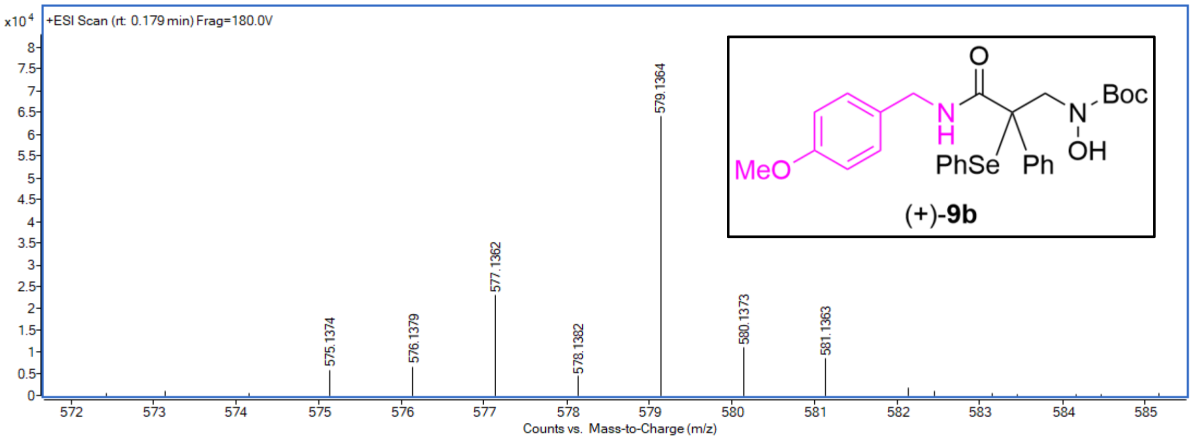

HRMS spectrum of **10** (ESI-QTOF, MeOH) *m/z*: $[M+Na]^+$ calculated for $C_{21}H_{25}NNaO_5Se^+$, 474.0790; found, 474.0795.

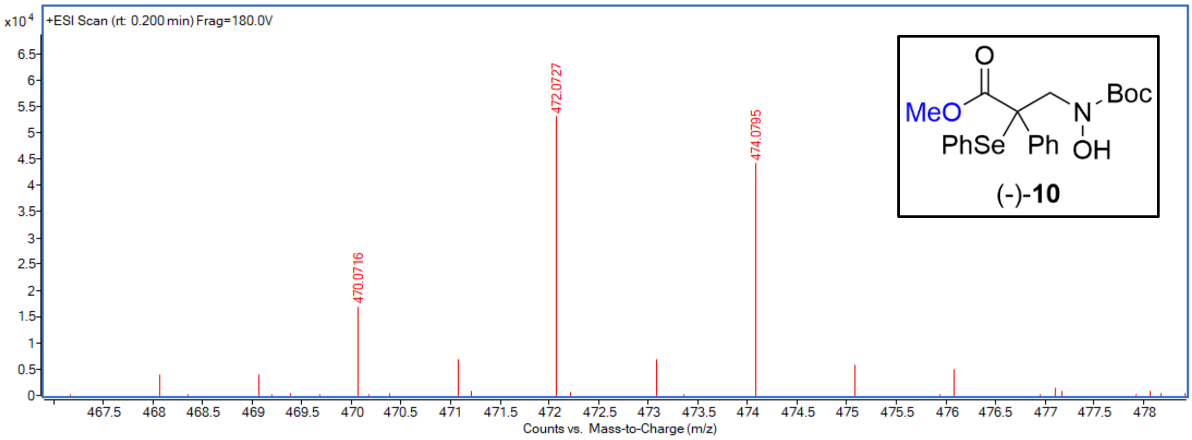

HRMS spectrum of **11** (ESI-QTOF, MeOH) *m/z*: $[M+Na]^+$ calculated for $C_{14}H_{15}NNaO_4^+$, 284.0893; found, 284.0893.

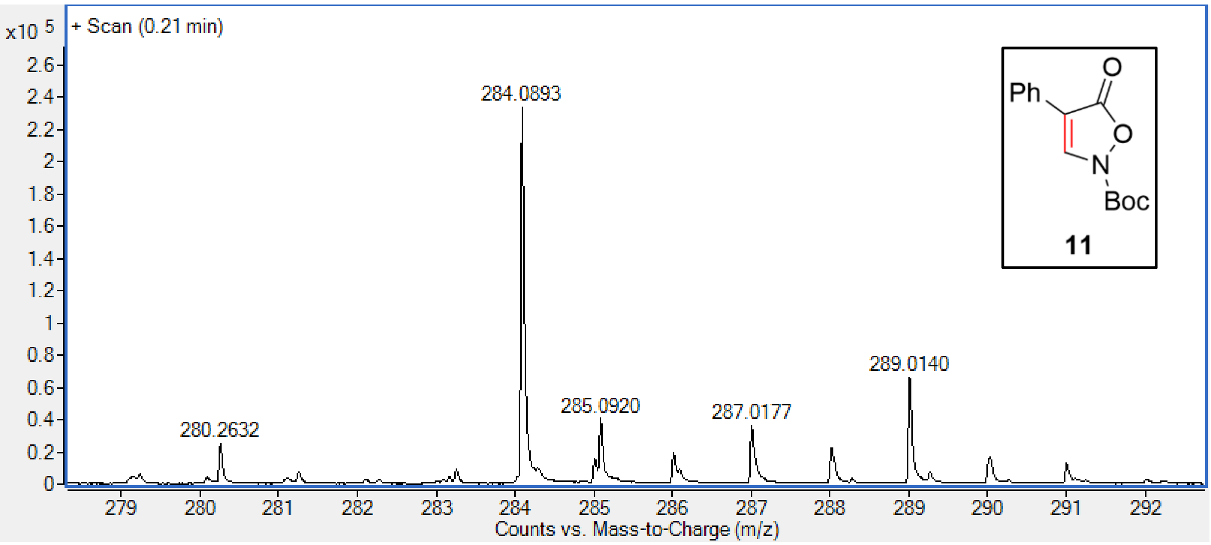

Supplement: OB-020-D1OB02235K-s001 [file OB-020-D1OB02235K-s001.pdf]
